# Trifluoromethyl Sulfoxides: Reagents for Metal‐Free C−H Trifluoromethylthiolation

**DOI:** 10.1002/anie.202005531

**Published:** 2020-07-15

**Authors:** Dong Wang, C. Grace Carlton, Masanori Tayu, Joseph J. W. McDouall, Gregory J. P. Perry, David J. Procter

**Affiliations:** ^1^ Department of Chemistry University of Manchester Oxford Road Manchester M13 9PL UK; ^2^ Department of Chemistry Meiji Pharmaceutical University 2-522-1 Noshio Kiyose Tokyo 204-8588 Japan

**Keywords:** arenes, Pummerer reaction, reaction mechanisms, sulfoxides, trifluoromethylthiolation

## Abstract

Trifluoromethyl sulfoxides are a new class of trifluoromethylthiolating reagent. The sulfoxides engage in metal‐free C−H trifluoromethylthiolation with a range of (hetero)arenes. The method is also applicable to the functionalization of important compound classes, such as ligand derivatives and polyaromatics, and in the late‐stage trifluoromethylthiolation of medicines and agrochemicals. The isolation and characterization of a sulfonium salt intermediate supports an interrupted Pummerer reaction mechanism.

## Graphical abstract

**May I interrupt?** Taking advantage of the interrupted Pummerer reaction, trifluoromethyl sulfoxides engage in the metal‐free C−H trifluoromethylthiolation of (hetero)arenes, including drug molecules and natural products. This new class of trifluoromethylthiolating reagent exploits a new strategy for trifluoromethylthiolation in which sulfonium salts are assembled and selectively deconstructed.

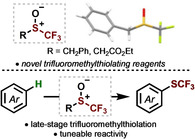

Incorporating fluorine into organic compounds is a useful tool in drug design and development. The fluoro group is well known to improve the pharmacokinetic properties of a molecule and fluorine‐18 is an important radioisotope in molecular imaging.^1^, ^2^ Trifluoromethylthio (SCF_3_) groups are commonly found in drug molecules and veterinary medicines.^3^, ^4^ By combining a fluorinated moiety with a heteroatom, many have turned to the SCF_3_ group to impart useful properties, such as high lipophilicity, to a compound of interest.^5^

An attractive route for incorporating SCF_3_ groups into organic molecules is through the direct, metal‐free functionalization of C−H bonds.^6^ Early methods using trifluoromethylsulfenyl chloride have fallen from favor because of concerns over handling and toxicity of the reagent.^7^ This triggered a push to develop shelf‐stable, easy‐to‐handle trifluoromethylthiolating agents (Scheme 1 A).^8^ Despite the advantages of these reagents, they are generally limited to the C−H trifluoromethylthiolation of highly electron‐rich (hetero)arenes, such as indoles and phenols, whereas reactions involving less nucleophilic arenes, such as anisole and toluene, are scarce.8a, 8b, 8d, 8o, 8p Furthermore, few reports describe the use of these reagents for the late‐stage trifluoromethylthiolation of complex molecules.8a, 8b, 8j, 8m, 8n

**Scheme 1:**
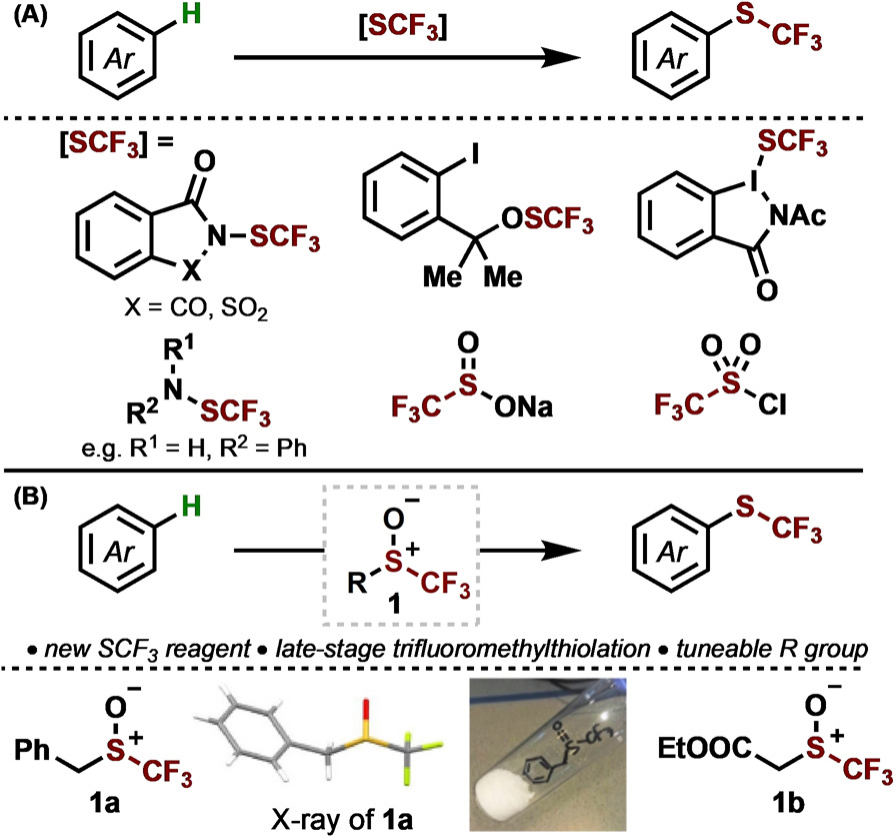
A) Current methods for transition metal‐free C−H trifluoromethylthiolation. B) This work: C−H trifluoromethylthiolation by an interrupted Pummerer reaction.

In recent years, our group^9^ and others^10^ have explored the so‐called interrupted Pummerer reaction of sulfoxides and its use for the functionalization of C−H bonds.^11^ For example, we have described the thioarylation of simple arenes using sulfoxides as sulfide precursors.9c Key to these reactions is the in situ formation of a highly electrophilic sulfonium salt, by activation of the sulfoxide with an acid anhydride, which is susceptible to reaction with a range of nucleophiles.

We were keen to assess whether underutilized trifluoromethyl sulfoxides would engage in C−H trifluoromethylthiolation. We reasoned that trifluoromethylsulfonium salts, generated from trifluoromethyl sulfoxides by an interrupted Pummerer reaction, would prove versatile intermediates en route to the incorporation of SCF_3_ into nucleophilic arenes. Herein, we present trifluoromethyl sulfoxides as novel, tuneable trifluoromethylthiolating agents (Scheme 1 B). The easy to prepare, bench‐stable and novel trifluoromethyl sulfoxides^12^ allow SCF_3_ incorporation into a variety of heteroarenes and arenes, including drug molecules, at the expense of C−H bonds. In contrast to current methods for trifluoromethylthiolation, which involve direct attack of an arene on an electrophilic SCF_3_ reagent, our unique strategy builds the desired connectivity to give sulfonium salts that are selectively deconstructed in situ to deliver trifluoromethylthiolated products.

Our first aim was to design and synthesize a sulfoxide suitable for general and selective trifluoromethylthiolation.^13^ Key to our mechanistic hypothesis for trifluoromethylthiolation is the selective loss of the R group, rather than the CF_3_ group, from the sulfoxide **1** (Scheme 1 B). As this step likely occurs by nucleophilic substitution in a sulfonium salt intermediate (see below), we identified the benzyl‐substituted trifluoromethyl sulfoxide **1 a** as a candidate for enabling trifluoromethylthiolation: the activating effect of the adjacent π‐system, combined with the inhibitory effect of fluoro groups towards incoming nucleophiles,^14^ would make the benzyl group more susceptible to removal. We developed a new route for the synthesis of **1 a**, which was obtained as a free‐flowing, bench‐stable, crystalline solid and has been characterized by X‐ray crystallographic analysis (Scheme 1 B).^15^

With a novel sulfoxide in hand, we attempted the trifluoromethylthiolation of indole (**2 a**; Scheme 2). The sulfoxide was activated using triflic anhydride^9^ to give the desired trifluoromethylthiolated indole **3 a** in 70 % yield. The reaction tolerated substitution at all positions around the indole motif [C4 (**3 b**, **3 f**, **3 h**), C5 (**3 d**, **3 e**, **3 i**), C6 (**3 c**, **3 g**), C7 (**3 j**) and C3 (**3 k**)], including various electron‐withdrawing (**3 b**–**g**) and electron‐donating (**3 i**–**k**) groups. We were pleased to find that functional groups that can undergo subsequent transformations, such as halides (**3 b**, **3 c**), nitriles (**3 d**), esters (**3 e**–**3 g**), and boronate esters (**3 h**), were well tolerated. *N*‐methyl indoles also worked well in the procedure (**3 l**–**n**). A range of other heteroaromatic compounds also underwent efficient C−H trifluoromethylthiolation, such as benzothiophene (**3 o**), thiophenes (**3 p**, **3 q**), benzofuran (**3 r**) and pyrroles (**3 s**, **3 t**). The reaction was also executed on a gram scale without severe detriment to the yield (**3 a**).

**Scheme 2:**
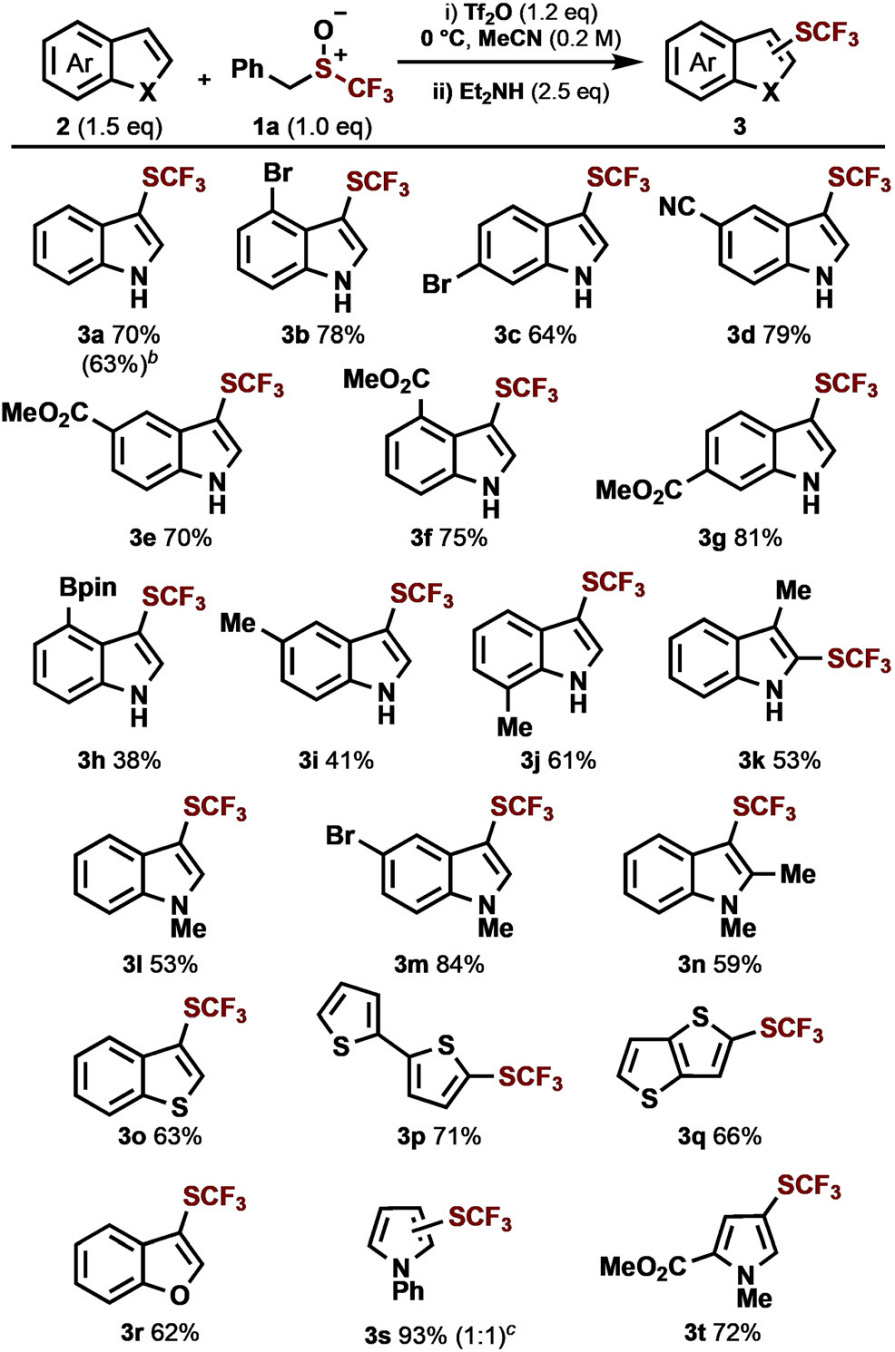
Scope^[a]^ of the metal‐free C−H trifluoromethylthiolation of heteroarenes. [a] Procedure A, conditions: i) **2** (0.3 mmol, 1.5 equiv), **1 a** (0.2 mmol, 1.0 equiv), Tf_2_O (0.24 mmol, 1.2 equiv), MeCN (1.0 mL, 0.2 m) at 0 °C for 1 h. ii) Et_2_NH (0.5 mmol, 2.5 equiv). [b] Reaction run on a gram scale. [c] Numbers within parenthesis indicate ratio of C2 versus C3 trifluoromethylthiolation.

In comparison to heteroarenes, the trifluoromethylthiolation of arenes has received less attention.8a, 8b, 8d, 8o, 8p Initial results using **1 a** gave poor yields of the desired trifluoromethylthiolated arenes, however, a novel ester‐derived trifluoromethyl sulfoxide, **1 b**, showed good reactivity (Scheme 3). This outcome suggests that the structure of the sulfoxide can be tuned for optimization with a specific class of substrate.^16^ With **1 b**, anisole, phenol and other alkylated arenes were responsive to trifluoromethylthiolation (**5 a**–**e**).^17^ Unfortunately, free amines were not tolerated in this reaction (**5 f**).^18^ A range of 1,2‐ (**5 g**–**j**) 1,3‐ (**5 k**) and 1,4‐disubstituted (**5 l**, **5 m**) arenes, bearing various functionalities, such as halogens and esters, also performed well under our reaction conditions. The reaction was also applicable to trisubstituted arenes (**5 n**, **5 o**) and naphthalenes (**5 p**, **5 q**). Finally, we showcased our method using substrates relevant in catalysis, materials, medicine, and agriculture. We were able to trifluoromethylthiolate a BINOL derivative (**5 r**), pyrene (**5 s**), drugs (**5 t**), pesticides (**5 u**), and a natural product derivative (**5 v**).

**Scheme 3:**
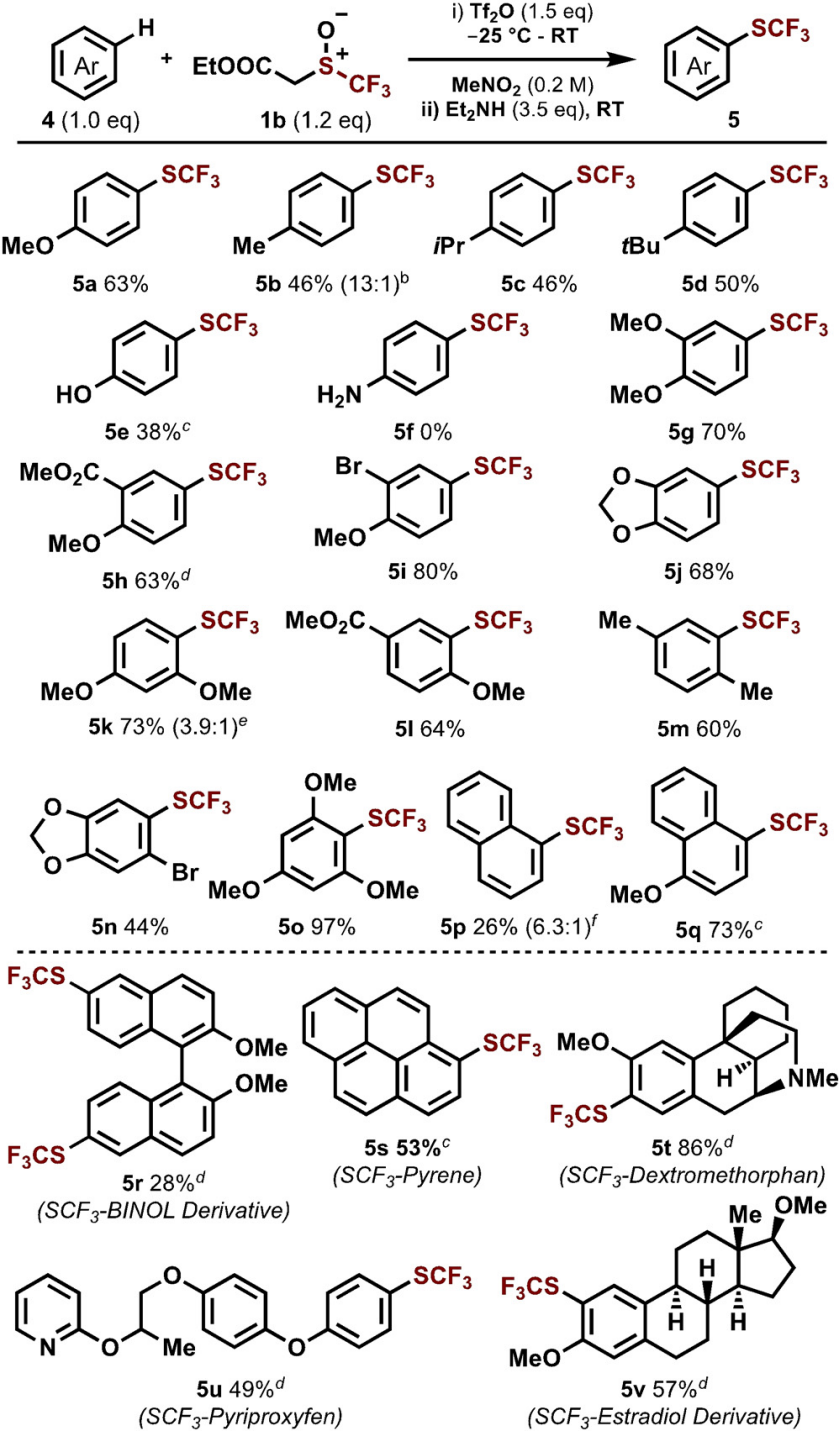
Scope^[a]^ of the metal‐free C−H trifluoromethylthiolation of arenes. [a] Procedure B, conditions: i) **4** (0.2 mmol, 1.0 equiv), **1 b** (0.24 mmol, 1.2 equiv), Tf_2_O (0.3 mmol, 1.5 equiv), MeNO_2_ (1.0 mL, 0.2 m) at −25 °C for 10 min, then at RT for 3 h. ii) Et_2_NH (0.7 mmol, 3.5 equiv) at RT for 15 h. [b] Numbers within parenthesis indicate ratio of C4 versus C2 trifluoromethylthiolation. The major regioisomer is shown. [c] Procedure A (see Scheme 2). [d] See the Supporting Information for modified reaction stoichiometry. [e] Numbers within parenthesis indicate ratio of C4 versus C2 trifluoromethylthiolation. The major regioisomer is shown. [f] Numbers within parenthesis indicate ratio of C1 versus C2 trifluoromethylthiolation. The major regioisomer is shown.

A mechanistic proposal for the trifluoromethylthiolation is summarized in Scheme 4. The trifluoromethyl sulfoxides **1** are initially activated through reaction with Tf_2_O to produce the electrophilic intermediates **6**. The intermediates **6** then undergo the so‐called interrupted Pummerer reaction with a (hetero)arene (e.g. **4**) to give the sulfonium salts **7**. Selective removal of the R group by Et_2_NH reveals the trifluoromethylthiolated products (e.g. **5**). Experimental and computational studies provided support for our proposed mechanism. Firstly, the sulfonium salt **7 m** was isolated from the reaction between *p*‐xylene (**4 m**) and **1 b**.^15^, ^19^ We then modelled the dealkylation step using DFT calculations. These results showed that the transition state for attack of the amine (Et_2_NH) at the ‐CH_2_CO_2_Et group lies 40.8 kJ mol^−1^ lower in energy than the transition state for attack at the ‐CF_3_ group. In addition, the expected side‐product, Et_2_NCH_2_CO_2_Et (**8**), was detected by GCMS. It is likely that attack at the ‐CF_3_ group is disfavored because of unfavorable electrostatic interactions,^14^ though further studies are required to fully delineate the intricacies of this mechanism. These studies highlight our unique strategy for trifluoromethylthiolation; whereas current methods proceed through direct attack of an arene on an electrophilic SCF_3_ reagent,^8^ we have introduced alternative reactivity in which the desired connectivity is built, to give **7**, before inducing controlled deconstruction and release of the desired trifluoromethylthiolated products.

**Scheme 4:**
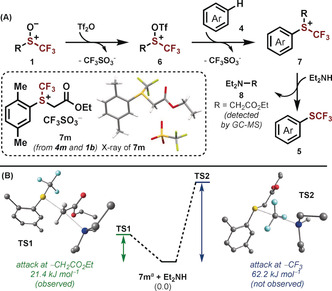
A) Proposed mechanism for the trifluoromethylthiolation of (hetero)arenes using sulfoxides. B) Computational investigation of the chemoselective dealkylation. [a] The process was modelled using the cation of **7 m**. See the Supporting Information for further details.

In summary, we have developed a new strategy for the metal‐free C−H trifluoromethylthiolation of (hetero)arenes. In this process, we utilize the interrupted Pummerer reaction to establish trifluoromethyl sulfoxides as novel trifluoromethylthiolating agents. Our method for incorporating SCF_3_ components exploits a build‐up/deconstruct strategy and is mechanistically distinct from current processes. A variety of (hetero)aromatic compounds underwent efficient trifluoromethylthiolation, including drug molecules and natural products. We expect trifluoromethyl sulfoxides to find application in other trifluoromethylthiolation reactions in the future.

## Conflict of interest

The authors declare no conflict of interest.

## Supporting information

As a service to our authors and readers, this journal provides supporting information supplied by the authors. Such materials are peer reviewed and may be re‐organized for online delivery, but are not copy‐edited or typeset. Technical support issues arising from supporting information (other than missing files) should be addressed to the authors.

Supplementary
