# Supplementary material for: Trifluoromethyl Sulfoxides: Reagents for Metal‐Free C−H Trifluoromethylthiolation
Source: Angew Chem Int Ed Engl. 2020 Jul 15;59(37):15918–22. doi: 10.1002/anie.202005531 (PMC7540508; doi:10.1002/anie.202005531)

Angewandte Chemie
Eine Zeitschrift der Gesellschaft Deutscher Chemiker

Supporting Information

Trifluoromethyl Sulfoxides: Reagents for Metal-Free C−H Trifluoromethylthiolation

*Dong Wang, C. Grace Carlton, Masanori Tayu, Joseph J. W. McDouall, Gregory J. P. Perry, and David J. Procter**

anie_202005531_sm_miscellaneous_information.pdf

Table of Contents

General Information S2
1. Reaction Optimization S3
1.1. Optimization of the C–H trifluoromethylthiolation of heteroarenes S3
1.2. Optimization of the C–H trifluoromethylthiolation of arenes S4
2. Sulfoxide Synthesis S5
2.1. Synthesis of (((trifluoromethyl)sulfinyl)methyl)benzene (**1a**) S5
2.2. Synthesis of ethyl 2-((trifluoromethyl)sulfinyl)acetate (**1b**) S6
3. General Procedure for the C–H Trifluoromethylthiolation S7
4. Substrate Scope of the C–H Trifluoromethylthiolation S8
5. Unsuccessful Substrates S23
6. Isolation of 7m S24
7. X-Ray Structures of 1a and 7m S25
8. Computational Studies S27
9. References S34
10. ^{1}H, ^{13}C, and ^{19}F NMR Spectra of Synthesized Compounds S35

General Information

All experiments were performed under an atmosphere of nitrogen, using anhydrous solvents, unless stated otherwise. Commercial anhydrous acetonitrile, dichloromethane, and nitromethane ($MeNO_2$) were used. $AgSCF_3$ was prepared according to a previously reported method[1]. All other solvents and reagents were purchased from commercial sources and used as supplied. ^{1}H, ^{13}C, and ^{19}F NMR spectra were recorded on 400 MHz (^{1}H: 400 MHz; ^{13}C 101 MHz; ^{19}F: 376 MHz) and 500 MHz (^{1}H: 500 MHz; ^{13}C: 126 MHz; ^{19}F: 471 MHz) spectrometers, using the residual solvent peak of $CDCl_3$ (^{1}H: $\delta = 7.26$; ^{13}C: $\delta = 77.00$) as an indirect reference to trimethylsilane (TMS). Chemical shift values are reported in parts per million (ppm) and coupling constants (J) are reported in Hertz (Hz). Abbreviations for NMR data are s (singlet), d (doublet), t (triplet), q (quartet), and m (multiplet). Infrared (IR) spectra were recorded as evaporated films or neat on a FT/IR spectrometer. Mass spectra were obtained using positive or negative atmospheric pressure chemical ionization (APCI), atmospheric pressure photoionization (APPI), atmospheric solid analysis probe (ASAP) techniques, or heated electrospray ionization (HESI). Column chromatography was carried out using silica gel 60 Å, 240-400 mesh. Thin layer chromatography (TLC) was performed on aluminium sheets pre-coated with silica gel, 0.20 mm (Macherey-Nagel, Polygram® Sil G/UV254). TLC plates were visualized using a 254 nm UV lamp or potassium permanganate stain followed by heating. Melting points were measured on solids as obtained after chromatography.

1. Reaction Optimization

1.1. Optimization of the C–H trifluoromethylthiolation of heteroarenes

Optimization reactions were conducted following general procedure A (see section 3) using 1-methylindole **2l** as model substrate.

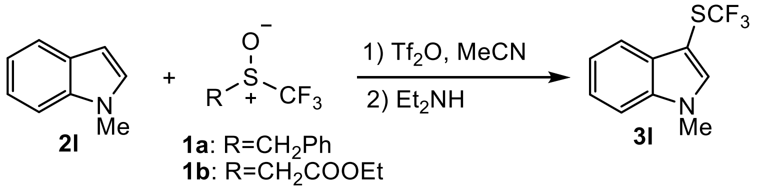

entry	**2l** (eq.)	sulfoxide (eq.)	Tf_2O (eq.)	T (°C)	t (h)	Et_2NH (eq.)	yield of **3l**[a]
1	2.0	**1a**,1.0	1.5	rt	0.5	3.5	46%
2	2.0	**1a**,1.0	1.2	rt	0.5	2.5	46%
3	1.5	**1a**,1.0	1.2	rt	0.5	2.5	45%
4	1.5	**1a**,1.0	1.2	0	1	2.5	54% (53%[b])
5	1.2	**1a**,1.0	1.2	rt	0.5	2.5	39%
6	2.0	**1a**,1.0	1.1	rt	0.5	2.5	44%
7	1.1	**1a**,1.0	1.1	rt	0.5	2.5	37%
8[c]	1.5	**1a**,1.0	1.2	0	1	2.5	n.d.
9[d]	1.0	**1b**,1.2	1.5	rt	3	3.5	17%

[a]NMR yield according to quantitative ^{19}F NMR data using trifluorotoluene as internal standard. [b]Isolated yield. [c]1.2 eq. of TFAA instead of Tf_2O was used. [d]$MeNO_2$ was used as the solvent; the reaction was pre-stirred at –25 °C for 10 min then allowed to warm up to rt for 3 h.

1.2. Optimisation of the C–H trifluoromethylthiolation of arenes

Optimisation reactions were conducted following general procedure B (see section 3) using *p*-xylene **4m** as model substrate.

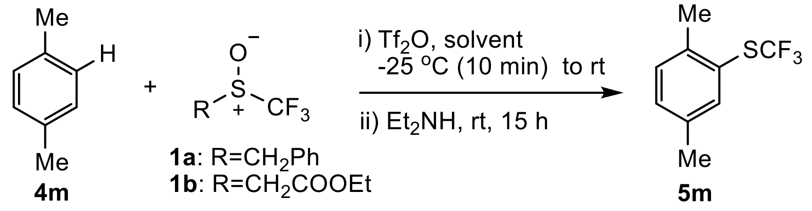

entry	**4m** (eq.)	sulfoxide (eq.)	Tf_2O (eq.)	solvent (0.2 M)	step1 T (°C), t (h)	Et_2NH (eq.)	yield of **5m**[a]
1	1.5	**1a**, 1.0	1.2	MeCN	0, 15	2.5	7%
2	1.5	**1b**, 1.0	1.2	MeCN	rt, 15	2.5	30%
3	1.5	**1b**, 1.0	1.2	CH_2Cl_2	rt, 15	2.5	17%
4	1.5	**1b**, 1.0	1.2	THF	rt, 15	2.5	n.d.
5	1.5	**1b**, 1.0	1.2	$MeNO_2$	rt, 15	2.5	53%
6[b]	1.5	**1b**, 1.0	1.2	$MeNO_2$	rt, 15	2.5	n.d.
7	1.5	**1b**, 1.0	1.2	$MeNO_2$	rt, 24	2.5	52%
8	1.5	**1b**, 1.0	1.5	$MeNO_2$	rt, 24	3.5	58%
9	1.5	**1b**, 1.0	1.5	$MeNO_2$	rt, 3	3.5	64%
10	1.0	**1b**, 1.1	1.5	$MeNO_2$	rt, 3	3.5	61%
11	1.0	**1b**, 1.2	1.5	$MeNO_2$	rt, 3	3.5	64% (60%[c])
12[d]	1.0	**1b**, 1.2	1.5	$MeNO_2$	rt, 3	3.5	51%
13[e]	1.0	**1b**, 1.2	1.5	$MeNO_2$	rt, 3	3.5	62%

[a]NMR yield according to quantitative ^{19}F NMR data using trifluoroethanol as internal standard. [b]1.2 eq. of TFAA instead of Tf_2O was used. [c]Isolated yield. [d]The molarity of the reaction was 0.4 M. [e]The molarity of the reaction was 0.1 M.

2. Sulfoxide Synthesis

2.1. Synthesis of (((trifluoromethyl)sulfinyl)methyl)benzene (1a)

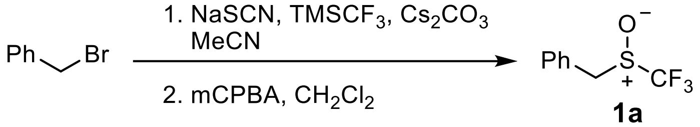

1a

Based on a previously described procedure[2], an oven-dried 100 mL round-bottom flask with a Teflon-coated stirrer bar was charged with sodium thiocyanate (1.62 g, 20 mmol). After exchanging the atmosphere three times with nitrogen, MeCN (40 mL) and benzyl bromide (3.4 g, 20 mmol) were added via syringe. The solution was heated under stirring at 60 °C for 1 h until completion of the reaction. After the reaction had cooled to room temperature, cesium carbonate (6.5 g, 20 mmol) and $TMSCF_3$ (5.7 g, 40 mmol) were added to the flask. The reaction was allowed to stir at room temperature overnight (15 h). After the reaction time, the resulting mixture was diluted with Et_2O (200 ml) and washed with water (2 × 100 mL) and brine (100 mL). The organic layer was dried over $MgSO_4$, filtered and concentrated under reduced pressure (700 mbar, 40 °C). The residue was purified by column chromatography (hexane/Et_2O 30:1) to give the corresponding sulfide as a colorless oil. The sulfide was dissolved in CH_2Cl_2 (1.0 M) followed by addition of 3-chloroperbenzoic acid (mCPBA, < 77 wt%, 1.05 eq.). The resulting mixture was allowed to stir at room temperature for 15 hours. The reaction was then quenched with saturated aqueous Na_2CO_3 at 0 °C and the aqueous phase extracted with CH_2Cl_2. The combined organic layers were dried with $MgSO_4$ and concentrated *in vacuo*. The residue was purified by column chromatography (CH_2Cl_2/Hexane = 1:1) to give the desired product as a white solid (2.42 g, 58%). ^1H NMR (500 MHz, $CDCl_3$) δ 7.45 – 7.40 (m, 3H), 7.39 – 7.34 (m, 2H), 4.26 (d, *J* = 13.1 Hz, 1H), 4.19 (d, *J* = 13.1 Hz, 1H). ^{13}C NMR (101 MHz, $CDCl_3$) *δ* 130.4, 129.34, 129.29, 127.5, 125.4 (q, *J* = 334.1 Hz), 55.7 (q, *J* = 3.1 Hz). ^{19}F NMR (471 MHz, $CDCl_3$) *δ* –72.7 (s) ppm. ν_{max}(neat)/cm^{-1} 2982, 1618, 1323, 1189, 1106, 1068, 852. HRMS (HESI) m/z calcd. for $C_8H_8OF_3S$ $[M+H]^+$: 209.0242; found: 209.0239.

2.2. Synthesis of ethyl 2-((trifluoromethyl)sulfinyl)acetate (1b)

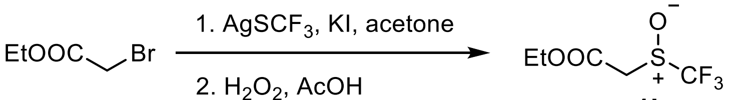

1b

Based on a previously described procedure[3], an oven-dried 500 mL round-bottom flask with a Teflon-coated stirrer bar was charged with $AgSCF_3$ (10.03 g, 48 mmol) and KI (7.64 g, 46 mmol). After exchanging the atmosphere three times with nitrogen, acetone (300 mL) was added via syringe. The mixture was stirred at room temperature until the solid completely dissolved. To the reaction was added ethyl bromoacetate (6.68 g, 40 mmol) in one portion and the reaction was allowed to stir at room temperature for 40 min. The solvent was removed under reduced pressure and the residue was treated with Et_2O (75 mL). The precipitate was removed by filtration and the filtrate was concentrated to give the corresponding sulfide as a red oil. The red oil was redissolved in acetic acid (140 mL) followed by addition of H_2O_2 (50 wt%, 2.27 mL, 40 mmol). The reaction was allowed to stir at 100 ^{o}C for 1 h. Acetic acid was removed by distillation. The crude mixture was purified by column chromatography (CH_2Cl_2/Hex = 2:1) to give the desired product as a colorless oil (4.82 g, 59%). ^{1}H NMR (400 MHz, $CDCl_3$) δ 4.32 (q, J = 7.1 Hz, 2H), 3.98 (d, J = 15.0 Hz, 1H), 3.87 (d, J = 15.0 Hz, 1H), 1.33 (t, J = 7.1 Hz, 3H) ppm. ^{13}C NMR (101 MHz, $CDCl_3$) δ 163.6, 125.2 (q, J = 334.6 Hz), 63.2, 53.8 (q, J = 3.0 Hz), 14.0 ppm. ^{19}F NMR (376 MHz, $CDCl_3$) δ –73.2 ppm. HRMS (HESI) m/z calcd. for $C_5H_8O_3F_3S$ $[M+H]^+$: 205.0141; found: 205.0140.

3. General Procedure for the C–H Trifluoromethylthiolation

General procedure A:

In an oven-dried 10 mL vial, a solution of substrate **2** or **4** (0.3 mmol, 1.5 eq.) and sulfoxide **1a** (0.2 mmol, 1.0 eq.) in MeCN (1 mL, 0.2 M) was cooled to 0 °C. Tf_2O (0.24 mmol, 1.2 eq.) was added dropwise and the reaction mixture was stirred for 1 h at 0 °C. Upon completion, Et_2NH (0.5 mmol, 2.5 eq.) was added to quench the reaction. The solvent was removed *in vacuo*, and the residue was purified by column chromatography (hexane/EtOAc or hexane/CH_2Cl_2) to afford the desired product.

General procedure B:

In an oven-dried 10 mL vial, a solution of substrate **2** or **4** (0.2 mmol, 1.0 eq.) and sulfoxide **1b** (0.24 mmol, 1.2 eq.) in $MeNO_2$ (1 mL, 0.2 M) was cooled to –25 °C. Tf_2O (0.3 mmol, 1.5 eq.) was added dropwise and the reaction mixture was stirred for 10 min at –25 °C, before being warmed to room temperature and stirred for another 3 h. Upon completion, Et_2NH (0.7 mmol, 3.5 eq.) was added and the reaction was stirred at room temperature for 15 h. After addition of H_2O (10 mL), the reaction was extracted with Et_2O (3 × 10 mL). The organic layers were combined, dried over $MgSO_4$, and concentrated under reduced pressure at 25 ^{o}C. The residue was purified by column chromatography (pentane, hexane, hexane/Et_2O, hexane/CH_2Cl_2, or CH_2Cl_2/MeOH) to afford the desired product.

4. Substrate Scope of the C–H Trifluoromethylthiolation

3-((Trifluoromethyl)thio)-1H-indole 3a[4]

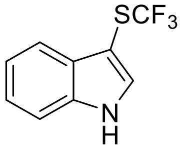

Indole (35.1 mg, 0.3 mmol), sulfoxide **1a** (41.6 mg, 0.2 mmol), Tf_2O (40 µL, 0.24 mmol) and Et_2NH (52 µL, 0.5 mmol) were reacted following general procedure A (eluent: hexane/EtOAc = 10:1) to afford the title compound as a colorless oil (30.4 mg, 70%). ^{1}H NMR (400 MHz, $CDCl_3$) δ 8.48 (s, 1H), 7.87 – 7.79 (m, 1H), 7.53 (d, J = 2.8 Hz, 1H), 7.46 – 7.38 (m, 1H), 7.36 – 7.27 (m, 2H) ppm. ^{13}C NMR (126 MHz, $CDCl_3$) δ 136.0, 132.8, 129.5 (q, J = 309.9 Hz), 129.5, 123.5, 121.7, 119.4, 111.7, 95.6 (q, J = 2.5 Hz) ppm. ^{19}F NMR (376 MHz, $CDCl_3$) δ –44.6 (s) ppm. ν_{max}(neat)/cm^{-1} 3402, 1455, 1104, 906, 729. HRMS (ASAP) m/z calcd. for $C_9H_6NF_3S$ $[M]^+$: 217.0168; found: 217.0171.

4-Bromo-3-((trifluoromethyl)thio)-1H-indole 3b[4]

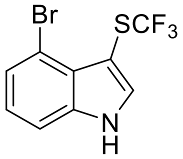

4-Bromoindole (49 µL, 0.3 mmol), sulfoxide **1a** (41.6 mg, 0.2 mmol), Tf_2O (40 µL, 0.24 mmol) and Et_2NH (52 µL, 0.5 mmol) were reacted following general procedure A (eluent: hexane/EtOAc = 6:1) to afford the title compound as a white solid (46.2 mg, 78%). m.p. 116 – 117 ºC. ^{1}H NMR (400 MHz, $CDCl_3$) δ 8.66 (s, 1H), 7.62 (d, J = 2.9 Hz, 1H), 7.43 (d, J = 7.6 Hz, 1H), 7.40 (d, J = 8.2 Hz, 1H), 7.12 (t, J = 7.9 Hz, 1H) ppm. ^{13}C NMR (126 MHz, $CDCl_3$) δ 137.1, 135.3, 129.0 (q, J = 309.6 Hz), 126.8, 126.1, 124.3, 114.4, 111.3, 96.5 (q, J = 2.9 Hz) ppm. ^{19}F NMR (376 MHz, $CDCl_3$) δ –45.5 (s) ppm. ν_{max}(neat)/cm^{-1} 3449, 3123, 1506, 1430, 1398, 1332, 1107, 909. 777, 740. HRMS (ASAP) m/z calcd. for $C_9H_5NBrF_3S$ $[M]^+$: 294.9273; found: 294.9283.

6-Bromo-3-((trifluoromethyl)thio)-1H-indole 3c[5]

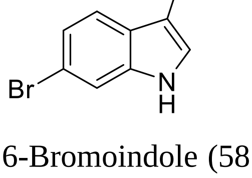

6-Bromoindole (58.8 mg, 0.3 mmol), sulfoxide **1a** (41.6 mg, 0.2 mmol), Tf_2O (40 µL, 0.24 mmol) and Et_2NH (52 µL, 0.5 mmol) were reacted following general procedure A (eluent: hexane/CH_2Cl_2 = 5:1) to afford the title compound as a colorless oil (37.9 mg, 64%). ^{1}H NMR (400 MHz, $CDCl_3$) δ 8.53 (s, 1H), 7.66 (d, J = 8.5 Hz, 1H), 7.59 (d, J = 1.7 Hz, 1H), 7.53 (d, J = 2.8 Hz, 1H), 7.38 (dd, J = 8.5, 1.7 Hz, 1H) ppm. ^{13}C NMR (126 MHz, $CDCl_3$) δ 136.8, 133.2, 129.3 (q, J = 310.0 Hz), 128.4, 125.1, 120.7, 117.1,

114.7, 96.2 (q, J = 2.5 Hz) ppm. ^{19}F NMR (376 MHz, $CDCl_3$) δ –44.5 (s) ppm. ν_{max}(neat)/cm^{-1} 3419, 3116, 1612, 1505, 1442, 1386, 1093, 890, 805. HRMS (ASAP) m/z calcd. for $C_9H_5NBrF_3S$ $[M]^+$: 294.9273; found: 294.9283.

3-((Trifluoromethyl)thio)-1H-indole-5-carbonitrile 3d[4]

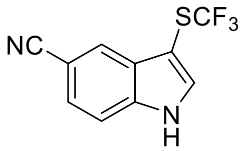

Indole-5-carbonitrile (42.6 mg, 0.3 mmol), sulfoxide **1a** (41.6 mg, 0.2 mmol), Tf_2O (40 µL, 0.24 mmol) and Et_2NH (52 µL, 0.5 mmol) were reacted following general procedure A (eluent: hexane/EtOAc = 6:1) to afford the title compound as a white solid (38.3 mg, 79%). m.p. 193 – 194 ºC. ^{1}H NMR (400 MHz, $CDCl_3$) δ 8.93 (s, 1H), 8.16 (s, 1H), 7.70 (d, J = 2.7 Hz, 1H), 7.58 – 7.48 (m, 2H) ppm. ^{13}C NMR (126 MHz, $CDCl_3$) δ 137.8, 134.9, 129.4, 129.1 (q, J = 310.0 Hz), 126.4, 125.1, 119.9, 112.9, 105.3, 97.2 (q, J = 2.8 Hz) ppm. ^{19}F NMR (376 MHz, $CDCl_3$) δ –44.2 (s) ppm. ν_{max}(neat)/cm^{-1} 3221, 2238, 1617, 1415, 1099, 809. HRMS (ASAP) m/z calcd. for $C_{10}H_6N_2F_3S$ $[M+H]^+$: 243.0198; found: 243.0201.

Methyl 3-((trifluoromethyl)thio)-1H-indole-5-carboxylate 3e[5]

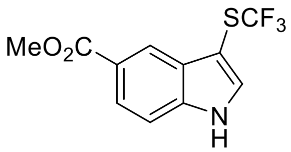

Methyl indole-5-carboxylate (52.6 mg, 0.3 mmol), sulfoxide **1a** (41.6 mg, 0.2 mmol), Tf_2O (40 µL, 0.24 mmol) and Et_2NH (52 µL, 0.5 mmol) were reacted following general procedure A (eluent: hexane/EtOAc = 6:1) to afford the title compound as a white solid (38.5 mg, 70%). m.p. 183 – 184 ºC. ^{1}H NMR (400 MHz, $CDCl_3$) δ 8.88 (s, 1H), 8.55 (s, 1H), 8.01 (dd, J = 8.6, 1.6 Hz, 1H), 7.64 (d, J = 2.8 Hz, 1H), 7.46 (dd, J = 8.7, 0.7 Hz, 1H), 3.97 (s, 3H) ppm. ^{13}C NMR (126 MHz, $CDCl_3$) δ 167.7, 138.6, 134.1, 129.22 (q, J = 309.9 Hz), 129.16, 124.9, 124.0, 122.2, 111.6, 97.4 (q, J = 2.4 Hz), 52.1 ppm. ^{19}F NMR (376 MHz, $CDCl_3$) δ –44.4 (s) ppm. ν_{max}(neat)/cm^{-1} 3264, 1689, 1618, 1424, 1291, 1110, 742. HRMS (HESI) m/z calcd. for $C_{11}H_9O_2NF_3S$ $[M+H]^+$: 276.0301; found: 276.0300.

Methyl 3-((trifluoromethyl)thio)-1H-indole-4-carboxylate 3f[6]

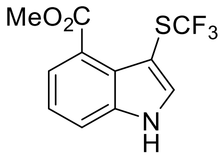

Methyl indole-4-carboxylate (52.6 mg, 0.3 mmol), sulfoxide **1a** (41.6 mg, 0.2 mmol), Tf_2O (40 µL, 0.24 mmol) and Et_2NH (52 µL, 0.5 mmol) were reacted following general procedure A (eluent: hexane/EtOAc = 6:1) to afford the title compound as a colorless oil (41.3 mg, 75%). ^{1}H NMR (400 MHz, $CDCl_3$) δ 9.27 (s, 1H), 7.61 (dd, J = 7.4, 1.0 Hz, 1H), 7.56 (d, J = 2.9 Hz, 1H), 7.52 (dd, J = 8.3, 1.0 Hz, 1H), 7.27 (dd,

J = 8.2, 7.4 Hz, 1H), 4.02 (s, 3H) ppm. ^{13}C NMR (101 MHz, $CDCl_3$) δ 169.0, 137.1, 135.6, 129.4 (q, *J* = 309.1 Hz), 125.6, 124.6, 123.4, 122.5, 115.5, 95.1 (q, *J* = 2.5 Hz), 52.1 ppm. ^{19}F NMR (376 MHz, $CDCl_3$) δ –45.1 (s) ppm. ν_{max}(neat)/cm^{-1} 3313, 2953, 1699, 1435, 1282, 1201, 1100, 907. 729. HRMS (ASAP) m/z calcd. for $C_{11}H_9O_2NF_3S$ $[M+H]^+$: 276.0301; found: 276.0306.

Methyl 3-((trifluoromethyl)thio)-1H-indole-6-carboxylate 3g[7]

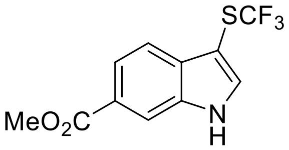

Methyl indole-6-carboxylate (52.6 mg, 0.3 mmol), sulfoxide **1a** (41.6 mg, 0.2 mmol), Tf_2O (40 µL, 0.24 mmol) and Et_2NH (52 µL, 0.5 mmol) were reacted following general procedure A (eluent: hexane/EtOAc = 6:1) to afford the title compound as a white solid (44.6 mg, 81%). m.p. 204 – 205 ºC. ^{1}H NMR (400 MHz, $CDCl_3$) δ 8.80 (s, 1H), 8.20 (dd, *J* = 1.3, 0.7 Hz, 1H), 7.97 (dd, *J* = 8.4, 1.4 Hz, 1H), 7.83 (d, *J* = 8.4 Hz, 1H), 7.70 (d, *J* = 2.9 Hz, 1H), 3.96 (s, 3H) ppm. ^{13}C NMR (126 MHz, $CDCl_3$) δ 167.5, 135.6, 135.4, 133.1, 129.2 (q, *J* = 310.0 Hz), 125.4, 122.6, 119.1, 114.1, 96.3 (q, *J* = 2.5 Hz), 52.2 ppm. ^{19}F NMR (376 MHz, $CDCl_3$) δ –44.5 (s) ppm. ν_{max}(neat)/cm^{-1} 3326, 1691, 1487, 1438, 1320, 1126, 1108, 772, 695. HRMS (ASAP) m/z calcd. for $C_{11}H_9O_2NF_3S$ $[M+H]^+$: 276.0301; found: 276.0306.

4-(4,4,5,5-Tetramethyl-1,3,2-dioxaborolan-2-yl)-3-((trifluoromethyl)thio)-1H-indole 3h

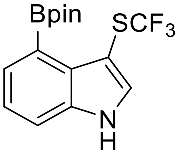

Indole-4-boronic acid pinacol ester (72.9 mg, 0.3 mmol), sulfoxide **1a** (41.6 mg, 0.2 mmol), Tf_2O (40 µL, 0.24 mmol) and Et_2NH (52 µL, 0.5 mmol) were reacted following general procedure A (eluent: hexane/EtOAc = 10:1) to afford the title compound as a white solid (26.1 mg, 38%). m.p. 186 – 187 ºC. ^{1}H NMR (400 MHz, $CDCl_3$) δ 8.62 (s, 1H), 7.57 – 7.50 (m, 2H), 7.44 (d, *J* = 8.2 Hz, 1H), 7.30 – 7.21 (m, 1H), 1.44 (s, 12H) ppm. ^{13}C NMR (101 MHz, $CDCl_3$) δ 135.7, 133.7, 131.7, 129.6 (q, *J* = 309.5 Hz), 129.0, 128.4, 122.6, 113.7, 96.4 (q, *J* = 2.4 Hz), 84.2, 24.9 ppm. ^{19}F NMR (376 MHz, $CDCl_3$) δ –45.0 (s) ppm. ν_{max}(neat)/cm^{-1} 3267, 2982, 1507, 1357, 1293, 1120, 1106, 750. HRMS (ASAP) m/z calcd. for $C_{15}H_{16}O_2NBF_3S$ $[M-H]^-$: 342.0952; found: 342.0946.

5-Methyl-3-((trifluoromethyl)thio)-1H-indole 3i[5]

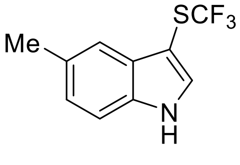

5-Methylindole (39.4 mg, 0.3 mmol), sulfoxide **1a** (41.6 mg, 0.2 mmol), Tf_2O (40 µL, 0.24 mmol) and Et_2NH (52 µL, 0.5 mmol) were reacted following general procedure A (eluent: hexane/EtOAc = 19:1) to

afford the title compound as a colorless oil (19.0 mg, 41%). ^{1}H NMR (400 MHz, $CDCl_3$) δ 8.45 (s, 1H), 7.59 (s, 1H), 7.50 (d, J = 2.8 Hz, 1H), 7.32 (d, J = 8.4 Hz, 1H), 7.12 (dd, J = 8.3, 1.7 Hz, 1H), 2.50 (s, 3H) ppm. ^{13}C NMR (126 MHz, $CDCl_3$) δ 134.3, 132.8, 131.2, 129.7, 129.4 (q, J = 310.0 Hz), 125.1, 118.9, 111.3, 94.9 (q, J = 2.5 Hz), 21.5 ppm. ^{19}F NMR (376 MHz, $CDCl_3$) δ –44.7 (s) ppm. ν_{max}(neat)/cm^{-1} 3401, 2924, 1481, 1406, 1100, 795, 750. HRMS (ASAP) m/z calcd. for $C_{10}H_8NF_3S$ $[M]^+$: 231.0324; found: 231.0334.

7-Methyl-3-((trifluoromethyl)thio)-1H-indole 3j[5]

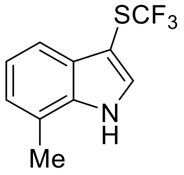

7-Methylindole (39.4 mg, 0.3 mmol), sulfoxide **1a** (41.6 mg, 0.2 mmol), Tf_2O (40 µL, 0.24 mmol) and Et_2NH (52 µL, 0.5 mmol) were reacted following general procedure A (eluent: hexane/EtOAc = 15:1) to afford the title compound as a white solid (28.2 mg, 61%). m.p. 69 – 70 ºC. ^{1}H NMR (400 MHz, $CDCl_3$) δ 8.48 (s, 1H), 7.66 (d, J = 7.9 Hz, 1H), 7.56 (d, J = 2.8 Hz, 1H), 7.20 (t, J = 7.6 Hz, 1H), 7.10 (d, J = 7.1 Hz, 1H), 2.52 (s, 3H) ppm. ^{13}C NMR (126 MHz, $CDCl_3$) δ 135.6, 132.4, 129.4 (q, J = 309.8 Hz), 129.1, 123.9, 121.8, 120.8, 117.0, 96.1 (q, J = 2.3 Hz), 16.3 ppm. ^{19}F NMR (376 MHz, $CDCl_3$) δ –44.6 (s) ppm. ν_{max}(neat)/cm^{-1} 3468, 2251, 1408, 1127, 1105, 905, 727. HRMS (ASAP) m/z calcd. for $C_{10}H_7NF_3S$ $[M\text{-}H]^-$: 230.0257; found: 230.0252.

3-Methyl-2-((trifluoromethyl)thio)-1H-indole 3k[8]

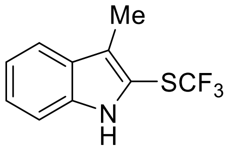

3-Methylindole (39.4 mg, 0.3 mmol), sulfoxide **1a** (41.6 mg, 0.2 mmol), Tf_2O (40 µL, 0.24 mmol) and Et_2NH (52 µL, 0.5 mmol) were reacted following general procedure A (eluent: hexane/CH_2Cl_2 = 9:1) to afford the title compound as a white solid (24.5 mg, 53%). m.p. 108 – 109 ºC. ^{1}H NMR (400 MHz, $CDCl_3$) δ 8.12 (s, 1H), 7.62 (d, J = 8.0 Hz, 1H), 7.37 (d, J = 8.3 Hz, 1H), 7.31 (ddd, J = 8.2, 6.8, 1.2 Hz, 1H), 7.17 (ddd, J = 8.0, 6.9, 1.2 Hz, 1H), 2.46 (s, 3H) ppm. ^{13}C NMR (101 MHz, $CDCl_3$) δ 137.4, 128.7 (q, J = 311.6 Hz), 127.9, 124.8, 123.7, 120.0, 120.0, 113.0 (q, J = 2.3 Hz), 111.1, 9.4 ppm. ^{19}F NMR (376 MHz, $CDCl_3$) δ –43.1 (s) ppm. ν_{max}(neat)/cm^{-1} 3392, 1448, 1120, 1105, 747. HRMS (APCI) m/z calcd. for $C_{10}H_7NF_3S$ $[M\text{-}H]^-$: 230.0257; found: 230.0250.

1-Methyl-3-((trifluoromethyl)thio)-1H-indole 3l[4]

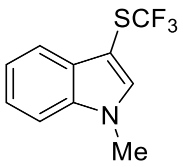

1-Methylindole (37.5 µL, 0.3 mmol), sulfoxide **1a** (41.6 mg, 0.2 mmol), Tf_2O (40 µL, 0.24 mmol) and Et_2NH (52 µL, 0.5 mmol) were reacted following general procedure A (eluent: hexane/EtOAc = 30:1) to afford the title compound as a white solid (24.5 mg, 53%). m.p. 59 – 60 ºC. ^{1}H NMR (400 MHz, $CDCl_3$) δ 7.80 (d, J = 7.7 Hz, 1H), 7.41 – 7.36 (m, 2H), 7.36 – 7.30 (m, 1H), 7.30 – 7.26 (m, 1H), 3.85 (s, 3H) ppm. ^{13}C NMR (101 MHz, $CDCl_3$) δ 137.2, 136.9, 130.2, 129.4 (q, J = 310.1 Hz), 122.9, 121.3, 119.4, 109.8, 93.1 (q, J = 2.6 Hz), 33.3 ppm. ^{19}F NMR (376 MHz, $CDCl_3$) δ –45.0 (s) ppm. ν_{max}(neat)/cm^{-1} 3291, 1484, 1120, 1102, 739. HRMS (ASAP) m/z calcd. for $C_{10}H_9NF_3S$ $[M+H]^+$: 232.0402; found: 232.0396.

5-Bromo-1-methyl-3-((trifluoromethyl)thio)-1H-indole 3m

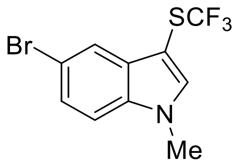

5-Bromo-1-methylindole (63.0 mg, 0.3 mmol), sulfoxide **1a** (41.6 mg, 0.2 mmol), Tf_2O (40 µL, 0.24 mmol) and Et_2NH (52 µL, 0.5 mmol) were reacted following general procedure A (eluent: hexane/EtOAc = 19:1) to afford the title compound as a white solid (52.1mg, 84%). m.p. 111 – 112 ºC. ^{1}H NMR (400 MHz, $CDCl_3$) δ 7.90 (d, J = 1.9 Hz, 1H), 7.42 – 7.37 (m, 2H), 7.23 (d, J = 8.7 Hz, 1H), 3.82 (s, 3H) ppm. ^{13}C NMR (101 MHz, $CDCl_3$) δ 137.9, 135.9, 131.8, 129.2 (q, J = 310.3 Hz), 126.0, 122.0, 115.0, 111.4, 92.8, 33.5 ppm. ^{19}F NMR (376 MHz, $CDCl_3$) δ –44.9 (s) ppm. ν_{max}(neat)/cm^{-1} 2950, 1655, 1512, 1462, 1120, 1104, 791. HRMS (ASAP) m/z calcd. for $C_{10}H_8NBrF_3S$ $[M+H]^+$: 309.9507; found: 309.9512.

1,2-Dimethyl-3-((trifluoromethyl)thio)-1H-indole 3n[8]

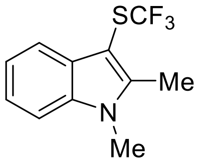

1,2-Dimethylindole (43.6 mg, 0.3 mmol), sulfoxide **1a** (41.6 mg, 0.2 mmol), Tf_2O (40 µL, 0.24 mmol) and Et_2NH (52 µL, 0.5 mmol) were reacted following general procedure A (eluent: hexane/EtOAc = 25:1) to afford the title compound as a white solid (28.9 mg, 59%). m.p. 118 – 119 ºC. ^{1}H NMR (400 MHz, $CDCl_3$) δ 7.76 – 7.70 (m, 1H), 7.35 – 7.29 (m, 1H), 7.29 – 7.20 (m, 2H), 3.74 (s, 3H), 2.58 (s, 3H) ppm. ^{13}C NMR (101 MHz, $CDCl_3$) δ 145.1, 136.8, 130.1, 129.7 (q, J = 311.0 Hz), 122.1, 121.1, 118.7, 109.2, 91.1 (q, J = 2.3 Hz), 30.4, 10.9 ppm. ^{19}F NMR (376 MHz, $CDCl_3$) δ –44.9 (s) ppm. ν_{max}(neat)/cm^{-1} 1526, 1474, 1401, 1151, 1100, 743. HRMS (ASAP) m/z calcd. for $C_{11}H_{11}NF_3S$ $[M+H]^+$: 246.0559; found:

246.0551.

3-((Trifluoromethyl)thio)benzo[b]thiophene 3o[9]

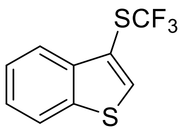

Benzothiophene (35 µL, 0.3 mmol), sulfoxide **1a** (41.6 mg, 0.2 mmol), Tf_2O (40 µL, 0.24 mmol) and Et_2NH (52 µL, 0.5 mmol) were reacted following general procedure A (eluent: hexane) to afford the title compound as a colorless oil (29.5 mg, 63%). ^{1}H NMR (400 MHz, $CDCl_3$) δ 8.04 (d, J = 8.1 Hz, 1H), 7.99 (s, 1H), 7.90 (dt, J = 8.0, 1.0 Hz, 1H), 7.52 (ddd, J = 8.2, 7.1, 1.2 Hz, 1H), 7.45 (ddd, J = 8.1, 7.0, 1.3 Hz, 1H) ppm. ^{13}C NMR (126 MHz, $CDCl_3$) δ 139.4, 139.4, 137.9, 129.0 (q, J = 310.0 Hz), 125.4, 125.3, 122.9, 122.8, 115.2 (q, J = 2.2 Hz) ppm. ^{19}F NMR (376 MHz, $CDCl_3$) δ –42.6 (s) ppm. ν_{max}(neat)/cm^{-1} 2360, 1421, 1102, 905, 668. HRMS (HESI) m/z calcd. for $C_9H_5F_3S_2$ $[M]^+$: 233.9779; found: 233.9781.

5-((Trifluoromethyl)thio)-2,2'-bithiophene 3p

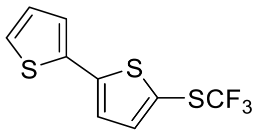

2,2’-Bithiophene (49.9 mg, 0.3 mmol), sulfoxide **1a** (41.6 mg, 0.2 mmol), Tf_2O (40 µL, 0.24 mmol) and Et_2NH (52 µL, 0.5 mmol) were reacted following general procedure A (eluent: hexane) to afford the title compound as a colorless oil (37.8 mg, 71%). ^{1}H NMR (400 MHz, $CDCl_3$) δ 7.32 (d, J = 3.8 Hz, 1H), 7.29 (dd, J = 5.1, 1.1 Hz, 1H), 7.23 (dd, J = 3.6, 1.1 Hz, 1H), 7.14 (d, J = 3.8 Hz, 1H), 7.05 (dd, J = 5.1, 3.6 Hz, 1H) ppm. ^{13}C NMR (101 MHz, $CDCl_3$) δ 145.9, 140.3, 136.0, 128.3 (q, J = 311.0 Hz), 128.1, 125.9, 125.0, 124.2, 118.9 (q, J = 2.5 Hz) ppm. ^{19}F NMR (376 MHz, $CDCl_3$) δ –45.2 (s) ppm. ν_{max}(neat)/cm^{-1} 2364, 1440, 1135, 1107, 905, 729, 697. HRMS (APPI) m/z calcd. for $C_9H_5F_3S_3$ $[M]^+$: 265.9500; found: 265.9488.

2-((Trifluoromethyl)thio)thieno[3,2-*b*]thiophene 3q

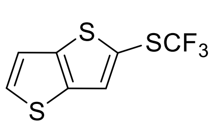

Thieno[3,2-*b*]thiophene (42.1 mg, 0.3 mmol), sulfoxide **1a** (41.6 mg, 0.2 mmol), Tf_2O (40 µL, 0.24 mmol) and Et_2NH (52 µL, 0.5 mmol) were reacted following general procedure A (eluent: hexane) to afford the title compound as a colorless oil (31.5 mg, 66%). ^{1}H NMR (400 MHz, $CDCl_3$) δ 7.63 (s, 1H), 7.57 (d, J = 5.3 Hz, 1H), 7.27 (d, J = 5.3 Hz, 1H) ppm. ^{13}C NMR (101 MHz, $CDCl_3$) δ 145.3, 138.5, 131.8, 130.1, 128.4 (q, J = 310.9 Hz), 122.0 (q, J = 2.5 Hz), 119.5 ppm. ^{19}F NMR (376 MHz, $CDCl_3$) δ –45.0 ppm. ν_{max}(neat)/cm^{-1} 3090, 1442, 1346, 1132, 1099, 709. HRMS (APCI) m/z calcd. for $C_7H_2F_3S_3[M-H]^-$: 238.9276; found: 238.9272.

3-((Trifluoromethyl)thio)benzofuran 3r[9]

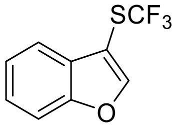

Benzofuran (33 µL, 0.3 mmol), sulfoxide **1a** (41.6 mg, 0.2 mmol), Tf_2O (40 µL, 0.24 mmol) and Et_2NH (52 µL, 0.5 mmol) were reacted following general procedure A (eluent: hexane) to afford the title compound as a colorless oil (27.1 mg, 62%). ^1H NMR (400 MHz, $CDCl_3$) δ 7.94 (s, 1H), 7.78 – 7.72 (m, 1H), 7.60 – 7.52 (m, 1H), 7.45 – 7.35 (m, 2H) ppm. ^{13}C NMR (101 MHz, $CDCl_3$) δ 155.5, 151.6, 128.9 (q, J = 309.9 Hz), 128.0, 125.6, 124.1, 120.0, 112.0, 103.2 (q, J = 2.5 Hz) ppm. ^{19}F NMR (376 MHz, $CDCl_3$) δ –43.1 (s) ppm. ν_{max}(neat)/cm^{-1} 2362, 1450, 1164, 1112, 904, 727. HRMS (HESI) m/z calcd. for $C_9H_5OF_3S$ $[M]^+$: 218.0008; found: 218.0010.

1-Phenyl-2/3-((trifluoromethyl)thio)-1H-pyrrole 3s

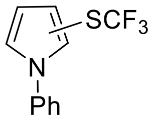

1-Phenylpyrrole (43.0 mg, 0.3 mmol), sulfoxide **1a** (41.6 mg, 0.2 mmol), Tf_2O (40 µL, 0.24 mmol) and Et_2NH (52 µL, 0.5 mmol) were reacted following general procedure A. After separation by column chromatography (eluent: hexane), two regioisomers of the title compound were obtained, namely 2-**3s**[7] and 3-**3s**, in a ratio of 1:1, The total yield was 45.2 mg (93%). Both 2-**3s** and 3-**3s** are colorless oils.

2-**3s** : ^1H NMR (400 MHz, $CDCl_3$) δ 7.51 – 7.40 (m, 3H), 7.35 – 7.29 (m, 2H), 7.13 (dd, J = 2.9, 1.8 Hz, 1H), 6.85 (dd, J = 3.8, 1.8 Hz, 1H), 6.38 (dd, J = 3.8, 2.9 Hz, 1H) ppm; ^{13}C NMR (101 MHz, $CDCl_3$) δ 138.9, 128.9, 128.7, 128.1 (q, J = 311.5 Hz), 128.1, 127.0, 123.9, 110.6 (q, J = 2.4 Hz), 110.0 ppm; ^{19}F NMR (376 MHz, $CDCl_3$) δ –45.3 ppm; ν_{max}(neat)/cm^{-1} 2253, 1500, 1321, 1146, 1108, 904, 725; HRMS (APCI) m/z calcd. for $C_{11}H_7NF_3S$ $[M\text{-}H]^-$: 242.0257; found: 242.0253.

3-**3s**: ^1H NMR (400 MHz, $CDCl_3$) δ 7.52 – 7.42 (m, 2H), 7.42 – 7.36 (m, 2H), 7.36 – 7.29 (m, 2H), 7.10 (t, J = 2.6 Hz, 1H), 6.52 (dd, J = 3.0, 1.7 Hz, 1H) ppm; ^{13}C NMR (101 MHz, $CDCl_3$) δ 139.8, 129.8, 129.3 (q, J = 308.5 Hz), 126.8, 126.8, 121.0, 120.9, 116.9, 103.5 (q, J = 2.6 Hz) ppm; ^{19}F NMR (376 MHz, $CDCl_3$) δ –45.2 ppm; ν_{max}(neat)/cm^{-1} 2253, 1601, 1507, 1330, 1152, 1108, 905, 728; HRMS (ASAP) m/z calcd. for $C_{11}H_9NF_3S$ $[M+H]^+$: 244.0402; found: 244.0410.

Methyl 1-methyl-4-((trifluoromethyl)thio)-1H-pyrrole-2-carboxylate 3t

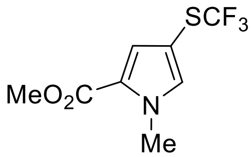

Methyl 1-methylpyrrole-2-carboxylate (41.7 mg, 0.3 mmol), sulfoxide **1a** (41.6 mg, 0.2 mmol), Tf_2O (40 µL, 0.24 mmol) and Et_2NH (52 µL, 0.5 mmol) were reacted following general procedure A (eluent:

hexane/CH_2Cl_2 = 2:1) to afford the title compound as a colorless oil (34.4 mg, 72%). ^{1}H NMR (400 MHz, $CDCl_3$) δ 7.10 (d, J = 1.9 Hz, 1H), 7.04 (d, J = 1.9 Hz, 1H), 3.95 (s, 3H), 3.83 (s, 3H) ppm. ^{13}C NMR (101 MHz, $CDCl_3$) δ 160.9, 135.5, 129.0 (q, J = 308.5 Hz), 124.1, 124.0, 100.8 (q, J = 2.6 Hz), 51.4, 37.3 ppm. ^{19}F NMR (376 MHz, $CDCl_3$) δ –45.2 (s) ppm. ν_{max}(neat)/cm^{-1} 2956, 1710, 1530, 1437, 1384, 1250, 1102, 754. HRMS (ASAP) m/z calcd. for $C_8H_9O_2NF_3S$ $[M+H]^+$: 240.0301; found: 240.0307.

(4-Methoxyphenyl)(trifluoromethyl)sulfide 5a[10]

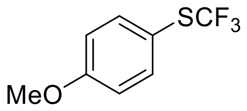

Anisole (22 µL, 0.2 mmol), sulfoxide **1b** (49 mg, 0.24 mmol), Tf_2O (50 µL, 0.3 mmol) and Et_2NH (72 µL, 0.7 mmol) were reacted following general procedure B (eluent: pentane) to afford the title compound as a colorless oil (26.2 mg, 63%). ^{1}H NMR (400 MHz, $CDCl_3$) δ 7.58 (d, J = 8.7 Hz, 2H), 6.93 (d, J = 8.8 Hz, 2H), 3.84 (s, 3H) ppm. ^{13}C NMR (101 MHz, $CDCl_3$) δ 161.8, 138.3, 129.6 (q, J = 308.1 Hz), 115.0, 114.8 (q, J = 2.3 Hz), 55.4 ppm. ^{19}F NMR (376 MHz, $CDCl_3$) δ –44.0 (s) ppm. ν_{max}(neat)/cm^{-1} 2950, 2397, 1592, 1494, 1253, 1113, 829. HRMS (ASAP) m/z calcd. for $C_8H_7OF_3S$ $[M]^+$: 208.0164; found: 208.0165.

***p*-Tolyl(trifluoromethyl)sulfide 5b**[11]

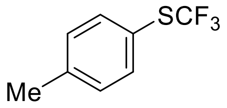

Toluene (21 µL, 0.2 mmol), sulfoxide **1b** (49 mg, 0.24 mmol), Tf_2O (50 µL, 0.3 mmol) and Et_2NH (72 µL, 0.7 mmol) were reacted following general procedure B (eluent: pentane) to afford the title compound as a colorless oil (17.7 mg, 46%). The product contains two regioisomers, *para*-**5b** and *ortho*-**5b**, in a ratio of 13:1. Major product (*para*-**5b**): ^{1}H NMR (500 MHz, $CDCl_3$) δ 7.54 (d, J = 8.0 Hz, 2H), 7.23 (d, J = 7.8 Hz, 2H), 2.39 (s, 3H) ppm. ^{13}C NMR (101 MHz, $CDCl_3$) δ 141.4, 136.4, 130.2, 129.7 (q, J = 308.1 Hz), 120.9 (q, J = 1.9 Hz), 21.3 ppm. ^{19}F NMR (376 MHz, $CDCl_3$) δ –43.2 (s) ppm. ν_{max}(neat)/cm^{-1} 1116, 903, 725. HRMS (APPI) m/z calcd. for $C_8H_7F_3S$ $[M]^+$: 192.0215; found: 192.0210.

(4-Isopropylphenyl)(trifluoromethyl)sulfide 5c

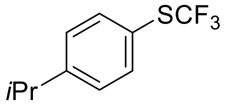

Isopropylbenzene (28 µL 0.2 mmol), sulfoxide **1b** (49 mg, 0.24 mmol), Tf_2O (50 µL, 0.3 mmol) and Et_2NH (72 µL, 0.7 mmol) were reacted following general procedure B (eluent: pentane) to afford the title compound as a colorless oil (20.3 mg, 46%). ^{1}H NMR (500 MHz, $CDCl_3$) δ 7.57 (d, J = 7.9 Hz, 2H), 7.28 (d, J = 8.1 Hz, 2H), 2.94 (septet, J = 6.9 Hz, 1H), 1.26 (d, J = 6.9 Hz, 6H) ppm. ^{13}C NMR (126 MHz, $CDCl_3$) δ 152.1, 136.5, 129.7 (q, J = 308.0 Hz), 127.7, 121.1 (q, J = 2.2 Hz), 34.0, 23.7 ppm. ^{19}F NMR

(376 MHz, $CDCl_3$) δ –43.1 (s) ppm. ν_{max}(neat)/cm^{-1} 2925, 1120, 903, 725, 650. HRMS (APPI) m/z calcd. for $C_{10}H_{11}F_3S$ $[M]^+$: 220.0528; found: 220.0525.

(4-(*tert*-Butyl)phenyl)(trifluoromethyl)sulfide 5d[11]

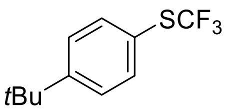

tert-Butylbenzene (31 µL, 0.2 mmol), sulfoxide **1b** (49 mg, 0.24 mmol), Tf_2O (50 µL, 0.3 mmol) and Et_2NH (72 µL, 0.7 mmol) were reacted following general procedure B (eluent: pentane) to afford the title compound as a colorless oil (23.4 mg, 50%). ^{1}H NMR (400 MHz, $CDCl_3$) δ 7.58 (d, J = 8.4 Hz, 2H), 7.43 (d, J = 8.5 Hz, 2H), 1.34 (s, 9H) ppm. ^{13}C NMR (101 MHz, $CDCl_3$) δ 154.4, 136.1, 129.7 (q, J = 307.9 Hz), 126.6, 120.9 (q, J = 2.3 Hz), 34.9, 31.1 ppm. ^{19}F NMR (376 MHz, $CDCl_3$) δ –43.0 (s) ppm. ν_{max}(neat)/cm^{-1} 2965, 1489, 1124, 1101, 831. HRMS (APPI) m/z calcd. for $C_{11}H_{13}F_3S$ $[M]^+$: 234.0685; found: 234.0681.

4-((trifluoromethyl)thio)phenol 5e[1]

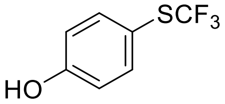

Phenol (28.2 mg, 0.3 mmol), sulfoxide **1a** (41.6 mg, 0.2 mmol), Tf_2O (40 µL, 0.24 mmol) and Et_2NH (52 µL, 0.5 mmol) were reacted following general procedure A (eluent: hexane/Et_2O = 6:1) to afford the title compound as a colorless oil (14.8 mg, 38%). ^{1}H NMR (400 MHz, $CDCl_3$) δ 7.54 (d, J = 8.7 Hz, 2H), 6.87 (d, J = 8.7 Hz, 2H), 5.15 (s, 1H) ppm. ^{13}C NMR (126 MHz, $CDCl_3$) δ 158.0, 138.6, 129.6 (q, J = 308.1 Hz), 116.5, 115.2 (q, J = 2.2 Hz) ppm. ^{19}F NMR (376 MHz, $CDCl_3$) δ –43.9 (s) ppm. ν_{max}(neat)/cm^{-1} 3370, 1586, 1115, 904, 726. HRMS (HESI) m/z calcd. for $C_7H_4OF_3S$ $[M-H]^-$: 192.9940; found: 192.9936.

(3,4-Dimethoxyphenyl)(trifluoromethyl)sulfide 5g[10]

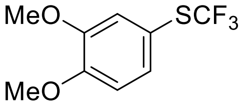

1,2-Dimethoxybenzene (25.5 µL, 0.2 mmol), sulfoxide **1b** (49 mg, 0.24 mmol), Tf_2O (50 µL, 0.3 mmol) and Et_2NH (72 µL, 0.7 mmol) were reacted following general procedure B (eluent: pentane/Et_2O = 25:1) to afford the title compound as a colorless oil (33.4 mg, 70%). ^{1}H NMR (400 MHz, $CDCl_3$) δ 7.25 (dd, J = 8.3, 2.1 Hz, 1H), 7.11 (d, J = 2.1 Hz, 1H), 6.88 (d, J = 8.4 Hz, 1H), 3.91 (s, 3H), 3.90 (s, 3H) ppm. ^{13}C NMR (101 MHz, $CDCl_3$) δ 151.5, 149.2, 130.2, 129.6 (q, J = 308.3 Hz), 118.9, 114.9 (q, J = 2.0 Hz), 111.4, 56.1, 56.0 ppm. ^{19}F NMR (376 MHz, $CDCl_3$) δ –43.7 (s) ppm. ν_{max}(neat)/cm^{-1} 2938, 2255, 1585, 1505, 1256, 1107, 1023, 730. HRMS (ASAP) m/z calcd. for $C_9H_9O_2F_3S$ $[M]^+$: 238.0270; found: 238.0274.

Methyl 2-methoxy-5-((trifluoromethyl)thio)benzoate 5h

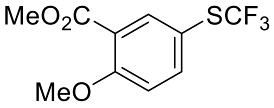

Methyl 2-methoxybenzoate (43 µL, 0.3 mmol), sulfoxide **1b** (40.8 mg, 0.2 mmol), Tf_2O (40 µL, 0.24 mmol) and Et_2NH (52 µL, 0.5 mmol) were reacted following general procedure B (eluent: CH_2Cl_2/MeOH = 50:1) to afford the title compound as a colorless oil (33.5 mg, 63%). ^1H NMR (400 MHz, $CDCl_3$) δ 8.08 (d, J = 2.4 Hz, 1H), 7.74 (dd, J = 8.7, 2.4 Hz, 1H), 7.02 (d, J = 8.8 Hz, 1H), 3.94 (s, 3H), 3.91 (s, 3H) ppm. ^{13}C NMR (101 MHz, $CDCl_3$) δ 165.3, 161.2, 141.8, 140.1, 129.4 (q, J = 308.4 Hz), 121.2, 114.8 (q, J = 2.4 Hz), 113.0, 56.3, 52.3 ppm. ^{19}F NMR (376 MHz, $CDCl_3$) δ –43.6 (s) ppm. ν_{max}(neat)/cm^{-1} 1726, 1489, 1245, 1121, 905, 727. HRMS (ASAP) m/z calcd. for $C_{10}H_{10}O_3F_3S$ $[M+H]^+$: 267.0297; found: 267.0297.

(3-Bromo-4-methoxyphenyl)(trifluoromethyl)sulfide 5i

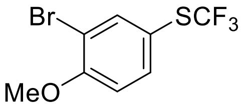

2-Bromoanisole (25 µL, 0.2 mmol), sulfoxide **1b** (49 mg, 0.24 mmol), Tf_2O (50 µL, 0.3 mmol) and Et_2NH (72 µL, 0.7 mmol) were reacted following general procedure B (eluent: hexane) to afford the title compound as a colorless oil (45.9 mg, 80%). ^1H NMR (400 MHz, $CDCl_3$) δ 7.84 (d, J = 2.2 Hz, 1H), 7.58 (dd, J = 8.6, 2.2 Hz, 1H), 6.92 (d, J = 8.6 Hz, 1H), 3.94 (s, 3H) ppm. ^{13}C NMR (126 MHz, $CDCl_3$) δ 158.2, 141.0, 137.3, 129.4 (q, J = 308.5 Hz), 116.1 (q, J = 2.4 Hz), 112.22, 112.20, 56.4 ppm. ^{19}F NMR (376 MHz, $CDCl_3$) δ –43.6 (s) ppm. ν_{max}(neat)/cm^{-1} 1583, 1268, 1114, 906, 730. HRMS (HESI) m/z calcd. for $C_8H_6OBrF_3S$ $[M]^+$: 285.9269; found: 285.9272.

5-((Trifluoromethyl)thio)benzo[*d*][1,3]dioxole 5j[10]

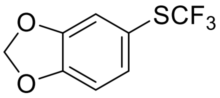

1,3-Benzodioxole (23 µL 0.2 mmol), sulfoxide **1b** (49 mg, 0.24 mmol), Tf_2O (50 µL, 0.3 mmol) and Et_2NH (72 µL, 0.7 mmol) were reacted following general procedure B (eluent: hexane) to afford the title compound as a colorless oil (30.2 mg, 68%). ^1H NMR (400 MHz, $CDCl_3$) δ 7.17 (dd, J = 8.1, 1.8 Hz, 1H), 7.09 (d, J = 1.8 Hz, 1H), 6.84 (d, J = 8.1 Hz, 1H), 6.04 (s, 2H) ppm. ^{13}C NMR (101 MHz, $CDCl_3$) δ 150.3, 148.3, 131.6, 129.5 (q, J = 308.2 Hz), 116.2, 116.0 (q, J = 2.3 Hz), 109.0, 101.9 ppm. ^{19}F NMR (376 MHz, $CDCl_3$) δ –43.9 (s) ppm. ν_{max}(neat)/cm^{-1} 2900, 1471, 1239, 1115, 1097, 1038, 907, 732. HRMS (APPI) m/z calcd. for $C_8H_5O_2F_3S$ $[M]^+$: 221.9957; found: 221.9950.

(3,5-Dimethoxyphenyl)(trifluoromethyl)sulfide 5k[1]

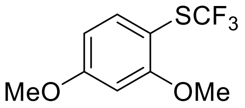

1,3-Dimethoxybenzene (26 µL, 0.2 mmol), sulfoxide **1b** (49 mg, 0.24 mmol), Tf_2O (50 µL, 0.3 mmol) and Et_2NH (72 µL, 0.7 mmol) were reacted following general procedure B (eluent: pentane) to afford the title compound as a colorless oil (34.8 mg, 73%). The product contains two regioisomers, 4-**5k** and 2-**5k**, in a ratio of 3.9:1. Major product (4-**5k**): ^{1}H NMR (400 MHz, $CDCl_3$) δ 7.53 (d, J = 8.3 Hz, 1H), 6.56 – 6.46 (m, 2H), 3.88 (s, 3H), 3.84 (s, 3H) ppm. ^{13}C NMR (101 MHz, $CDCl_3$) δ 163.9, 162.1, 140.2, 129.5 (q, J = 309.3 Hz), 105.6, 103.2 (q, J = 2.1 Hz), 99.3, 56.0, 55.5 ppm. ^{19}F NMR (376 MHz, $CDCl_3$) δ –43.7 (s) ppm. ν_{max}(neat)/cm^{-1} 1601, 1107, 904, 726. HRMS (HESI) m/z calcd. for $C_9H_{10}O_2F_3S$ $[M+H]^+$: 239.0348; found: 239.0351. Minor product (2-**5k**) : ^{1}H NMR (400 MHz, $CDCl_3$) δ 7.42 (t, J = 8.5 Hz, 1H), 6.61 (d, J = 8.4 Hz, 2H), 3.90 (s, 6H). ^{19}F NMR (376 MHz, $CDCl_3$) δ –43.7 ppm.

Methyl 4-methoxy-3-((trifluoromethyl)thio)benzoate 5l

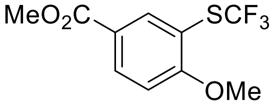

Methyl 4-methoxybenzoate (36.0 mg, 0.2 mmol), sulfoxide **1b** (49 mg, 0.24 mmol), Tf_2O (50 µL, 0.3 mmol) and Et_2NH (72 µL, 0.7 mmol) were reacted following general procedure B (eluent: hexane/CH_2Cl_2 = 1:1) to afford the title compound as a colorless oil (35.9 mg, 64%). ^{1}H NMR (400 MHz, $CDCl_3$) δ 8.31 (d, J = 2.2 Hz, 1H), 8.16 (dd, J = 8.7, 2.2 Hz, 1H), 7.01 (d, J = 8.7 Hz, 1H), 3.97 (s, 3H), 3.91 (s, 3H) ppm. ^{13}C NMR (101 MHz, $CDCl_3$) δ 165.7, 163.9, 140.1, 134.8, 129.3 (q, J = 309.1 Hz), 123.3, 112.8 (q, J = 1.9 Hz), 111.2, 56.4, 52.2 ppm. ^{19}F NMR (376 MHz, $CDCl_3$) δ –42.2 (s) ppm. ν_{max}(neat)/cm^{-1} 2254, 1716, 1598, 1436, 1272, 1108, 905, 726. HRMS (HESI) m/z calcd. for $C_{10}H_{10}O_3F_3S$ $[M+H]^+$: 267.0297; found: 267.0299.

(2,5-Dimethylphenyl)(trifluoromethyl)sulfide 5m

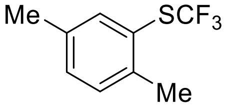

p-Xylene (25 µL, 0.2 mmol), sulfoxide **1b** (49 mg, 0.24 mmol), Tf_2O (50 µL, 0.3 mmol) and Et_2NH (72 µL, 0.7 mmol) were reacted following general procedure B (eluent: pentane) to afford the title compound as a colorless oil (24.7 mg, 60%). ^{1}H NMR (400 MHz, $CDCl_3$) δ 7.47 (s, 1H), 7.24 – 7.13 (m, 2H), 2.49 (s, 3H), 2.33 (s, 3H) ppm. ^{13}C NMR (126 MHz, $CDCl_3$) δ 140.8, 138.6, 136.6, 132.2, 130.8, 129.9 (q, J = 308.5 Hz), 123.3 (q, J = 2.0 Hz), 20.6, 20.6 ppm. ^{19}F NMR (376 MHz, $CDCl_3$) δ –42.4 (s) ppm. ν_{max}(neat)/cm^{-1} 1121, 903, 725. HRMS (APPI) m/z calcd. for $C_9H_9F_3S$ $[M]^+$: 206.0372; found: 206.0375.

5-Bromo-6-((trifluoromethyl)thio)benzo[*d*][1,3]dioxole 5n

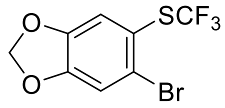

5-Bromo-1,3-benzodioxole (24 µL, 0.2 mmol), sulfoxide **1b** (49 mg, 0.24 mmol), Tf_2O (50 µL, 0.3 mmol) and Et_2NH (72 µL, 0.7 mmol) were reacted following general procedure B (eluent: hexane) to afford the title compound as a yellowish solid (26.5 mg, 44%). m.p. 44 – 46 ºC. ^1H NMR (500 MHz, $CDCl_3$) δ 7.20 (s, 1H), 7.16 (s, 1H), 6.06 (s, 2H) ppm. ^{13}C NMR (126 MHz, $CDCl_3$) δ 151.1, 147.8, 129.3 (q, J = 309.9 Hz), 123.8, 117.3, 117.2 (q, J = 2.2 Hz), 113.7, 102.7 ppm. ^{19}F NMR (376 MHz, $CDCl_3$) δ –42.9 (s) ppm. ν_{max}(neat)/cm^{-1} 2881, 1470, 1235, 1098, 1079, 1034, 800. HRMS (APCI): m/z calcd. for $C_8H_4O_2BrF_3S$ $[M]^+$: 299.9062; found: 299.9061.

(Trifluoromethyl)(2,4,6-trimethoxyphenyl)sulfide 5o[12]

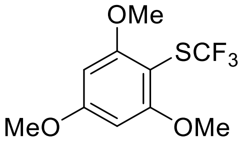

1,3,5-Trimethoxybenzene (33.6 mg, 0.2 mmol), sulfoxide **1b** (49 mg, 0.24 mmol), Tf_2O (50 µL, 0.3 mmol) and Et_2NH (72 µL, 0.7 mmol) were reacted following general procedure B (eluent: pentane) to afford the title compound as a colorless oil (52.0 mg, 97%). ^1H NMR (500 MHz, $CDCl_3$) δ 6.15 (s, 2H), 3.87 (s, 6H), 3.85 (s, 3H) ppm. ^{13}C NMR (126 MHz, $CDCl_3$) δ 164.5, 163.5, 129.5 (q, J = 310.5 Hz), 91.7, 91.0, 56.2, 55.5 ppm. ^{19}F NMR (471 MHz, $CDCl_3$) δ –43.5 (s) ppm. ν_{max}(neat)/cm^{-1} 2937, 1543, 1107, 904, 728. HRMS (APCI): m/z calcd. for $C_{10}H_{11}F_3O_3S$ $[M]^+$: 268.0376; found: 268.0376.

Naphthalen-1-yl(trifluoromethyl)sulfide 5p[13]

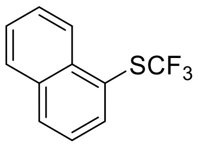

Naphthalene (25.6 mg, 0.2 mmol), sulfoxide **1b** (49 mg, 0.24 mmol), Tf_2O (50 µL, 0.3 mmol) and Et_2NH (72 µL, 0.7 mmol) were reacted following general procedure B (eluent: pentane) to afford the title compound as a colorless oil (11.9 mg, 26%). The product contains two regioisomers, 1-**5p** and 2-**5p**, in a ratio of 6.3:1. Major product (1-**5p**): ^1H NMR (400 MHz, $CDCl_3$) δ 8.56 (d, J = 8.5 Hz, 1H), 8.02 (d, J = 8.3 Hz, 1H), 7.98 (d, J = 7.1 Hz, 1H), 7.91 (d, J = 8.3 Hz, 1H), 7.67 (t, J = 7.5 Hz, 1H), 7.58 (t, J = 7.4 Hz, 1H), 7.52 (t, J = 7.7 Hz, 1H) ppm. ^{13}C NMR (126 MHz, $CDCl_3$) δ 137.8, 135.4, 134.3, 132.3, 129.7 (q, J = 309.4 Hz), 128.5, 127.7, 126.7, 125.8, 125.5, 121.6 (q, J = 2.0 Hz) ppm. ^{19}F NMR (376 MHz, $CDCl_3$) δ –42.2 (s) ppm. ν_{max}(neat)/cm^{-1} 1101, 905, 728. HRMS (APCI) m/z calcd. for $C_{11}H_6F_3S$ $[M-H]^-$: 227.0148; found: 227.0144.

(4-Methoxynaphthalen-1-yl)(trifluoromethyl)sulfide 5q[14]

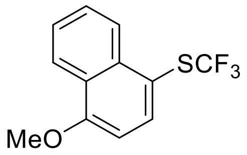

1-Methoxynaphthalene (44 µL, 0.3 mmol), sulfoxide **1a** (41.6 mg, 0.2 mmol), Tf_2O (40 µL, 0.24 mmol) and Et_2NH (52 µL, 0.5 mmol) were reacted following general procedure A (eluent: hexane) to afford the title compound as a colorless oil (37.7 mg, 73%). ^{1}H NMR (400 MHz, $CDCl_3$) δ 8.48 (d, J = 8.5 Hz, 1H), 8.33 (d, J = 8.3 Hz, 1H), 7.90 (d, J = 8.0 Hz, 1H), 7.67 (ddd, J = 8.4, 6.8, 1.4 Hz, 1H), 7.56 (ddd, J = 8.2, 6.9, 1.2 Hz, 1H), 6.84 (d, J = 8.1 Hz, 1H), 4.05 (s, 3H) ppm. ^{13}C NMR (101 MHz, $CDCl_3$) δ 158.7, 138.9, 136.2, 129.7 (q, J = 309.6 Hz), 128.0, 126.4, 125.9, 125.7, 122.5, 112.2 (q, J = 2.0 Hz), 103.9, 55.7 ppm. ^{19}F NMR (376 MHz, $CDCl_3$) δ –43.3 (s) ppm. ν_{max}(neat)/cm^{-1} 2255, 1620, 1460, 1268, 1106, 904, 728. HRMS (HESI) m/z calcd. for $C_{12}H_{10}OF_3S$ [M+H]$^+$: 259.0399; found: 259.0396.

(2,2'-Dimethoxy-[1,1'-binaphthalene]-6,6'-diyl)bis((trifluoromethyl)sulfide) 5r[13]

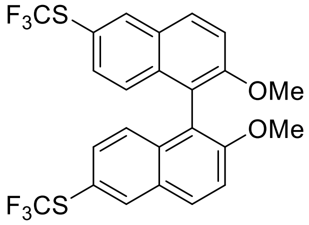

2,2'-Dimethoxy-1,1'-binaphthyl (62.9 mg, 0.2 mmol), sulfoxide **1b** (98 mg, 0.48 mmol), Tf_2O (101 µL, 0.6 mmol) and Et_2NH (134 µL, 1.3 mmol) were reacted following general procedure B (eluent: hexane/EtOAc = 20:1) to afford the title compound as a white solid (28.8 mg, 28%). m.p. 115 – 117 ºC. ^{1}H NMR (400 MHz, $CDCl_3$) δ 8.23 (s, 2H), 8.03 (d, J = 9.1 Hz, 2H), 7.52 (d, J = 9.1 Hz, 2H), 7.39 (d, J = 8.9 Hz, 2H), 7.09 (d, J = 8.9 Hz, 2H), 3.80 (s, 6H) ppm. ^{13}C NMR (126 MHz, $CDCl_3$) δ 156.5, 137.4, 134.5, 132.5, 130.2, 129.7 (q, J = 308.5 Hz), 129.0, 126.3, 118.7 (q, J = 2.3 Hz), 118.6, 114.6, 56.6 ppm. ^{19}F NMR (376 MHz, $CDCl_3$) δ –42.7 (s) ppm. ν_{max}(neat)/cm^{-1} 2361, 1270, 1112, 568. HRMS (APCI): m/z calcd. for $C_{24}H_{16}O_2F_6S_2$ [M]$^+$: 514.0490; found: 514.0487.

(4,8-Dihydropyren-1-yl)(trifluoromethyl)sulfide 5s

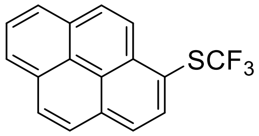

Pyrene (60.7 mg, 0.3 mmol), sulfoxide **1a** (41.6 mg, 0.2 mmol), Tf_2O (40 µL, 0.24 mmol) and Et_2NH (52 µL, 0.5 mmol) were reacted following general procedure A (eluent: hexane/CH_2Cl_2 = 10:1) to afford the title compound as a white solid (32.0 mg, 53%). m.p. 141 - 142 ºC. ^{1}H NMR (400 MHz, $CDCl_3$) δ 8.81 (d, J = 9.3 Hz, 1H), 8.38 (d, J = 8.0 Hz, 1H), 8.33 – 8.24 (m, 3H), 8.19 (dd, J = 8.5, 3.2 Hz, 2H), 8.08 (dd, J = 8.4, 6.4 Hz, 2H) ppm. ^{13}C NMR (126 MHz, $CDCl_3$) δ 135.9, 135.0, 133.7, 131.0, 130.6, 129.8 (q, J =

309.8 Hz), 129.6, 129.4, 127.0, 126.6, 126.3, 126.3, 125.3, 124.9, 124.8, 124.0, 117.8 (q, J = 2.0 Hz) ppm. ^{19}F NMR (376 MHz, $CDCl_3$) δ –42.3 (s) ppm. ν_{max}(neat)/cm^{-1} 3039, 1583, 1102, 845. HRMS (HESI) m/z calcd. for $C_{17}H_{10}F_3S$ [M+H]$^+$: 303.0450; found: 303.0442.

(4*bS*,8*aR*,9*S*)-3-Methoxy-11-methyl-2-((trifluoromethyl)thio)-6,7,8,8*a*,9,10-hexahydro-5H-9,4b-(epiminoethano)phenanthrene 5t

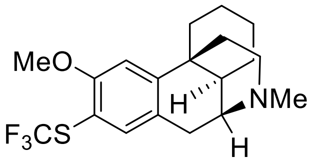

Dextromethorphan (54.3 mg, 0.2 mmol), sulfoxide **1b** (61.2 mg, 0.3 mmol), Tf_2O (84 µL, 0.5 mmol) with additional TfOH (18 µL ,0.2 mmol), and Et_2NH (134 µL, 1.3 mmol) were were reacted following general procedure B (eluent: CH_2Cl_2/MeOH = 19:1) to afford the title compound as a colorless oil (63.9 mg, 86%). ^{1}H NMR (400 MHz, $CDCl_3$) δ 7.34 (s, 1H), 6.83 (s, 1H), 3.86 (s, 3H), 2.98 (d, J = 18.2 Hz, 1H), 2.82 (dd, J = 5.8, 3.2 Hz, 1H), 2.58 (dd, J = 18.2, 5.8 Hz, 1H), 2.45 (ddd, J = 12.2, 4.9, 1.9 Hz, 1H), 2.40 (s, 3H), 2.35 (d, J = 13.8 Hz, 1H), 2.05 (td, J = 12.2, 3.3 Hz, 1H), 1.84 (dt, J = 12.8, 3.3 Hz, 1H), 1.77 (td, J = 12.8, 4.8 Hz, 1H), 1.71 – 1.52 (m, 2H), 1.48 – 1.22 (m, 5H), 1.10 (qd, J = 12.3, 3.5 Hz, 1H) ppm. ^{13}C NMR (101 MHz, $CDCl_3$) δ 159.0, 146.1, 137.4, 130.9, 129.6 (q, J = 309.0 Hz), 109.4 (q, J = 2.2 Hz), 108.6, 57.6, 56.2, 47.1, 45.2, 42.8, 41.9, 37.8, 36.7, 26.7, 26.4, 23.2, 22.2 ppm. ^{19}F NMR (376 MHz, $CDCl_3$) δ –42.4 (s) ppm. ν_{max}(neat)/cm^{-1} 2931, 1490, 1111, 904, 726. HRMS (HESI) m/z calcd. for $C_{19}H_{25}ONF_3S$ [M+H]$^+$: 372.1603; found: 372.1597.

2-((1-(4-(4-((Trifluoromethyl)thio)phenoxy)phenoxy)propan-2-yl)oxy)pyridine 5u[15]

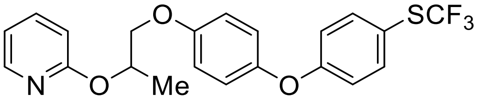

Pyriproxyfen (64.3 mg, 0.2 mmol), sulfoxide **1b** (49 mg, 0.24 mmol), Tf_2O (84 µL, 0.5 mmol) and Et_2NH (114 µL, 1.1 mmol) were reacted following general procedure B (eluent: hexane/CH_2Cl_2 = 1:1) to afford the title compound as a colorless oil (41.3 mg, 49%). ^{1}H NMR (400 MHz, $CDCl_3$) δ 8.16 (dd, J = 5.2, 2.0 Hz, 1H), 7.63 – 7.50 (m, 3H), 7.02 – 6.94 (m, 4H), 6.94 – 6.90 (m, 2H), 6.87 (ddd, J = 7.0, 5.0, 1.0 Hz, 1H), 6.75 (d, J = 8.4 Hz, 1H), 5.68 – 6.52 (m, 1H), 4.21 (dd, J = 9.9, 5.3 Hz, 1H), 4.09 (dd, J = 9.9, 4.8 Hz, 1H), 1.49 (d, J = 6.4 Hz, 3H) ppm. ^{13}C NMR (101 MHz, $CDCl_3$) δ 163.1, 161.3, 156.0, 148.7, 146.7, 138.7, 138.3, 129.5 (q, J = 308.3 Hz), 121.6, 117.6, 116.8, 116.4 (q, J = 2.0 Hz), 116.0, 111.7, 71.0, 69.2, 17.0 ppm. ^{19}F NMR (376 MHz, $CDCl_3$) δ –43.6 (s) ppm. ν_{max}(neat)/cm^{-1} 3355, 1596, 1487, 1224, 1114, 1083, 730. HRMS (ASAP) m/z calcd. for $C_{21}H_{19}O_3NF_3S$ [M+H]$^+$: 422.1032; found: 422.1032.

((8*R*,9*S*,13*S*,14*S*)-3,17-Dimethoxy-13-methyl-7,8,9,11,12,13,14,15,16,17-decahydro-6H-cyclopenta[*a*]phenanthren-2-yl)(trifluoromethyl)sulfide 5v

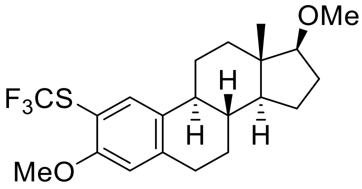

Estradiol dimethyl ether (60.1 mg, 0.2 mmol), sulfoxide **1b** (49 mg, 0.24 mmol), Tf_2O (84 µL, 0.5 mmol) and Et_2NH (114 µL, 1.1 mmol) were reacted following general procedure B (eluent: hexane/EtOAc = 19:1) to afford the title compound as a white solid (45.7 mg, 57%). m.p. 151 – 152 ºC. ^1H NMR (400 MHz, $CDCl_3$) δ 7.50 (s, 1H), 6.67 (s, 1H), 3.86 (s, 3H), 3.38 (s, 3H), 3.32 (t, J = 8.3 Hz, 1H), 2.93 – 2.85 (m, 2H), 2.33 – 2.24 (m, 1H), 2.18 (td, J = 10.8, 4.1 Hz, 1H), 2.13 – 2.00 (m, 2H), 1.90 (ddd, J = 9.9, 4.7, 2.6 Hz, 1H), 1.75 – 1.63 (m, 1H), 1.55 – 1.27 (m, 6H), 1.27 – 1.14 (m, 1H), 0.80 (s, 3H) ppm. ^{13}C NMR (126 MHz, $CDCl_3$) δ 158.4, 142.5, 136.1, 133.6, 129.6 (q, J = 309.1 Hz), 112.0, 108.9 (q, J = 1.7 Hz), 90.7, 57.9, 56.1, 50.2, 43.6, 43.2, 38.3, 37.8, 30.0, 27.7, 26.9, 26.3, 23.0, 11.5 ppm. ^{19}F NMR (376 MHz, $CDCl_3$) δ –42.8 (s) ppm. ν_{max}(neat)/cm^{-1} 2940, 2248, 1599, 1490, 1259, 1103, 905, 727. HRMS (ASAP) m/z calcd. for $C_{21}H_{27}O_2F_3S$ $[M]^+$: 400.1678; found: 400.1679.

5. Unsuccessful Substrates

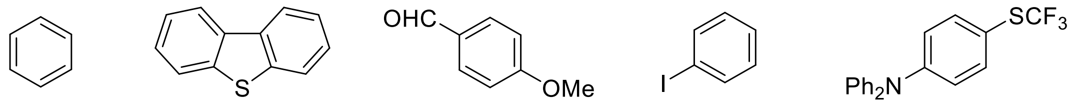

N,N-Diphenyl-4-((trifluoromethyl)thio)aniline[16]

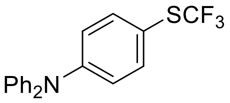

Triphenylamine (73.6 mg, 0.3 mmol), sulfoxide **1a** (41.6 mg, 0.2 mmol), Tf_2O (40 µL, 0.24 mmol) and Et_2NH (52 µL, 0.5 mmol) were reacted following general procedure A (eluent: hexane) to afford the title compound (35% NMR yield using CF_3CH_2OH as an internal standard according to quantitative ^{19}F NMR data). Due to difficulties with purification, an isolated yield is not provided, however, a sample for confirming the formation of the trifluoromethylthiolated product was obtained. ^1H NMR (400 MHz, $CDCl_3$) δ 7.54 (d, *J* = 8.7 Hz, 2H), 7.45 – 7.38 (m, 4H), 7.29 – 7.21 (m, 6H), 7.11 (d, *J* = 8.8 Hz, 2H) ppm. ^{19}F NMR (376 MHz, $CDCl_3$) *δ* –43.7 ppm.

6. Isolation of 7m

(2,5-Dimethylphenyl)(2-ethoxy-2-oxoethyl)(trifluoromethyl)sulfonium trifluoromethanesulfonate 7m

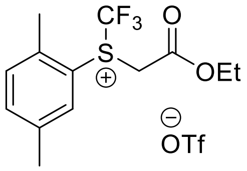

In an oven-dried 10 mL vial, a solution of *p*-xylene (38 µL, 0.3 mmol, 1.5 eq.) and sulfoxide **1b** (40.8 mg, 0.2 mmol, 1.0 eq.) in $MeNO_2$ (1 mL, 0.2 M) was cooled to –25 °C. Tf_2O (40.3 µL, 0.24 mmol, 1.2 eq.) was added dropwise and the mixture was stirred for 10 min, then warmed to room temperature and stirred for 15 h. The reaction was diluted with CH_2Cl_2 (5 mL), followed by slow addition of Et_2O (5 mL) to allow precipitation of **7m**. The product was obtained by filtration and drying under vacuum as a white solid (35 mg, 40%). ^{1}H NMR (400 MHz, $CDCl_3$) δ 7.70 (s, 1H), 7.56 (d, *J* = 7.9 Hz, 1H), 7.44 (d, *J* = 8.0 Hz, 1H), 5.68 (dd, *J* = 16.2, 1.4 Hz, 1H), 5.38 (d, *J* = 16.3 Hz, 1H), 4.36 (q, *J* = 7.2 Hz, 2H), 2.74 (s, 3H), 2.48 (s, 3H), 1.32 (t, *J* = 7.2 Hz, 3H) ppm. ^{13}C NMR (101 MHz, $CDCl_3$) δ (2 × $\mathit{C}F_3$ missing) 161.8, 142.1, 140.7, 138.1, 133.1, 130.1, 114.7, 65.0, 45.2, 20.9, 19.7, 13.7. ^{19}F NMR (376 MHz, $CDCl_3$) δ –51.7, –78.6 ppm. HRMS (HESI) m/z calcd. for $C_{13}H_{16}O_2F_3S$ $[M – OTf]^+$: 293.0818; found: 293.0804.

7. X-Ray Structures of 1a and 7m

(((Trifluoromethyl)sulfinyl)methyl)benzene 1a

CCDC 1993042

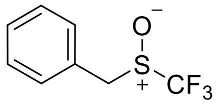

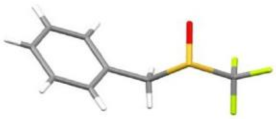

Table S1. Crystal data and details of data collection and refinement for compound **1a**

Bond precision	C-C = 0.0109 Å	Wavelength	0.71073
Cell	a = 8.7191 (13)	b = 5.1444 (8)	C = 20.007 (3)
	alpha = 90	beta = 90	gamma = 90
Temperature	150 K		
	Calculated	Reported	
Volume	897.4 (2)	897.4 (2)	
Space group	P n a 21	P n a 21	
Hall group	P 2c -2n	P 2c -2n	
Moiety formula	$C_8H_7F_3OS$	$C_8H_7F_3OS$	
Sum formula	$C_8H_7F_3OS$	$C_8H_7F_3OS$	
Mr	208.20	208.20	
Dx,g cm^{-3}	1.541	1.541	
Z	4	4	
Mu (mm^{-1})	0.363	0.363	
F000	424.0	424.0	
F000’	424.81		
h,k,lmax	12, 7, 27	11,7,27	
Nref	2549 [1309]	2254	
Tmin,Tmax	0.350, 0.696	0.027, 1.000	
Tmin’	0.324		
Correction method = # Reported T Limits: Tmin = 0.027 Tmax = 1.000 AbsCorr = MULTI-SCAN			
Data completeness	1.72/0.88	Theta(max)	29.720
R(reflections)	0.0741 (1447)	wR2(reflections)	0.2161 (2254)
S	0.982	Npar	118

(2,5-Dimethylphenyl)(2-ethoxy-2-oxoethyl)(trifluoromethyl)sulfonium trifluoromethanesulfonate 7m

CCDC1993043

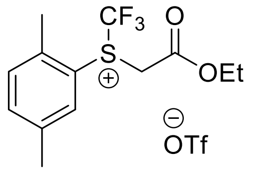

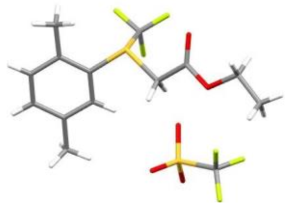

Table S2. Crystal data and details of data collection and refinement for compound **7m**

Bond precision	C-C = 0.0095 Å	Wavelength	0.71073
Cell	a = 32.1117 (12)	b = 7.4041 (3)	C = 7.7128 (5)
	alpha = 90	beta = 90	gamma = 90
Temperature	150 K		
	Calculated	Reported	
Volume	1833.78 (16)	1833.78 (16)	
Space group	P c a 21	P c a 21	
Hall group	P 2c -2ac	P 2c -2ac	
Moiety formula	$C_{13}H_{16}F_3O_2S$, CF_3O_3S	$C_{13}H_{16}F_3O_2S$, CF_3O_3S	
Sum formula	$C_{14}H_{16}F_6O_5S_2$	$C_{14}H_{16}F_6O_5S_2$	
Mr	442.39	442.39	
Dx,g cm^{-3}	1.602	1.602	
Z	4	4	
Mu (mm^{-1})	0.372	0.372	
F000	904.0	904.0	
F000’	905.73		
h,k,lmax	38, 8, 9	38, 8, 9	
Nref	3224 [1745]	2940	
Tmin,Tmax	0.894, 0.928	0.717, 1.000	
Tmin’	0.894		
Correction method = # Reported T Limits: Tmin = 0.717 Tmax = 1.000 AbsCorr = MULTI-SCAN			
Data completeness	1.68/0.91	Theta(max)	24.998
R(reflections)	0.0510 (2556)	wR2(reflections)	0.1164 (2940)
S	1.061	Npar	247

8. Computational Studies

Density functional calculations were carried out to assess the relative barriers for the attack of Et_2NH on the cation of the **7m** salt, as depicted below.

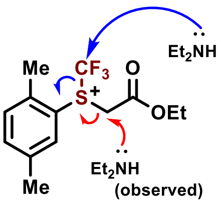

All calculations were performed using the Gaussian suite of programs.[17] Geometry optimisations were performed using the hybrid PBE0 functional[18] and the Def2-SVP basis set,[19] employing the "ultrafine" grid for quadrature. All stationary points were characterised as minima or saddle points using vibrational analysis. Optimisations were carried out in the gas phase, followed by treatment of solvation using the polarisable continuum model (PCM) with parameters for nitromethane solvent.

In the gas phase, the transition structure (TS1) for attack at the $–CH_2CO_2Et$ group lies 36.4 kJ $mol^{–1}$ lower than the transition structure (TS2) for attack at the $–CF_3$ group. Inclusion of solvent polarisation increases the difference to 40.8 kJ $mol^{–1}$ making the reaction at the $–CH_2CO_2Et$ group significantly more facile (see below). The barriers relative to isolated reactants are shown in the figure below.

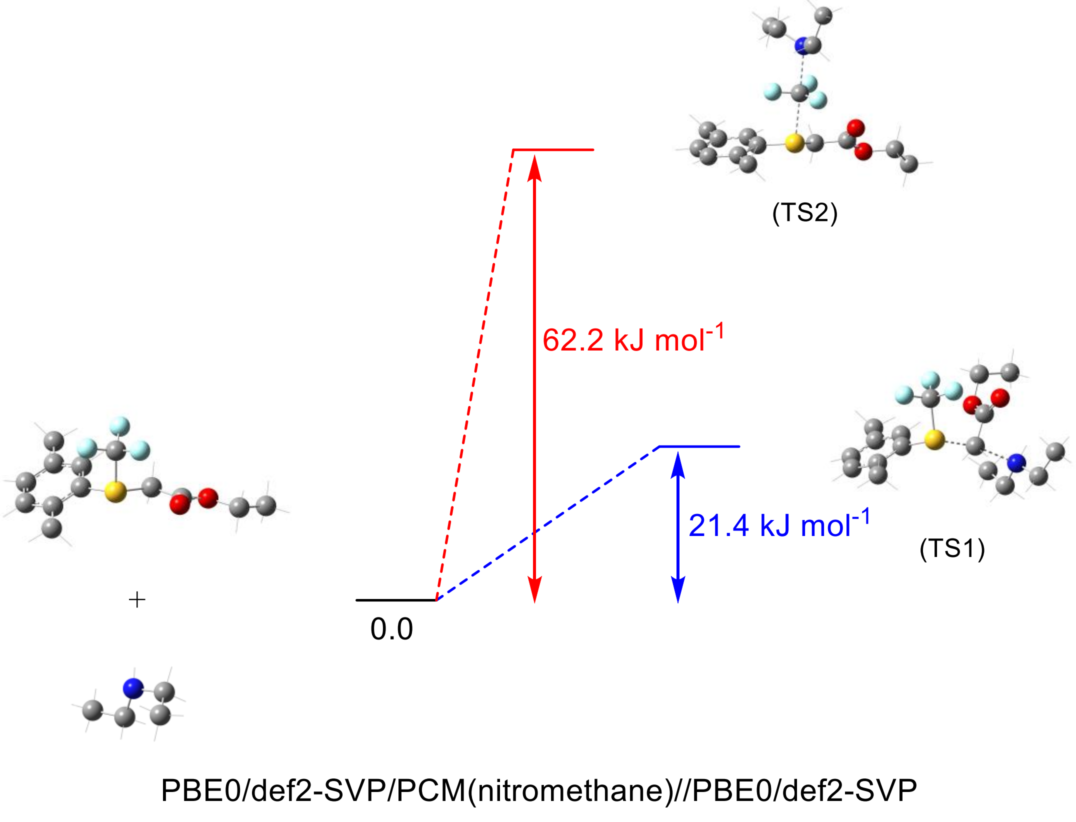

Coordinates and energies of computed structures

(All data correspond to PBE0/Def2-SVP/PCM(nitromethane)//PBE0/Def2-SVP)

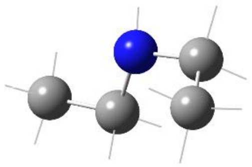

Et_2NH (minimum)

E = -213.379472286

N	-0.046404000	0.580746000	0.308866000
H	-0.417121000	1.527652000	0.325819000
C	1.308291000	0.597872000	-0.203505000
C	2.101638000	-0.641184000	0.180365000
H	1.812311000	1.483277000	0.217630000
H	1.341787000	0.721823000	-1.310971000
H	3.143418000	-0.560834000	-0.165994000
H	2.103094000	-0.769661000	1.273355000
H	1.679148000	-1.555122000	-0.265010000
C	-0.968554000	-0.277072000	-0.401203000
C	-2.364460000	-0.188943000	0.185344000
H	-0.611745000	-1.318415000	-0.325336000
H	-1.006988000	-0.053381000	-1.493372000
H	-3.058567000	-0.867192000	-0.332898000
H	-2.350870000	-0.449638000	1.254225000
H	-2.771126000	0.832232000	0.094484000

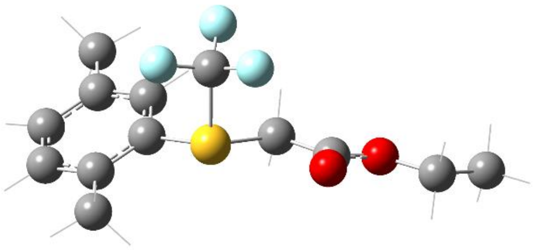

Cation of 7m (minimum)

E = -1351.00232077

S	0.000202000	-0.662307000	-0.441957000
O	3.462447000	1.073108000	-0.272295000
F	-0.126133000	-0.745030000	2.201634000
F	-1.023666000	-2.406590000	1.165004000
F	1.120802000	-2.255485000	1.322757000

O	2.758967000	-1.031290000	-0.663205000
C	0.003348000	-1.598673000	1.210379000
C	2.572882000	0.122726000	-0.384131000
C	-1.886139000	1.174974000	0.432028000
H	-1.131369000	1.566134000	1.116618000
C	-3.149727000	1.760669000	0.418767000
C	-2.540291000	-0.453953000	-1.309411000
C	-1.600033000	0.098136000	-0.419257000
C	-4.098299000	1.224177000	-0.467619000
H	-5.099316000	1.662545000	-0.504266000
C	4.851008000	0.715230000	-0.502735000
H	5.110668000	-0.090816000	0.200017000
H	4.926548000	0.306837000	-1.521764000
C	-3.803086000	0.152136000	-1.299496000
H	-4.573037000	-0.234262000	-1.971503000
C	-2.252706000	-1.625921000	-2.202274000
H	-1.370945000	-1.459233000	-2.840199000
H	-3.106508000	-1.820688000	-2.863518000
H	-2.073965000	-2.544579000	-1.620863000
C	1.178064000	0.667470000	-0.092401000
H	0.912132000	1.500107000	-0.762009000
H	1.087321000	1.012265000	0.948066000
C	-3.492065000	2.914707000	1.311227000
H	-4.334835000	2.661076000	1.972679000
H	-3.799061000	3.790605000	0.719002000
H	-2.644064000	3.211900000	1.942522000
C	5.689544000	1.946898000	-0.308512000
H	5.593843000	2.338726000	0.714170000
H	5.407882000	2.738179000	-1.017795000
H	6.746135000	1.696059000	-0.479803000

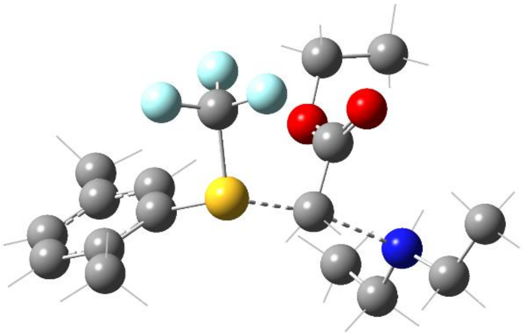

TS1 (saddle point)

E = -1564.37365559

S	-0.919397000	-1.197098000	-0.798421000
O	1.369908000	0.503741000	1.379126000
F	-0.840959000	-1.545764000	1.831354000
F	-2.364190000	-2.642985000	0.793920000
F	-0.303523000	-3.257092000	0.636846000
O	2.140868000	-1.544843000	0.860379000
C	-1.115796000	-2.234764000	0.740686000
C	1.596956000	-0.516405000	0.562227000
C	-1.825223000	1.117604000	0.392705000
H	-0.886447000	1.108066000	0.951275000
C	-2.743234000	2.146083000	0.602375000
C	-3.292580000	0.046676000	-1.275738000
C	-2.103555000	0.095656000	-0.524415000
C	-3.931730000	2.111134000	-0.141854000
H	-4.671576000	2.904639000	-0.005180000
C	1.820342000	0.363947000	2.745690000
H	1.174623000	1.047390000	3.312476000
H	1.623370000	-0.668182000	3.068193000
C	-4.195814000	1.092895000	-1.049849000
H	-5.135971000	1.101372000	-1.606766000
C	-3.613486000	-1.054442000	-2.242840000
H	-2.847395000	-1.152405000	-3.027503000
H	-4.575490000	-0.865582000	-2.736287000
H	-3.684889000	-2.027640000	-1.732652000
C	1.068421000	-0.250255000	-0.816488000
H	1.405656000	-0.920797000	-1.603100000
H	0.650650000	0.721819000	-1.077513000
C	-2.479958000	3.243766000	1.589709000

H	-3.202117000	3.205045000	2.420558000
H	-2.583018000	4.233849000	1.120009000
H	-1.471057000	3.172929000	2.018589000
C	3.278691000	0.722286000	2.891875000
H	3.476047000	1.746987000	2.543148000
H	3.918761000	0.016270000	2.343080000
H	3.562954000	0.669370000	3.953205000
N	3.050242000	0.779465000	-1.311391000
H	3.621349000	0.858645000	-0.467612000
C	3.740566000	-0.064885000	-2.288092000
C	4.324669000	-1.322467000	-1.677632000
H	4.541147000	0.521206000	-2.778588000
H	3.021571000	-0.311970000	-3.089795000
H	4.824393000	-1.916778000	-2.455328000
H	5.082605000	-1.078956000	-0.916010000
H	3.564626000	-1.954454000	-1.196584000
C	2.731289000	2.115859000	-1.811412000
C	2.294264000	3.069931000	-0.718764000
H	1.945602000	2.012484000	-2.580911000
H	3.610714000	2.532514000	-2.338678000
H	2.067411000	4.055009000	-1.149920000
H	1.400662000	2.717483000	-0.183543000
H	3.093389000	3.214656000	0.025703000

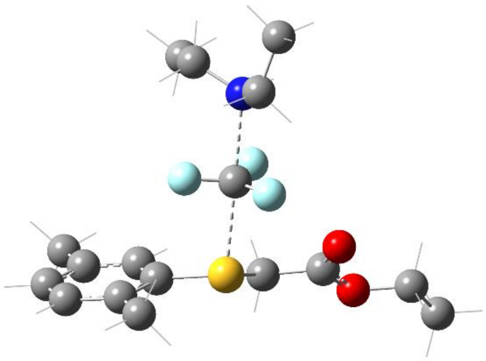

TS2 (saddle point)

E = -1564.3580962

S	-0.854442000	-0.590555000	-0.970398000
O	-3.981213000	1.207067000	0.326337000
F	0.355892000	1.143713000	0.894307000
F	1.789919000	0.003607000	-0.350189000
F	0.694201000	1.710010000	-1.219774000
O	-2.079331000	1.983682000	-0.581507000
C	0.899621000	0.918366000	-0.245823000
C	-2.734482000	1.108614000	-0.073913000
C	-0.396601000	-2.725180000	0.770457000
H	-0.961074000	-2.185522000	1.532980000
C	0.130987000	-3.975046000	1.099071000
C	0.500804000	-2.863947000	-1.508055000
C	-0.219463000	-2.182855000	-0.507835000
C	0.852935000	-4.655370000	0.109403000
H	1.279667000	-5.636423000	0.334279000
C	-4.629767000	2.486626000	0.158707000
H	-4.048914000	3.240356000	0.712630000
H	-4.583814000	2.754728000	-0.907870000
C	1.031562000	-4.110044000	-1.156022000
H	1.594378000	-4.669851000	-1.907337000
C	0.699342000	-2.302066000	-2.885789000
H	-0.259676000	-2.045620000	-3.363129000
H	1.212641000	-3.027701000	-3.530167000
H	1.311935000	-1.385362000	-2.875019000
C	-2.173371000	-0.269883000	0.220615000
H	-2.942492000	-1.050107000	0.131327000
H	-1.786771000	-0.274468000	1.251739000
C	-0.077340000	-4.578942000	2.456301000

H	-0.717955000	-5.472949000	2.392642000
H	-0.555721000	-3.874136000	3.150173000
H	0.877630000	-4.899182000	2.899819000
C	-6.041844000	2.364988000	0.661622000
H	-6.063746000	2.093172000	1.726743000
H	-6.601326000	1.606771000	0.095228000
H	-6.557321000	3.328865000	0.543778000
N	2.575406000	2.320851000	0.422958000
C	3.119075000	3.067503000	-0.717484000
C	3.590198000	1.615316000	1.211577000
C	3.980838000	4.269377000	-0.362047000
H	2.269359000	3.404712000	-1.330572000
H	3.689767000	2.354855000	-1.336070000
C	3.051567000	1.032382000	2.503457000
H	4.429503000	2.293296000	1.446824000
H	4.011244000	0.819847000	0.573836000
H	4.290336000	4.789101000	-1.280418000
H	3.422884000	4.993553000	0.252888000
H	4.896696000	3.991462000	0.179322000
H	3.873755000	0.580857000	3.076356000
H	2.601481000	1.810355000	3.141052000
H	2.300431000	0.247102000	2.336373000
H	2.080219000	2.974221000	1.035068000

9. References

[1] Xu, C.; Ma, B.; Shen, Q., *Angew Chem., Int. Ed.* **2014,** *53*, 9316-20.

[2] Matheis, C.; Wang, M.; Krause, T.; Goossen, L. J., *Synlett* **2015,** *26*, 1628-1632.

[3] Jiang, M.; Zhu, F.; Xiang, H.; Xu, X.; Deng, L.; Yang, C., *Org. Biomol. Chem.* **2015,** *13*, 6935-6939.

[4] Ghosh, A.; Lecomte, M.; Kim-Lee, S.-H.; Radosevich, A. T., *Angew. Chem., Int. Ed.* **2019,** *58*, 2864-2869.

[5] Jiang, L.; Yan, Q.; Wang, R.; Ding, T.; Yi, W.; Zhang, W., *Chem. Eur. J.* **2018,** *24*, 18749-18756.

[6] Guo, J.-Y.; Dai, R.-H.; Xu, W.-C.; Wu, R.-X.; Tian, S.-K., *Chem. Commun.* **2018,** *54*, 8980-8982.

[7] Honeker, R.; Ernst, J. B.; Glorius, F., *Chem. Eur. J.* **2015,** *21*, 8047-8051.

[8] Zhao, X.; Wei, A.; Yang, B.; Li, T.; Li, Q.; Qiu, D.; Lu, K., *J. Org. Chem.* **2017,** *82*, 9175-9181.

[9] Zhang, P.; Li, M.; Xue, X.-S.; Xu, C.; Zhao, Q.; Liu, Y.; Wang, H.; Guo, Y.; Lu, L.; Shen, Q., *J. Org. Chem.* **2016,** *81*, 7486-7509.

[10] Shen, F.; Zheng, H.; Xue, X.-S.; Lu, L.; Shen, Q., *Org. Lett.* **2019,** *21*, 6347-6351.

[11] Weng, Z.; He, W.; Chen, C.; Lee, R.; Tan, D.; Lai, Z.; Kong, D.; Yuan, Y.; Huang, K.-W., *Angew. Chem., Int. Ed.* **2013,** *52*, 1548-1552.

[12] Yang, X.-G.; Zheng, K.; Zhang, C., *Org. Lett.* **2020,** *22*, 2026-2031.

[13] Pluta, R.; Nikolaienko, P.; Rueping, M., *Angew. Chem., Int. Ed.* **2014,** *53*, 1650-1653.

[14] Yan, Q.; Jiang, L.; Yi, W.; Liu, Q.; Zhang, W., *Adv. Synth. Catal.* **2017,** *359*, 2471-2480.

[15] Berger, F.; Plutschack, M. B.; Riegger, J.; Yu, W.; Speicher, S.; Ho, M.; Frank, N.; Ritter, T., *Nature* **2019,** *567*, 223-228.

[16] Teverovskiy, G.; Surry, D. S.; Buchwald, S. L., *Angew. Chem., Int. Ed.* **2011,** *50*, 7312-7314.

[17] Gaussian 09, Revision D.01, Frisch, M. J.; Trucks, G. W.; Schlegel, H. B.; Scuseria, G. E.; Robb, M. A.; Cheeseman, J. R.; Scalmani, G.; Barone, V.; Mennucci, B.; Petersson, G. A.; Nakatsuji, H.; Caricato, X. Li, H. P. Hratchian, A. F. Izmaylov, J. Bloino, G. Zheng, J. L. Sonnenberg, M. Hada, M. Ehara, M.; Toyota, K.; Fukuda, R.; Hasegawa, J.; Ishida, M.; Nakajima, T.; Honda, Y.; Kitao, O.; Nakai, H.; Vreven, T.; Montgomery, J. A. Jr.; Peralta, J. E.; Ogliaro, F.; Bearpark, M.; Heyd, J. J.; Brothers, E.; Kudin, K. N.; Staroverov, V. N.; Keith, T.; Kobayashi, R.; Normand, J.; Raghavachari, K.; Rendell, A.; Burant, J. C.; Iyengar, S. S.; Tomasi, J.; Cossi, M.; Rega, N.; Millam, J. M.; Klene, M.; Knox, J. E.; Cross, J. B.; Bakken, V.; Adamo, C.; Jaramillo, J.; Gomperts, R.; Stratmann, R. E.; Yazyev, O.; Austin, A. J.; Cammi, R.; Pomelli, C.; Ochterski, J. W.; Martin, R. L.; Morokuma, K.; Zakrzewski, V. G.; Voth, G. A.; Salvador, P.; Dannenberg, J. J.; Dapprich, S.; Daniels, A. D.; Farkas, O.; Foresman, J. B.; Ortiz, J. V.; Cioslowski, J.; Fox, D. J. Gaussian, Inc., Wallingford CT, 2010.

[18] C. Adamo, V. Barone, *J. Chem. Phys.* **1999**, *110*, 6158–6170.

[19] F. Weigend, R. Ahlrichs, *Phys. Chem. Chem. Phys.* **2005**, *7*, 3297–3305.

10. ^{1}H, ^{13}C, and ^{19}F NMR Spectra of Synthesized Compounds

1a ^{1}H NMR (500 MHz, $CDCl_3$)

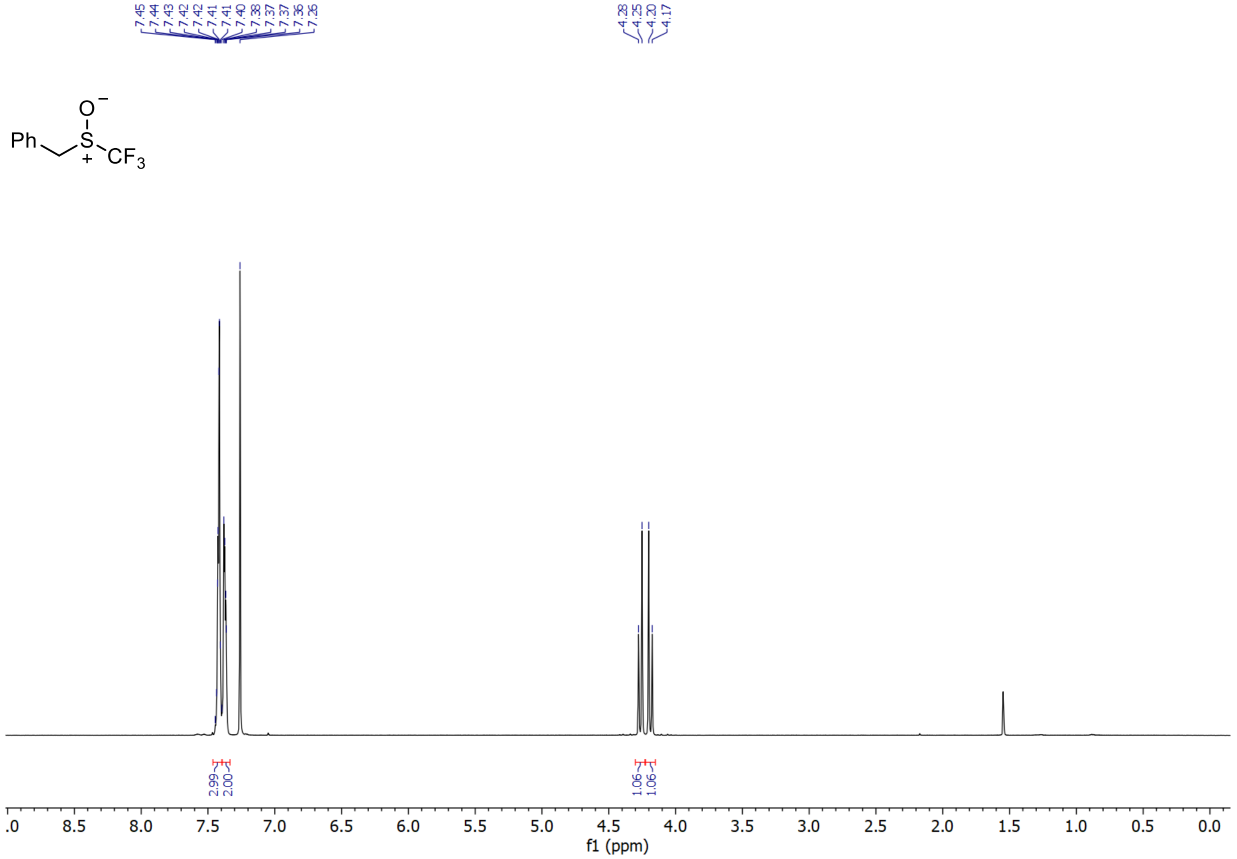

1a ^{13}C NMR (101 MHz, $CDCl_3$)

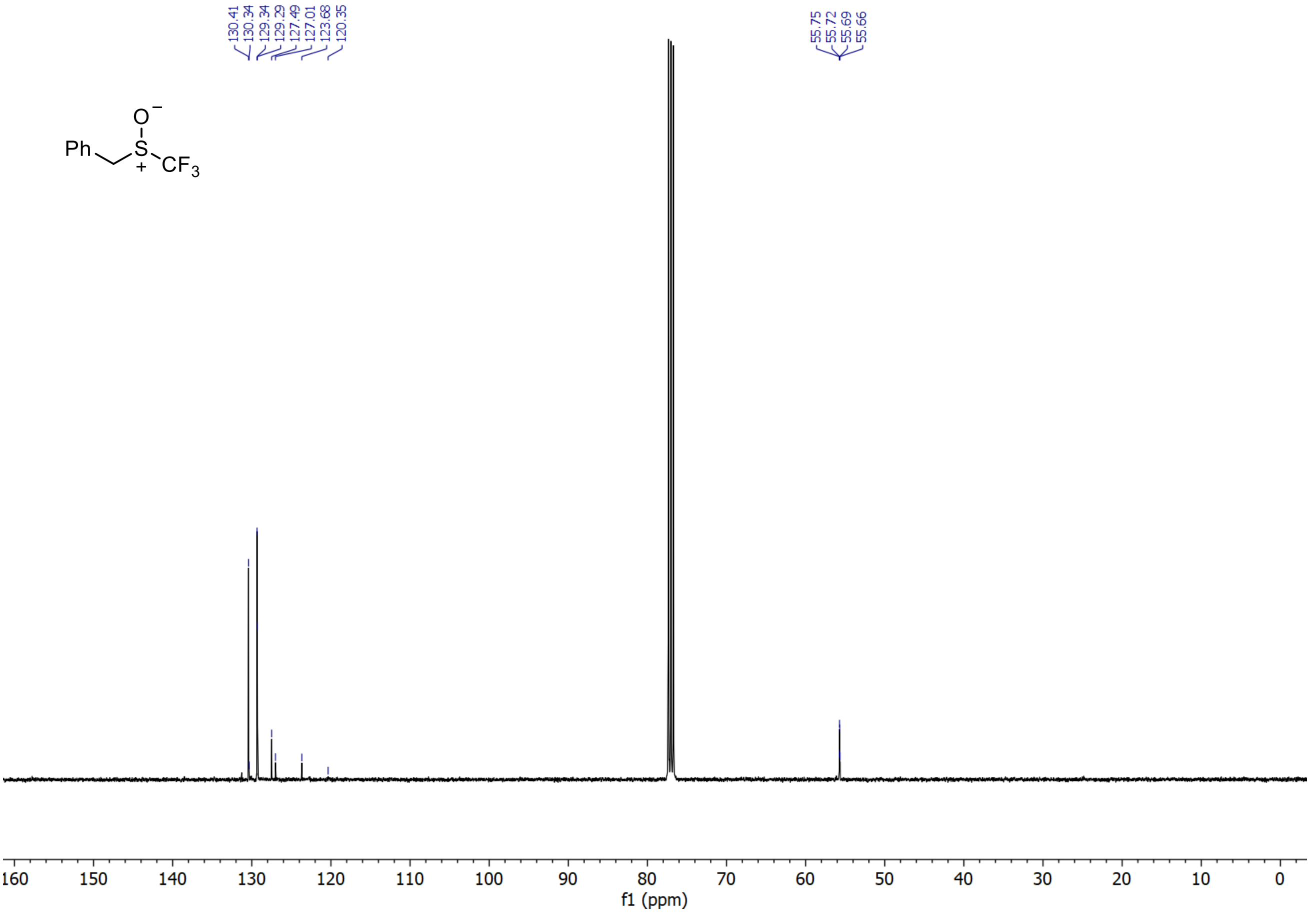

1a ^{19}F NMR (471 MHz, $CDCl_3$)

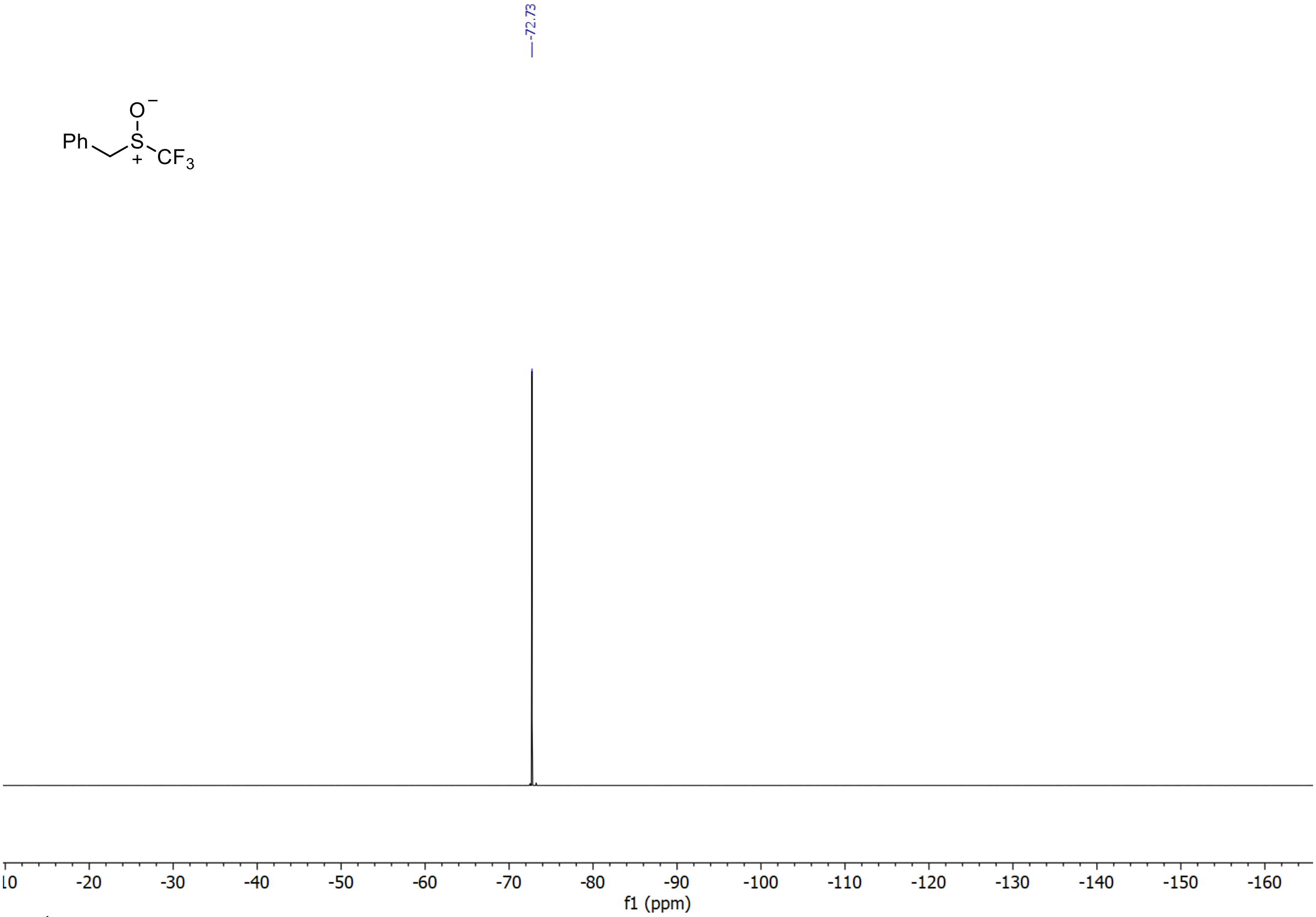

1b ^{1}H NMR (400 MHz, $CDCl_3$)

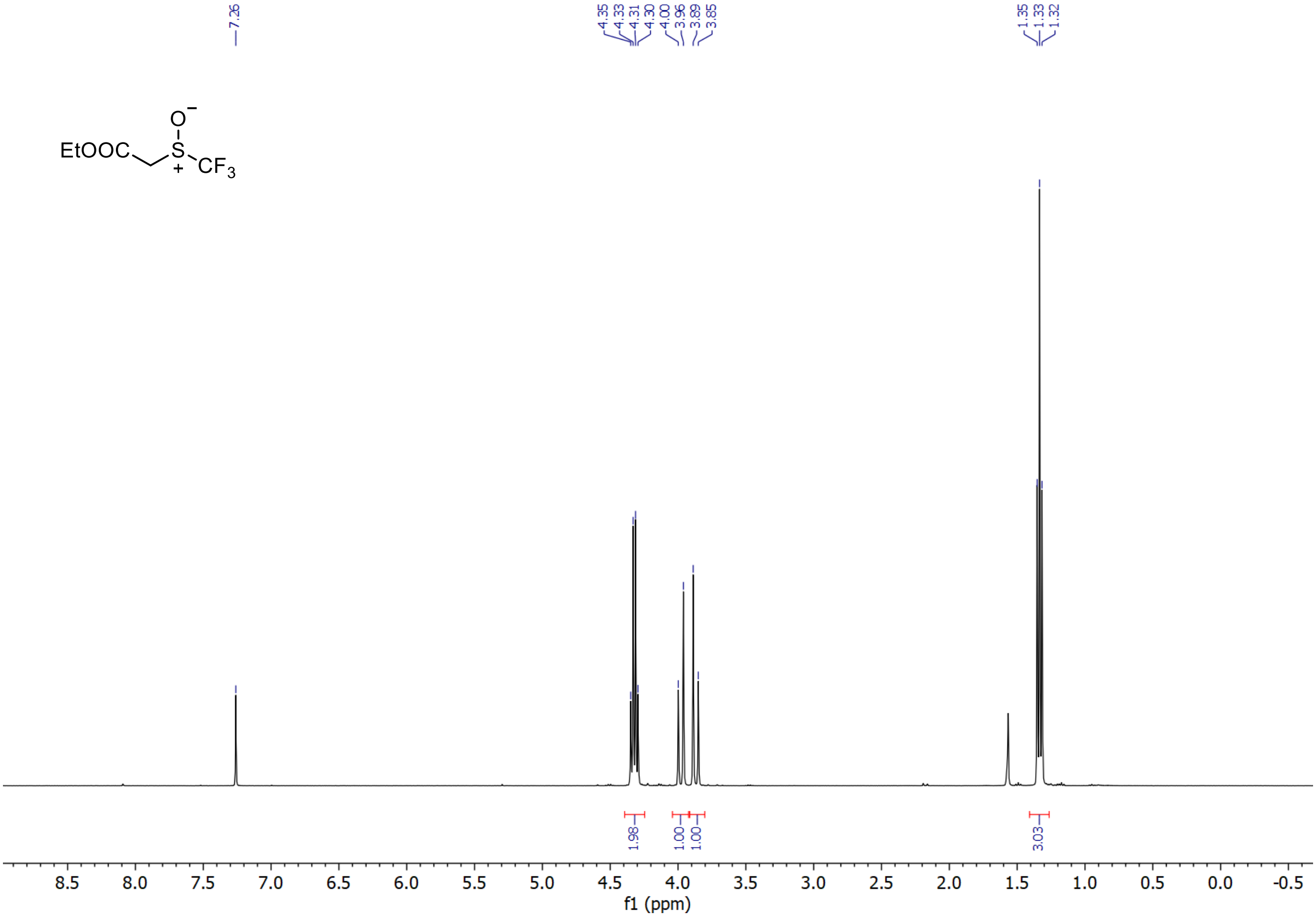

1b ^{13}C NMR (101 MHz, $CDCl_3$)

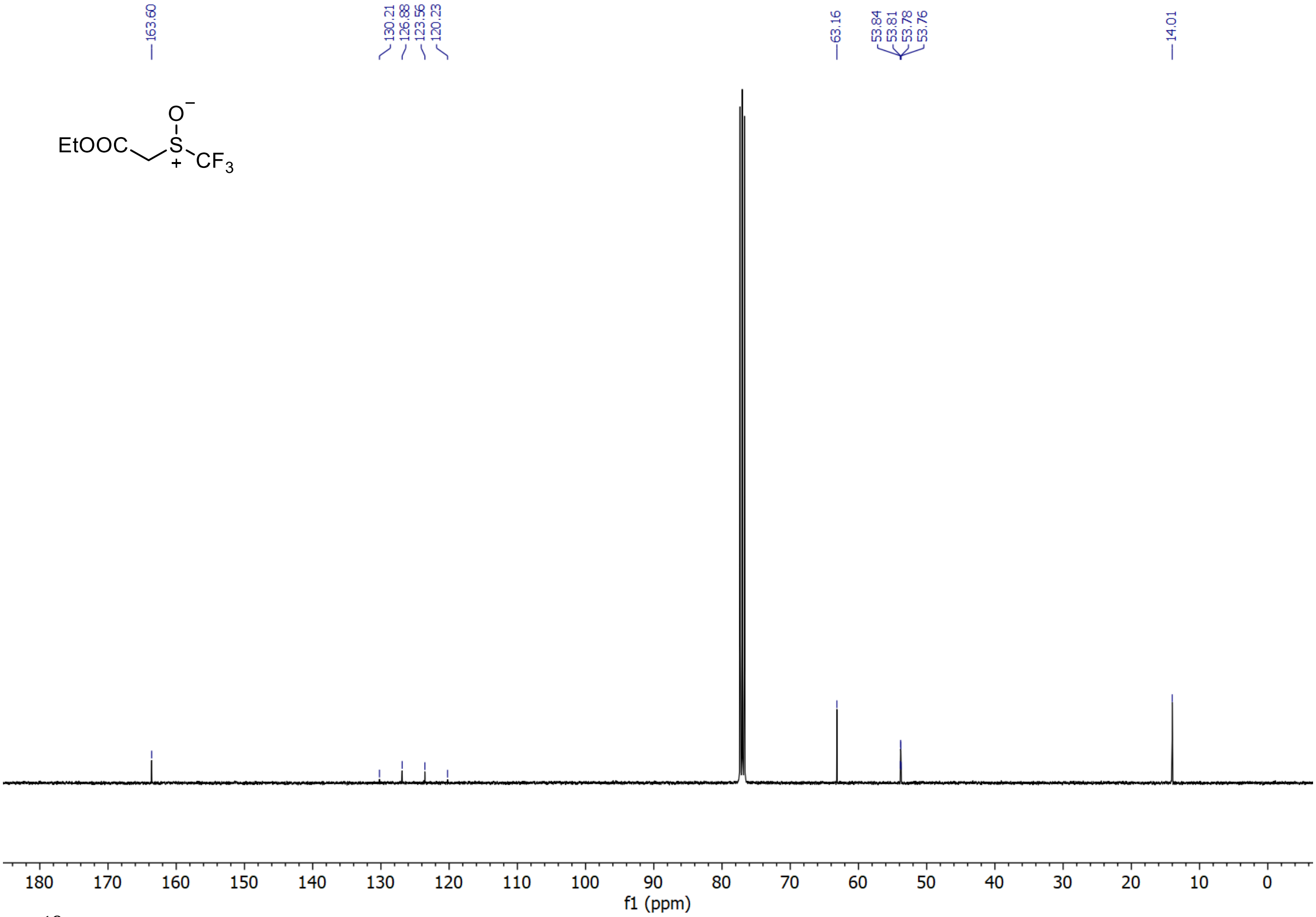

1b ^{19}F NMR (376 MHz, $CDCl_3$)

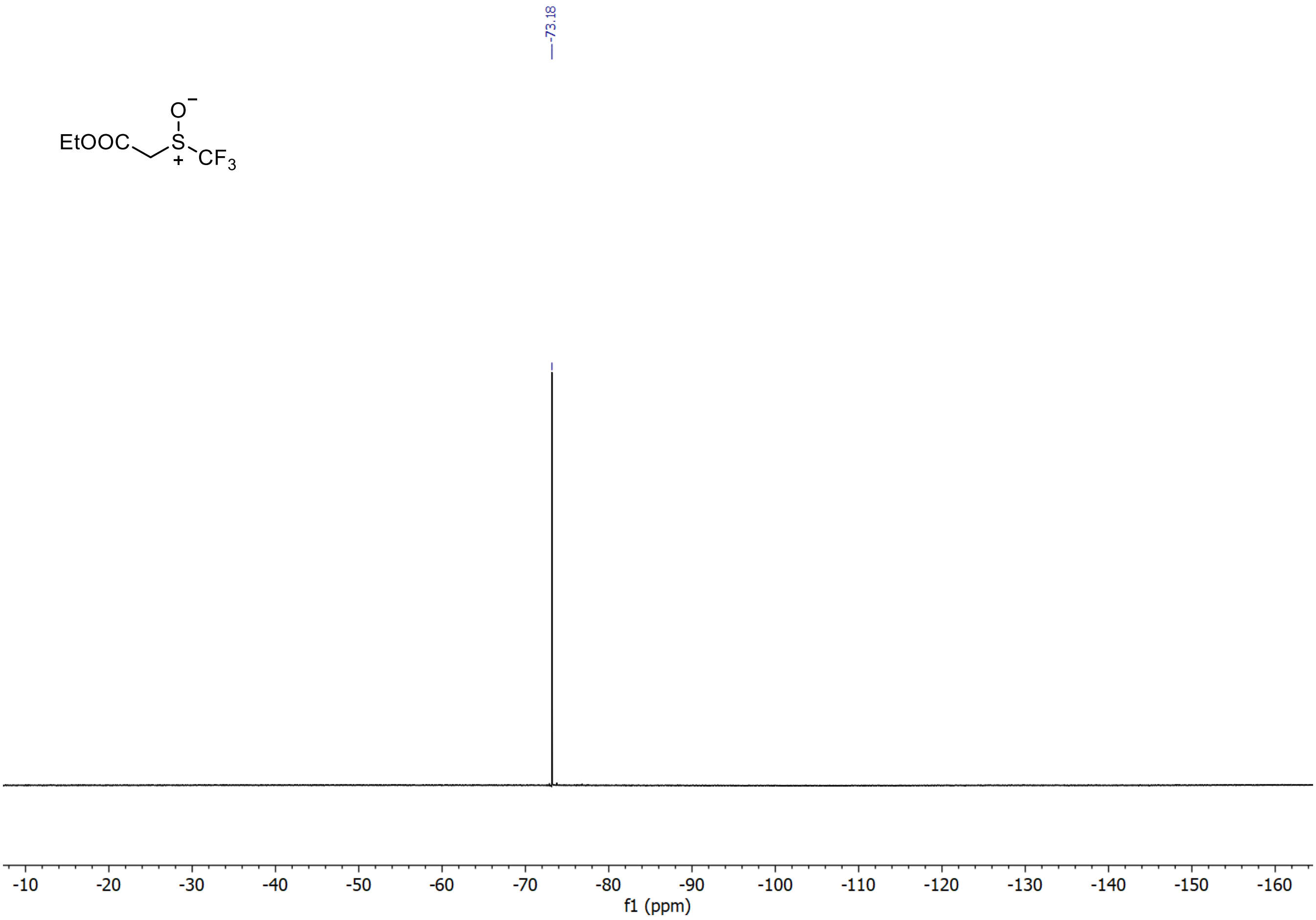

3a ^{1}H NMR (400 MHz, $CDCl_3$)

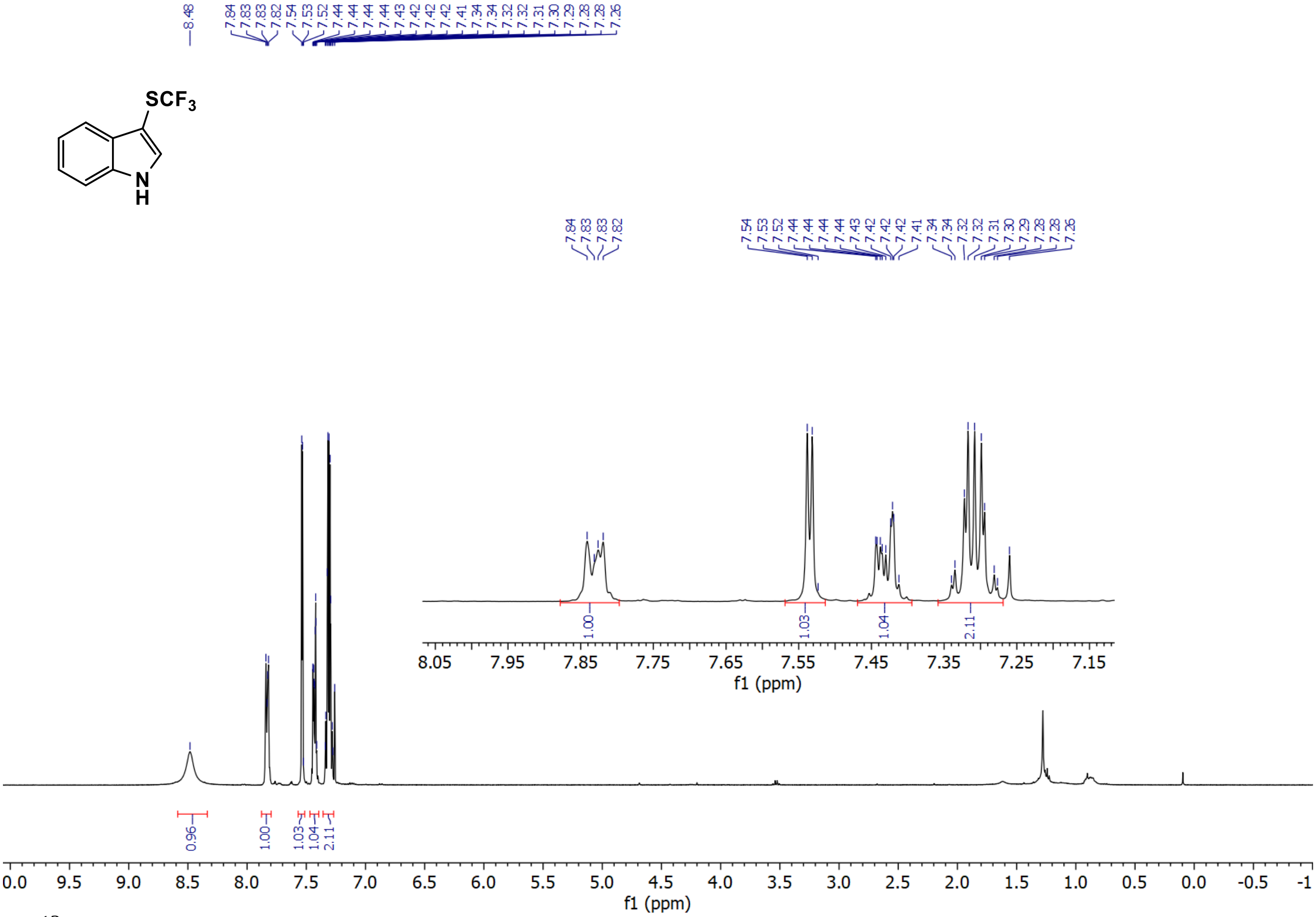

3a ^{13}C NMR (126 MHz, $CDCl_3$)

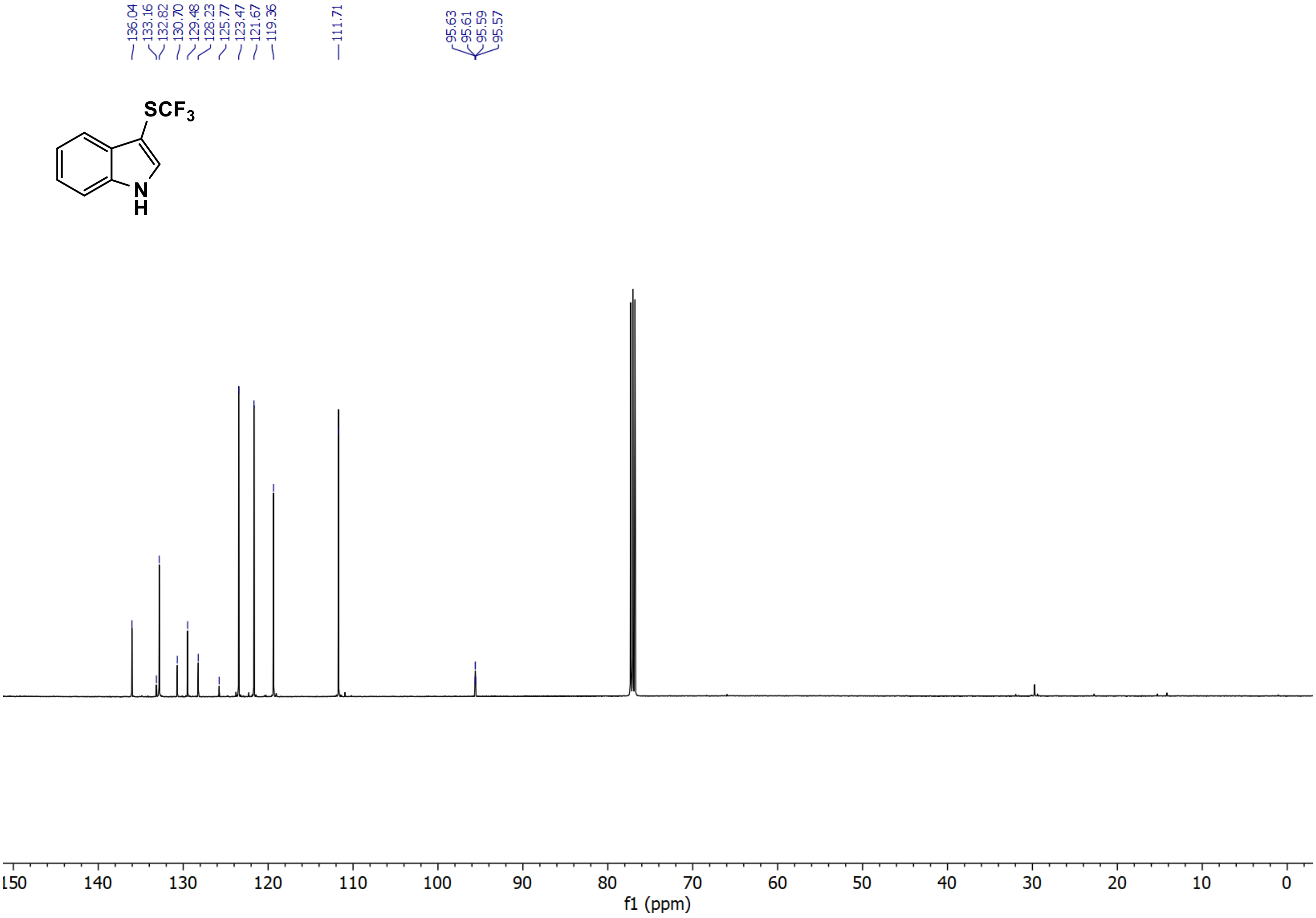

3a ^{19}F NMR (376 MHz, $CDCl_3$)

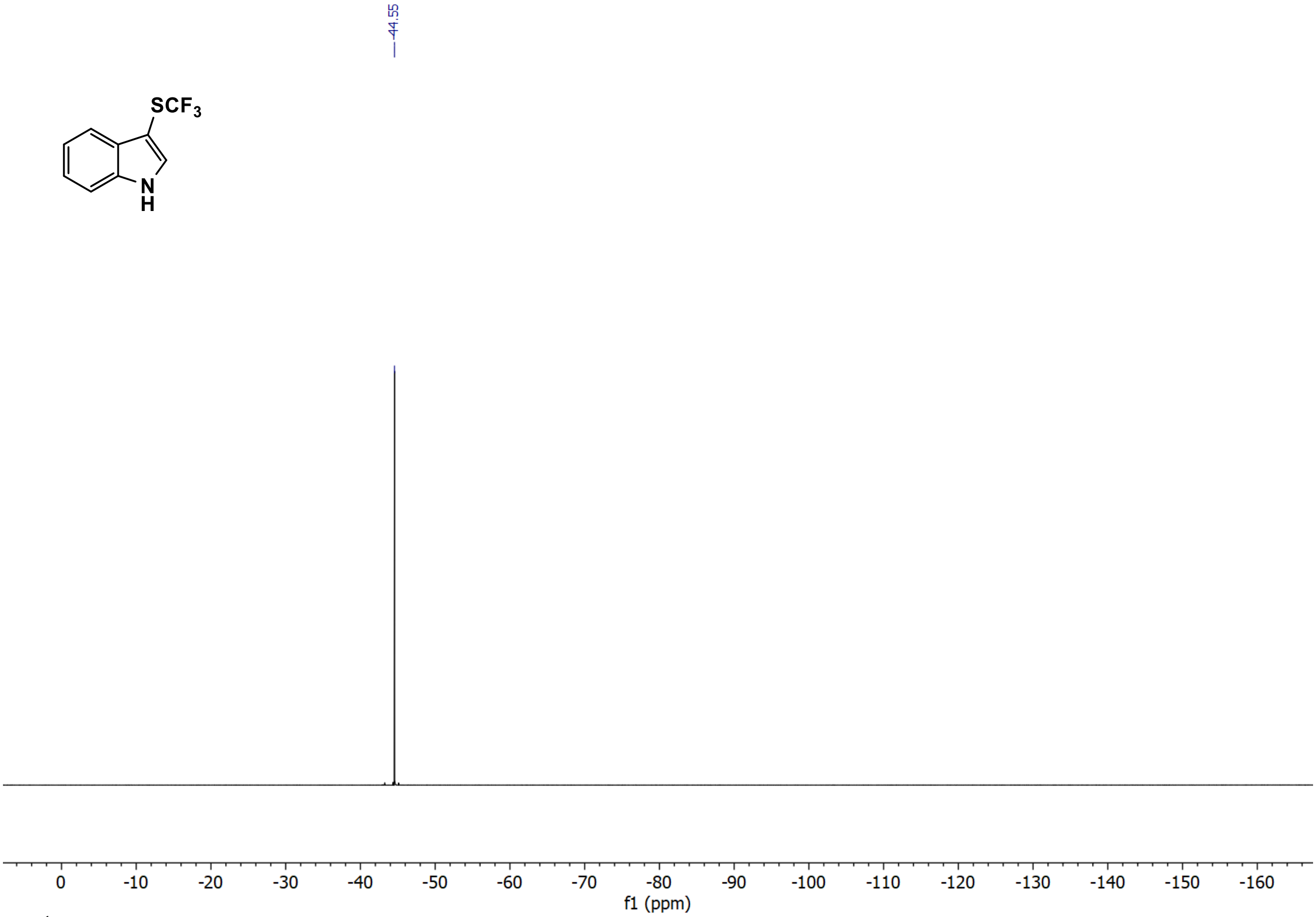

3b ^{1}H NMR (400 MHz, $CDCl_3$)

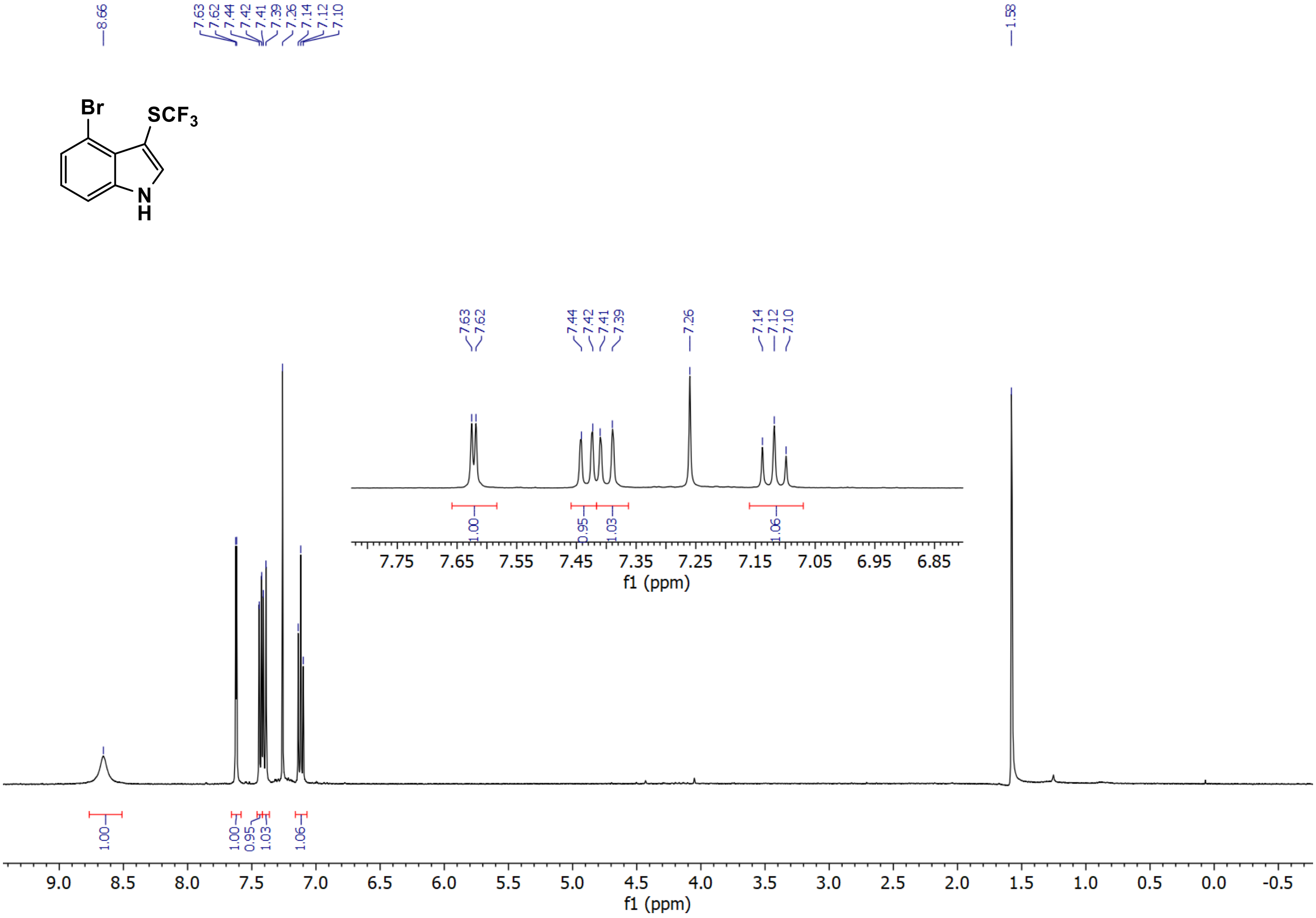

3b ^{13}C NMR (126 MHz, $CDCl_3$)

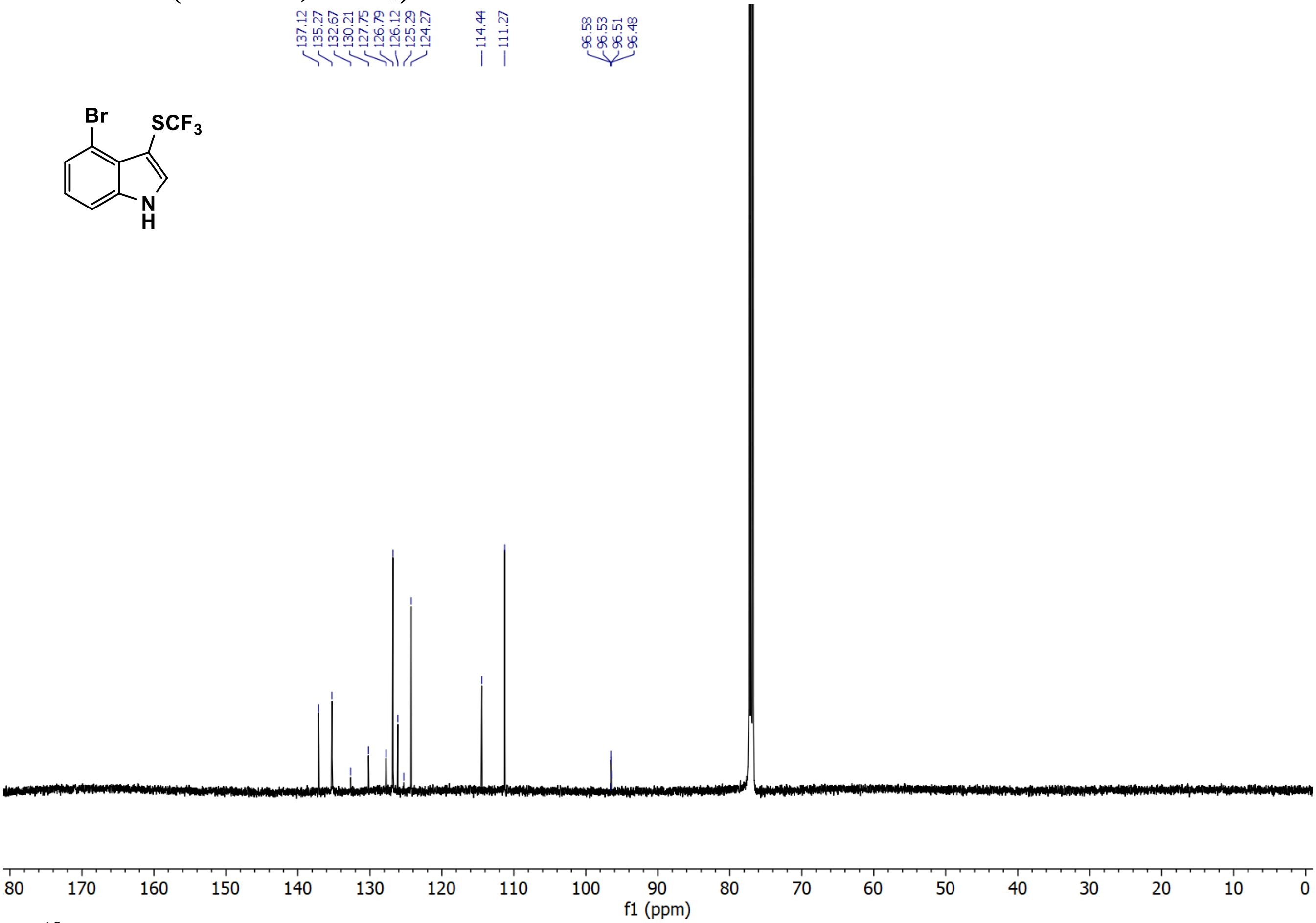

3b ^{19}F NMR (376 MHz, $CDCl_3$)

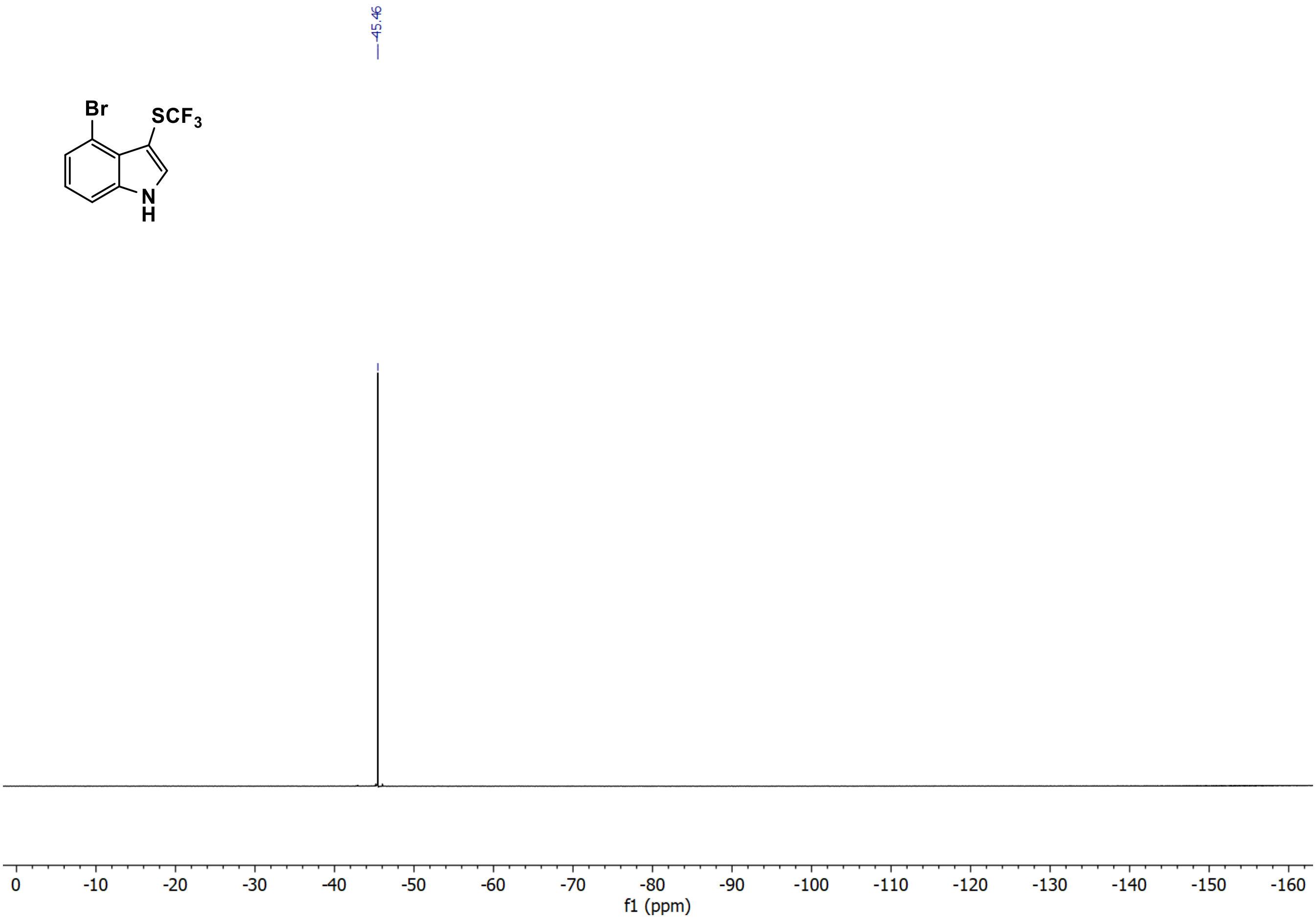

3c ^{1}H NMR (400 MHz, $CDCl_3$)

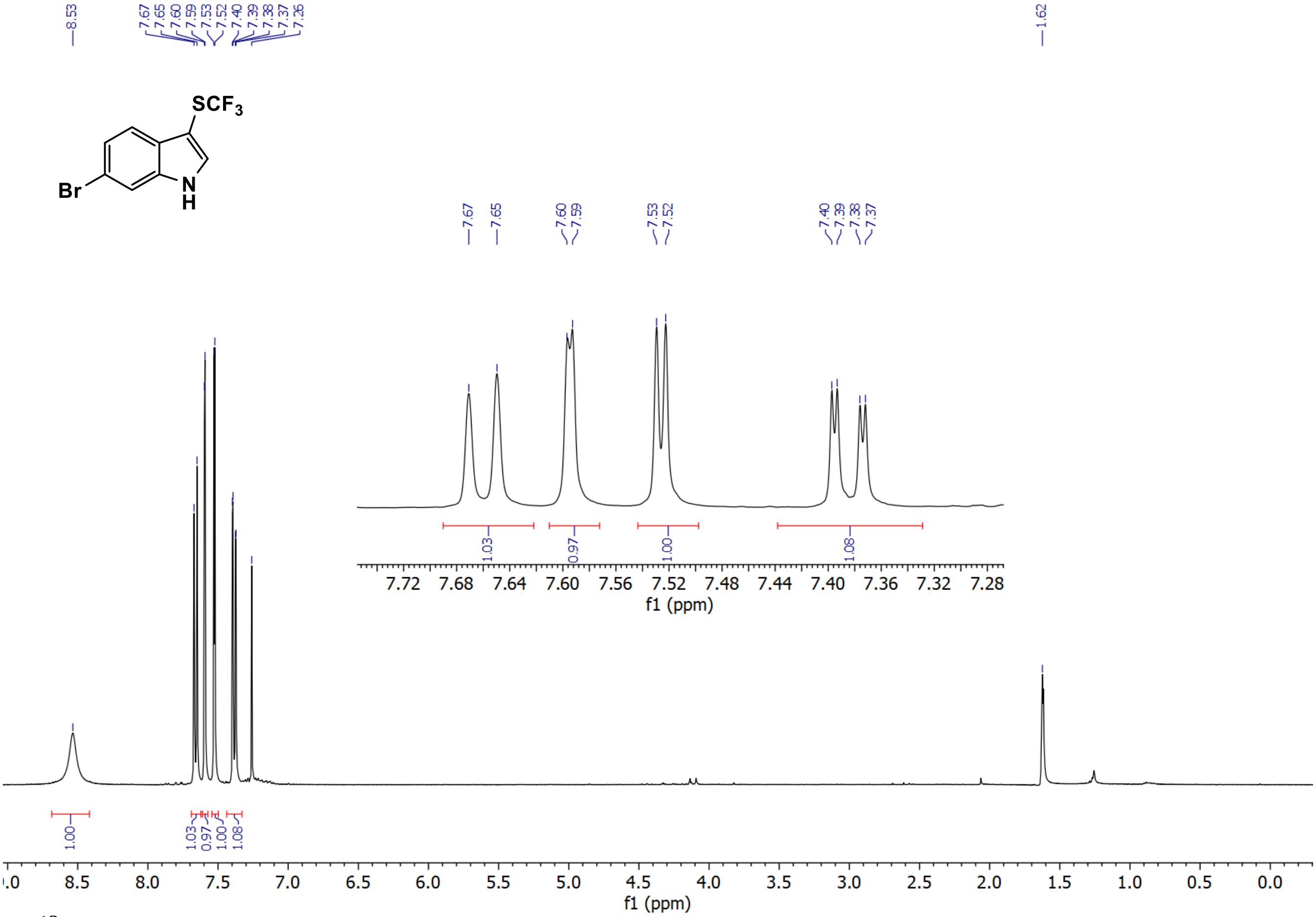

3c ^{13}C NMR (126 MHz, $CDCl_3$)

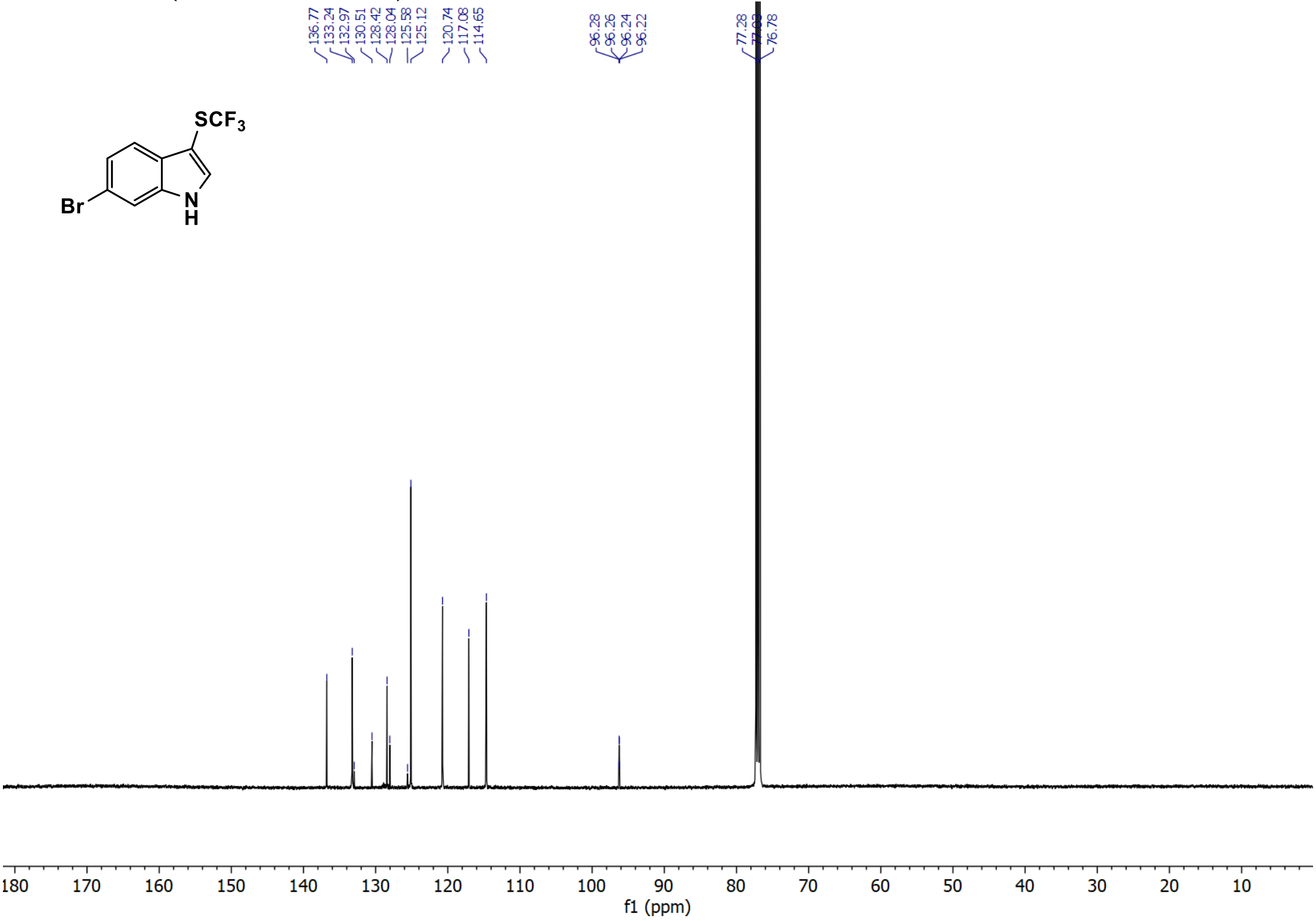

3c ^{19}F NMR (376 MHz, CDCl$_3$)

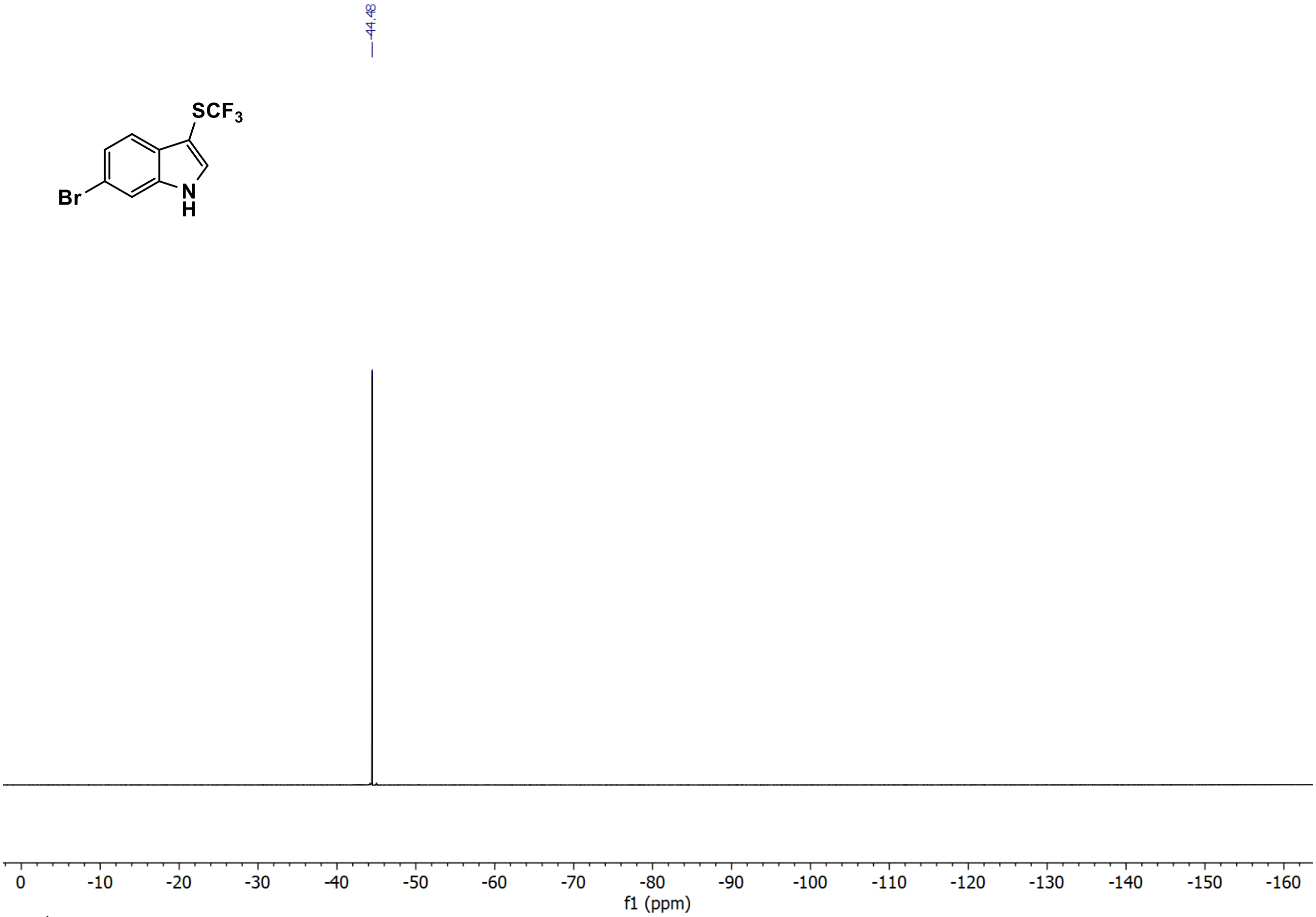

3d ^{1}H NMR (400 MHz, CDCl$_3$)

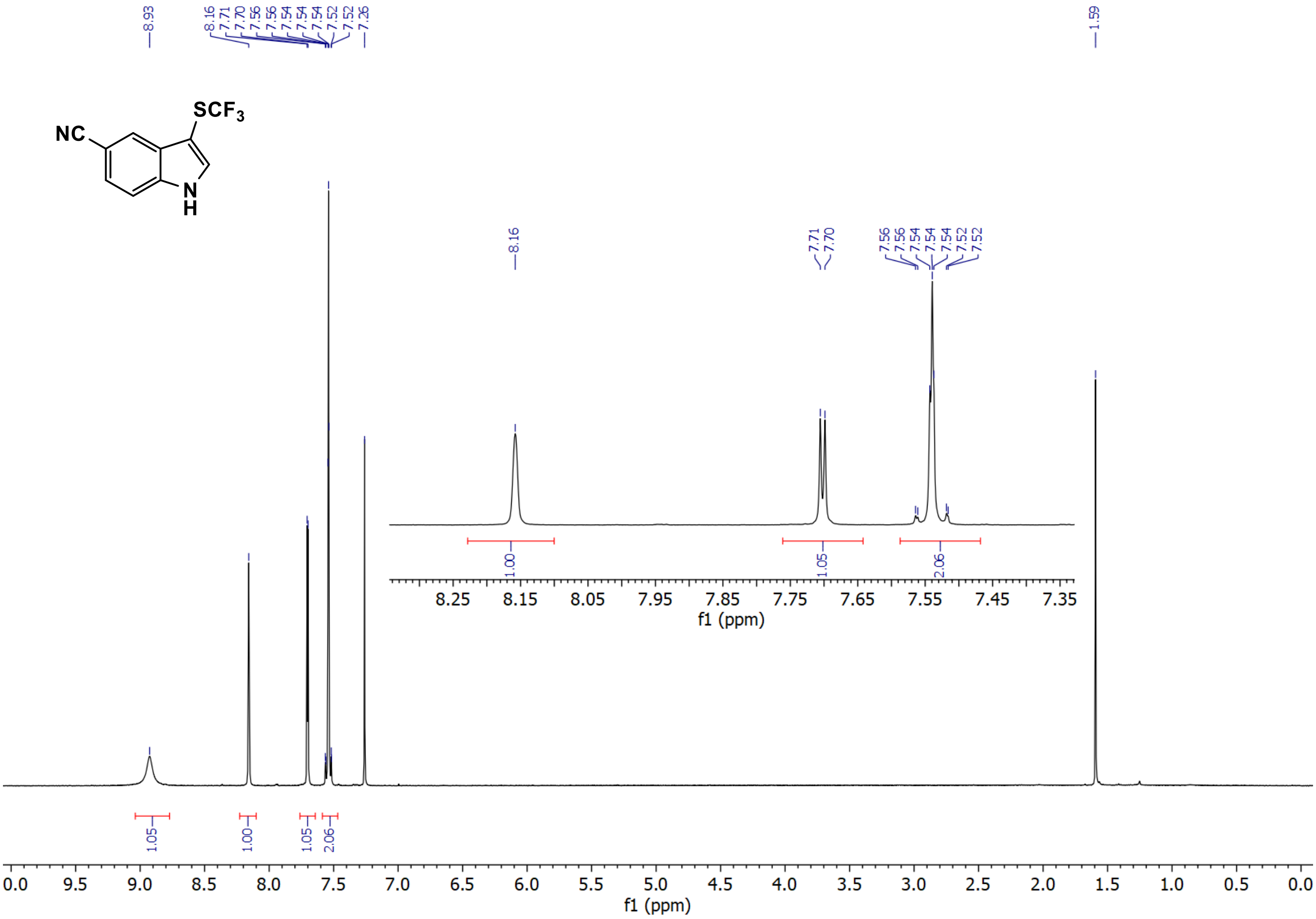

3d ^{13}C NMR (126 MHz, $CDCl_3$)

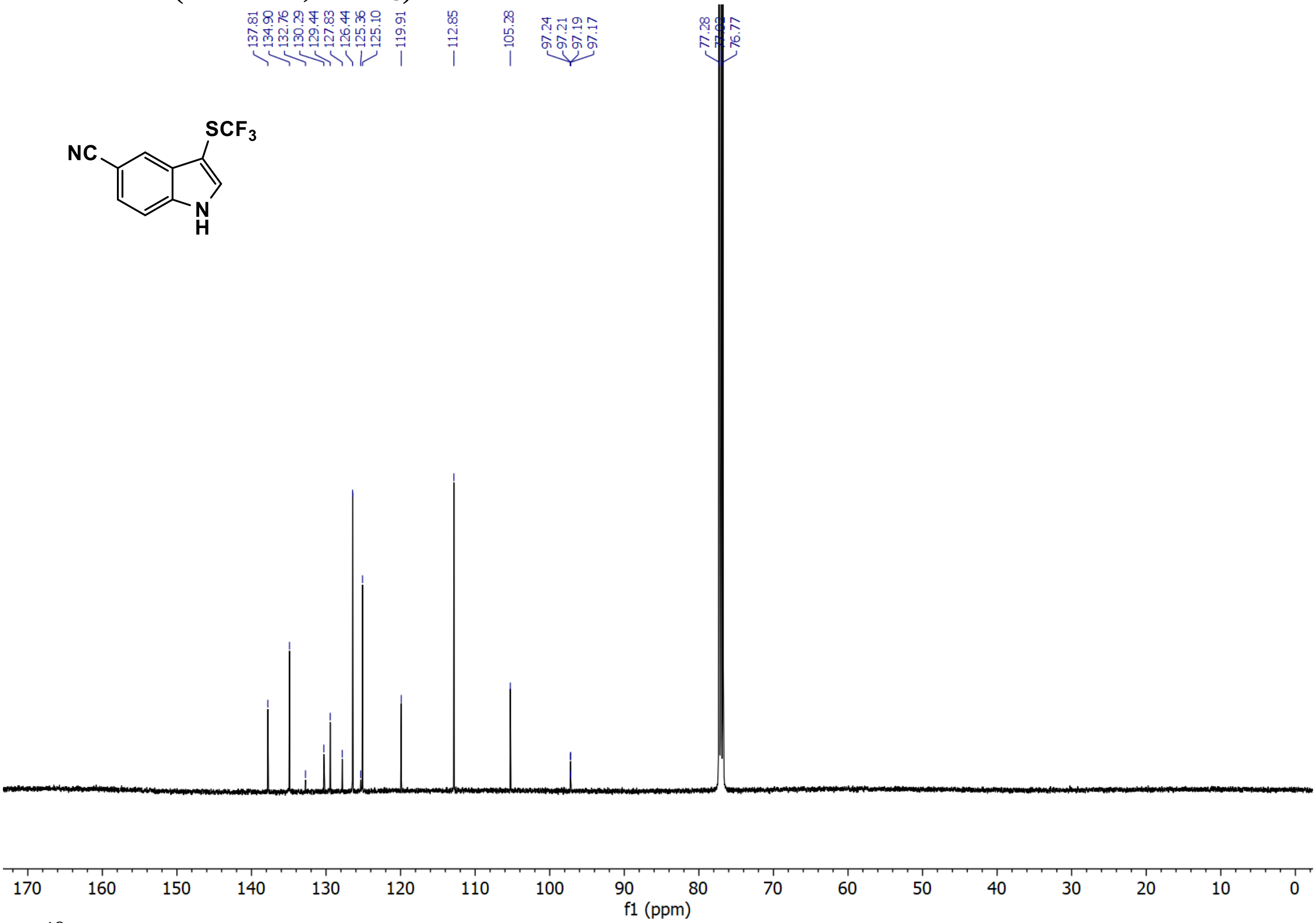

3d ^{19}F NMR (376 MHz, $CDCl_3$)

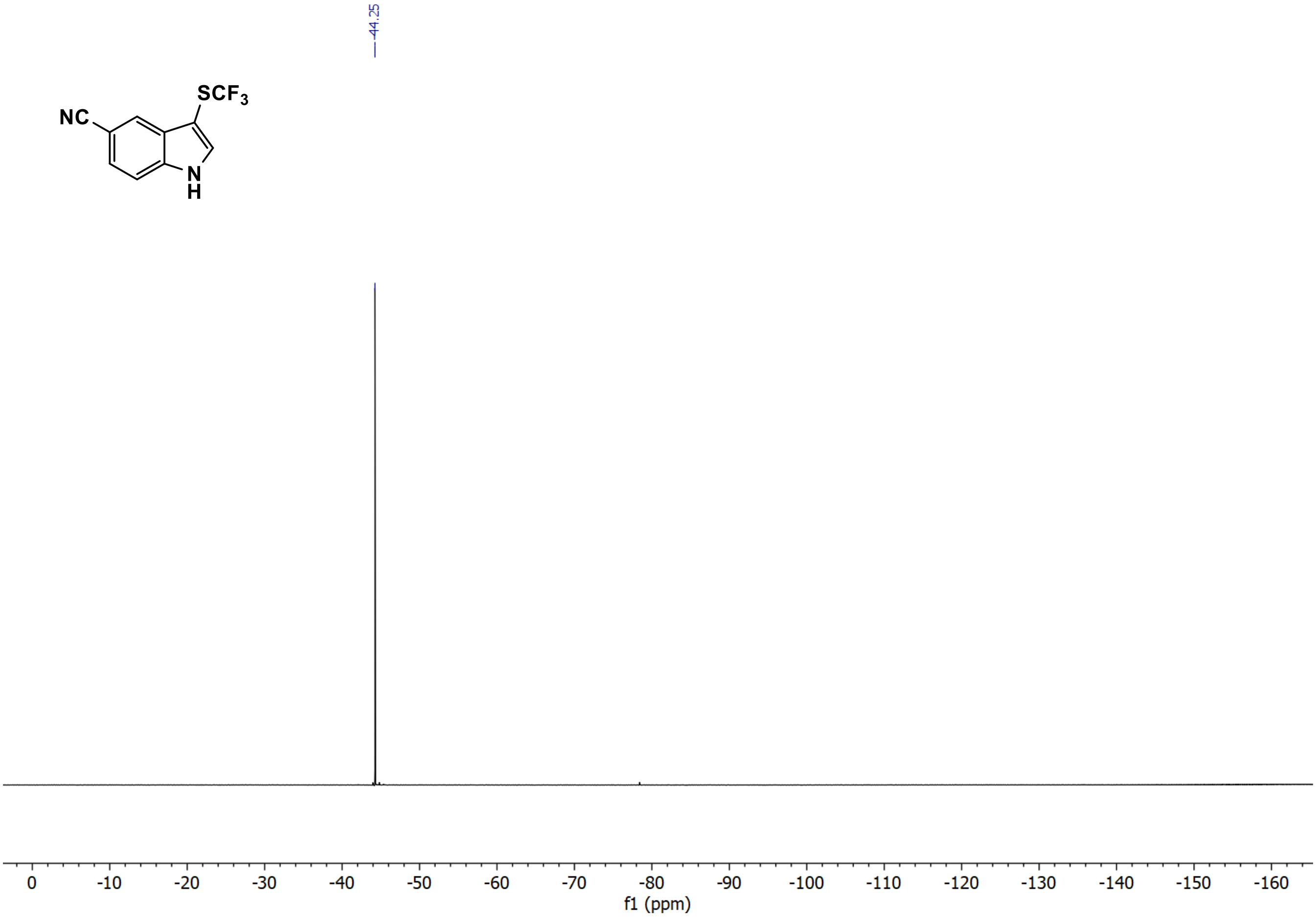

3e ^{1}H NMR (400 MHz, $CDCl_3$)

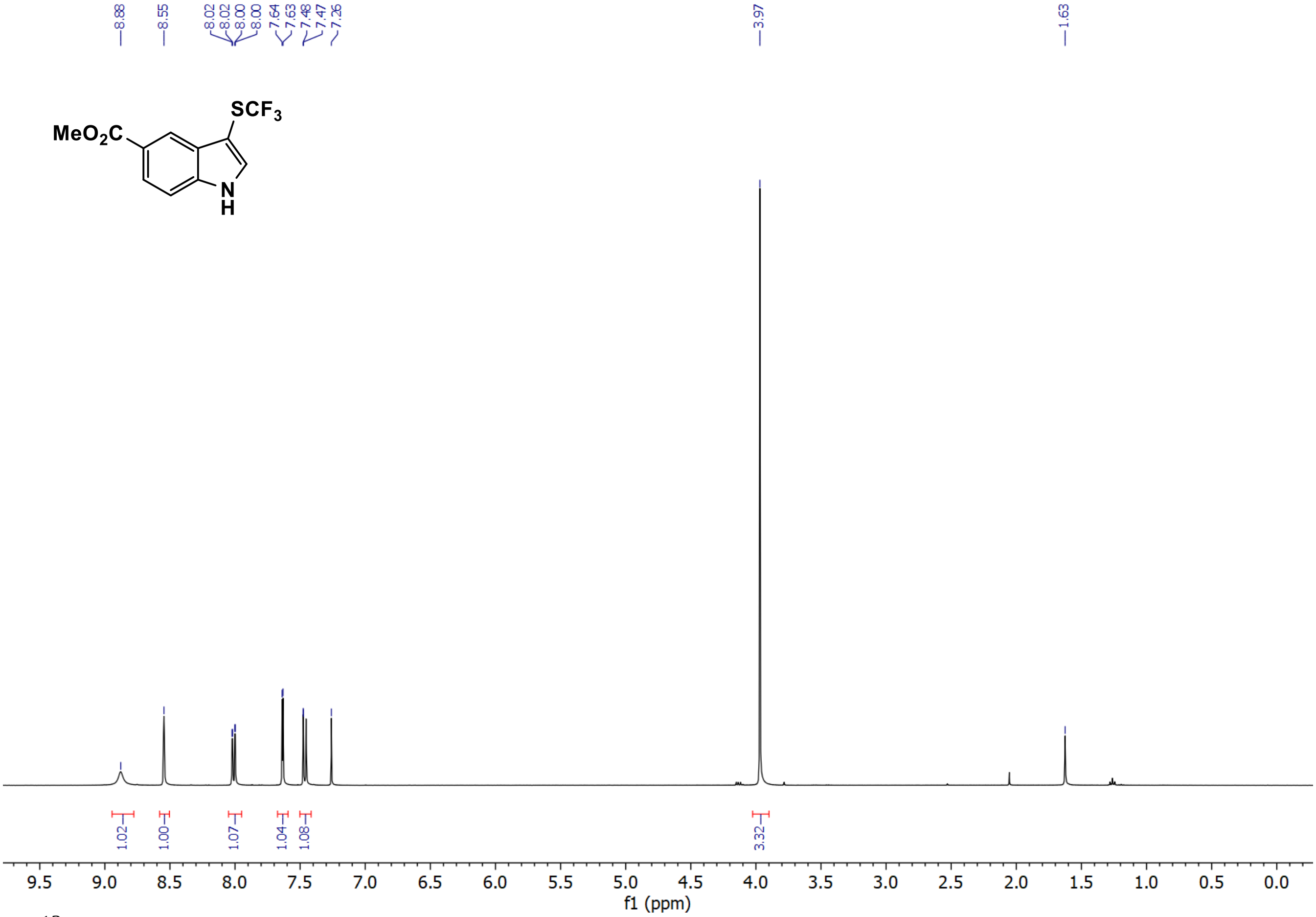

3e ^{13}C NMR (126 MHz, $CDCl_3$)

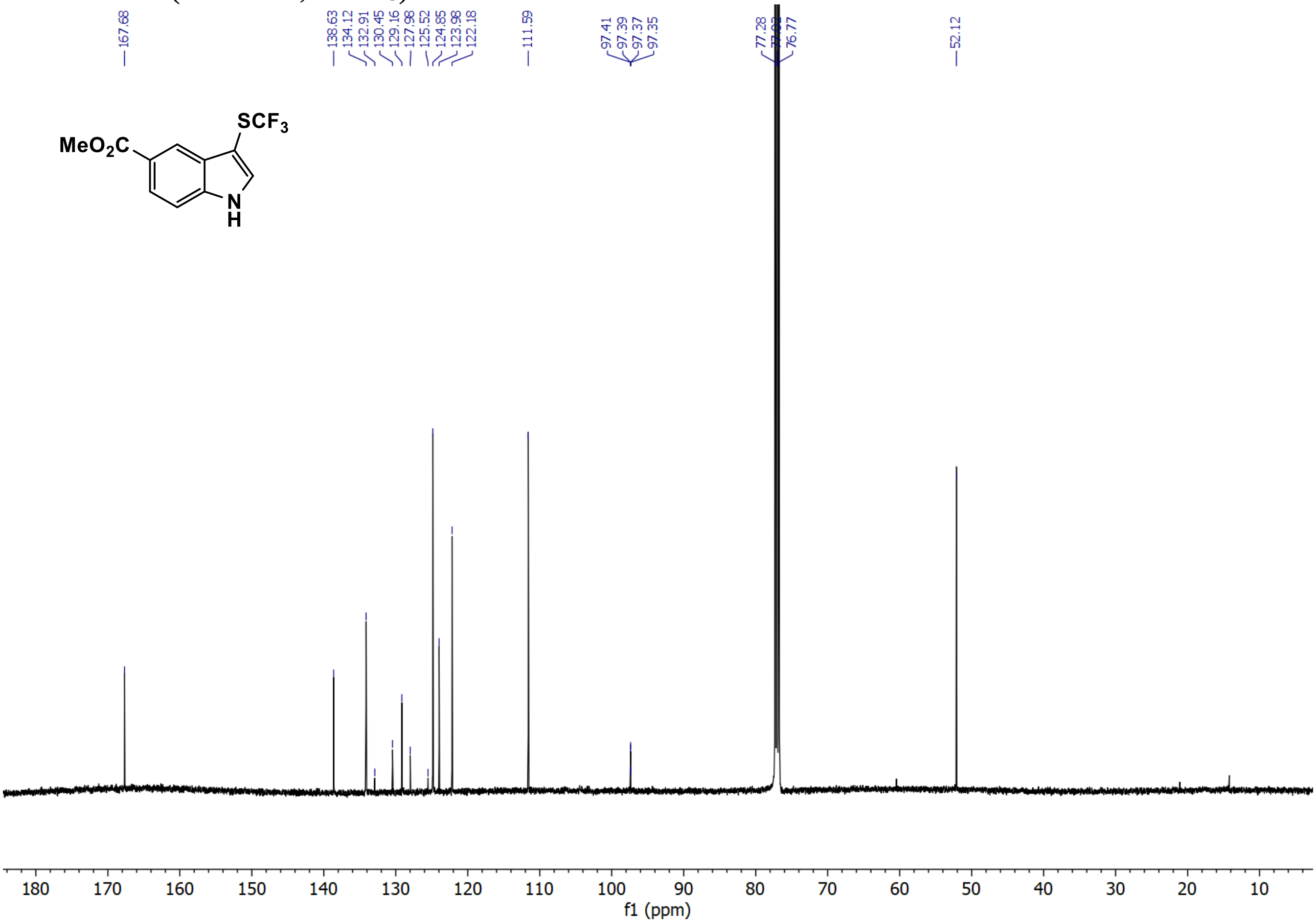

3e ^{19}F NMR (376 MHz, $CDCl_3$)

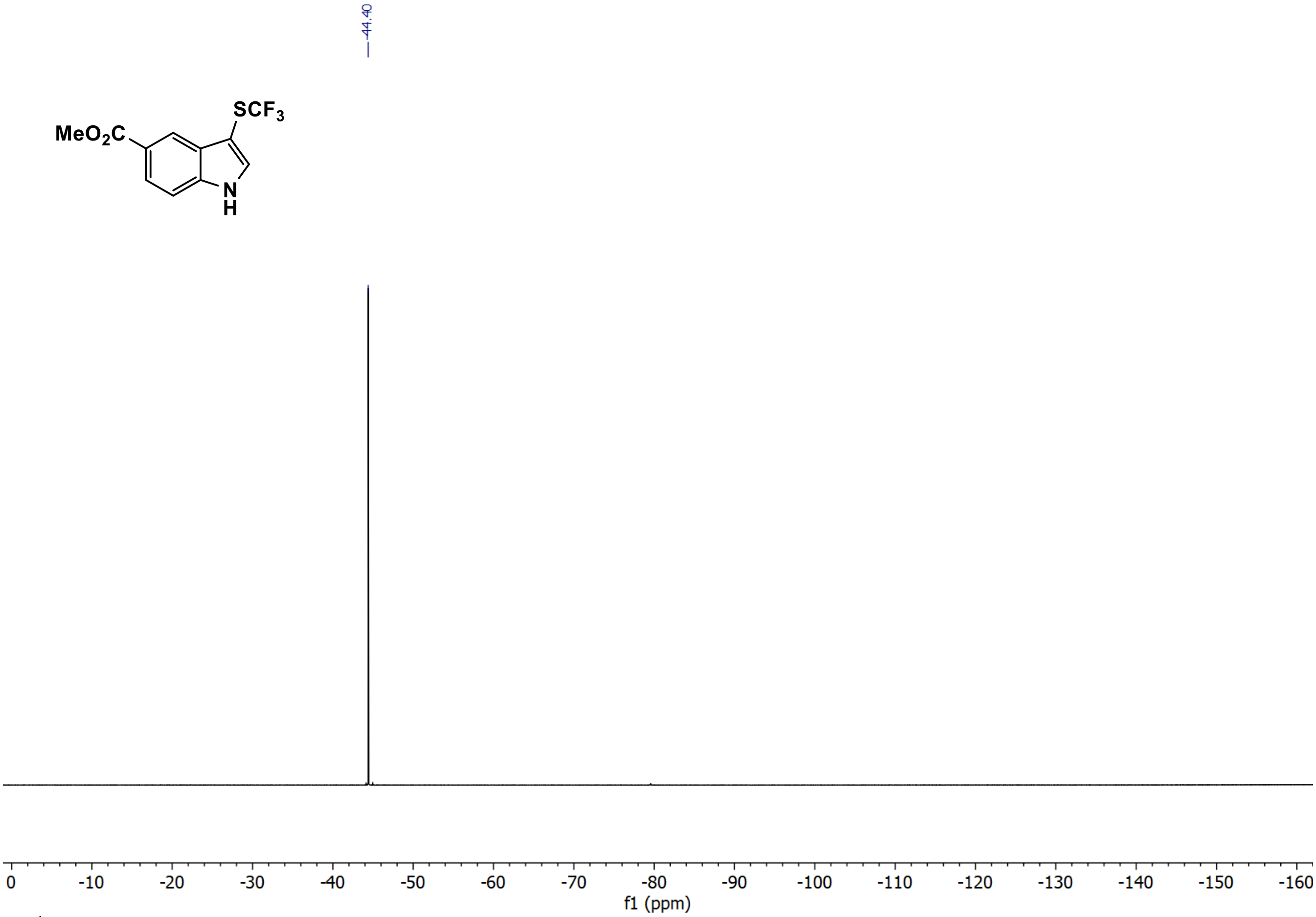

3f ^{1}H NMR (400 MHz, $CDCl_3$)

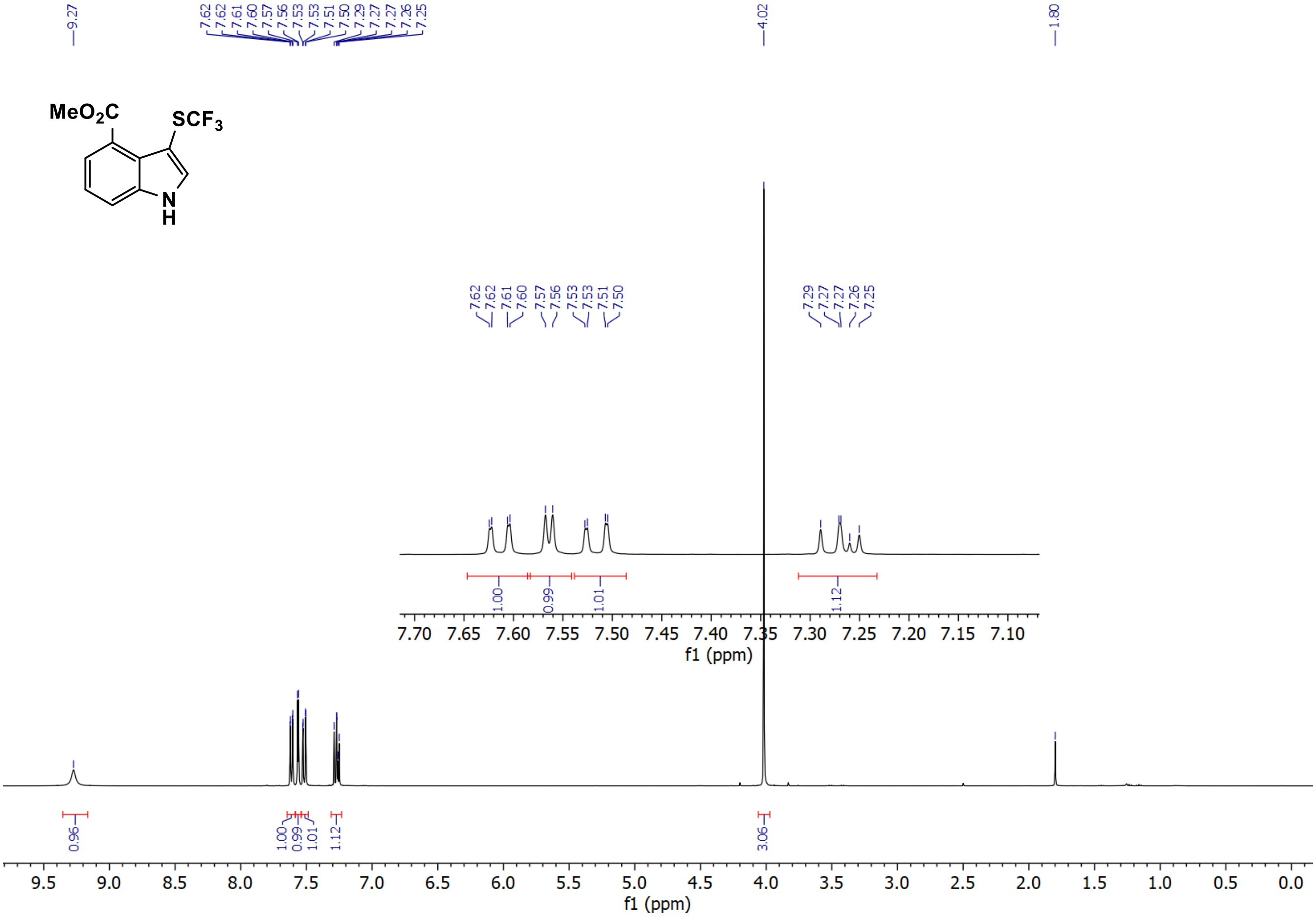

3f ^{13}C NMR (101 MHz, $CDCl_3$)

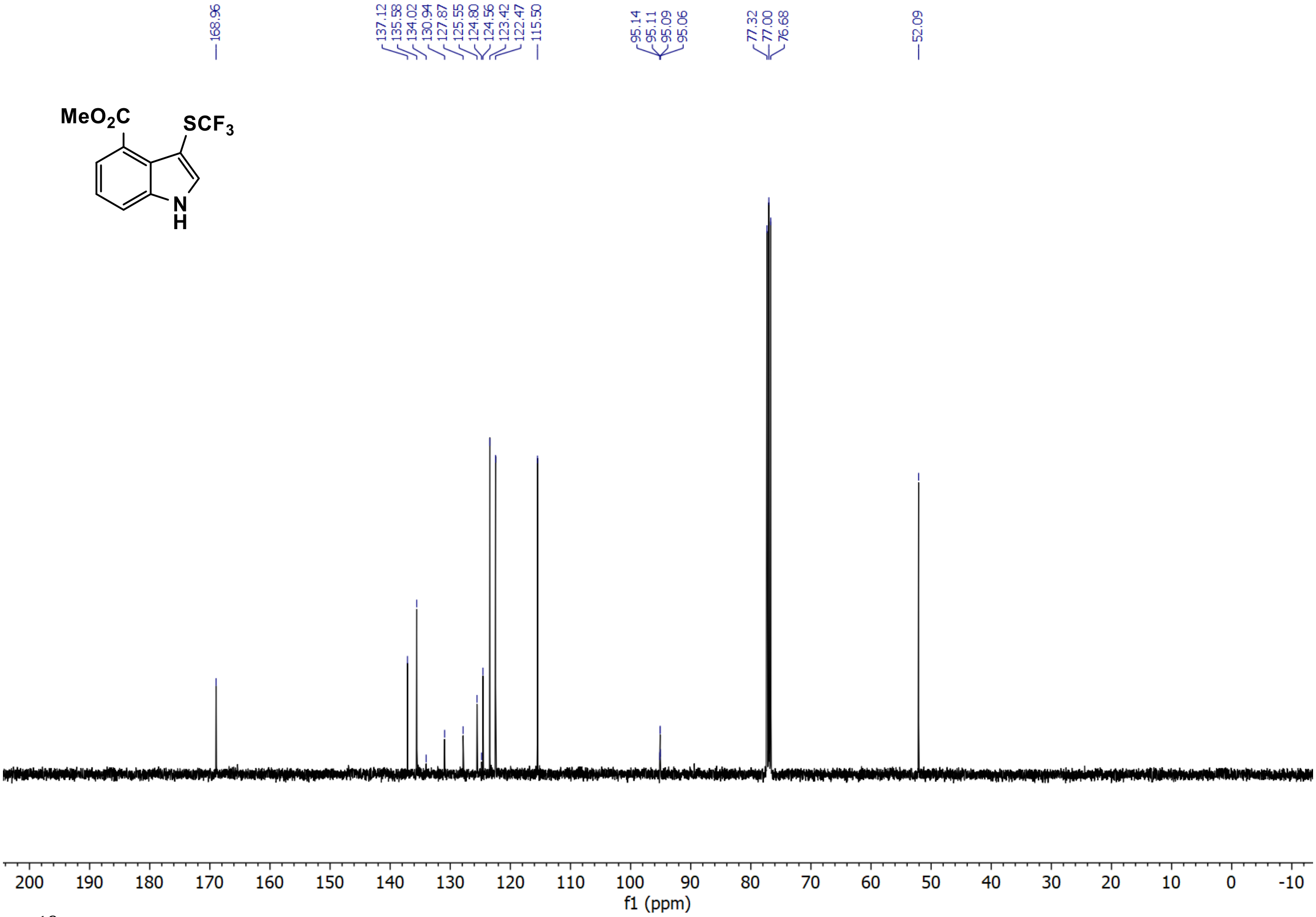

3f ^{19}F NMR (376 MHz, $CDCl_3$)

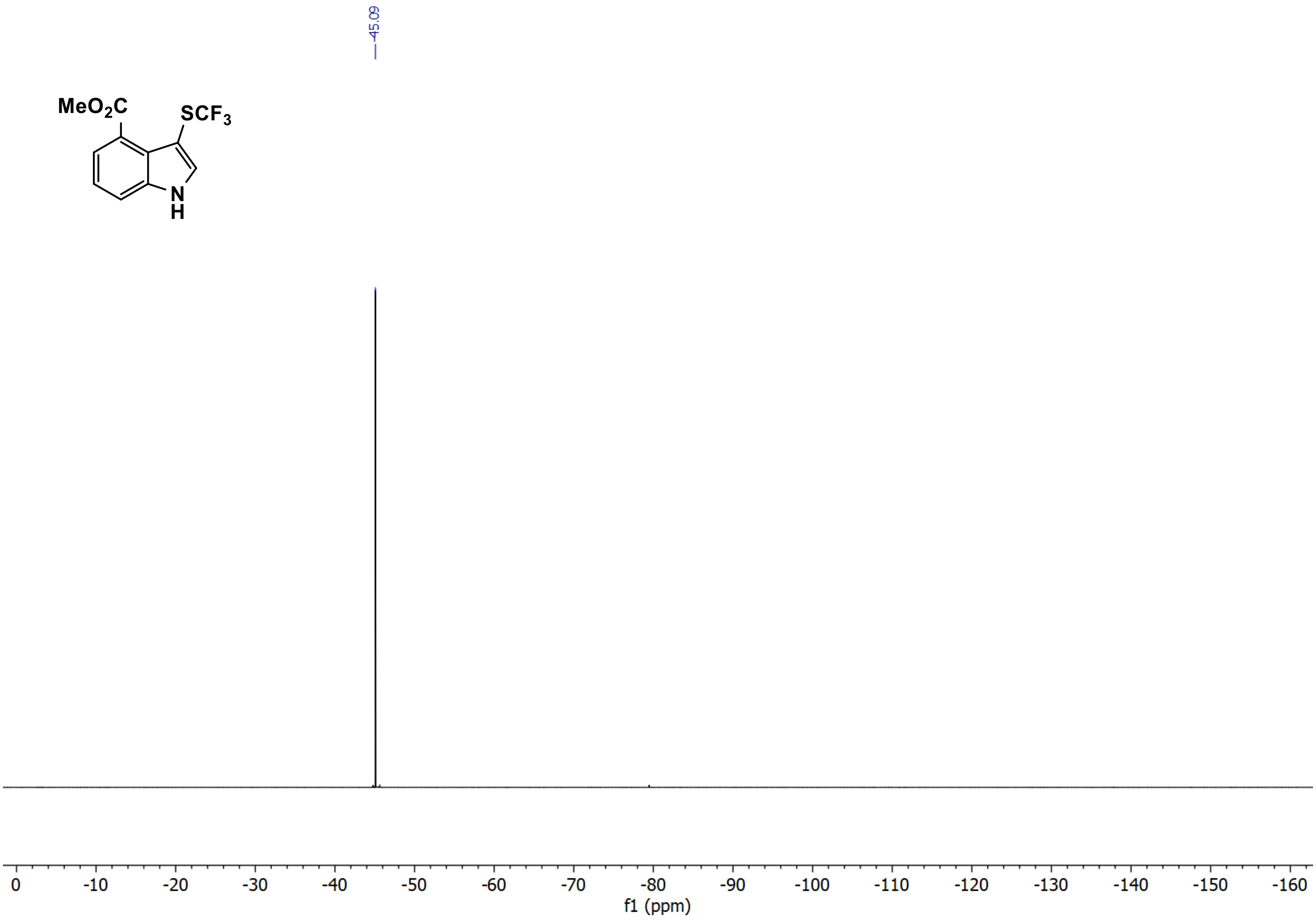

3g ^{1}H NMR (400 MHz, $CDCl_3$)

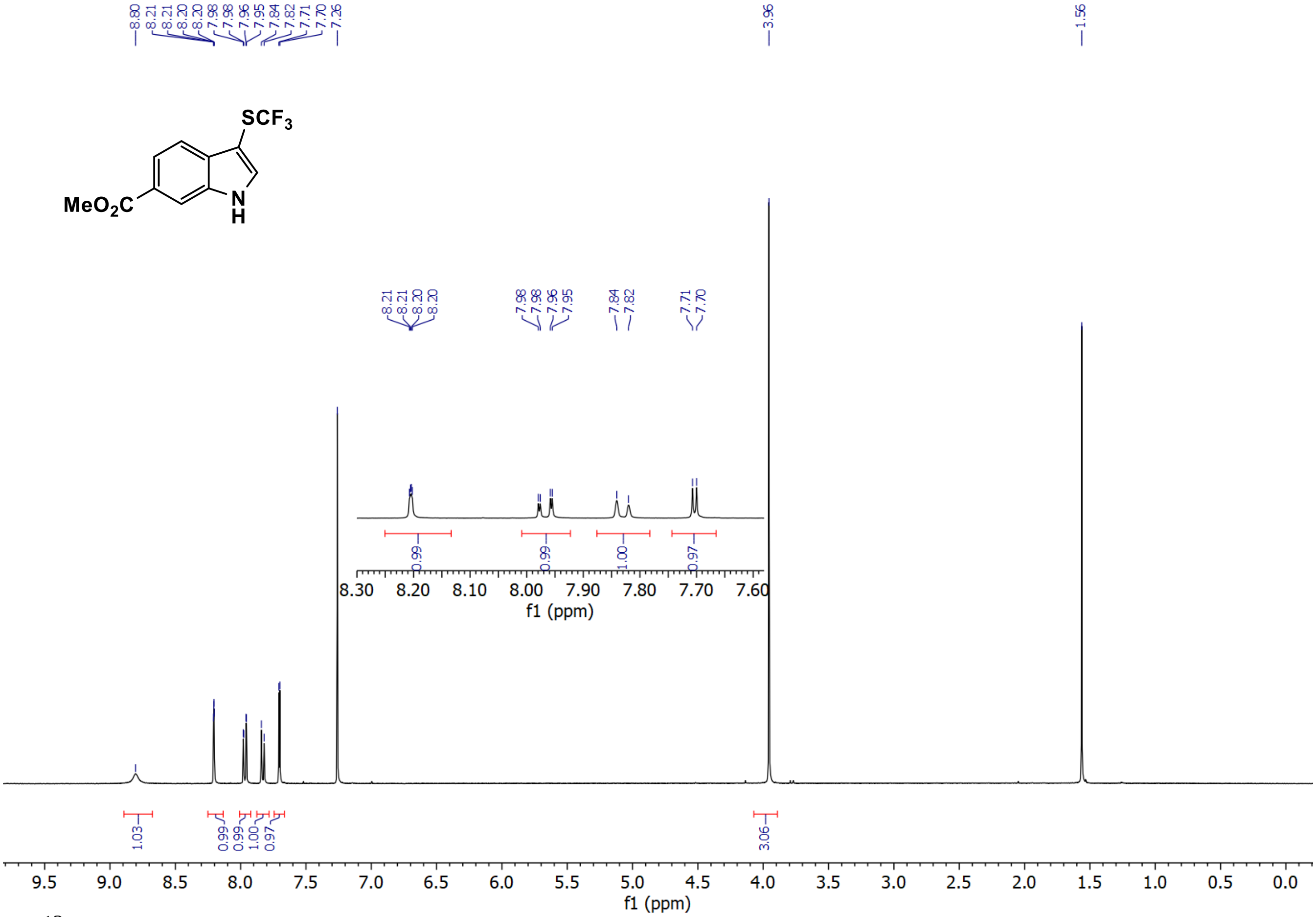

3g ^{13}C NMR (126 MHz, $CDCl_3$)

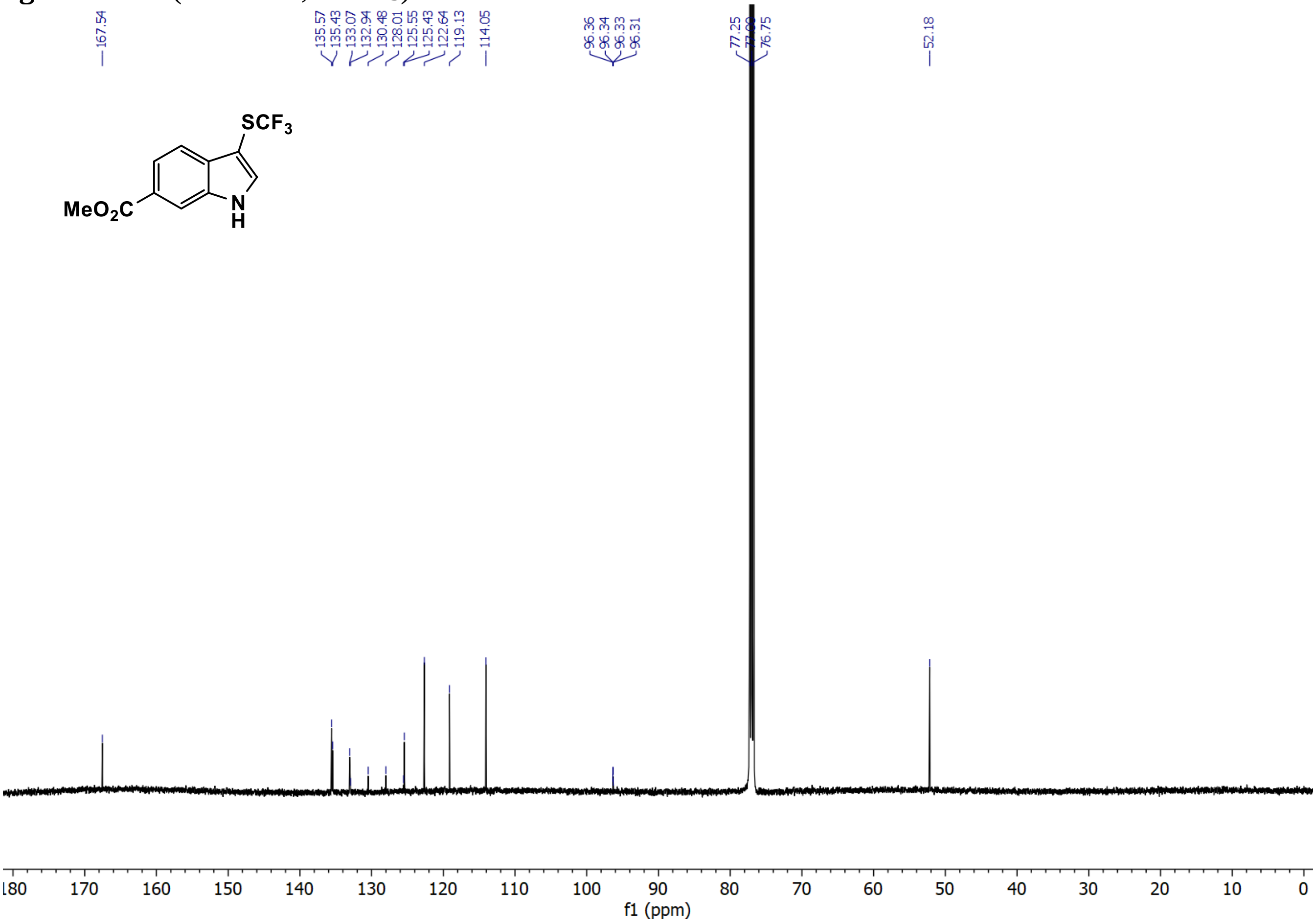

3g ^{19}F NMR (376 MHz, $CDCl_3$)

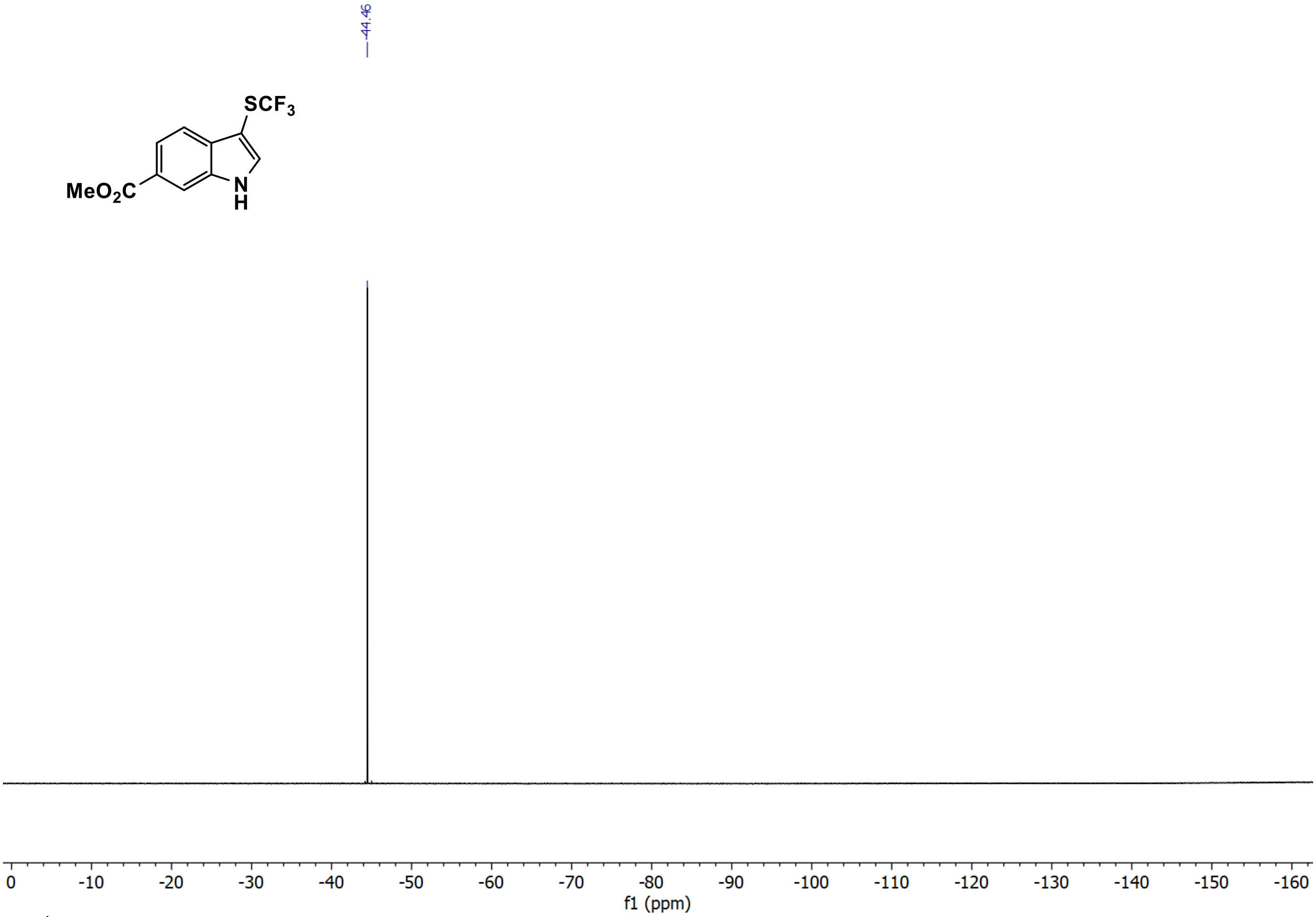

3h ^{1}H NMR (400 MHz, $CDCl_3$)

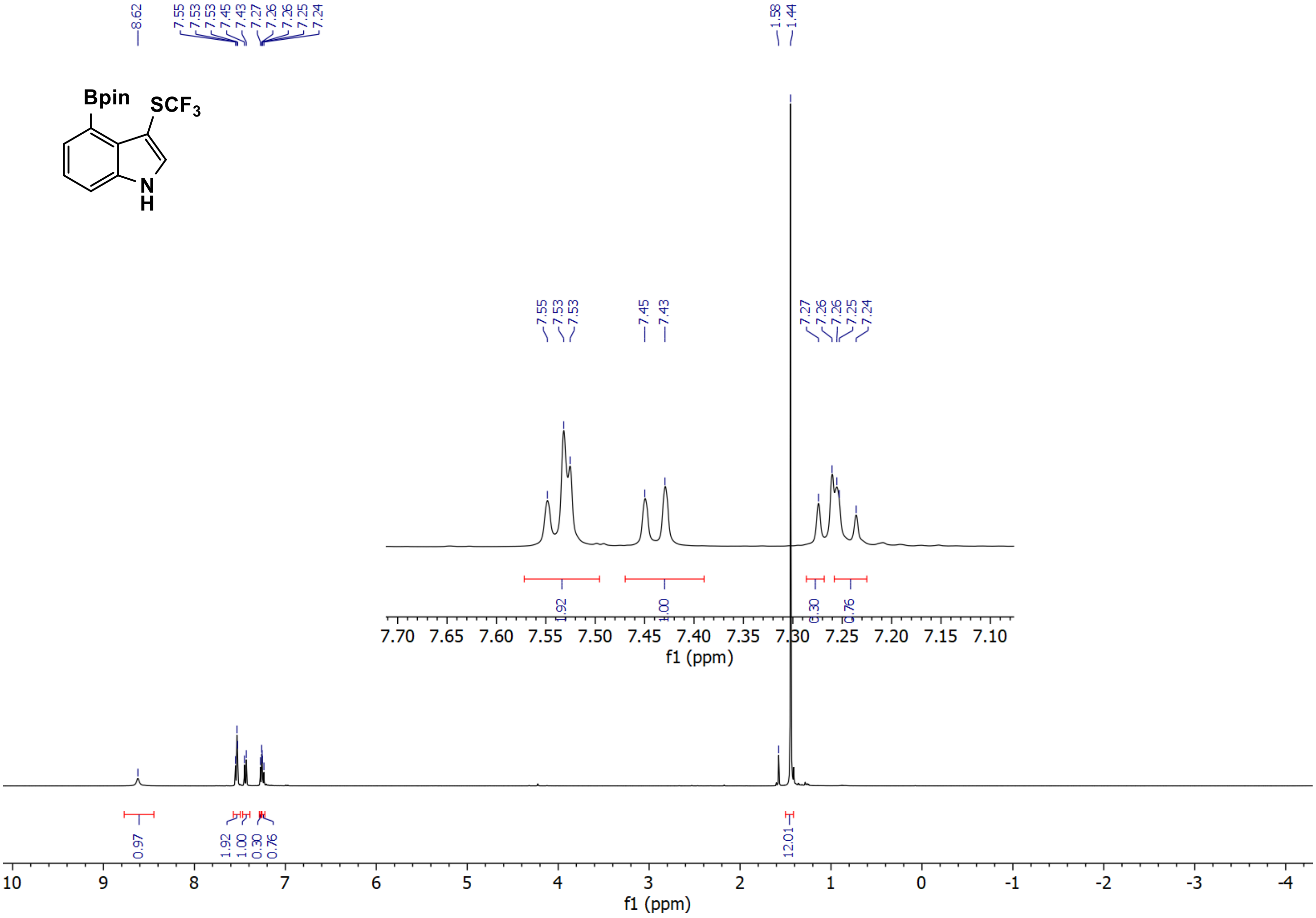

3h ^{13}C NMR (101 MHz, $CDCl_3$)

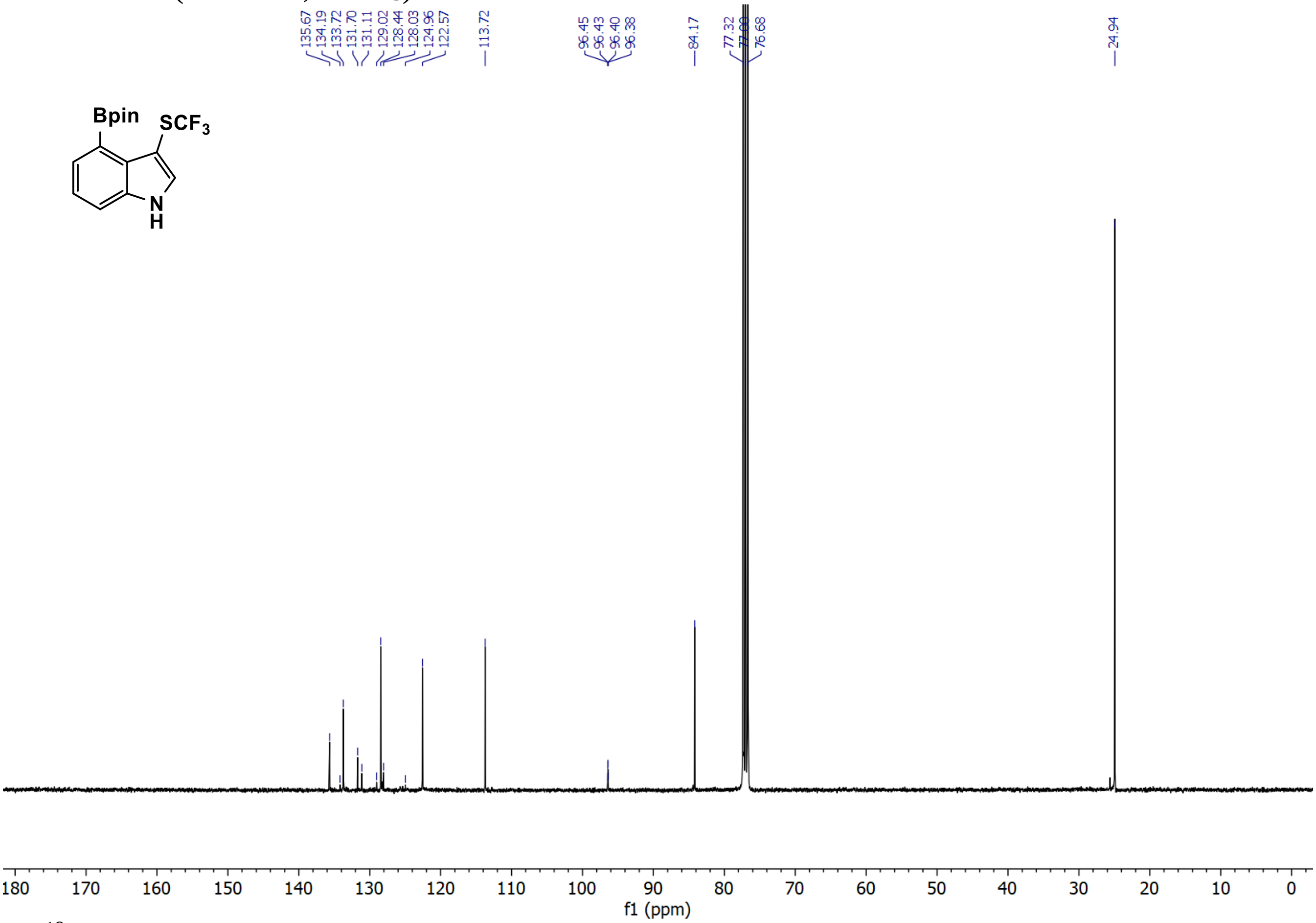

3h ^{19}F NMR (376 MHz, $CDCl_3$)

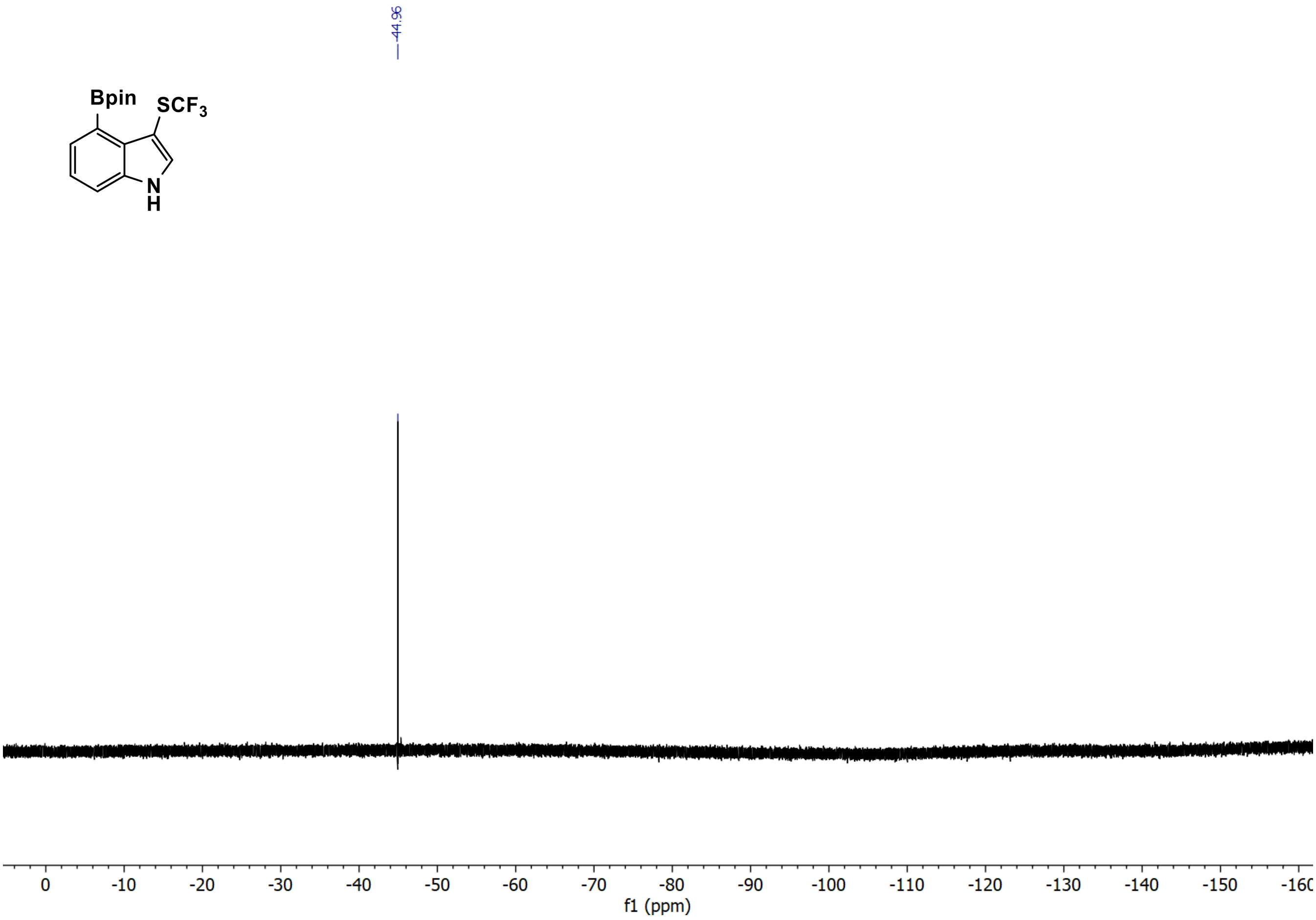

3i ^{1}H NMR (400 MHz, $CDCl_3$)

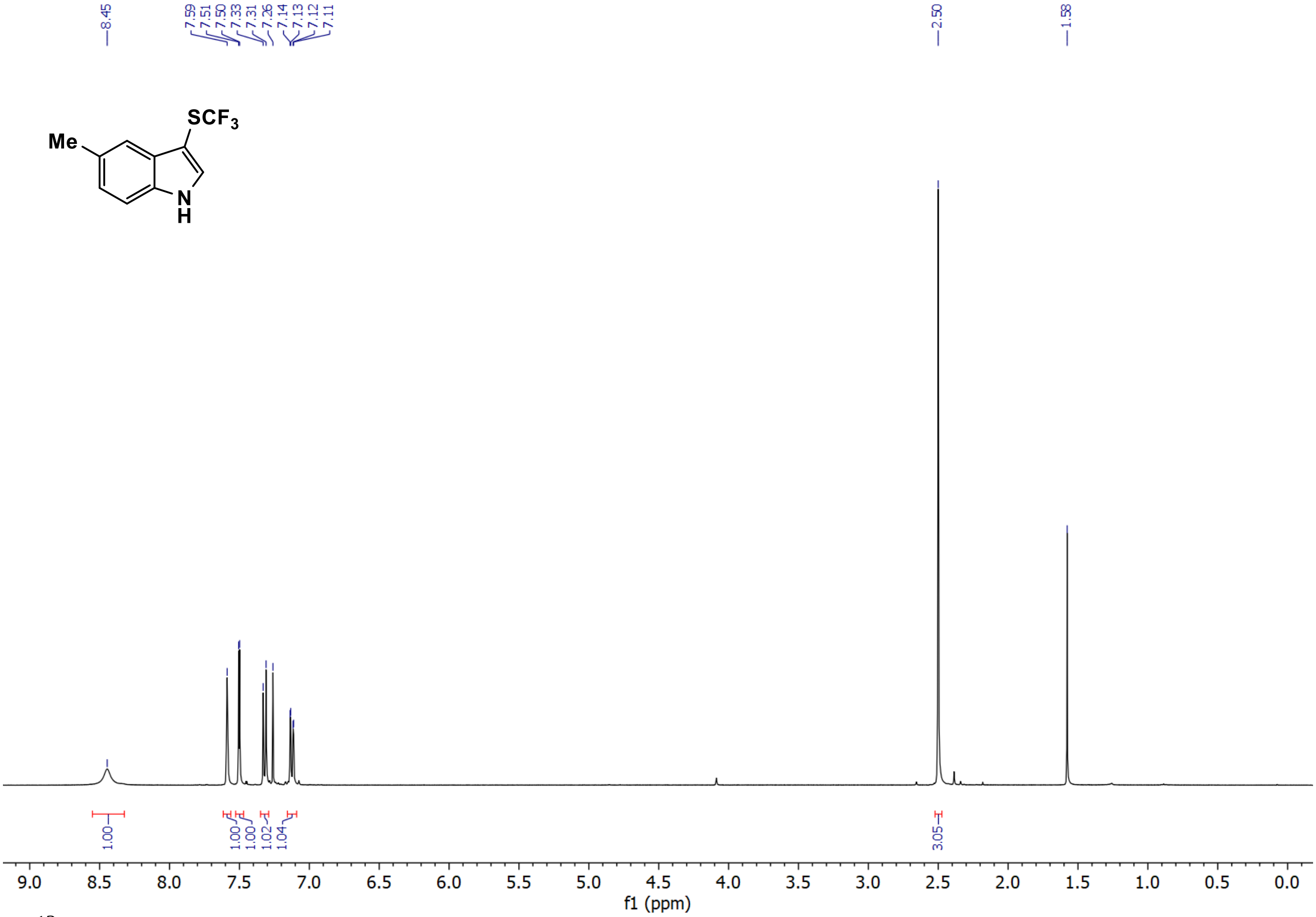

3i ^{13}C NMR (126 MHz, $CDCl_3$)

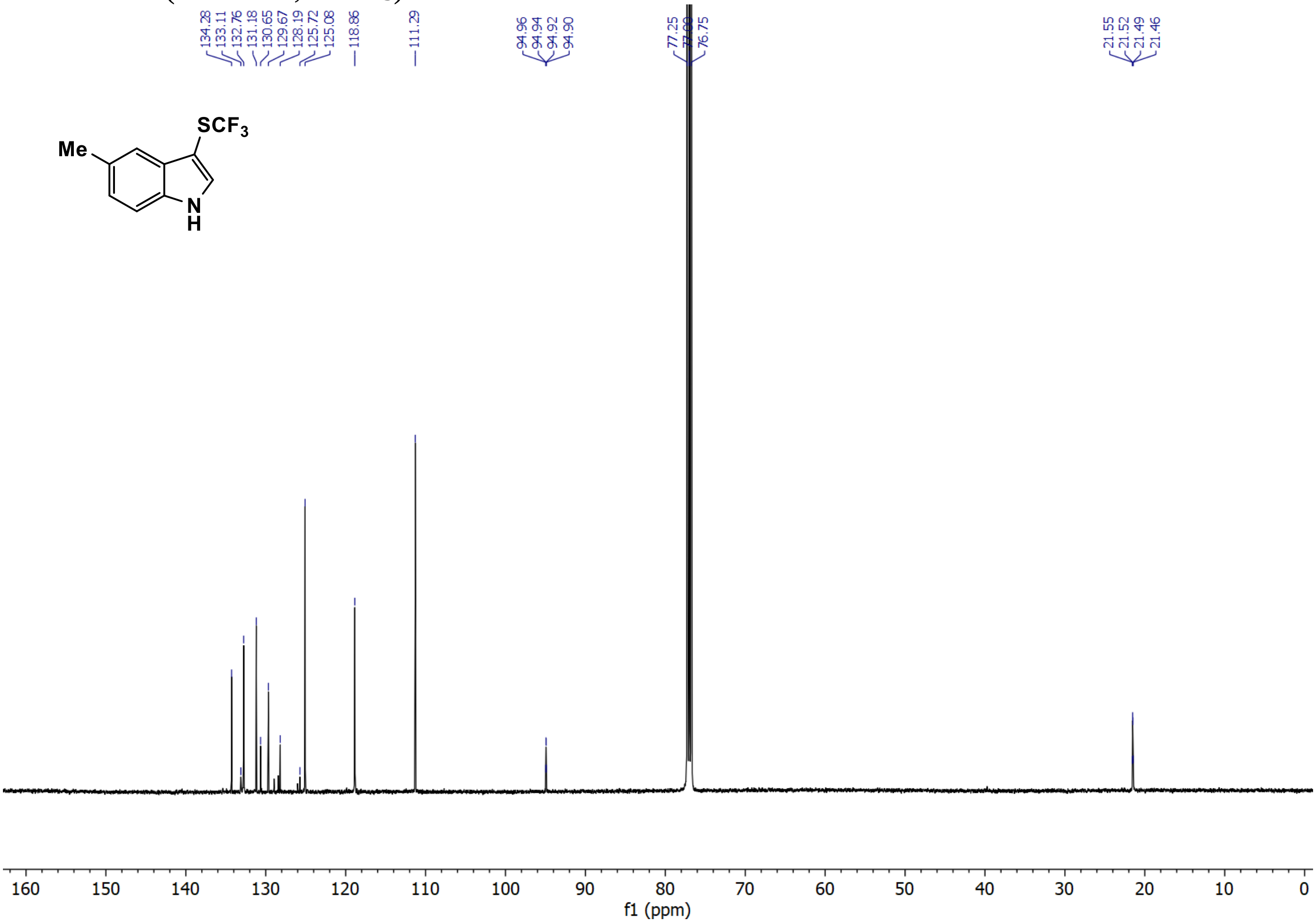

3i ^{19}F NMR (376 MHz, $CDCl_3$)

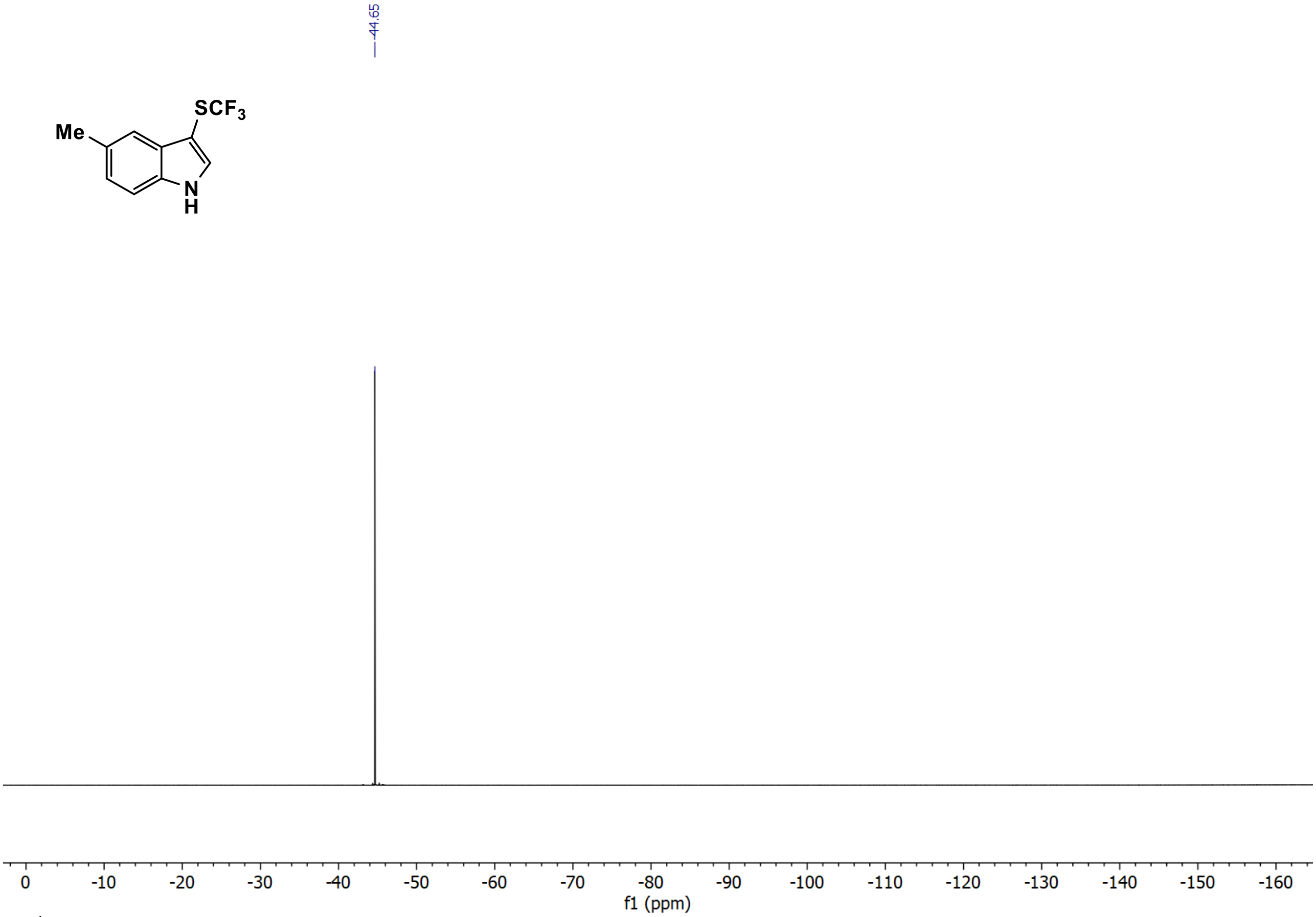

3j ^{1}H NMR (400 MHz, $CDCl_3$)

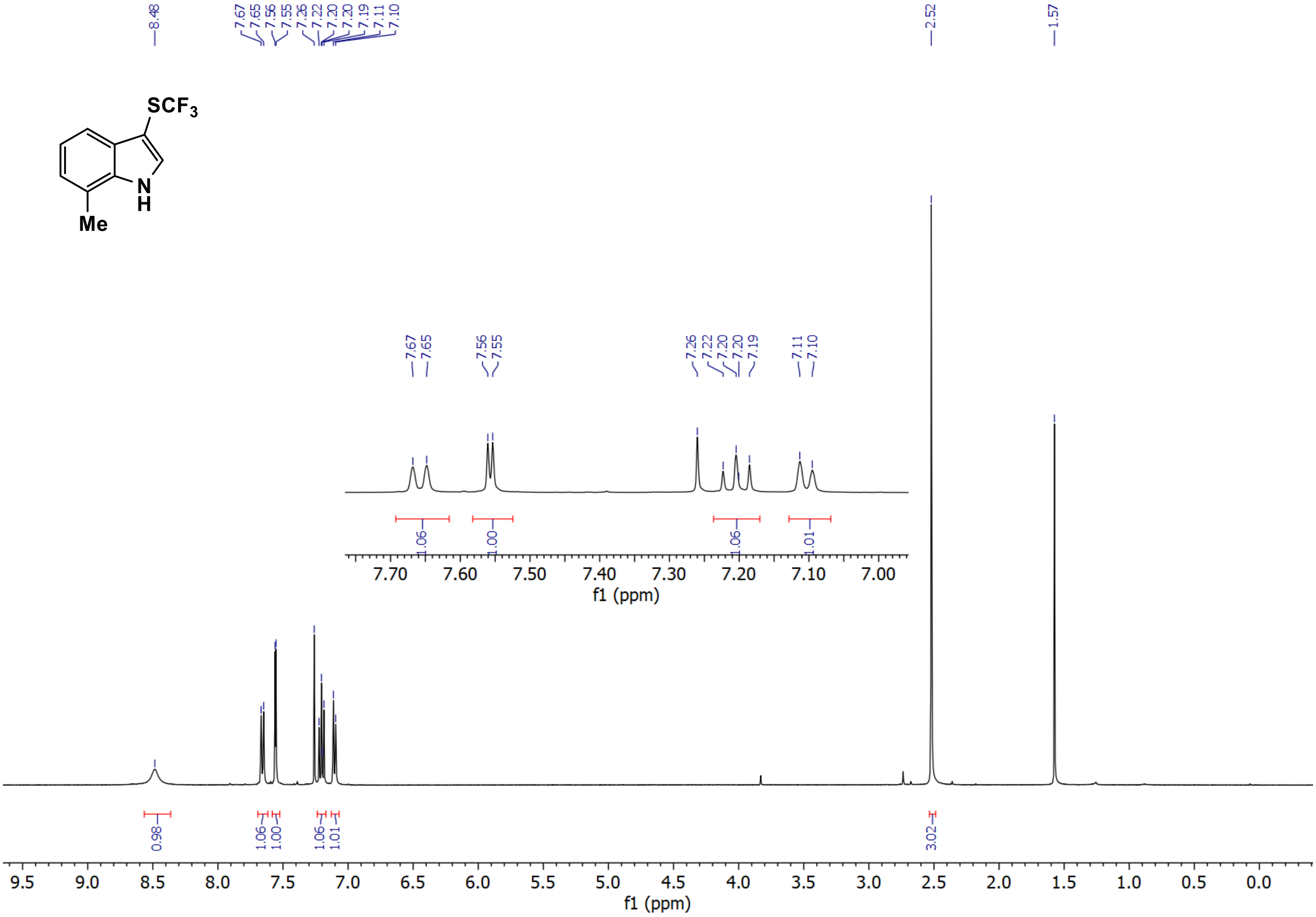

3j ^{13}C NMR (126 MHz, $CDCl_3$)

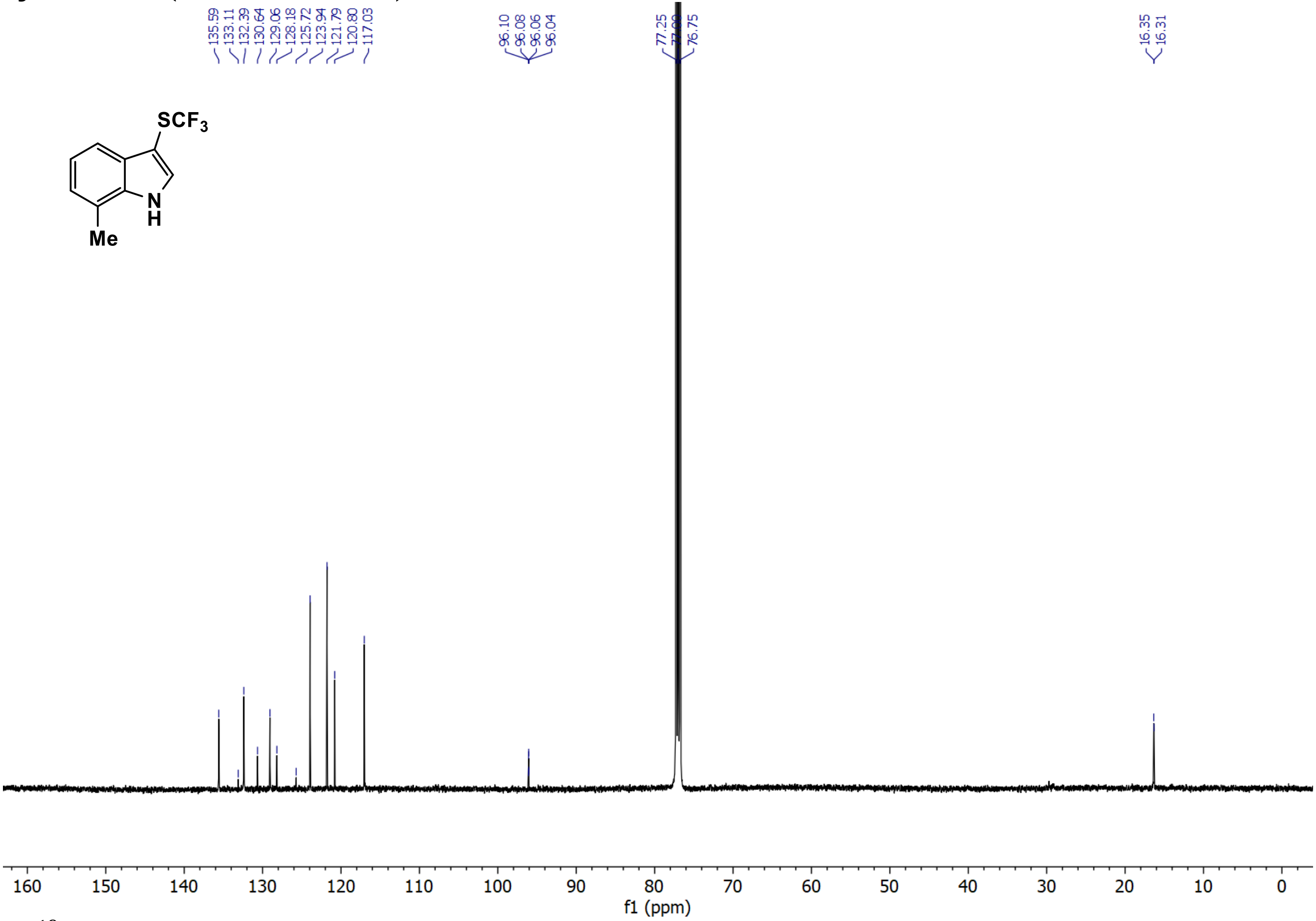

3j ^{19}F NMR (376 MHz, $CDCl_3$)

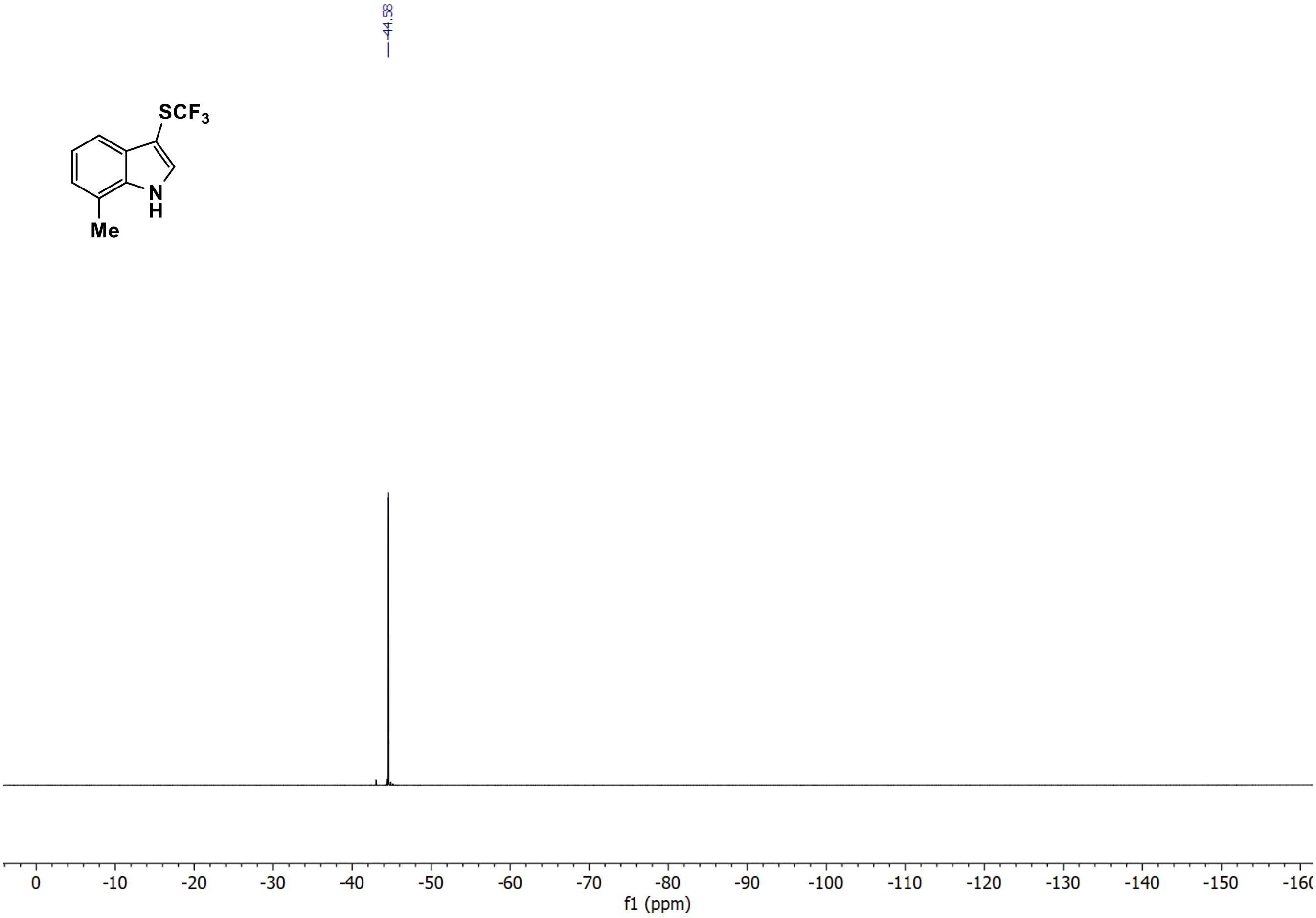

3k ^{1}H NMR (400 MHz, $CDCl_3$)

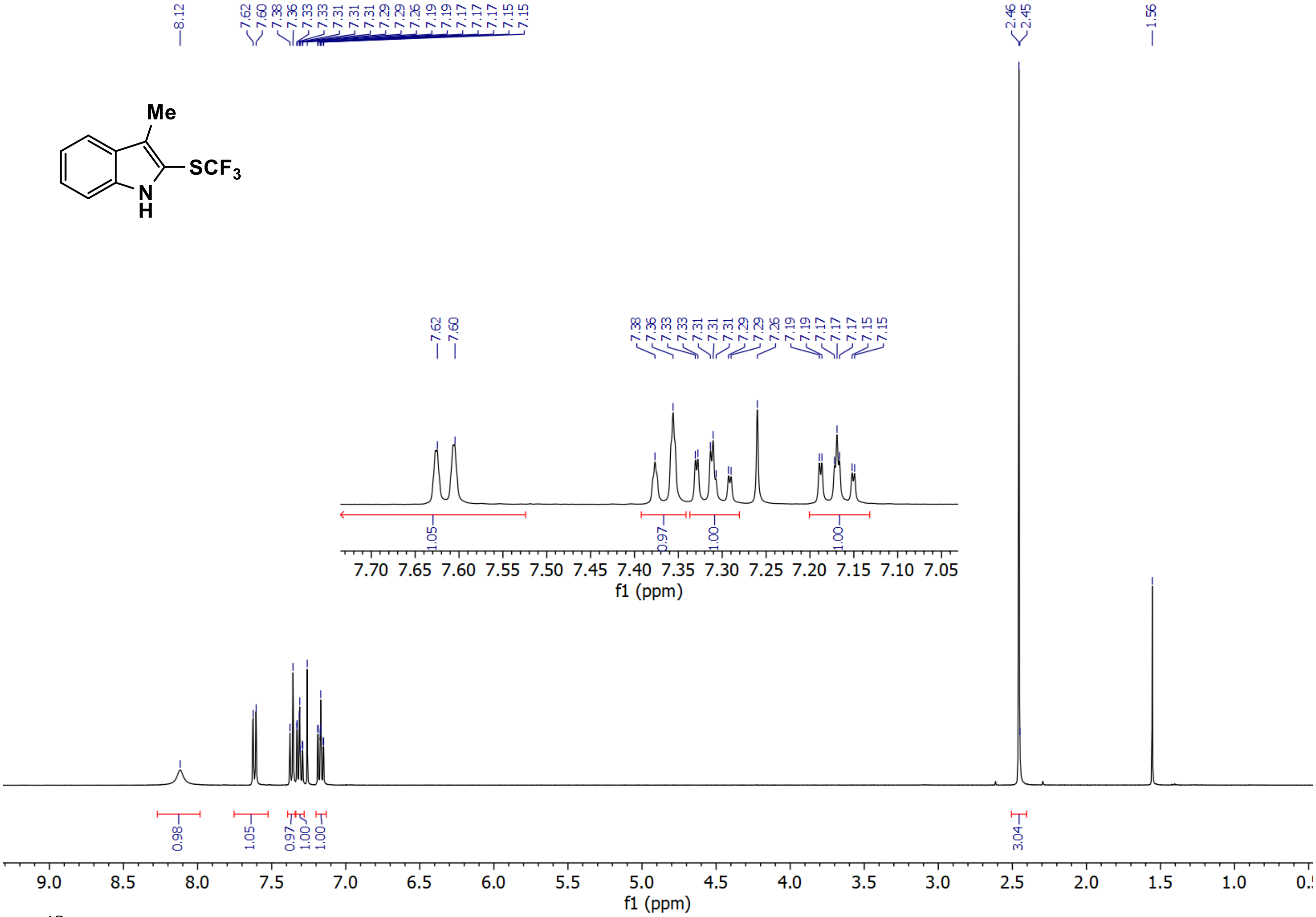

3k ^{13}C NMR (101 MHz, $CDCl_3$)

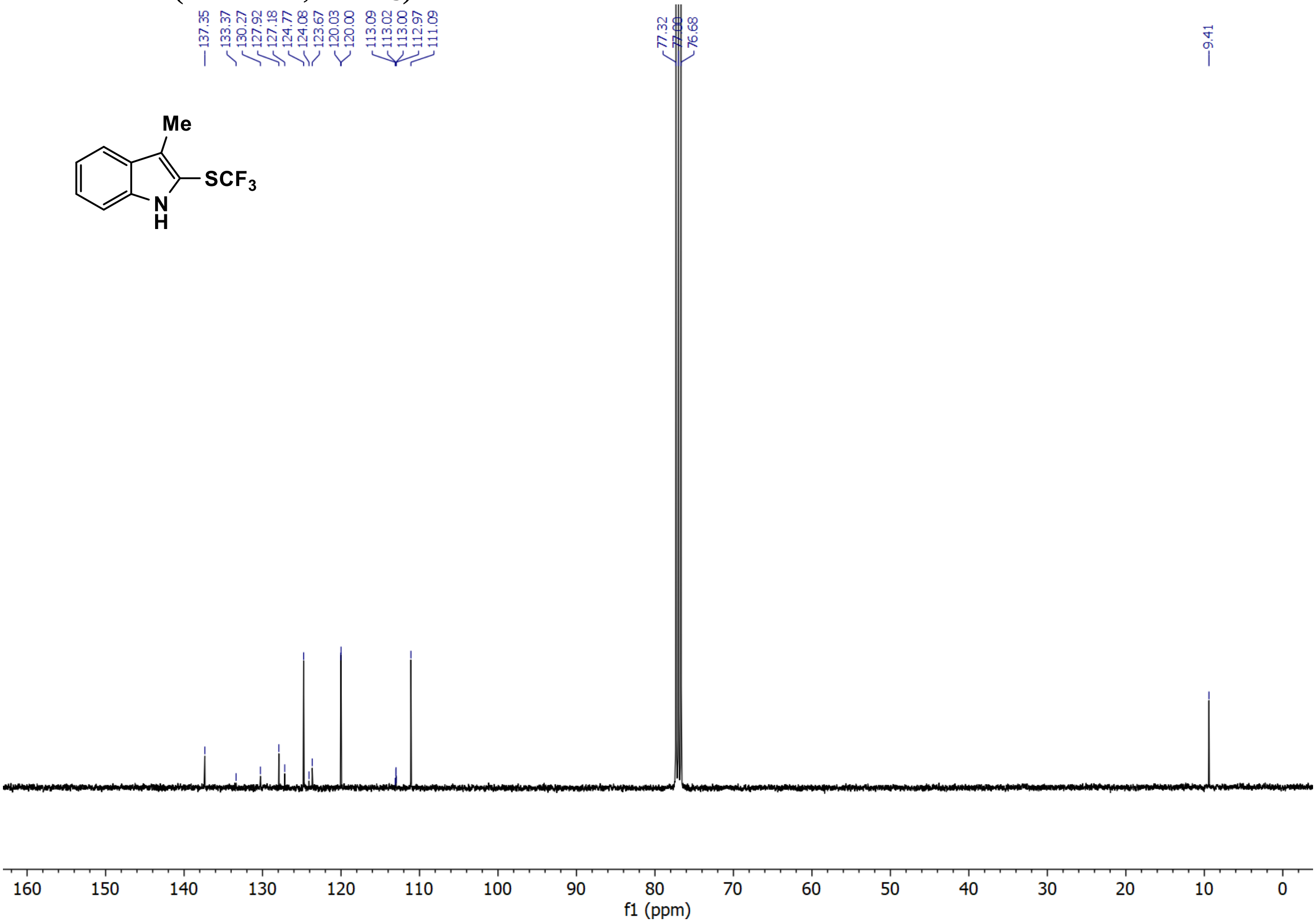

3k ^{19}F NMR (376 MHz, $CDCl_3$)

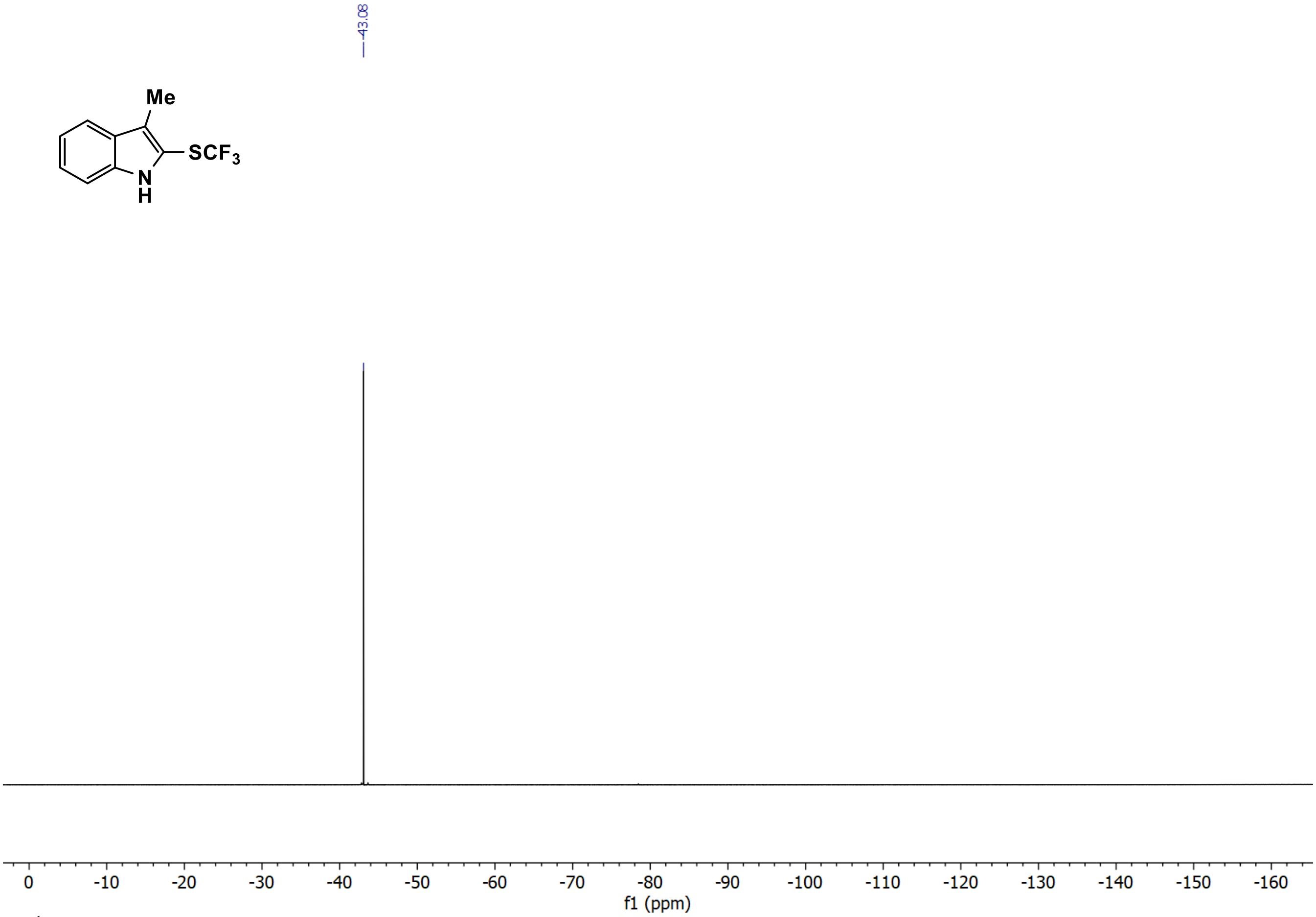

3l ^{1}H NMR (400 MHz, $CDCl_3$)

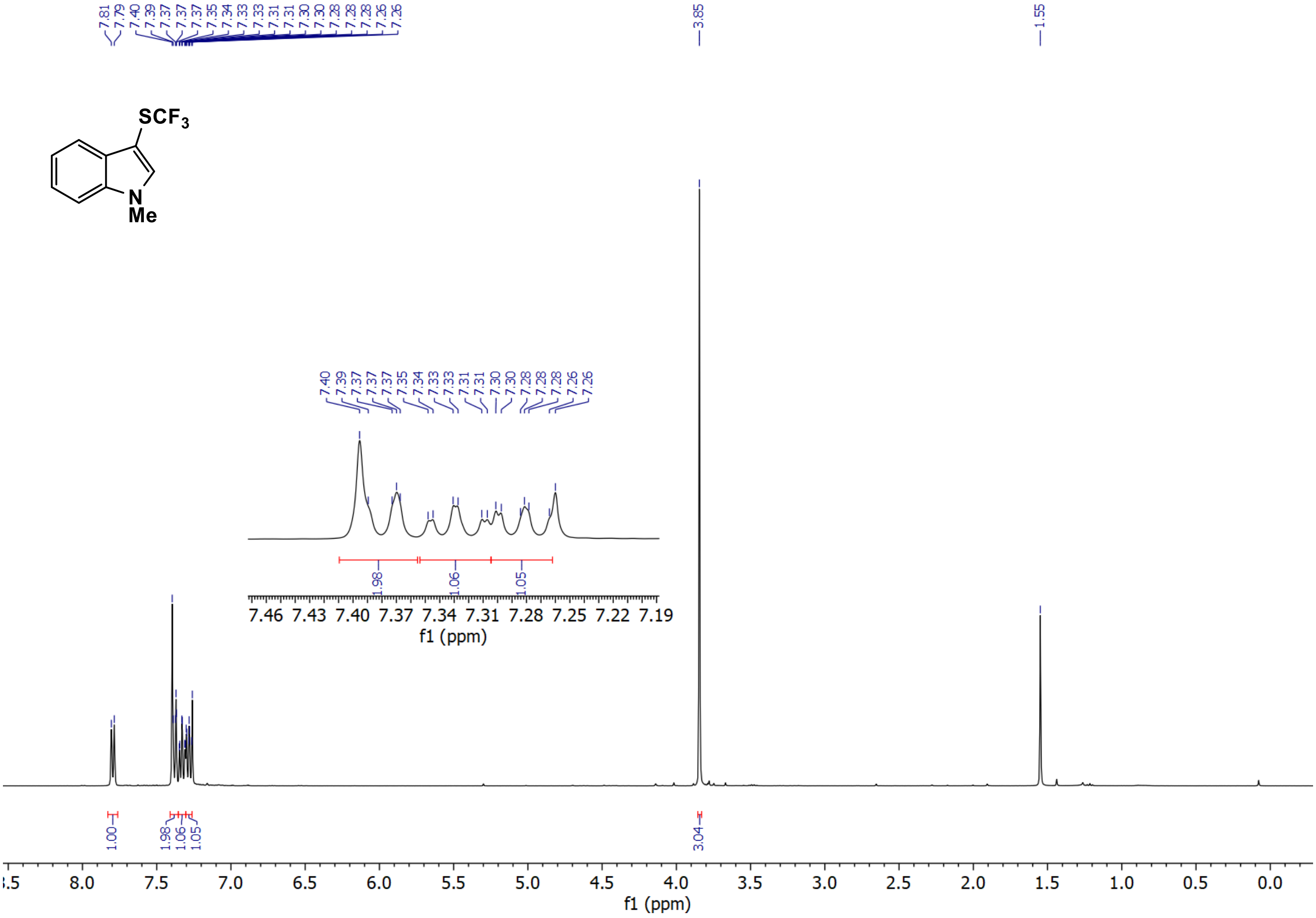

3l ^{13}C NMR (101 MHz, $CDCl_3$)

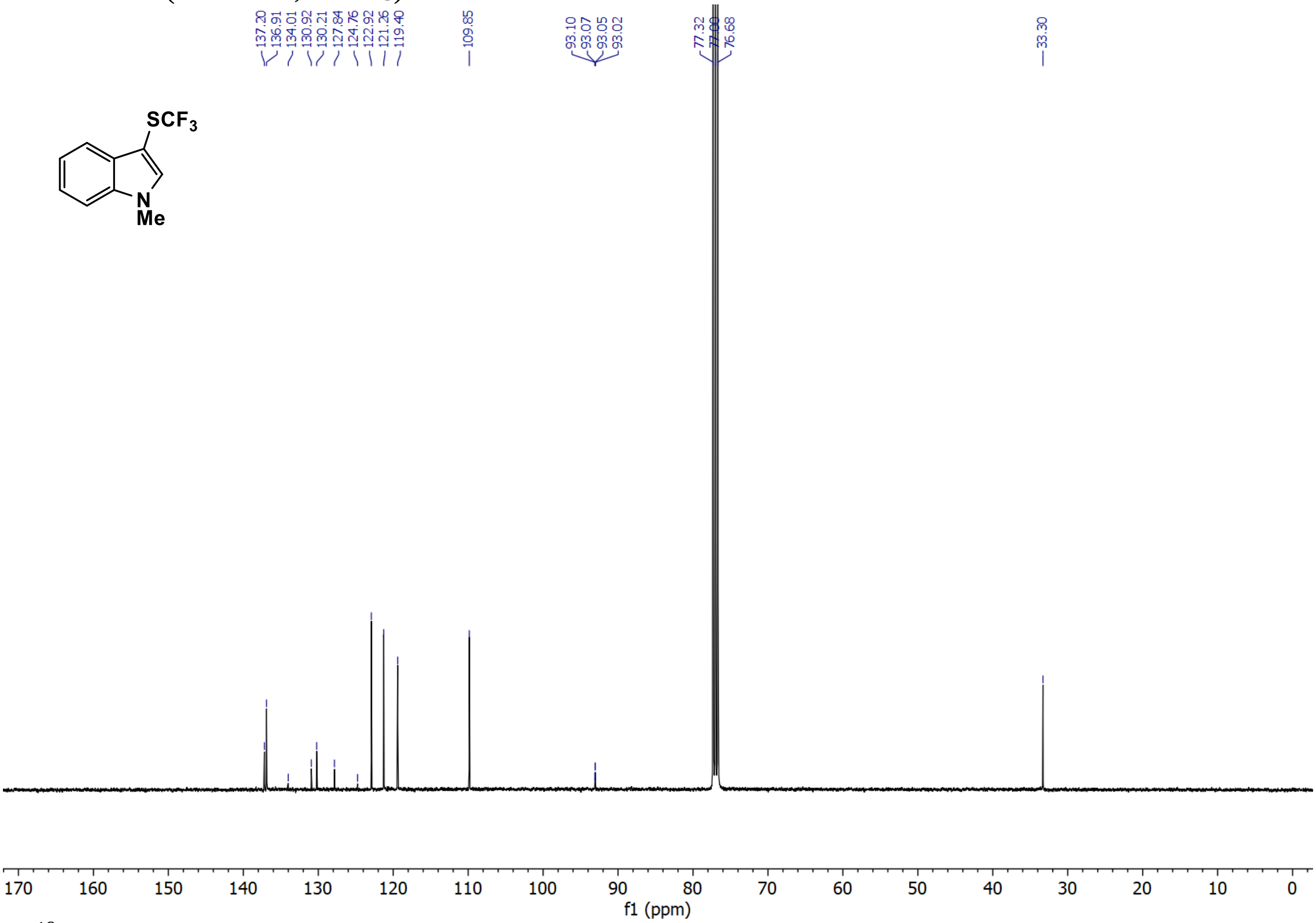

3l ^{19}F NMR (376 MHz, $CDCl_3$)

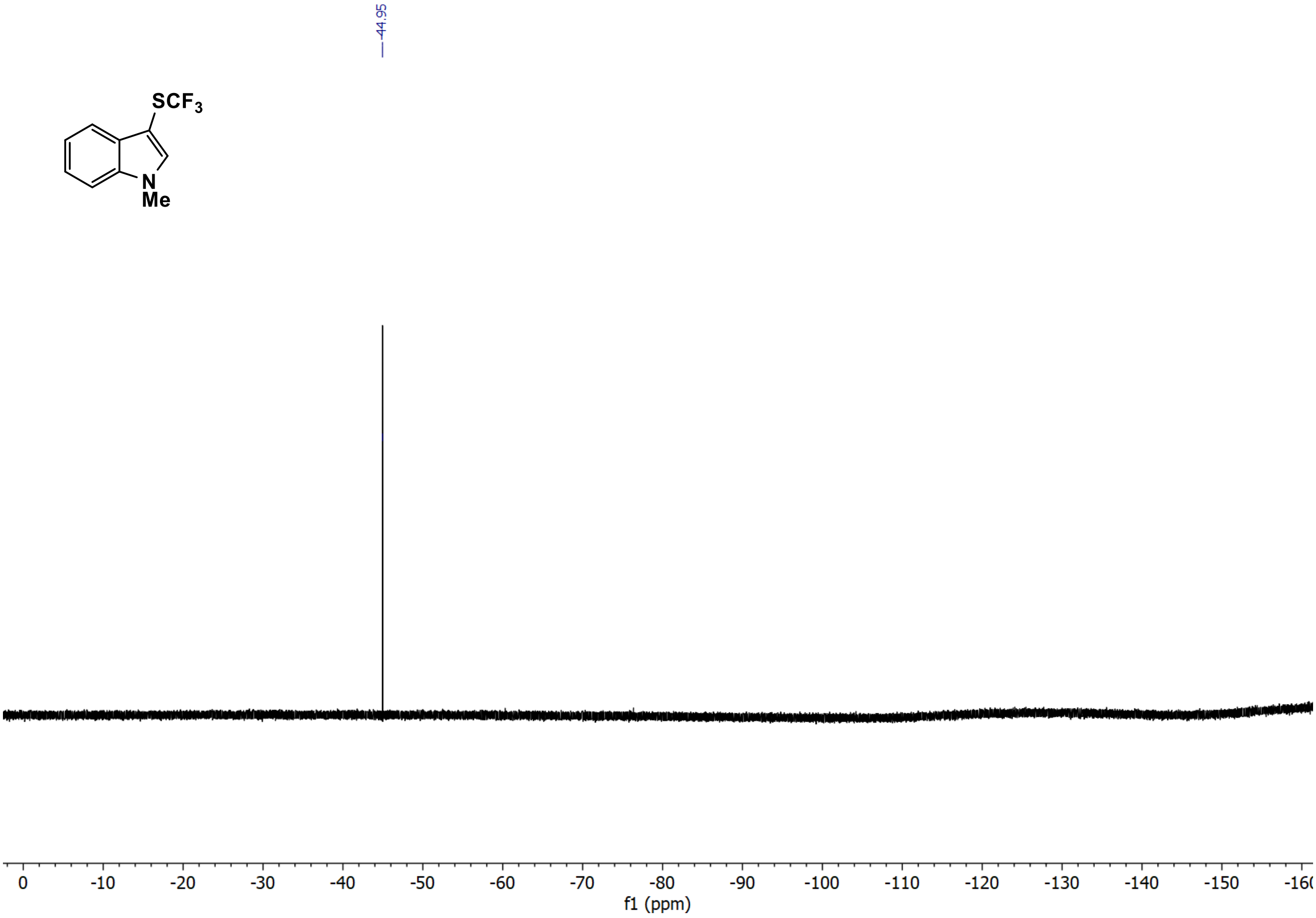

3m ^{1}H NMR (400 MHz, $CDCl_3$)

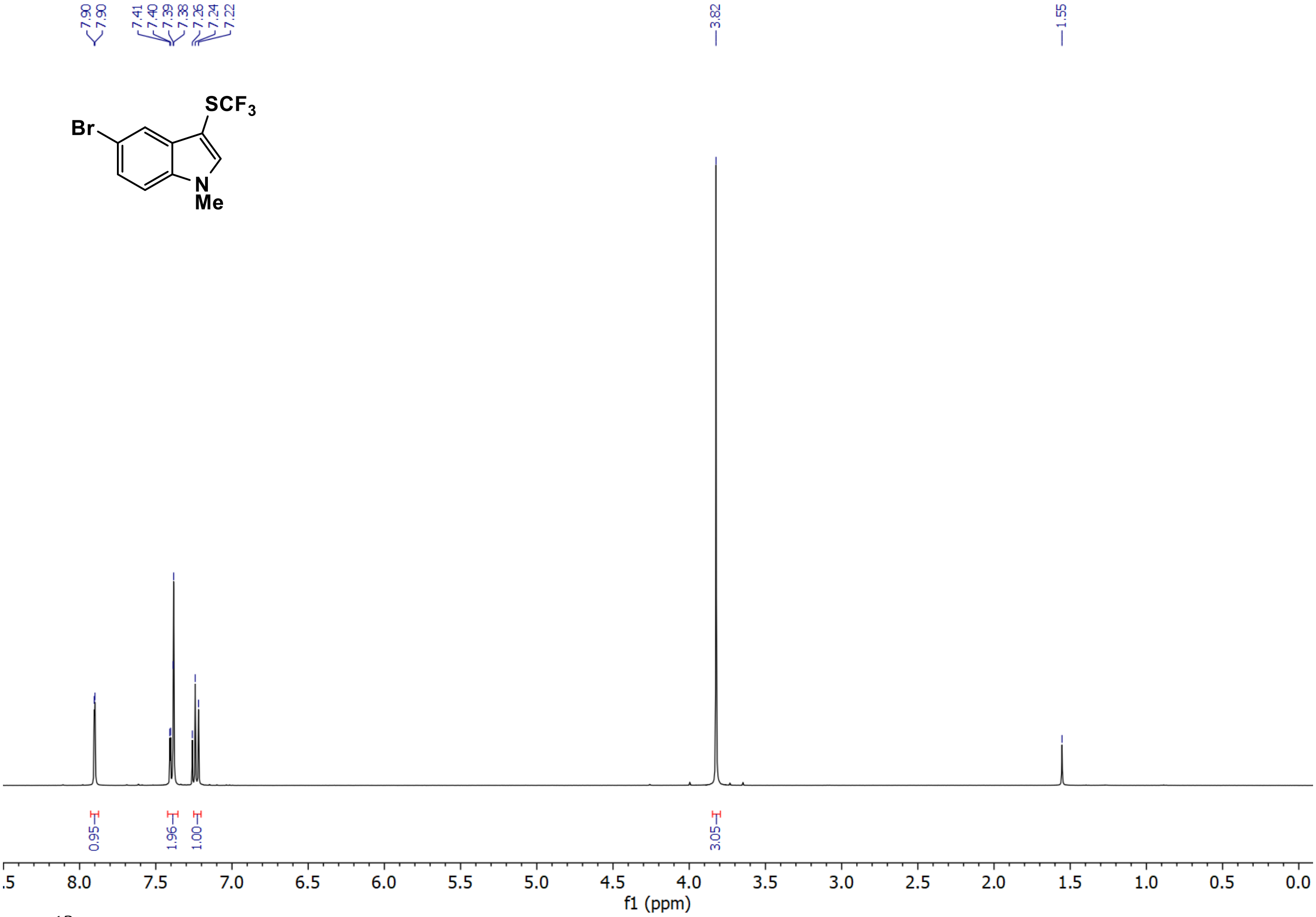

3m ^{13}C NMR (101 MHz, $CDCl_3$)

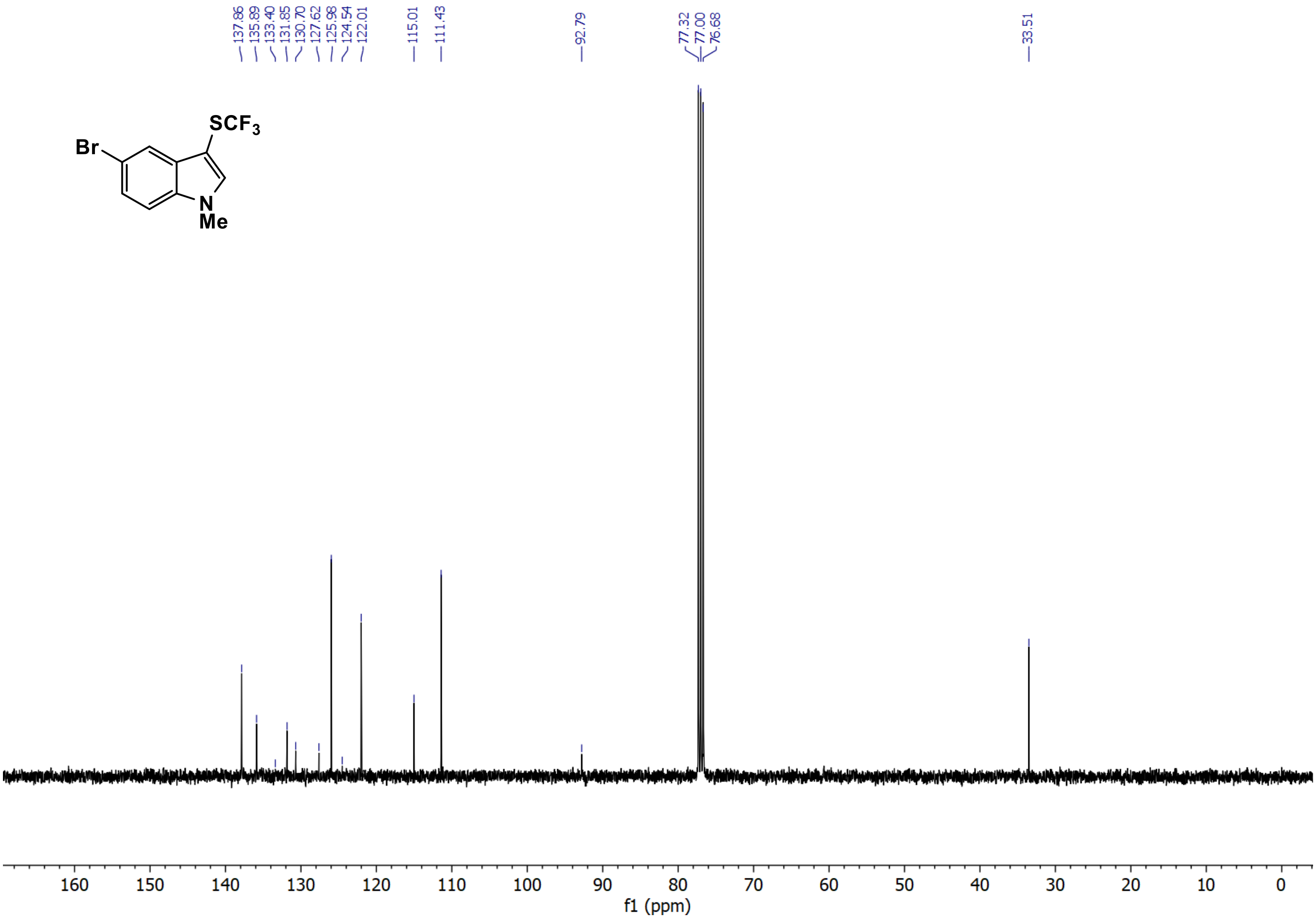

3m ^{19}F NMR (376 MHz, $CDCl_3$)

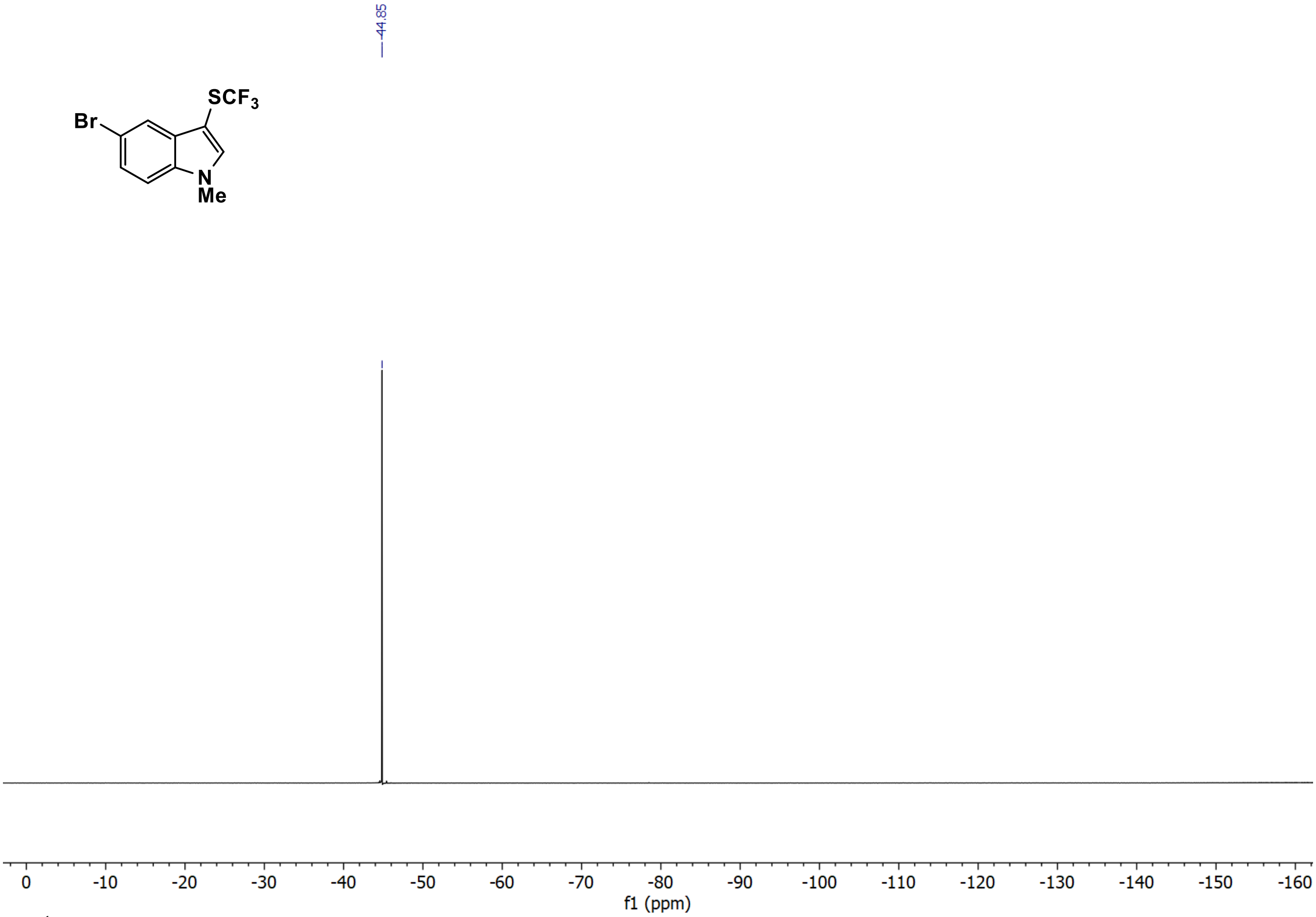

3n ^{1}H NMR (400 MHz, $CDCl_3$)

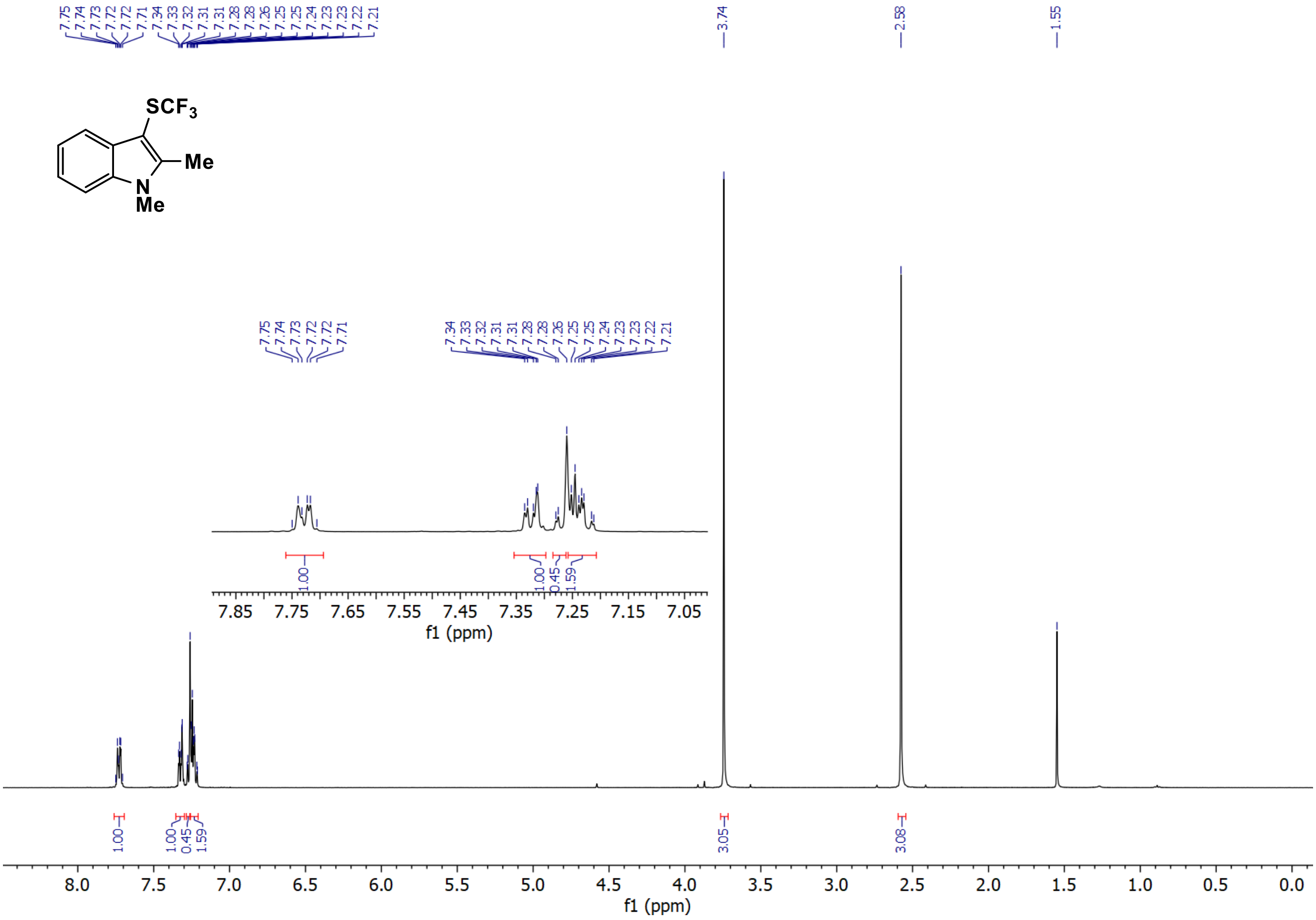

3n ^{13}C NMR (101 MHz, $CDCl_3$)

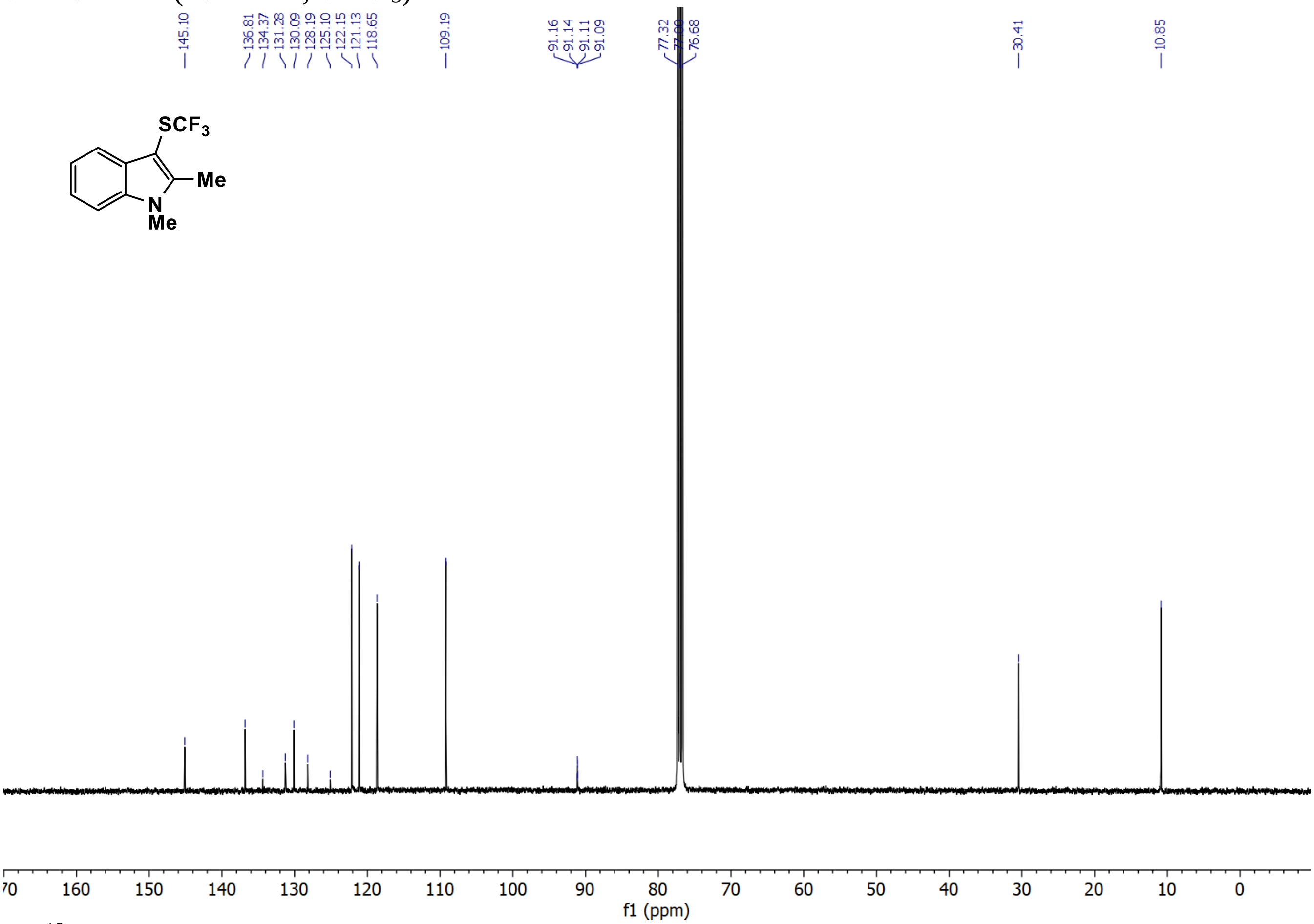

3n ^{19}F NMR (376 MHz, $CDCl_3$)

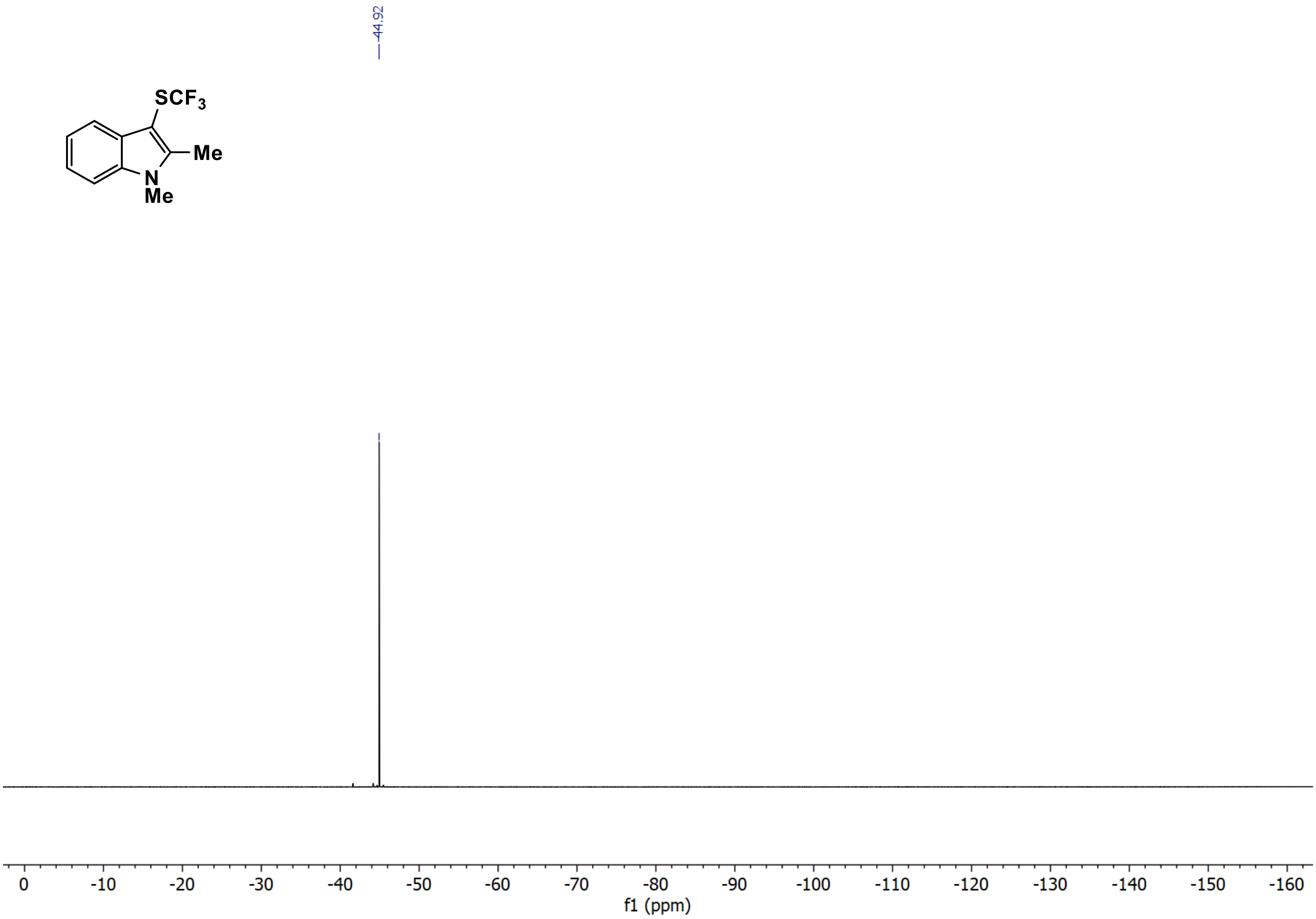

3o ^{1}H NMR (400 MHz, $CDCl_3$)

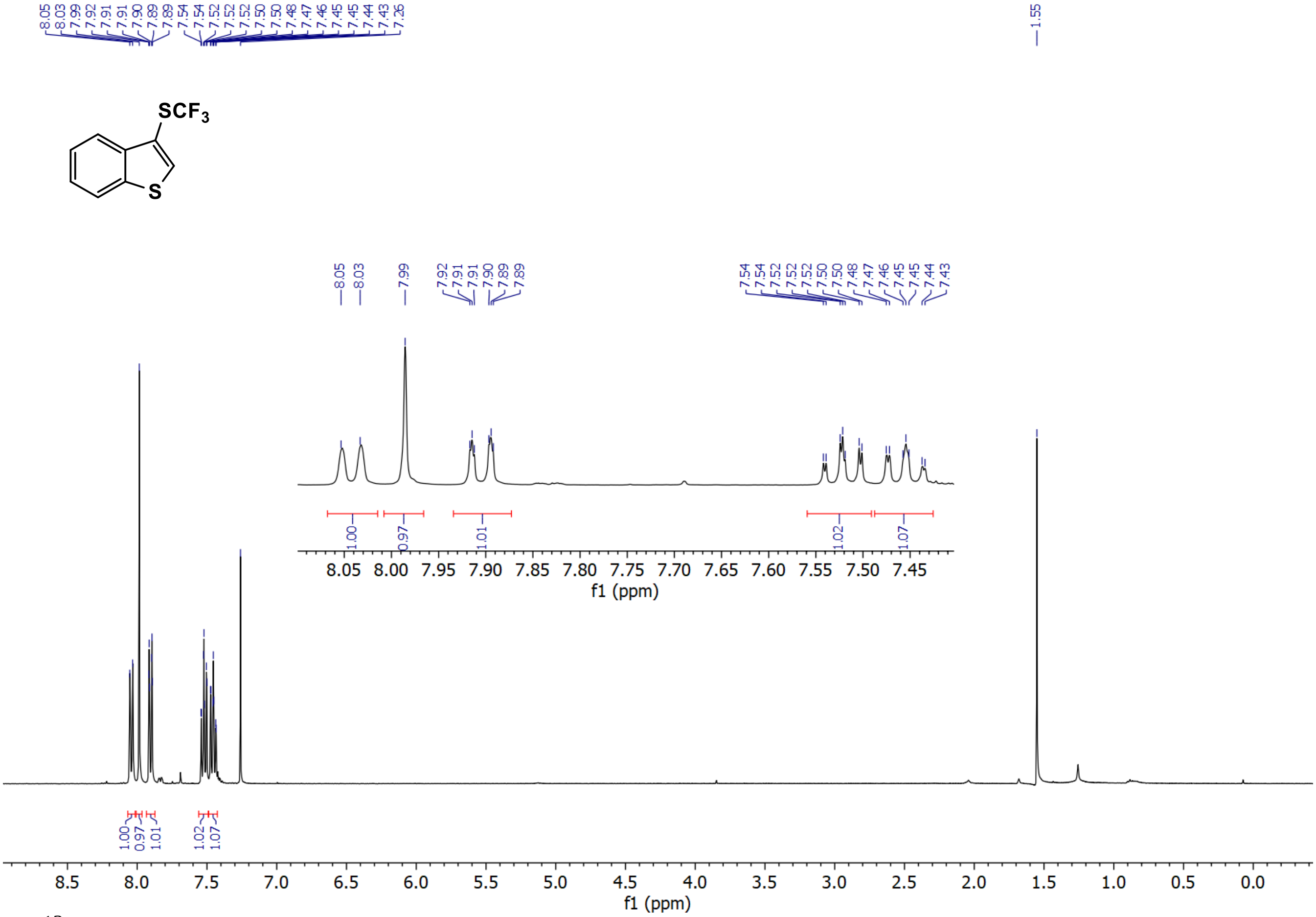

3o ^{13}C NMR (126 MHz, $CDCl_3$)

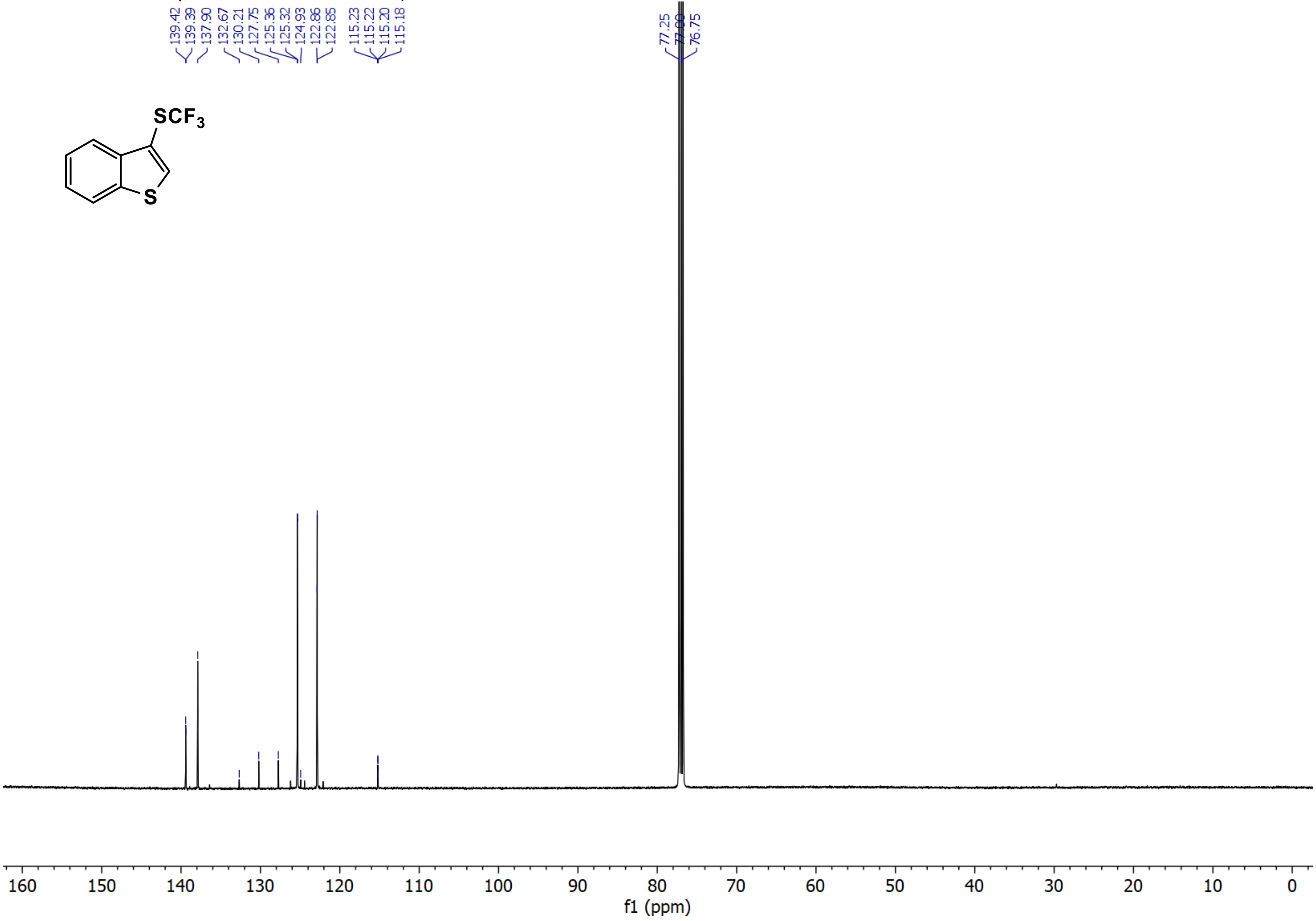

3o ^{19}F NMR (376 MHz, $CDCl_3$)

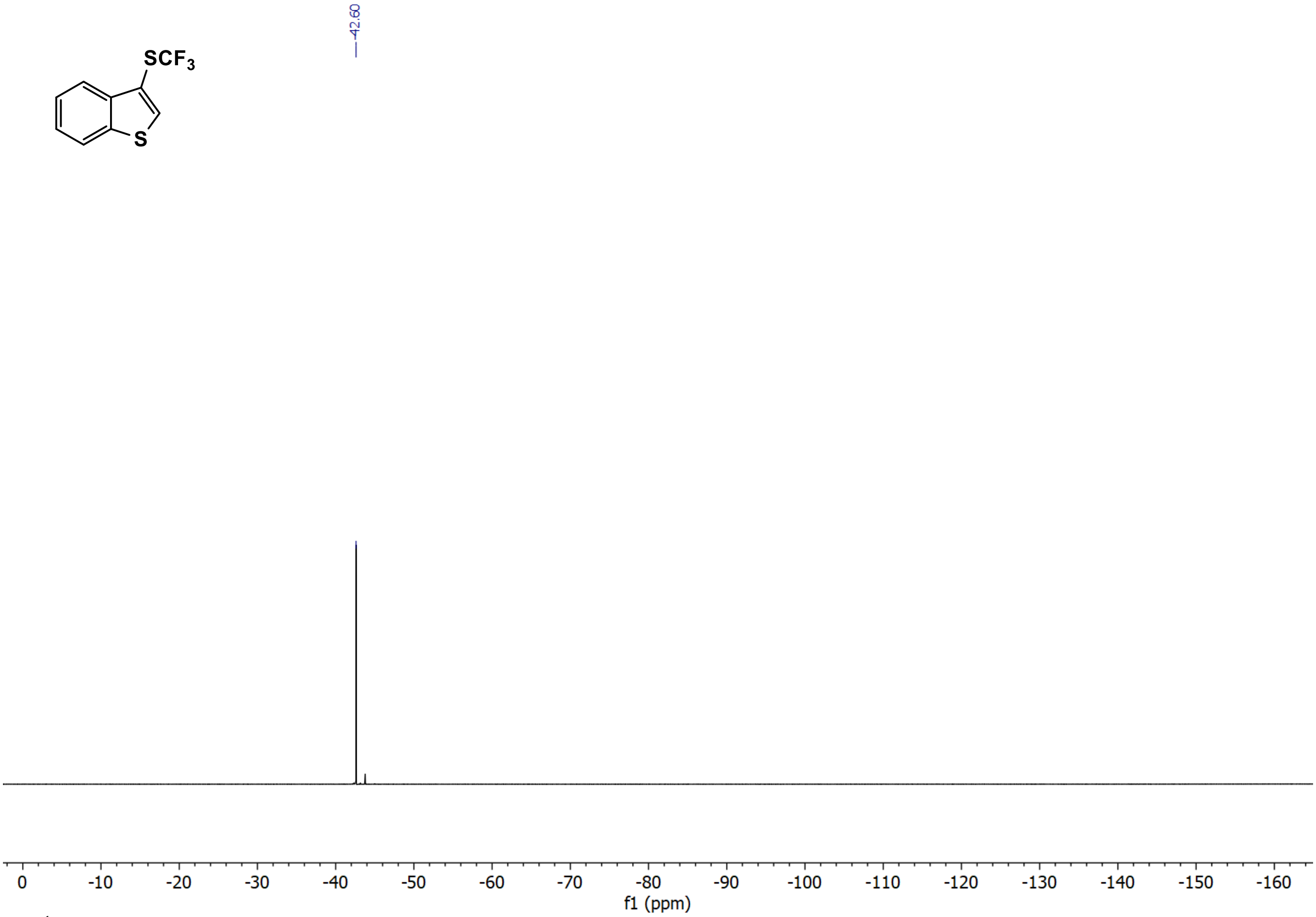

3p ^{1}H NMR (400 MHz, $CDCl_3$)

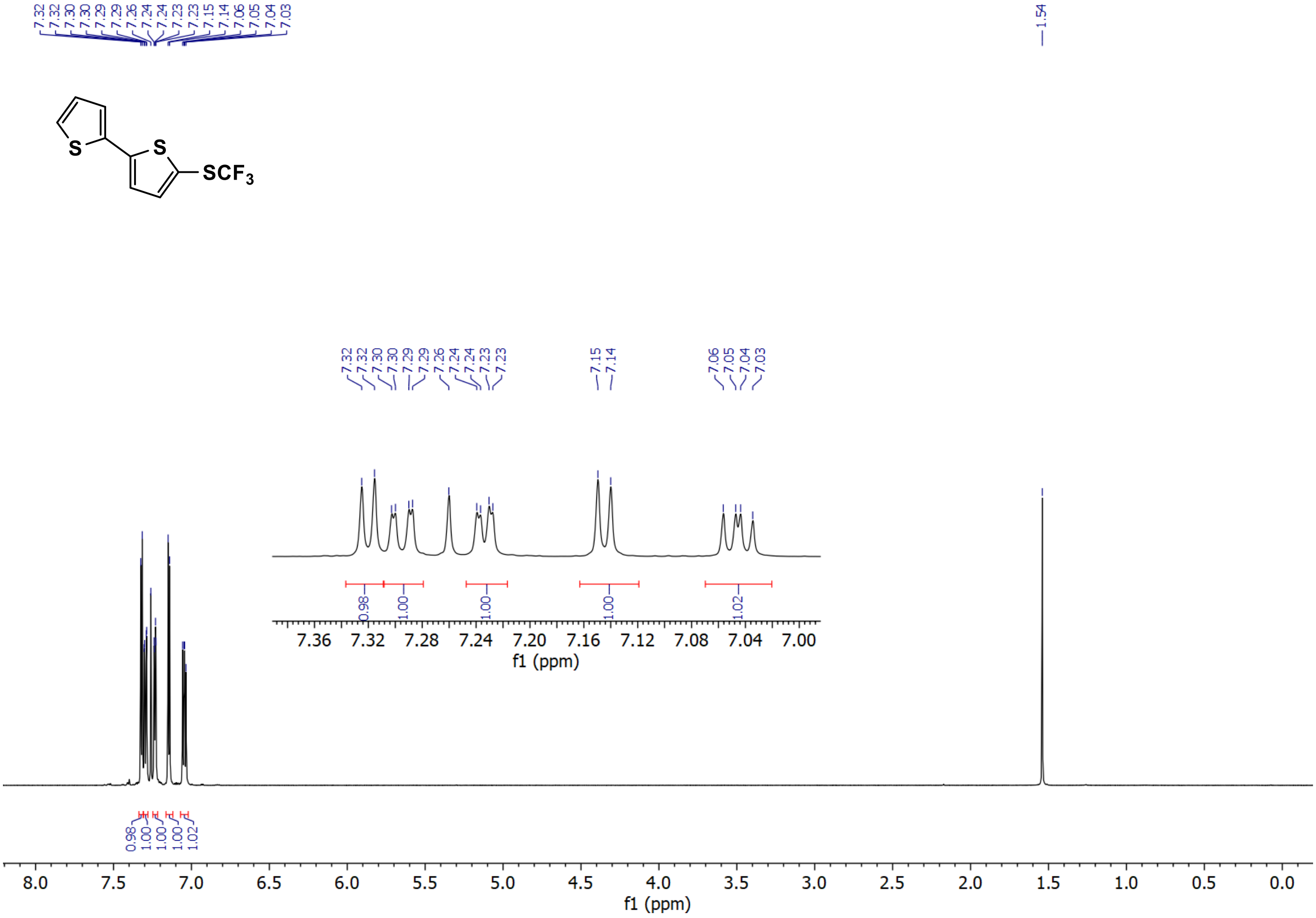

3p ^{13}C NMR (101 MHz, $CDCl_3$)

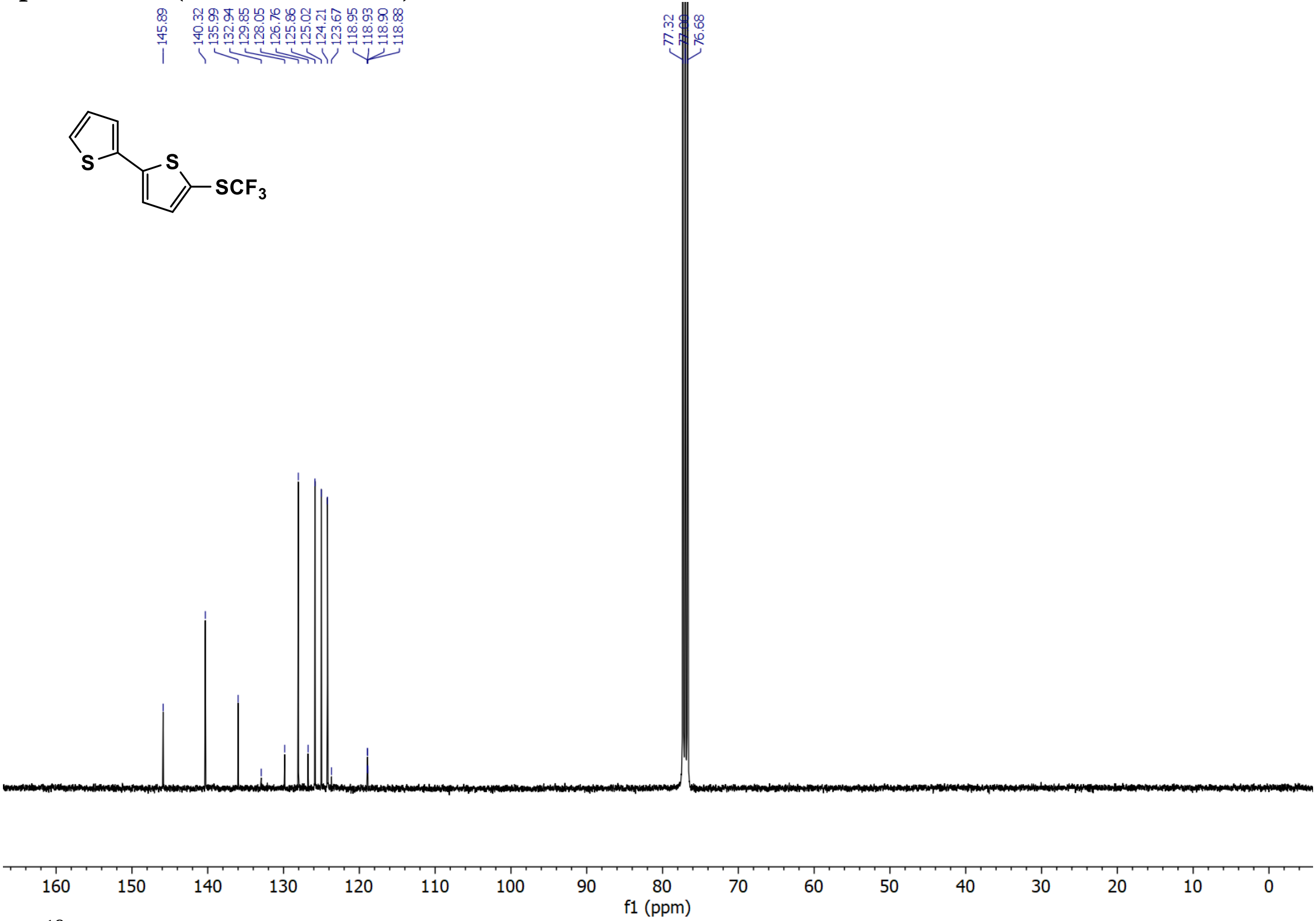

3p ^{19}F NMR (376 MHz, $CDCl_3$)

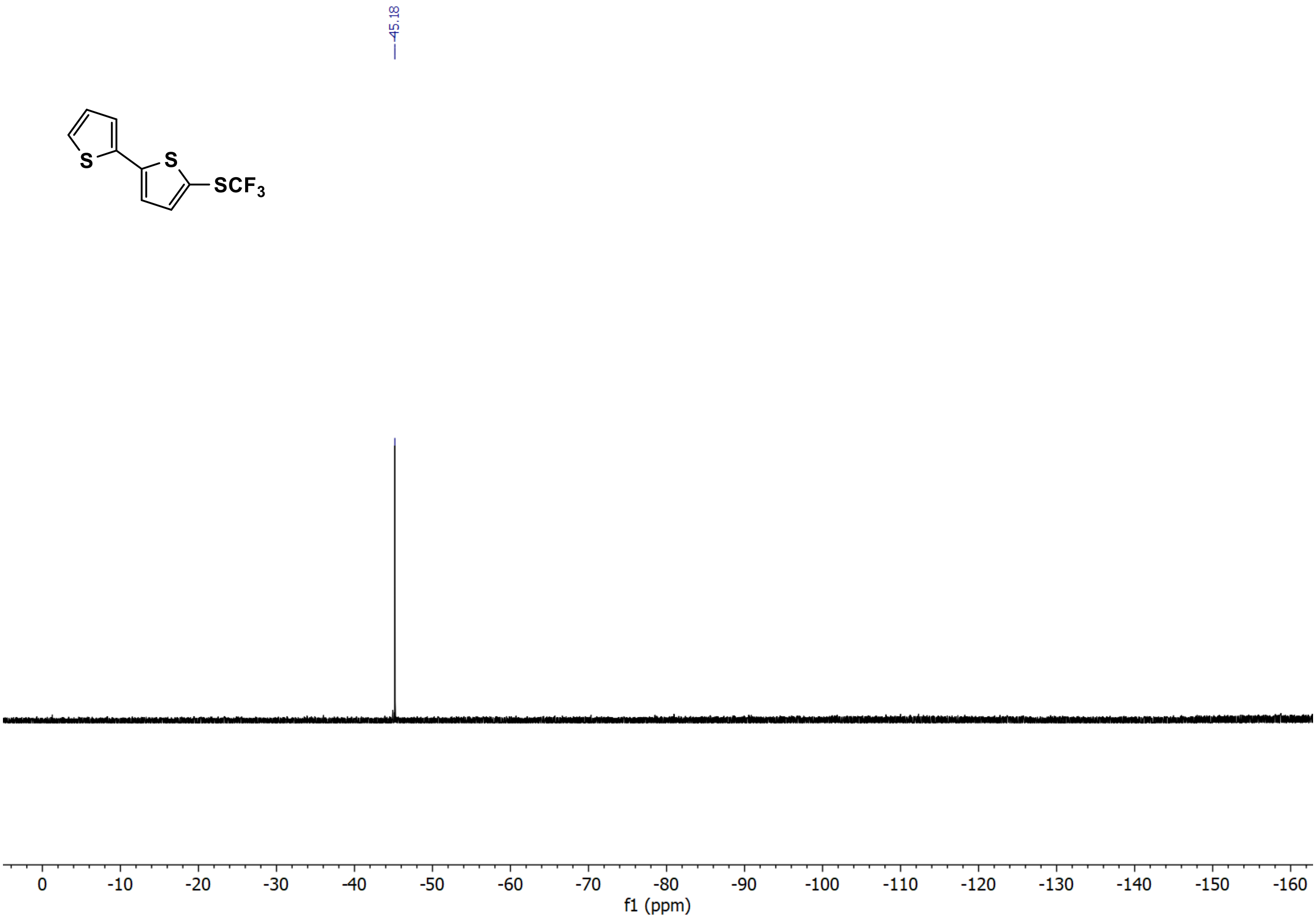

3q ^{1}H NMR (400 MHz, $CDCl_3$)

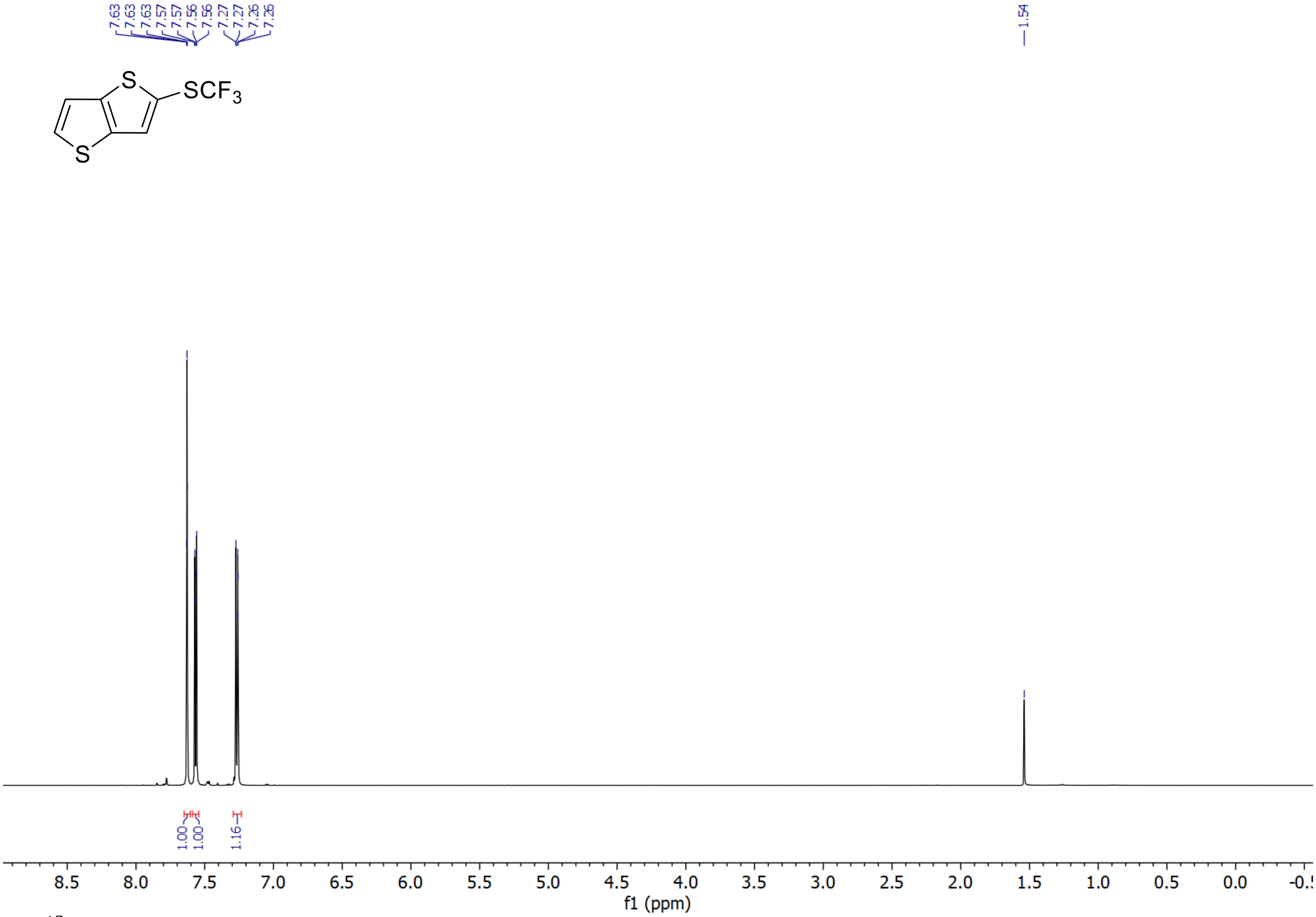

3q ^{13}C NMR (101 MHz, $CDCl_3$)

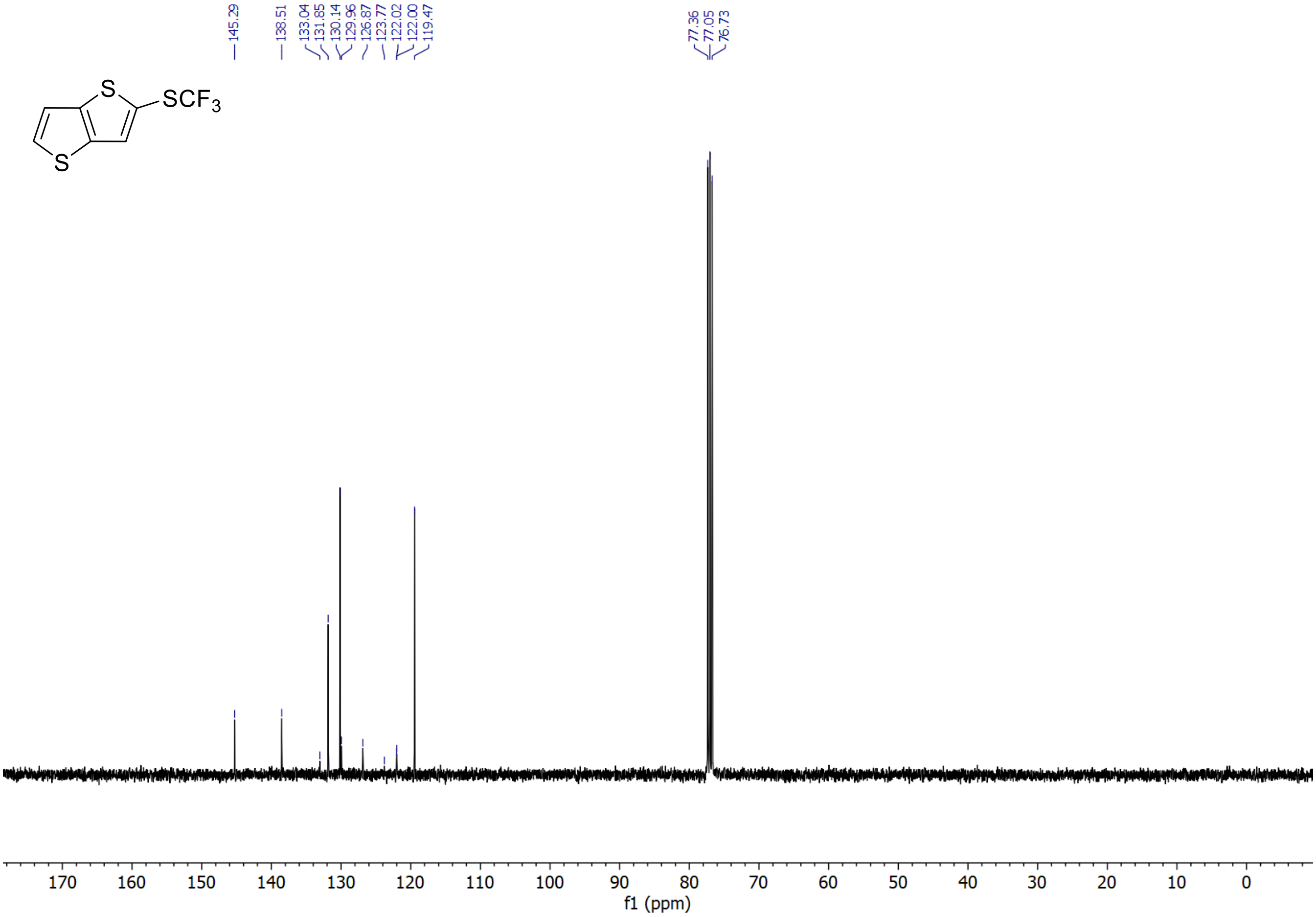

3q ^{19}F NMR (376 MHz, $CDCl_3$)

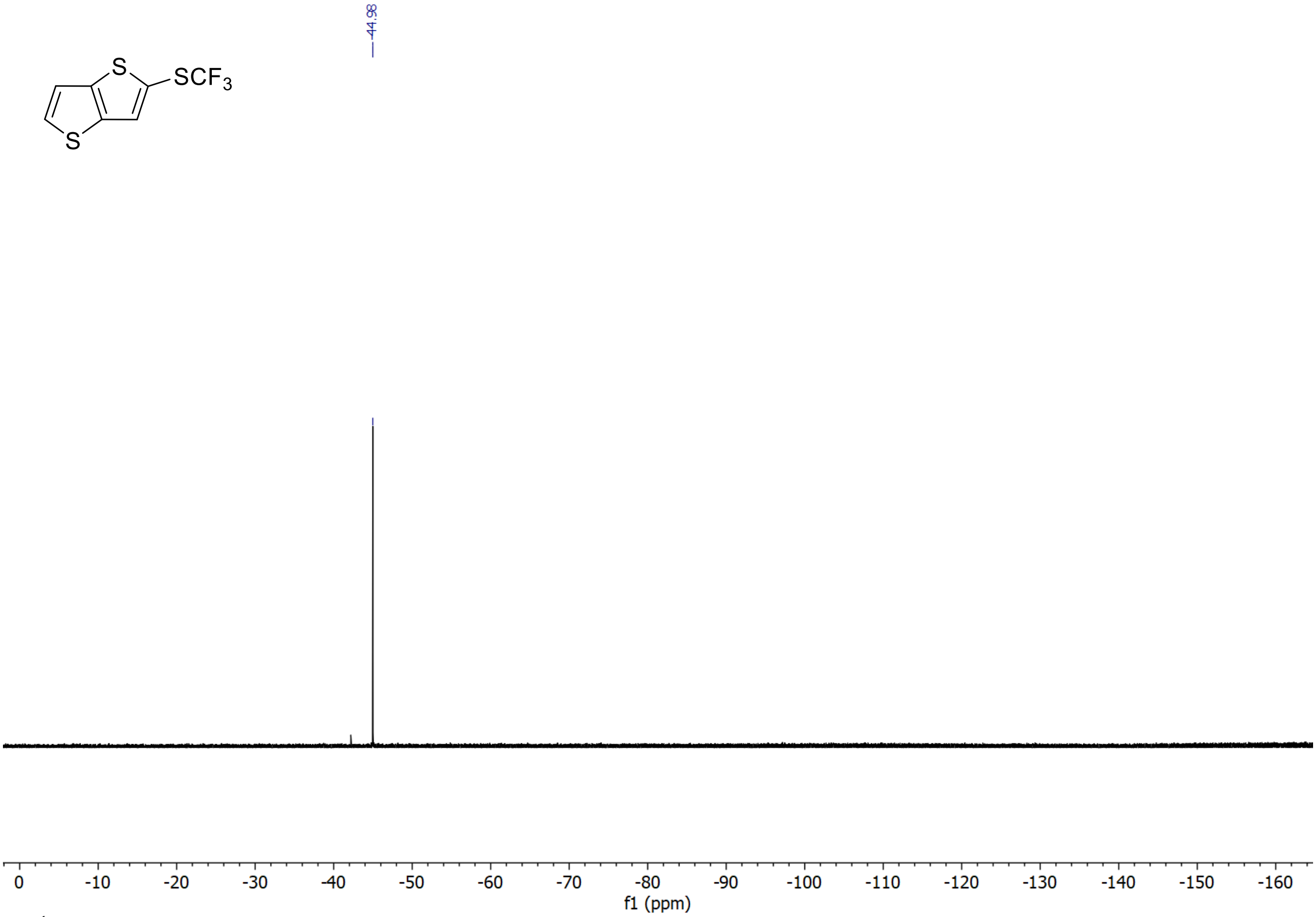

3r ^{1}H NMR (400 MHz, $CDCl_3$)

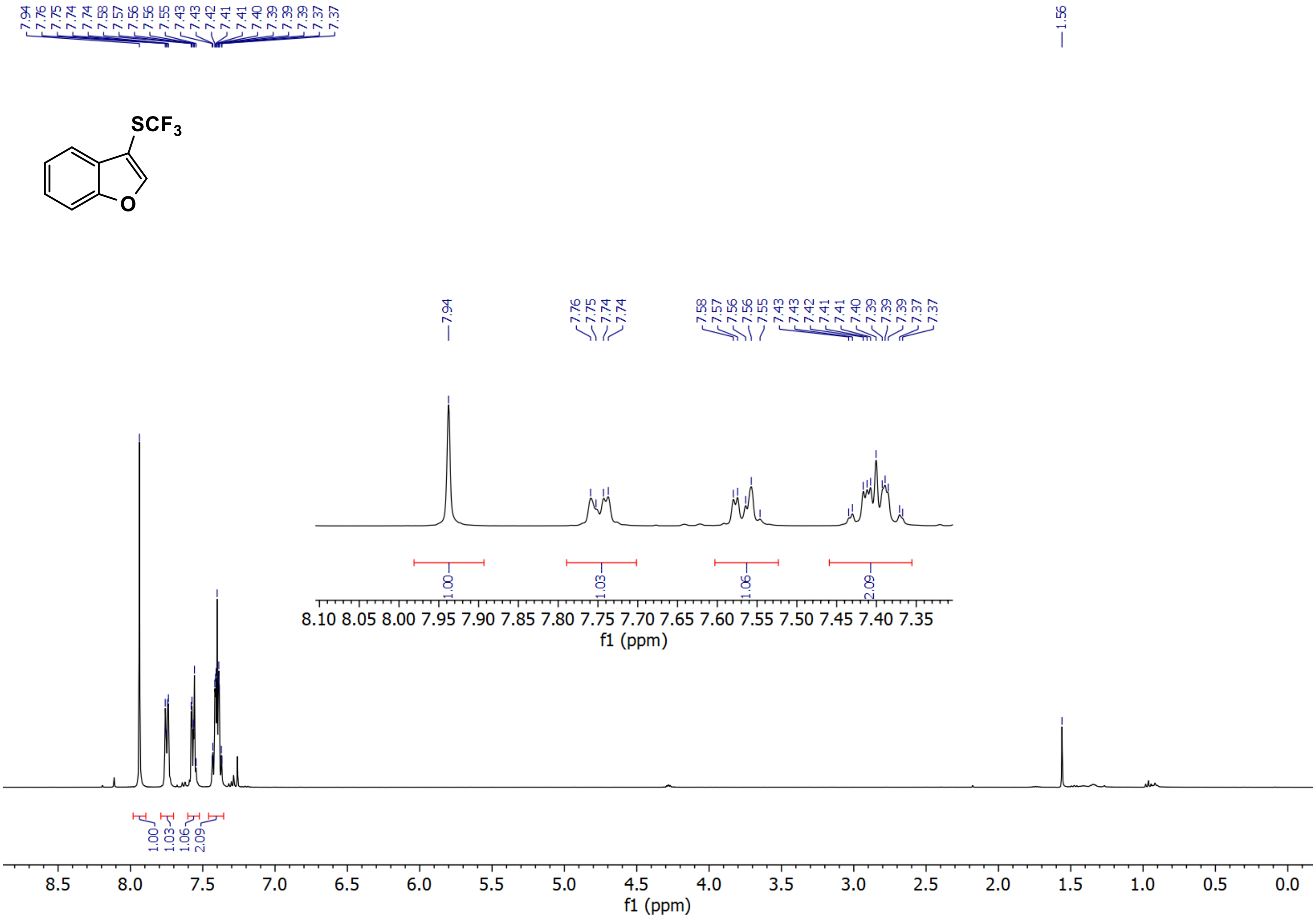

3r ^{13}C NMR (101 MHz, $CDCl_3$)

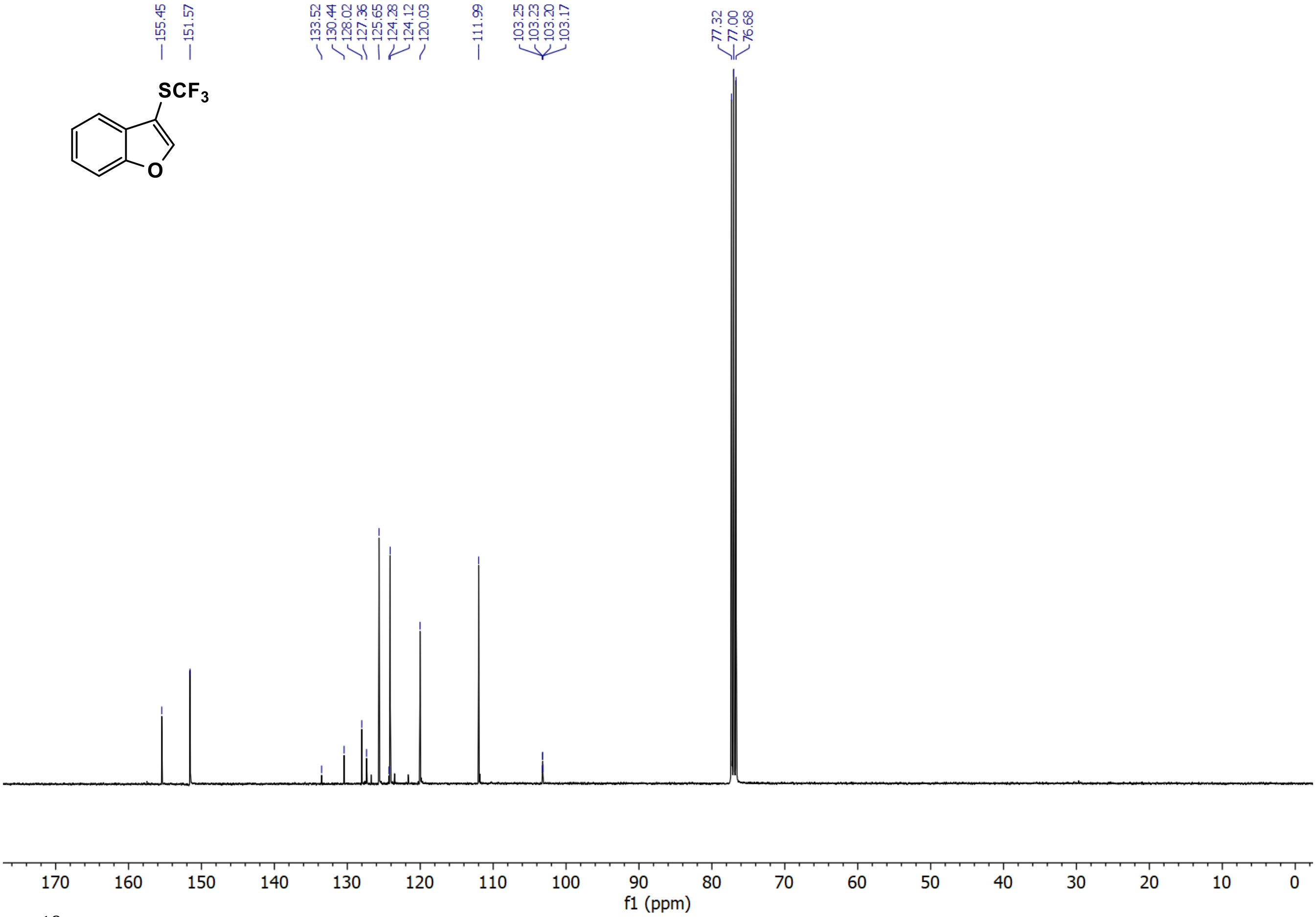

3r ^{19}F NMR (376 MHz, $CDCl_3$)

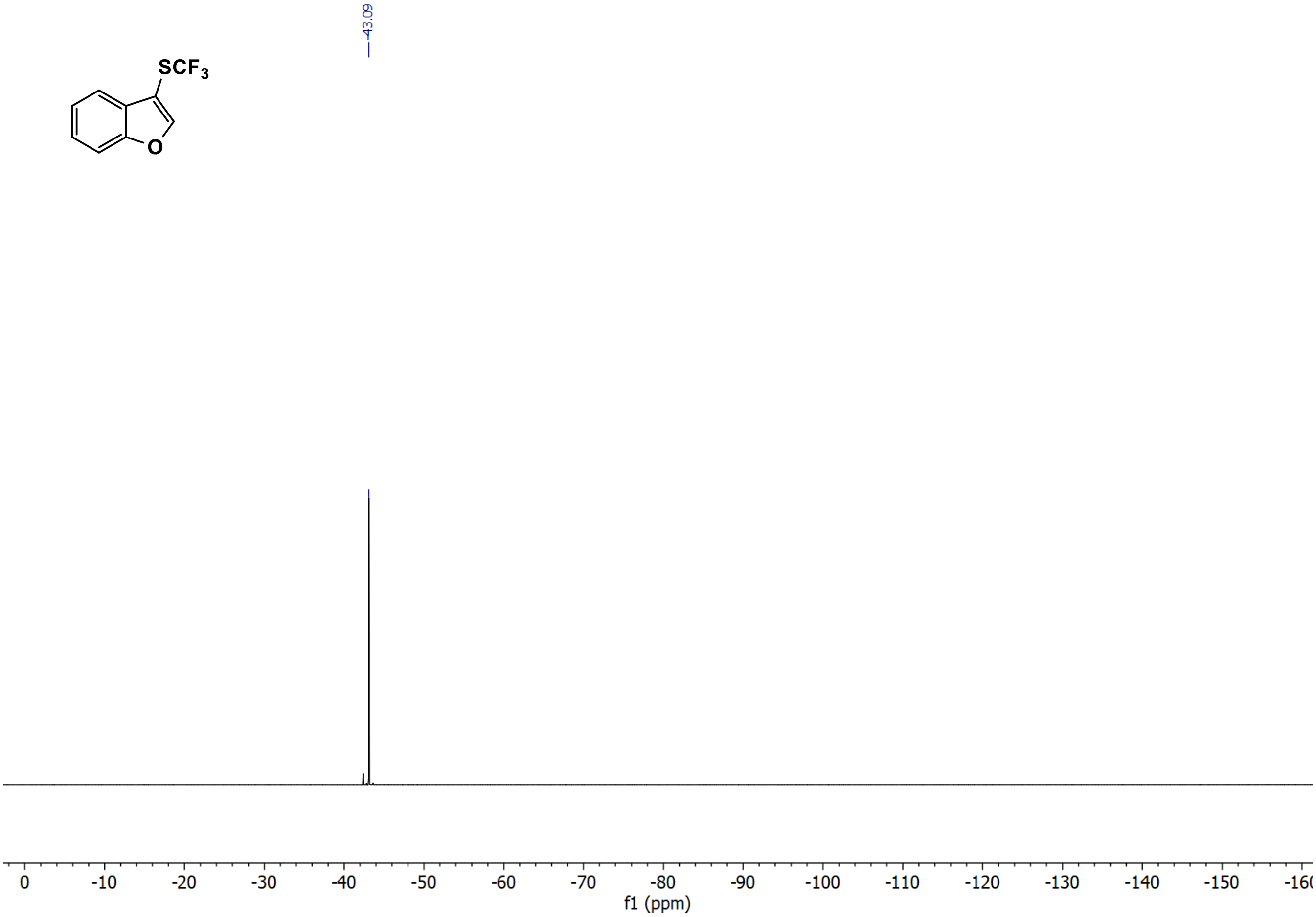

2-**3s** ^{1}H NMR (400 MHz, $CDCl_3$)

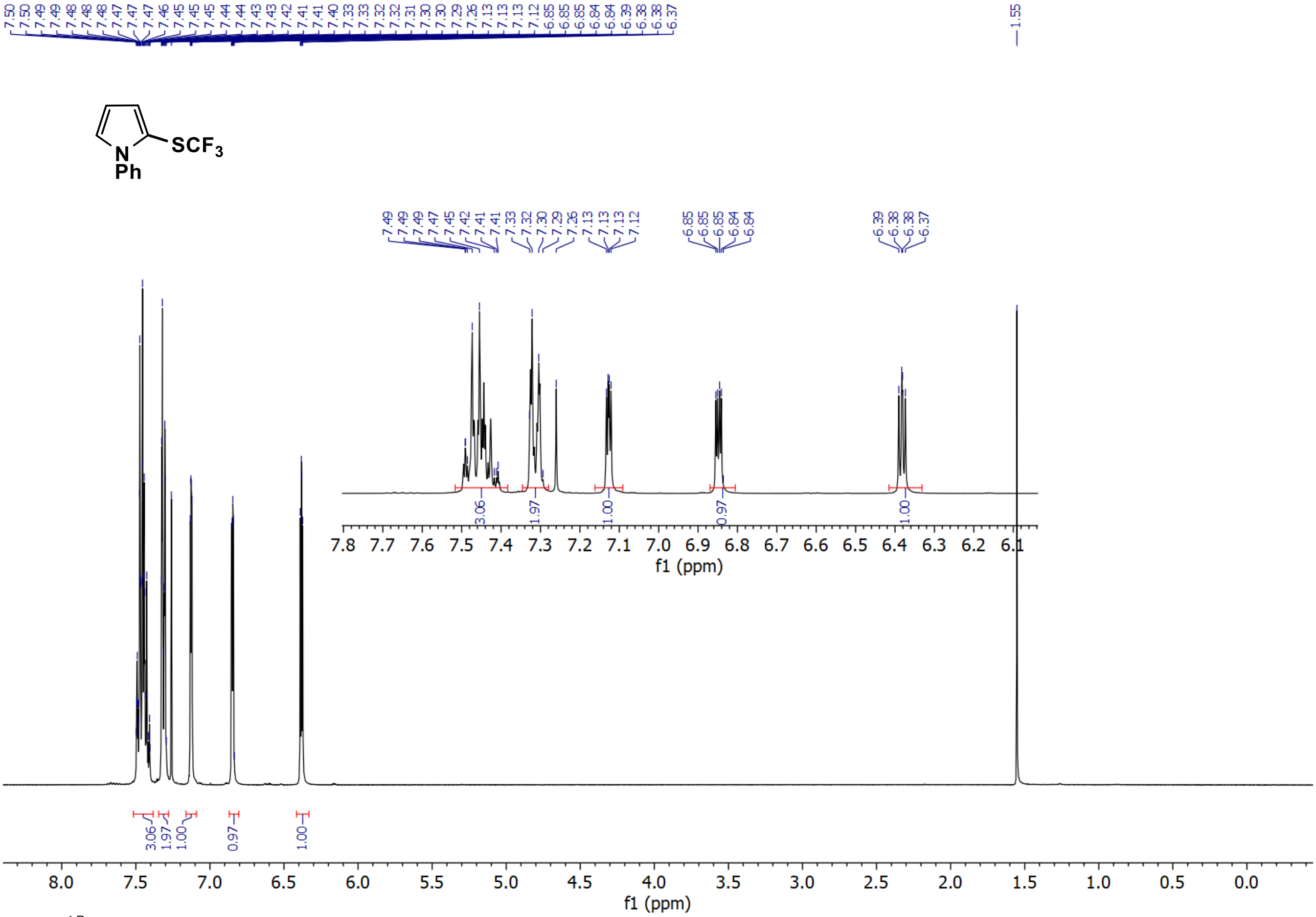

2-**3s** ^{13}C NMR (101 MHz, $CDCl_3$)

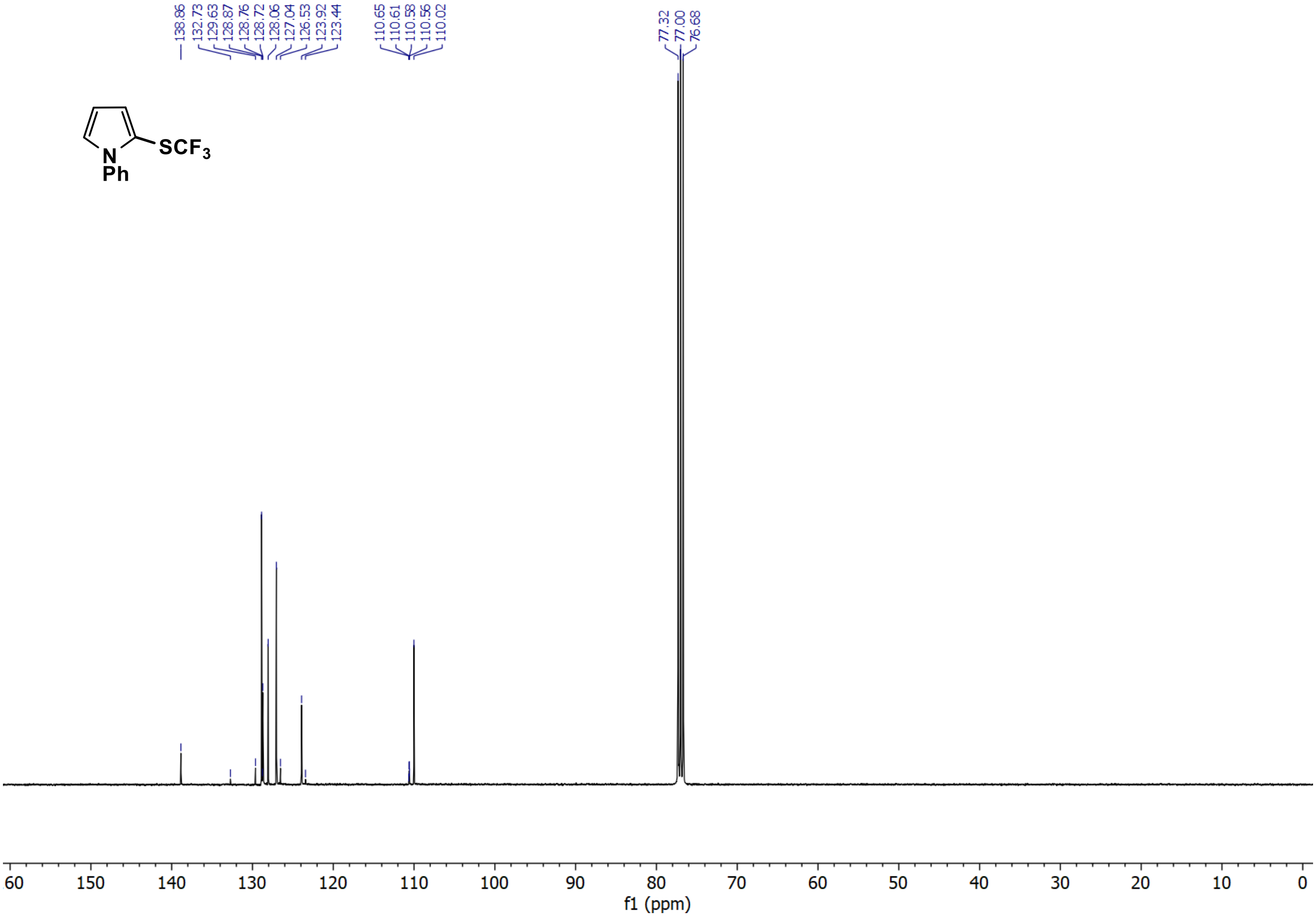

2-**3s** ^{19}F NMR (376 MHz, $CDCl_3$)

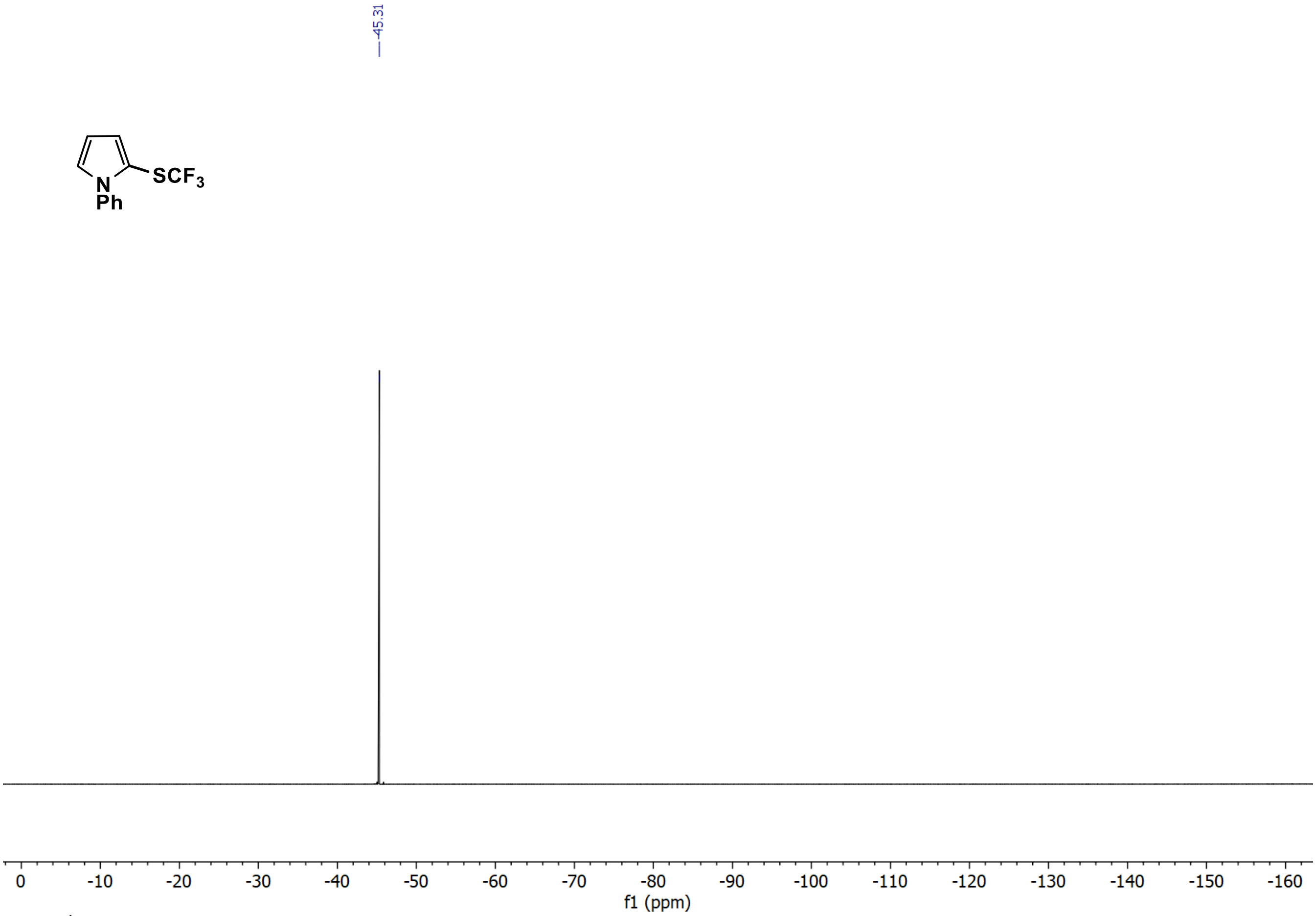

3-**3s** ^{1}H NMR (400 MHz, $CDCl_3$)

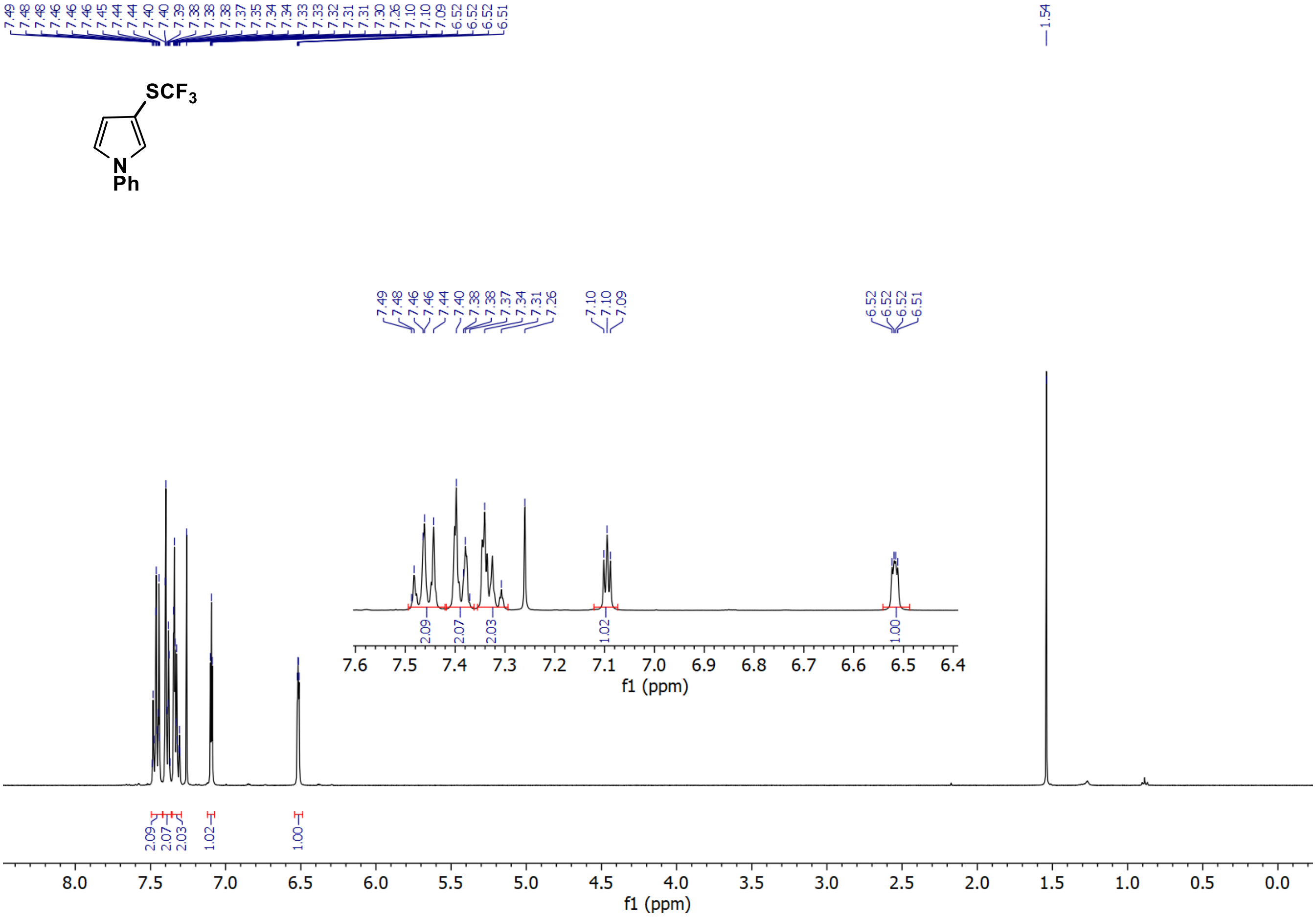

3-**3s** ^{13}C NMR (101 MHz, $CDCl_3$)

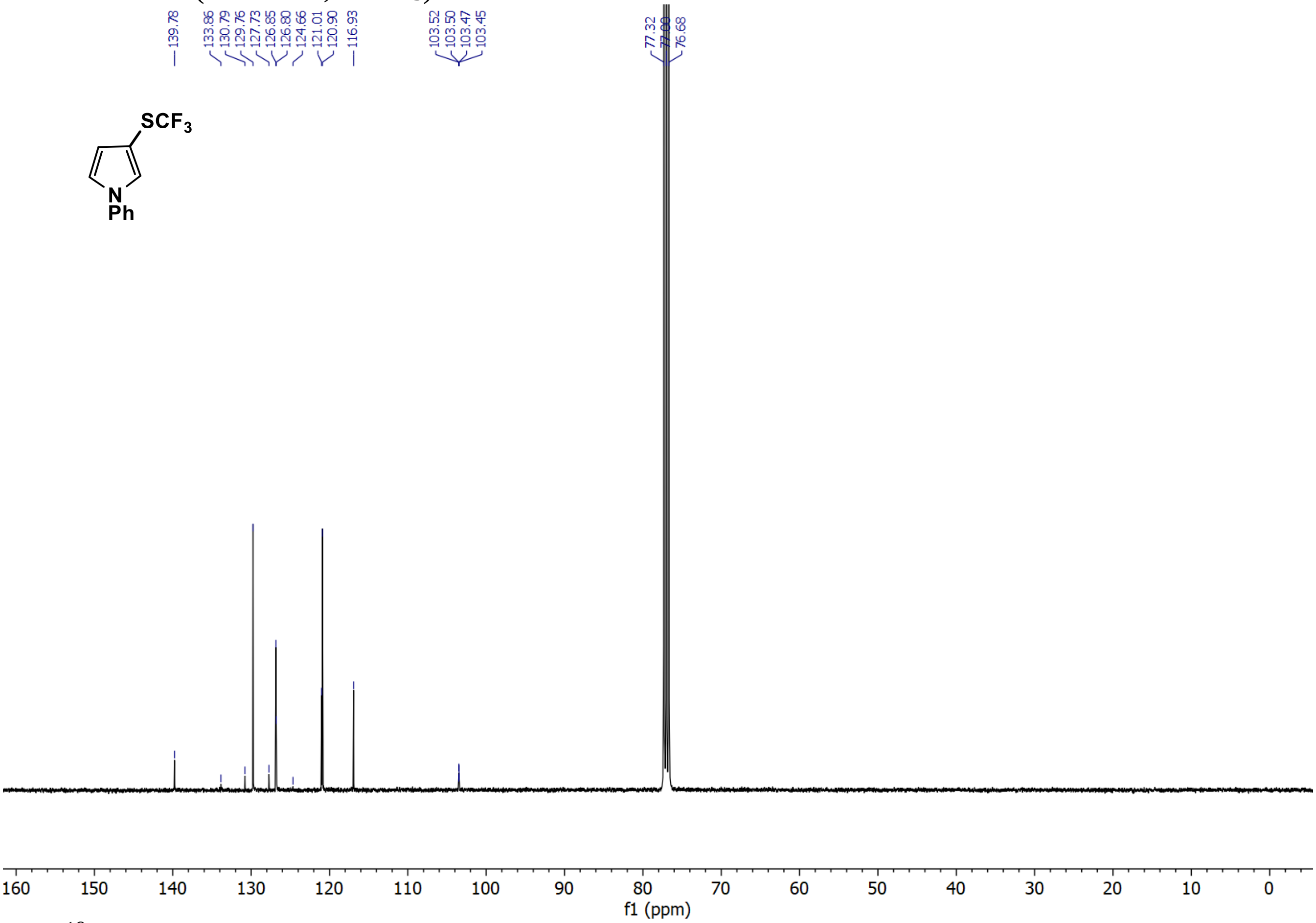

3-**3s** ^{19}F NMR (376 MHz, $CDCl_3$)

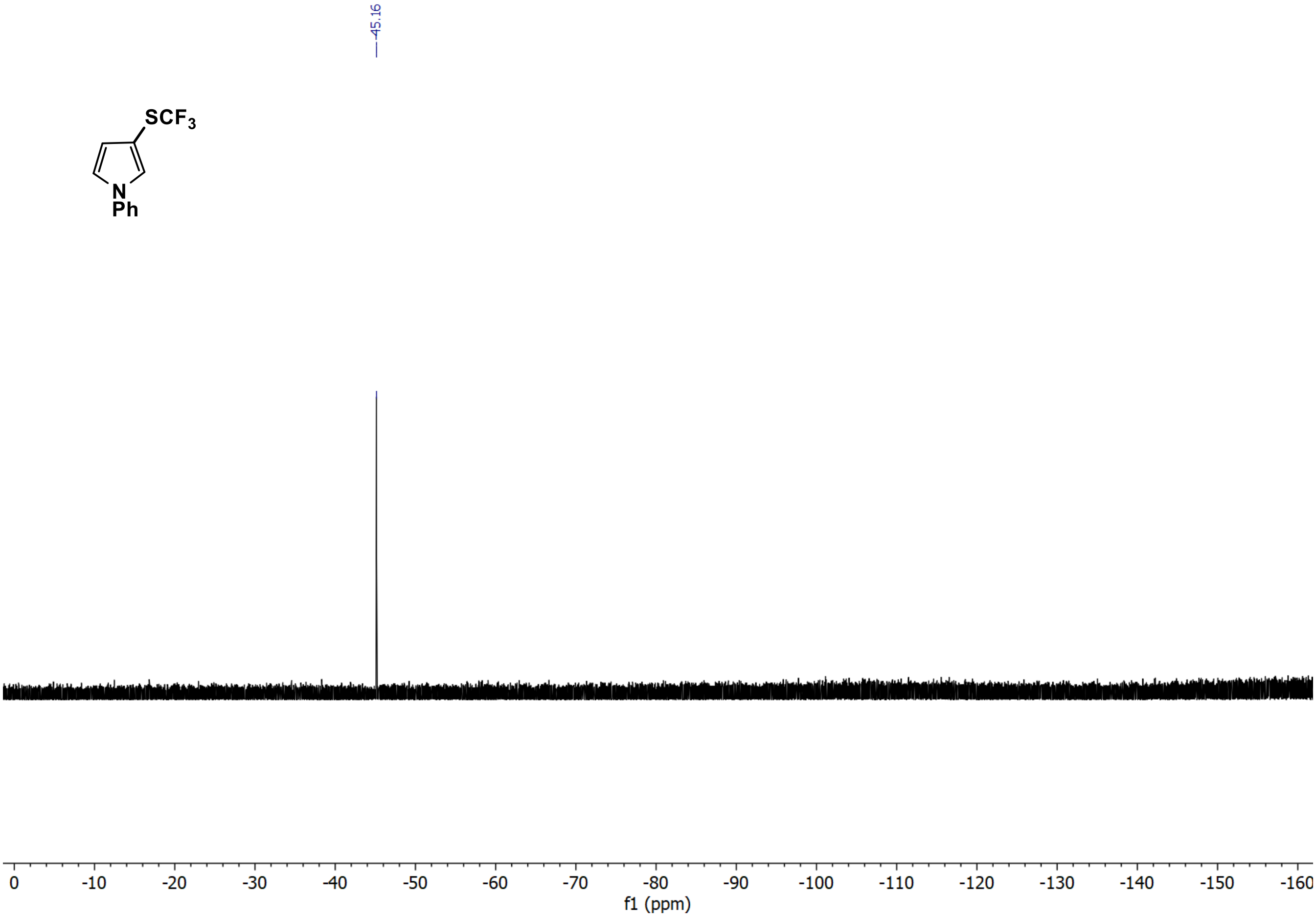

3t ^{1}H NMR (400 MHz, $CDCl_3$)

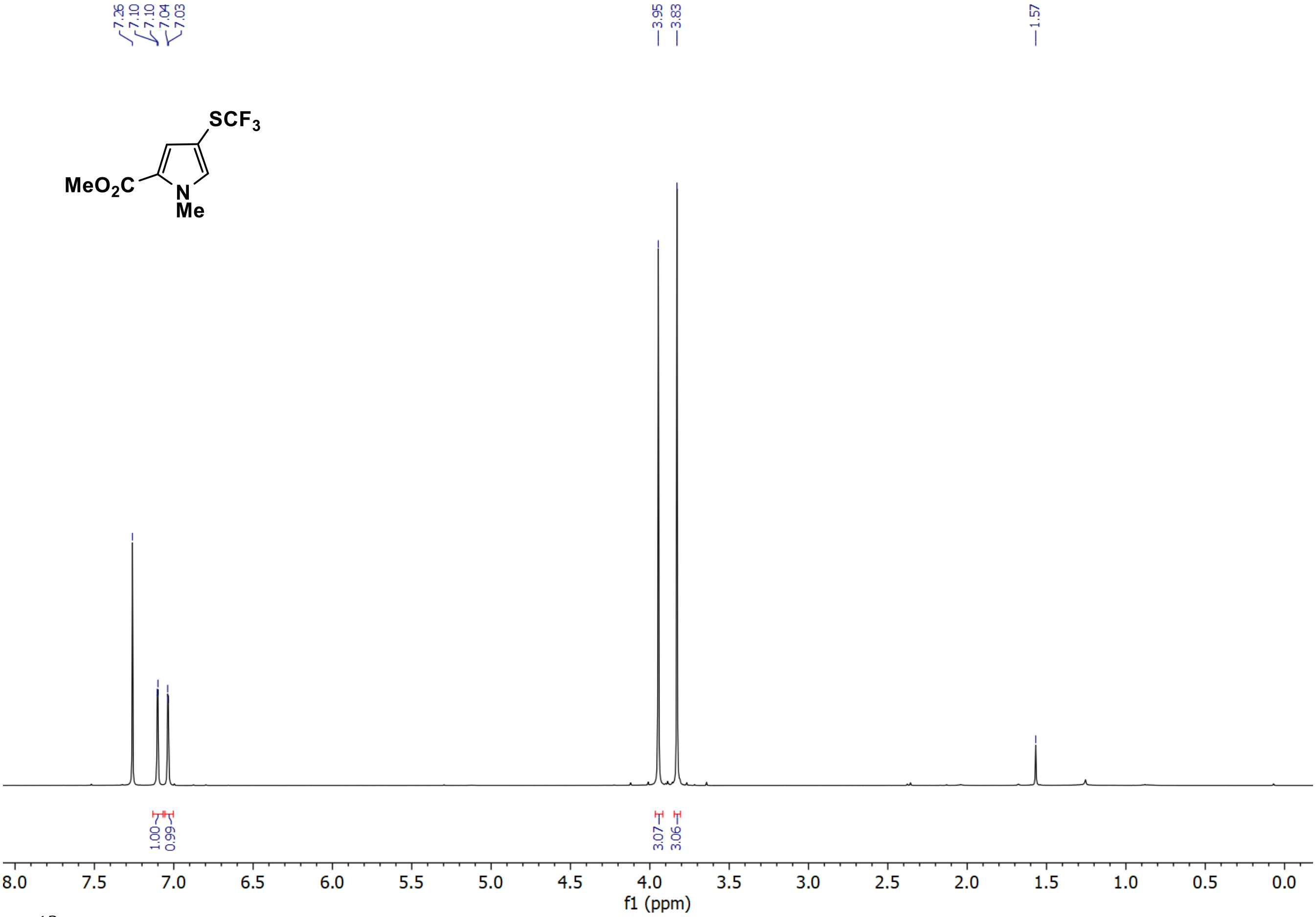

3t ^{13}C NMR (101 MHz, $CDCl_3$)

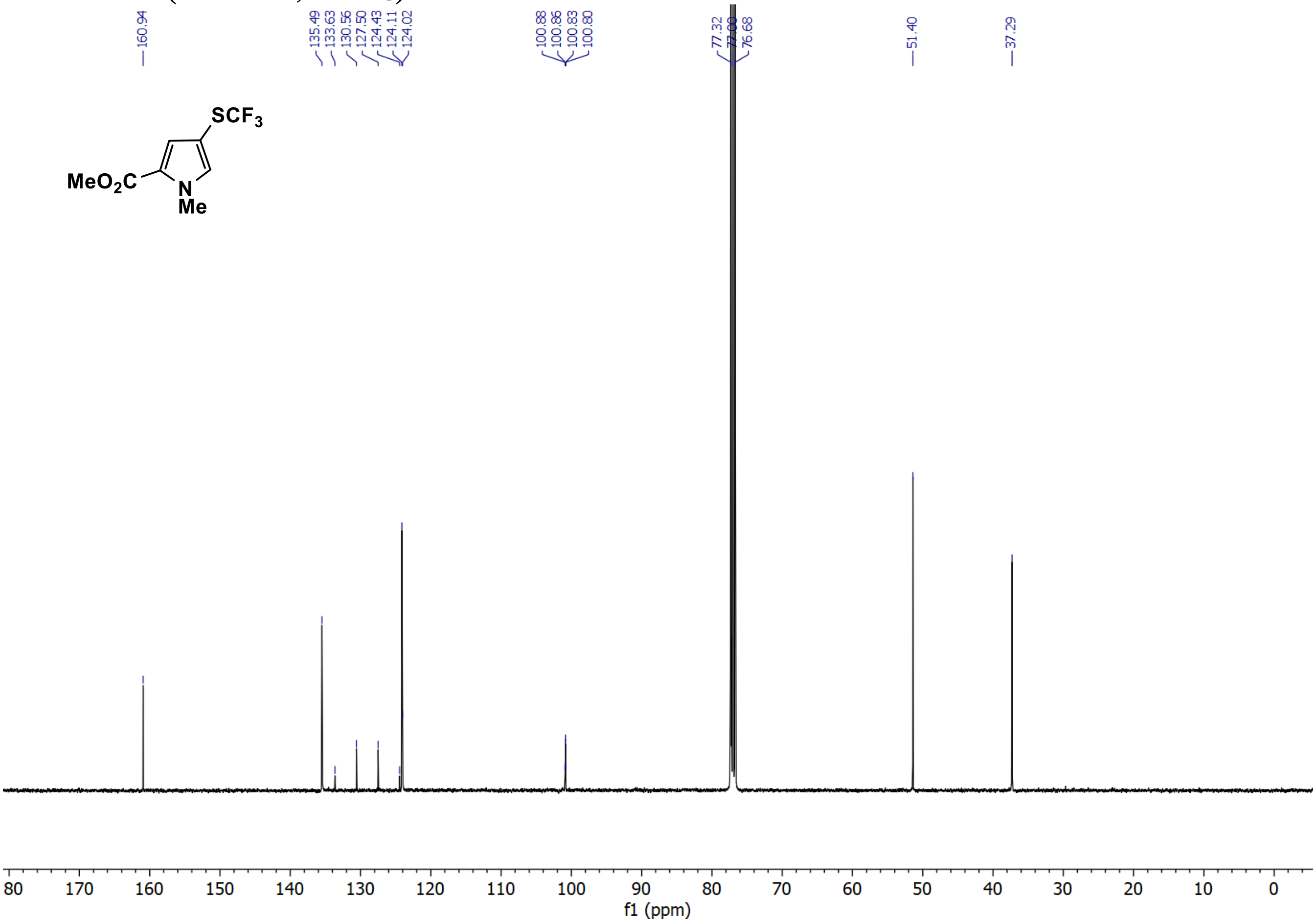

3t ^{19}F NMR (376 MHz, $CDCl_3$)

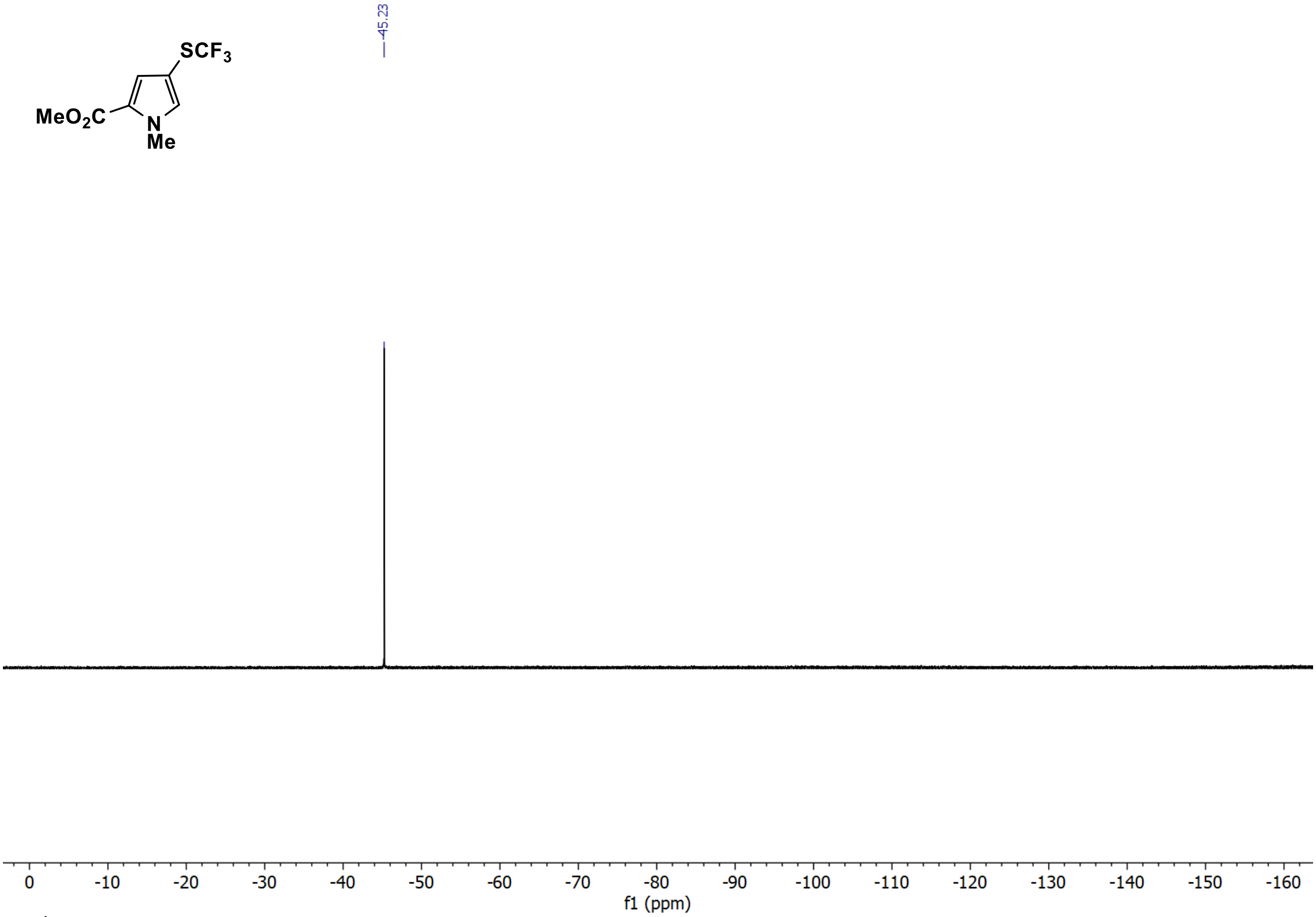

5a ^{1}H NMR (400 MHz, $CDCl_3$)

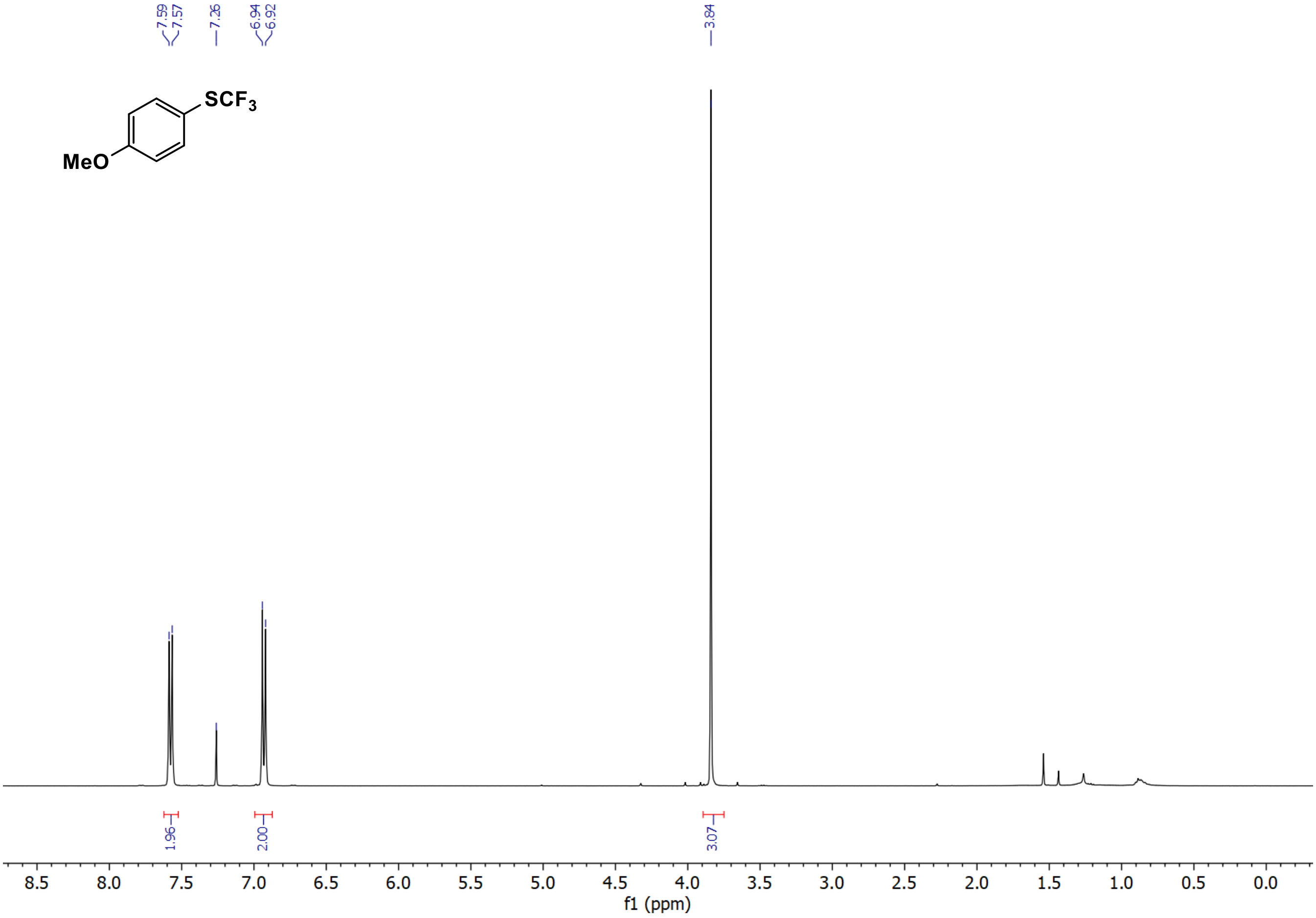

5a ^{13}C NMR (101 MHz, $CDCl_3$)

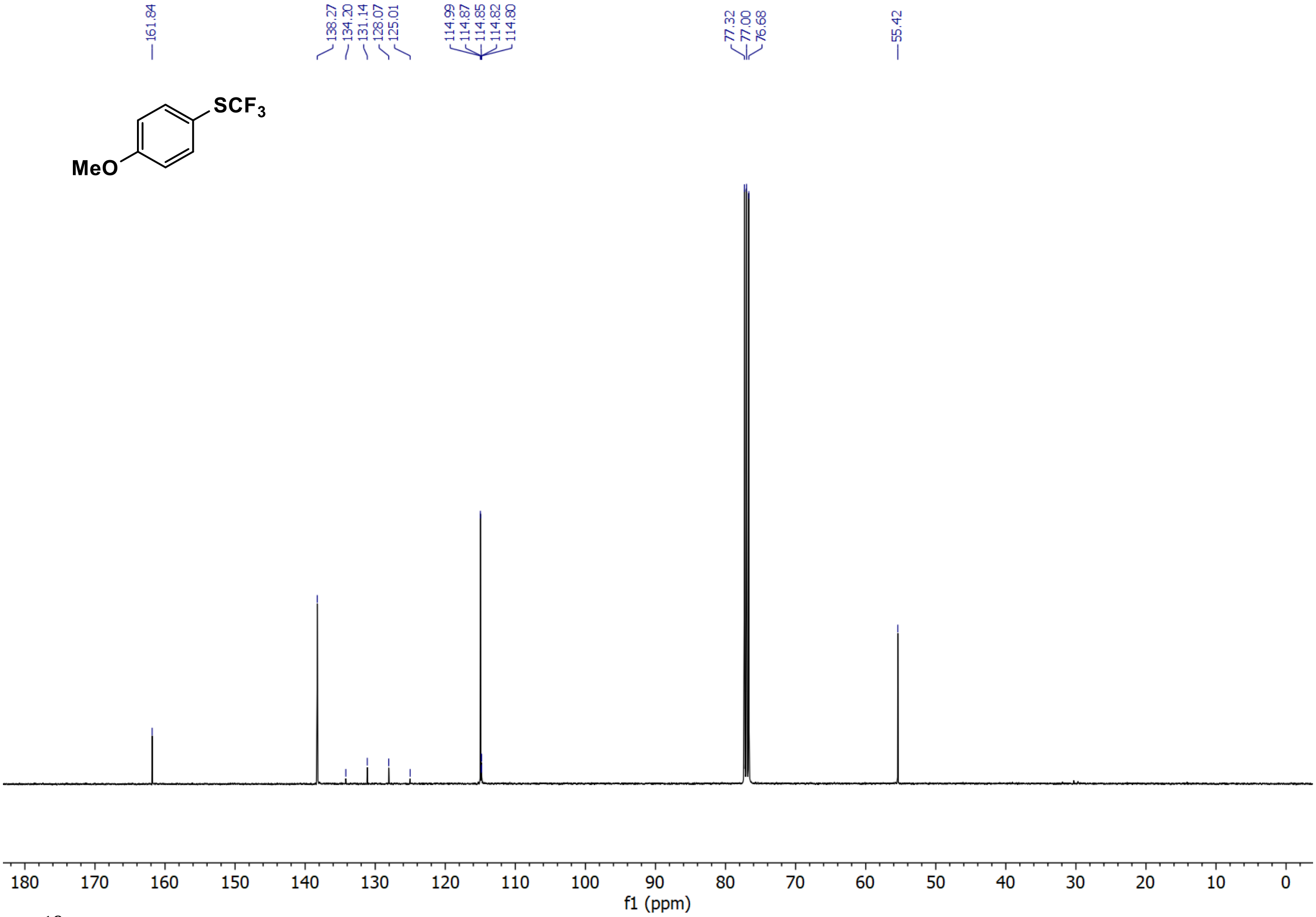

5a ^{19}F NMR (376 MHz, $CDCl_3$)

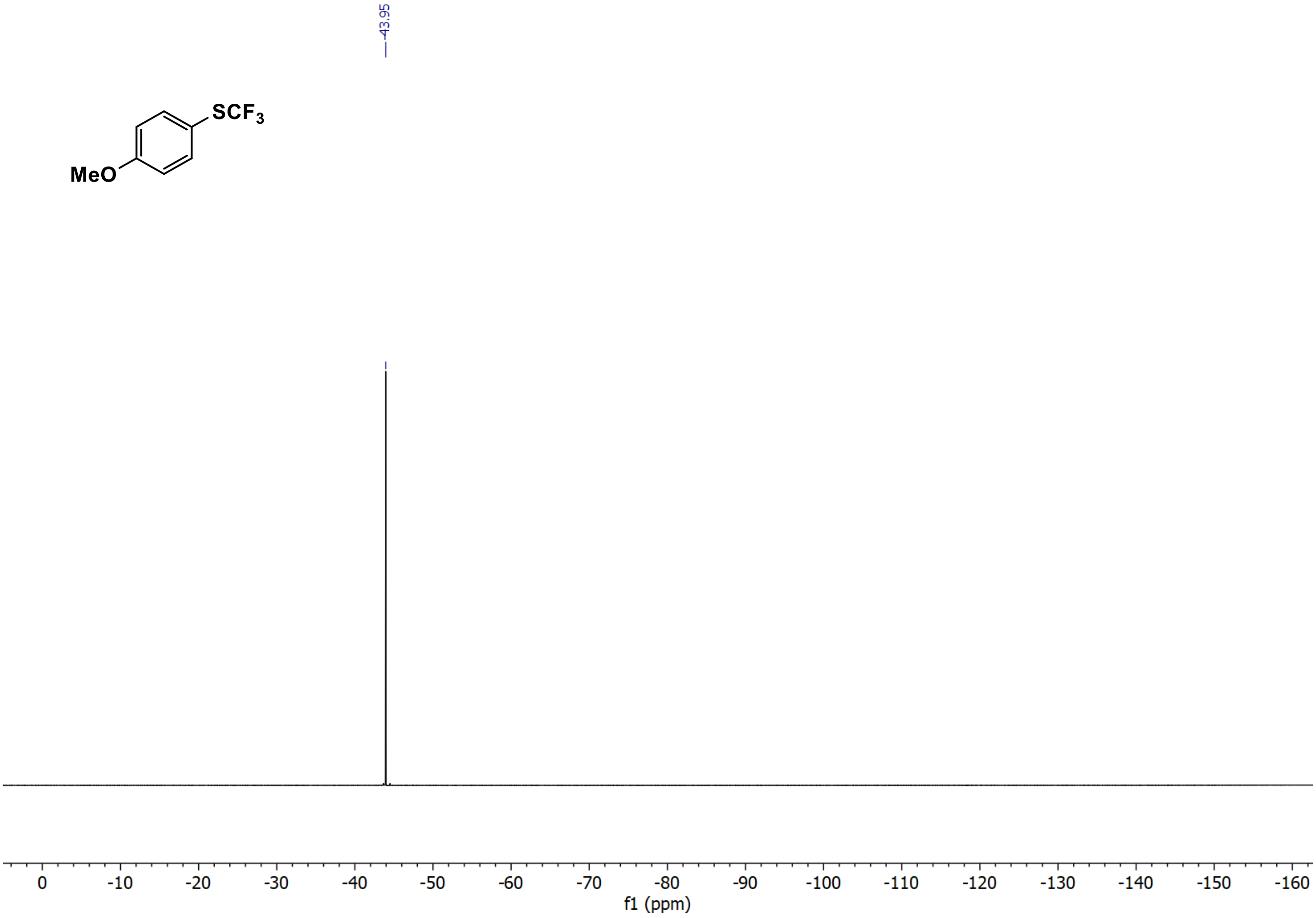

5b (13:1 mixture of regioisomers *para*-**5b** and *ortho*-**5b**) ^{1}H NMR (500 MHz, $CDCl_3$)

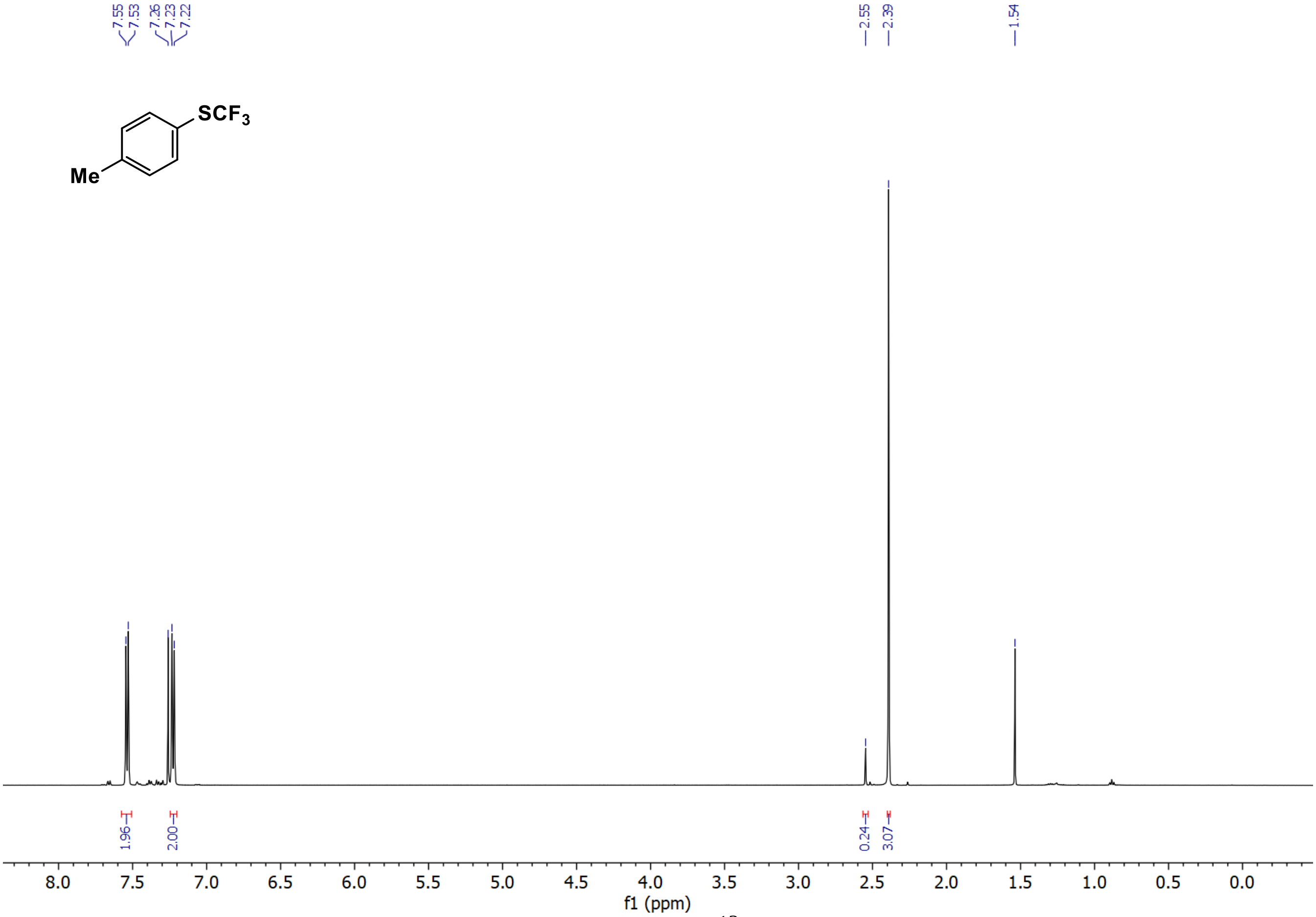

5b (13:1 mixture of regioisomers *para*-**5b** and *ortho*-**5b**) ^{13}C NMR (101 MHz, $CDCl_3$)

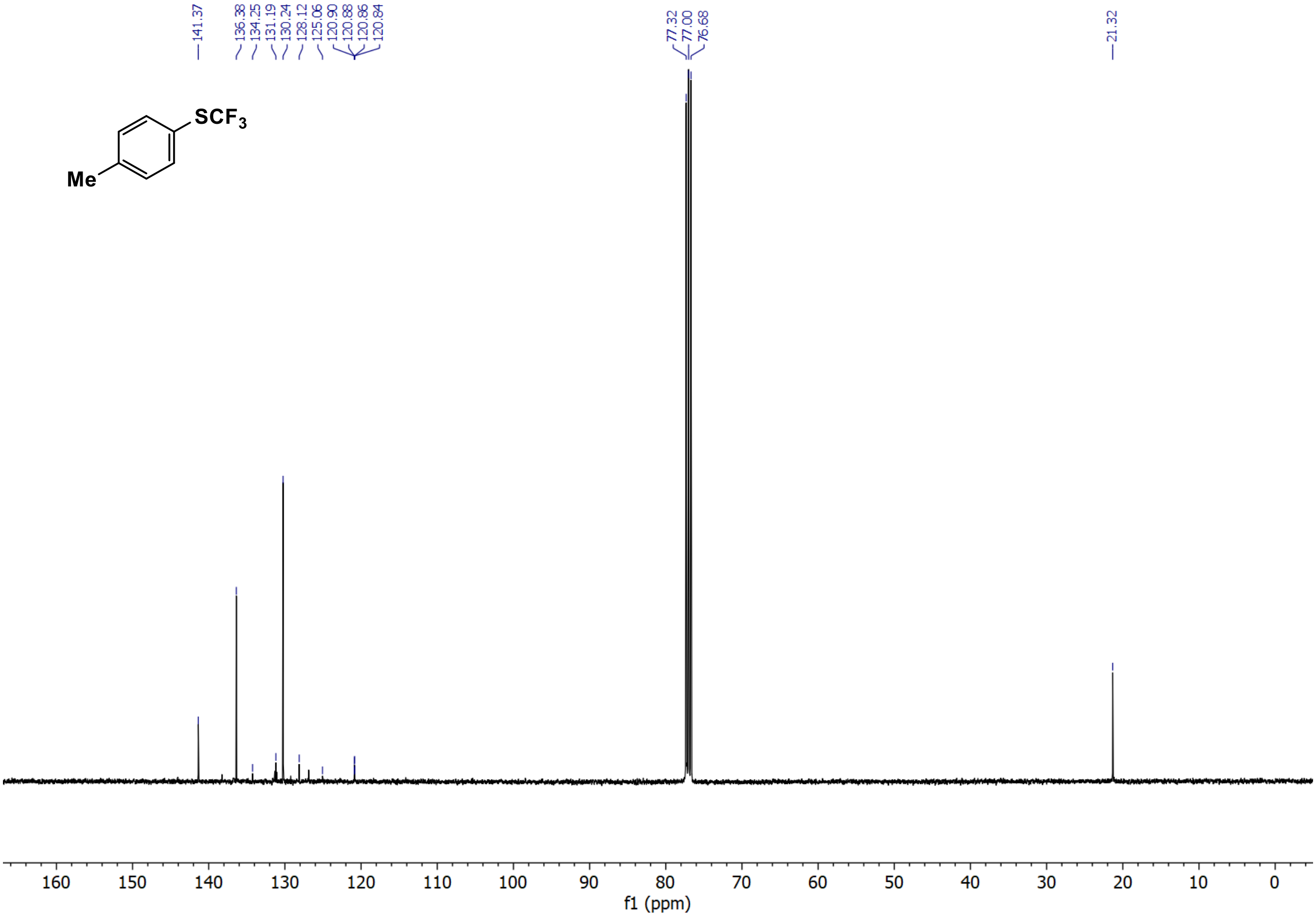

5b (13:1 mixture of regioisomers *para*-**5b** and *ortho*-**5b**) ^{19}F NMR (376 MHz, $CDCl_3$)

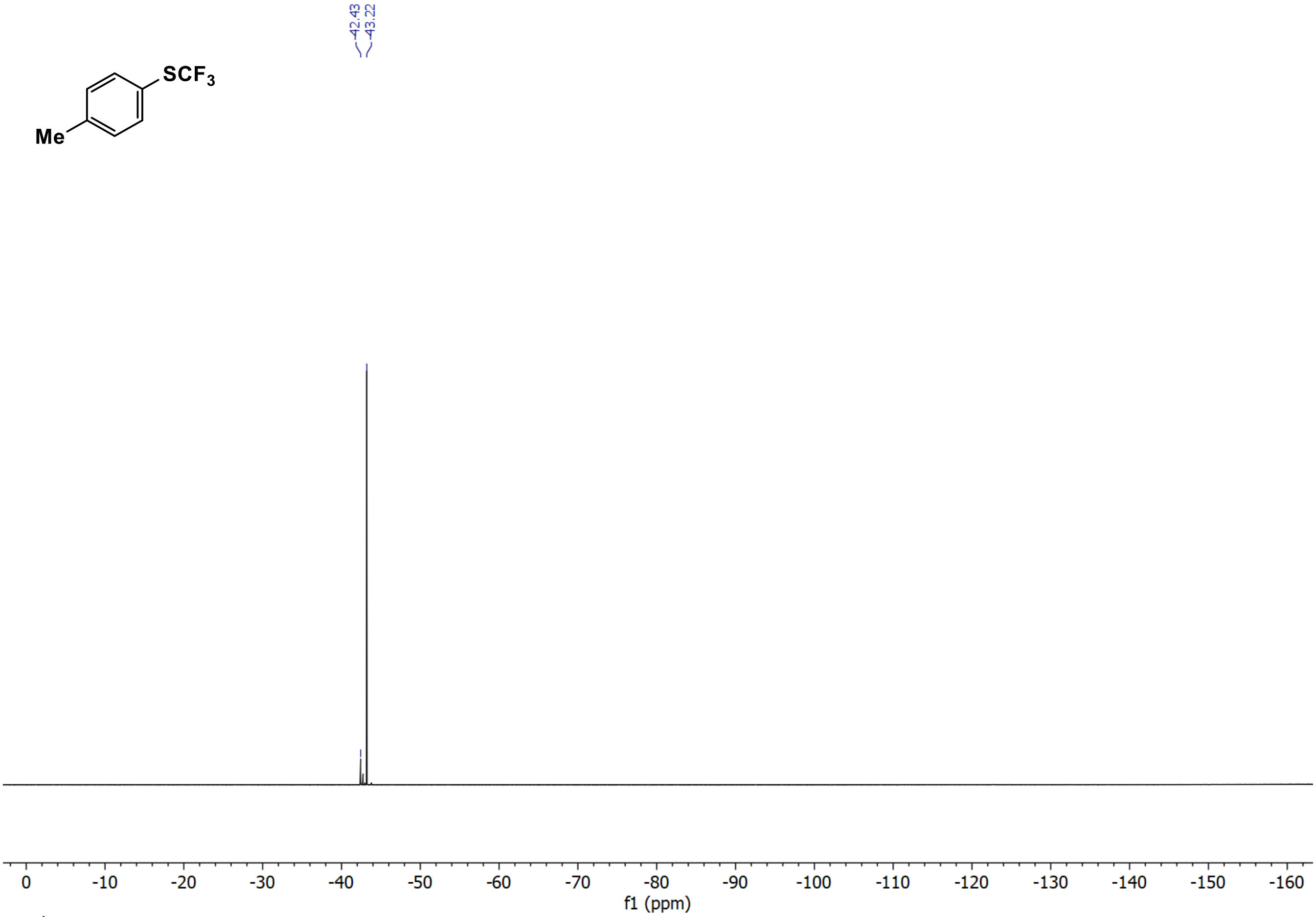

5c ^{1}H NMR (500 MHz, $CDCl_3$)

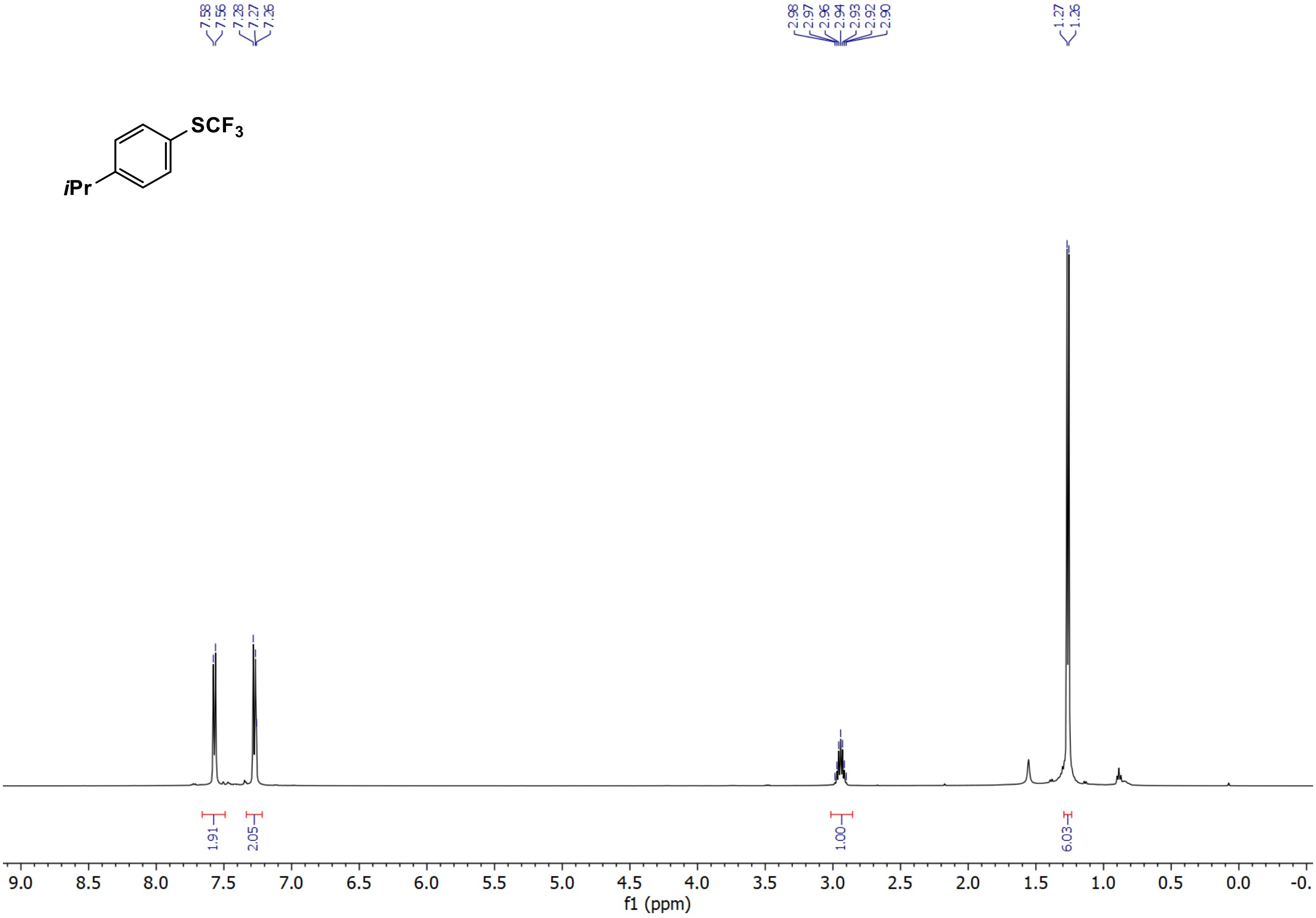

5c ^{13}C NMR (126 MHz, $CDCl_3$)

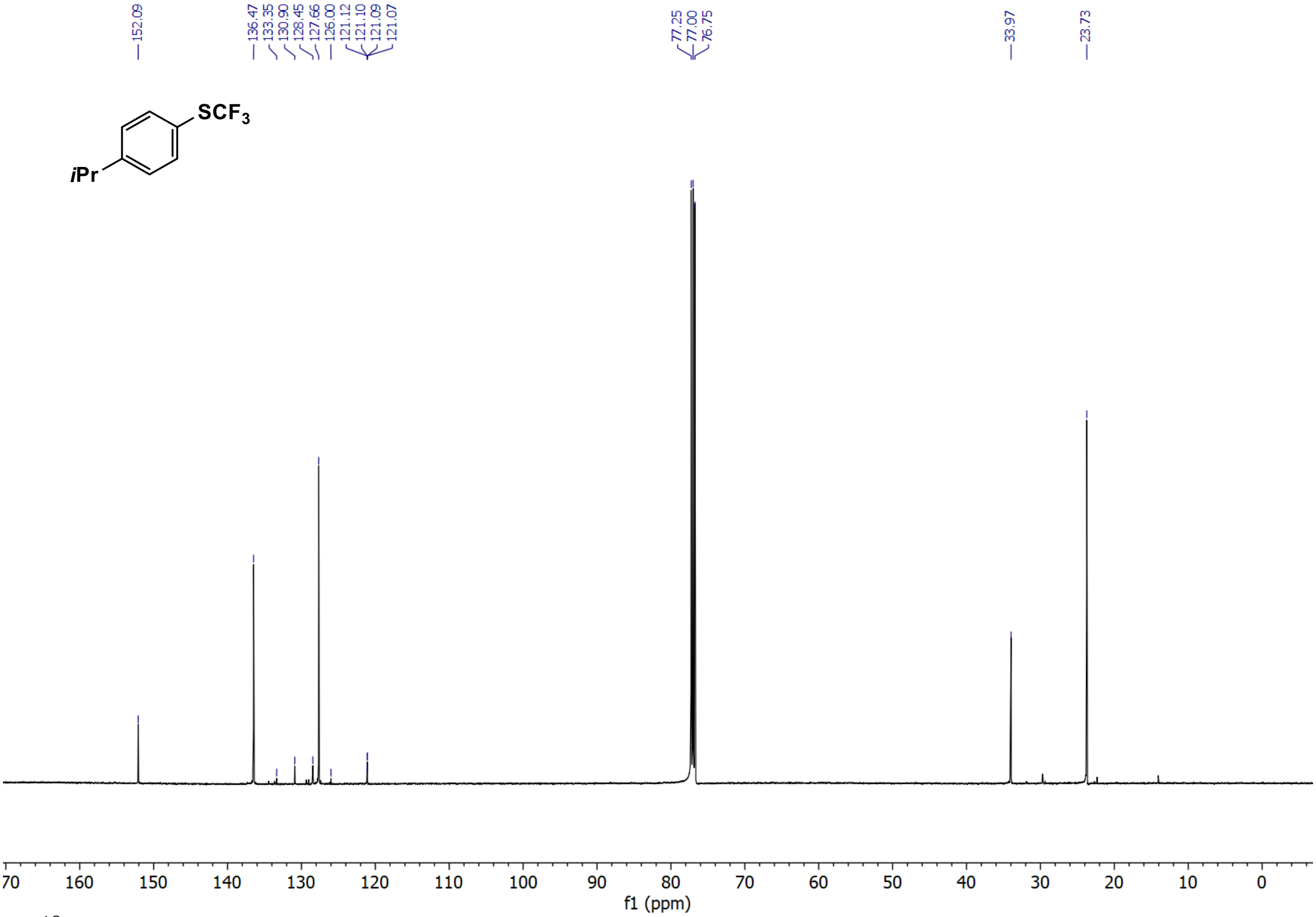

5c ^{19}F NMR (376 MHz, $CDCl_3$)

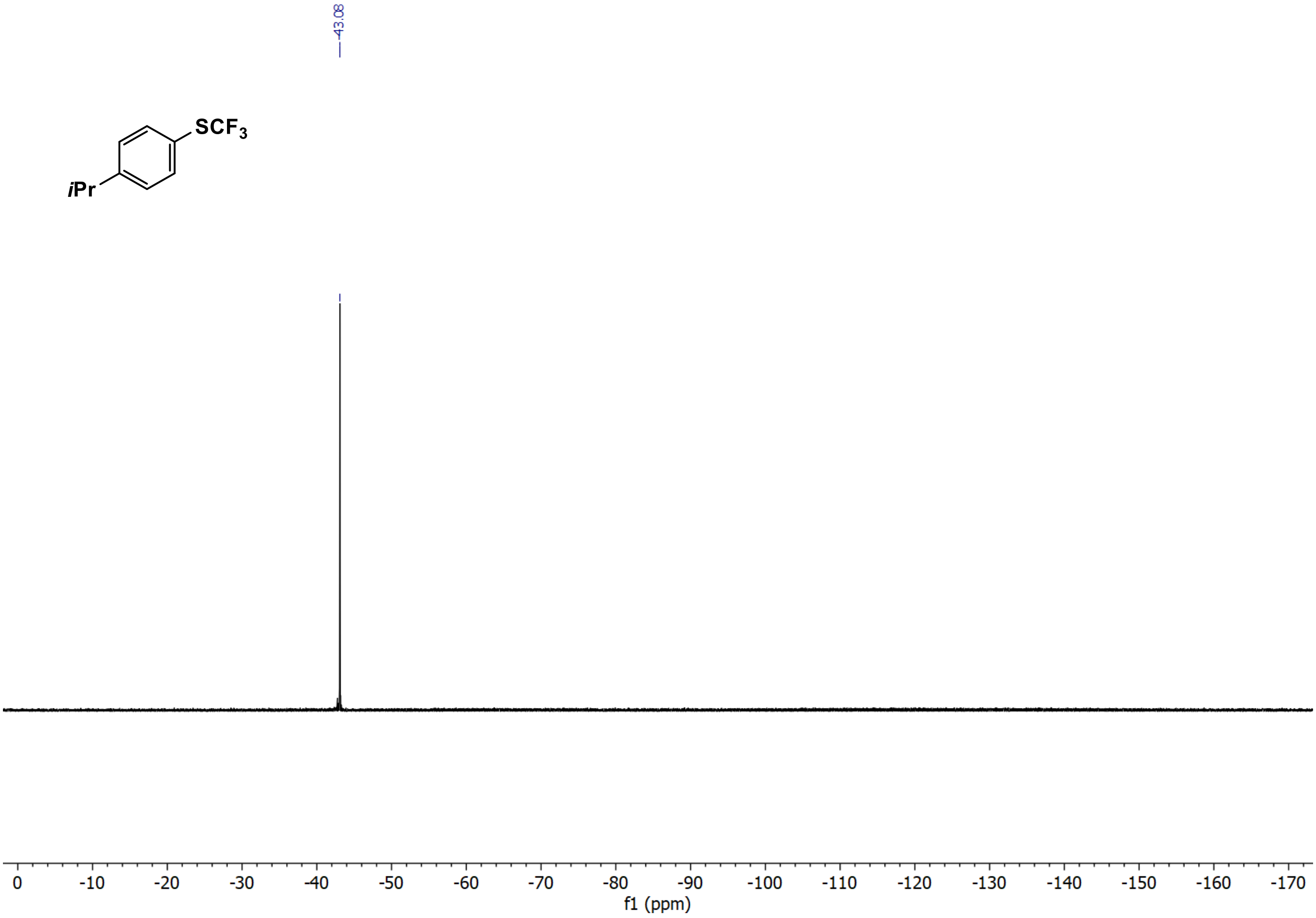

5d ^{1}H NMR (400 MHz, $CDCl_3$)

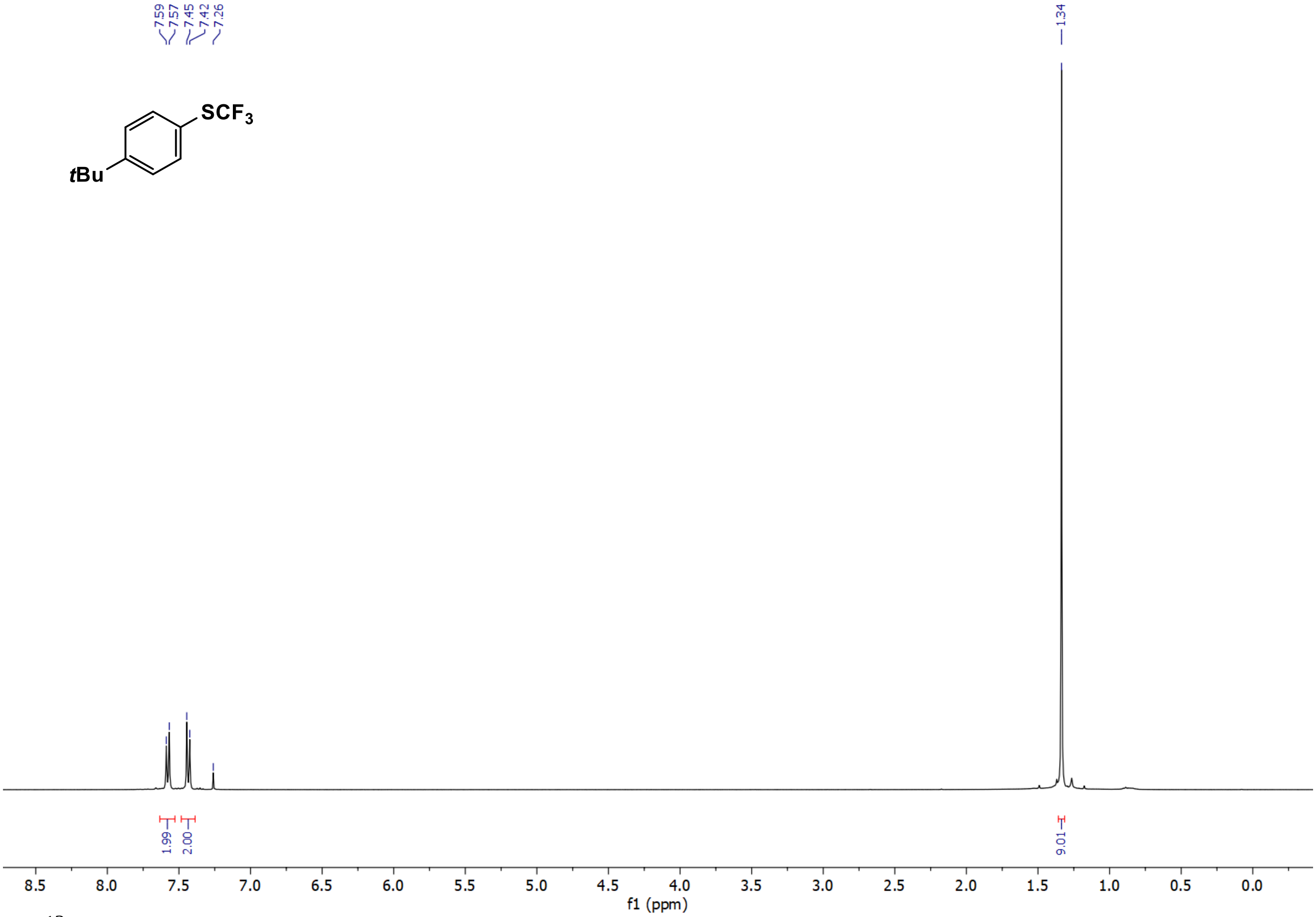

5d ^{13}C NMR (101 MHz, $CDCl_3$)

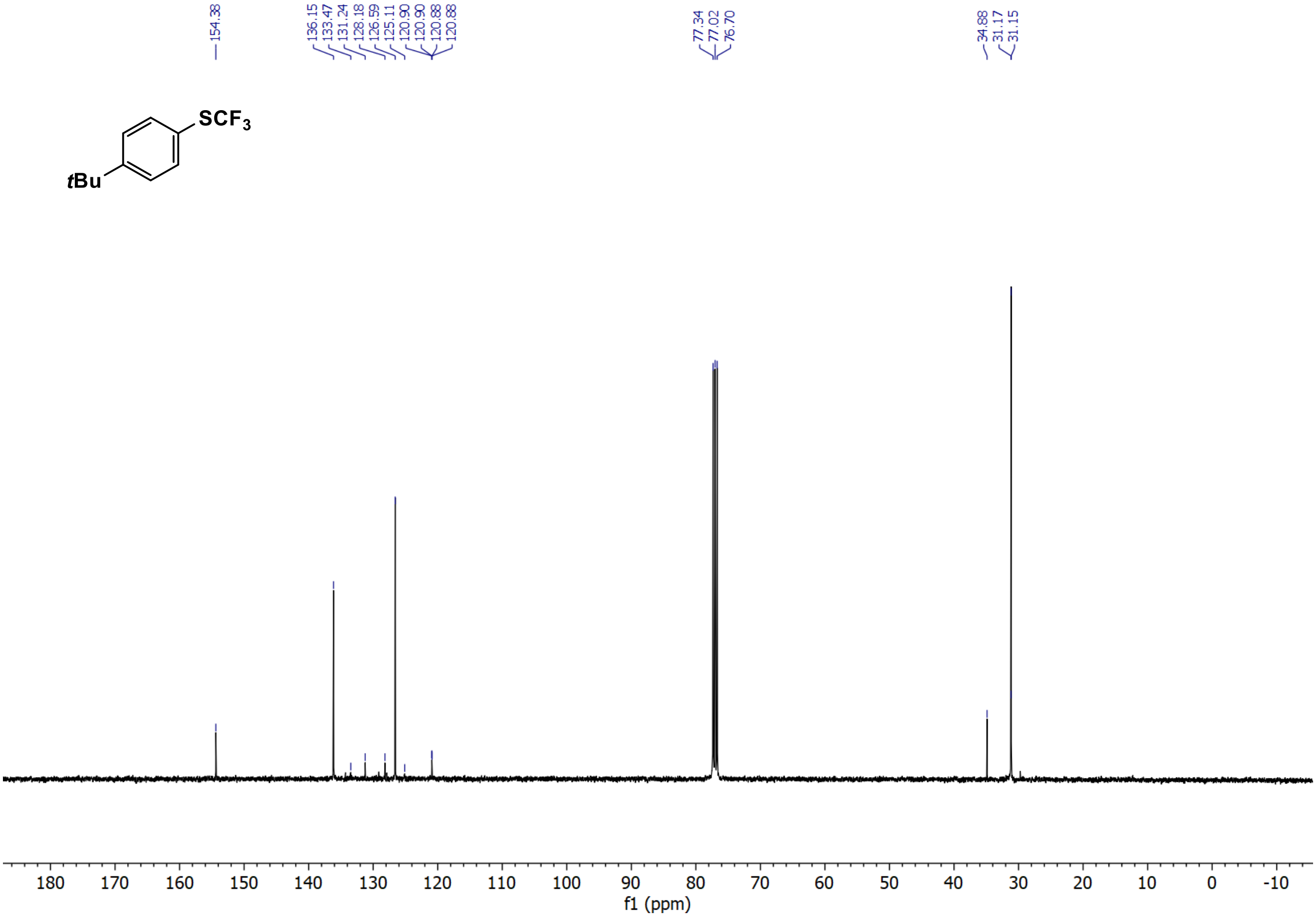

5d ^{19}F NMR (376 MHz, $CDCl_3$)

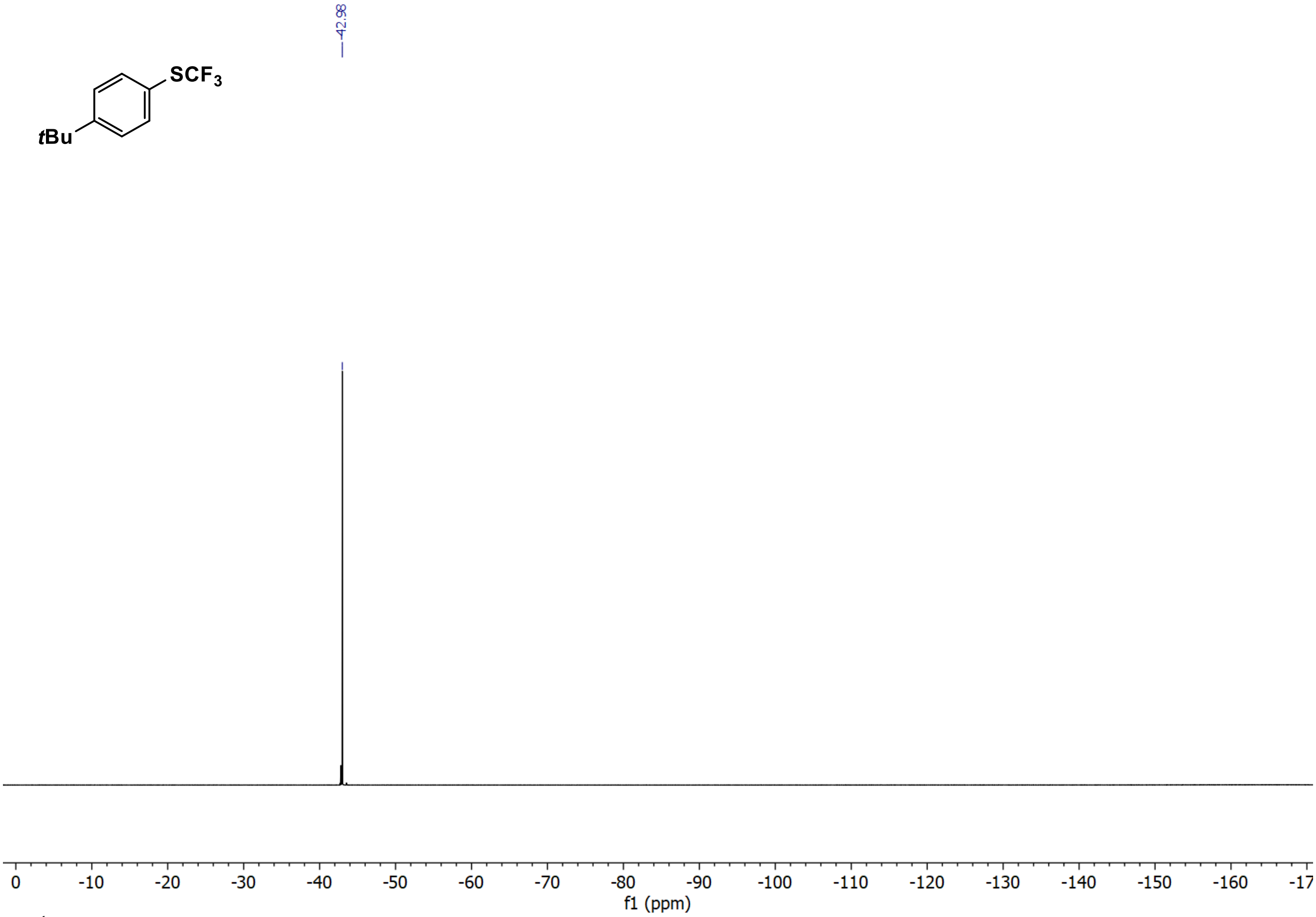

5e ^{1}H NMR (400 MHz, $CDCl_3$)

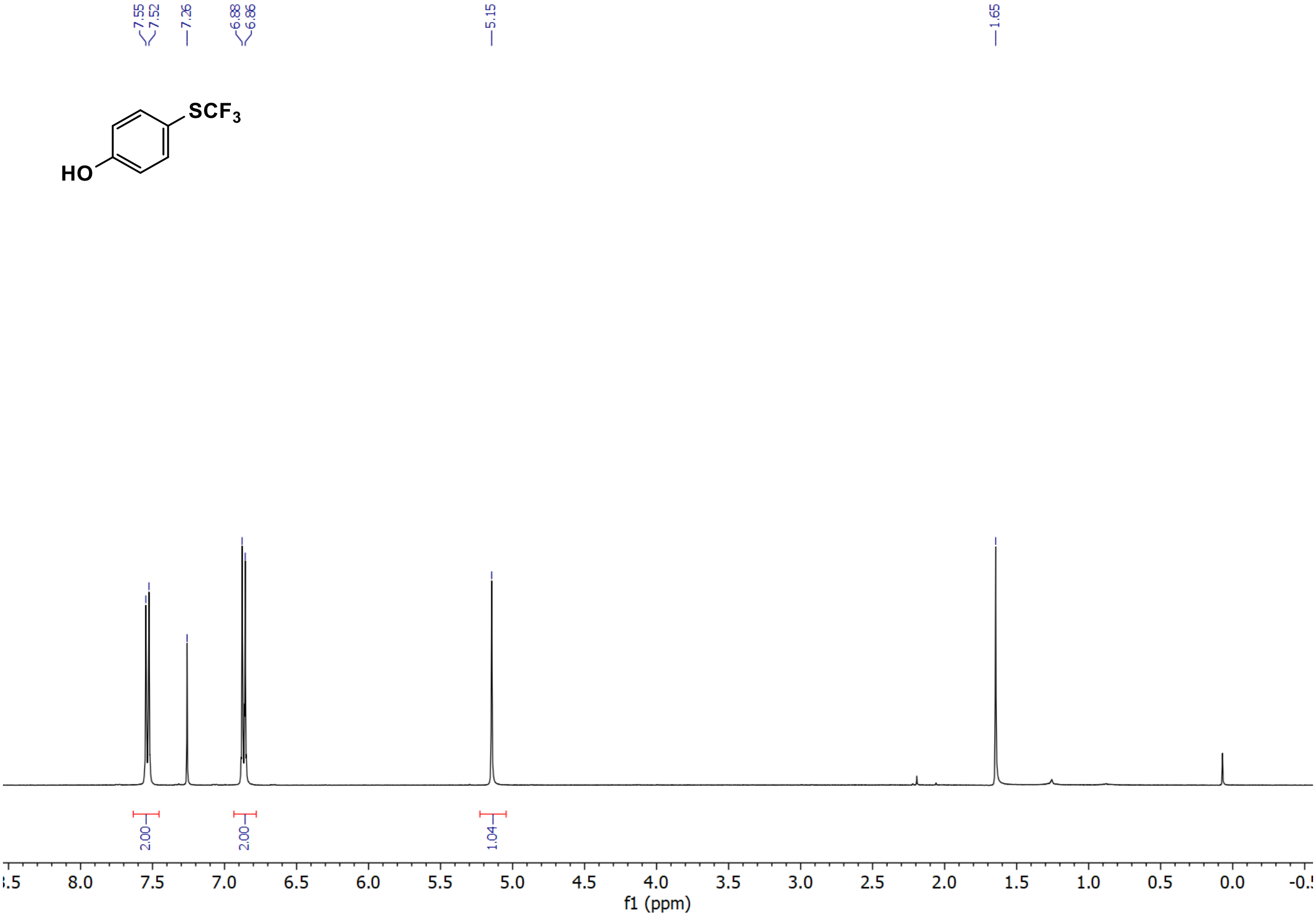

5e ^{13}C NMR (126 MHz, $CDCl_3$)

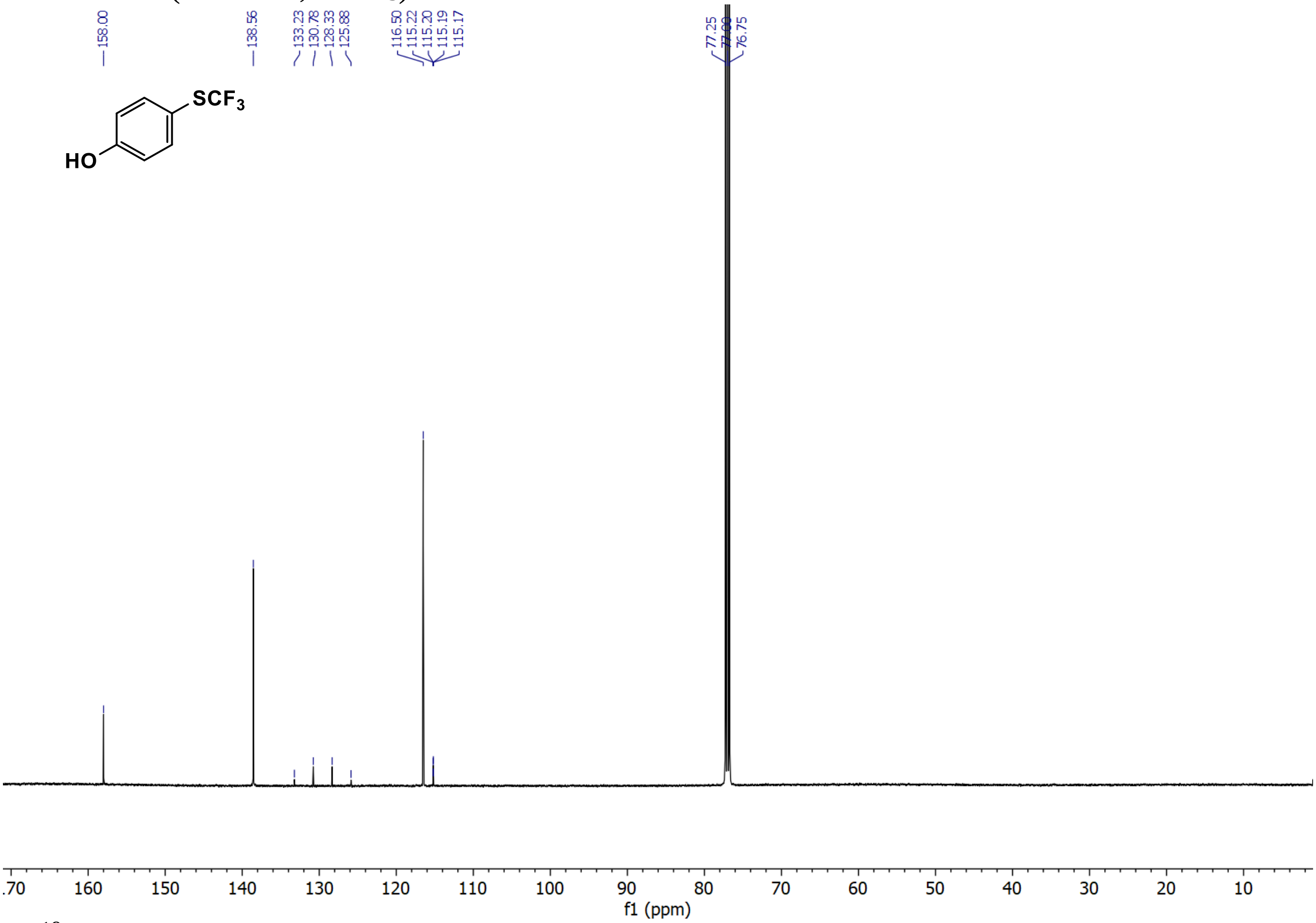

5e ^{19}F NMR (376 MHz, $CDCl_3$)

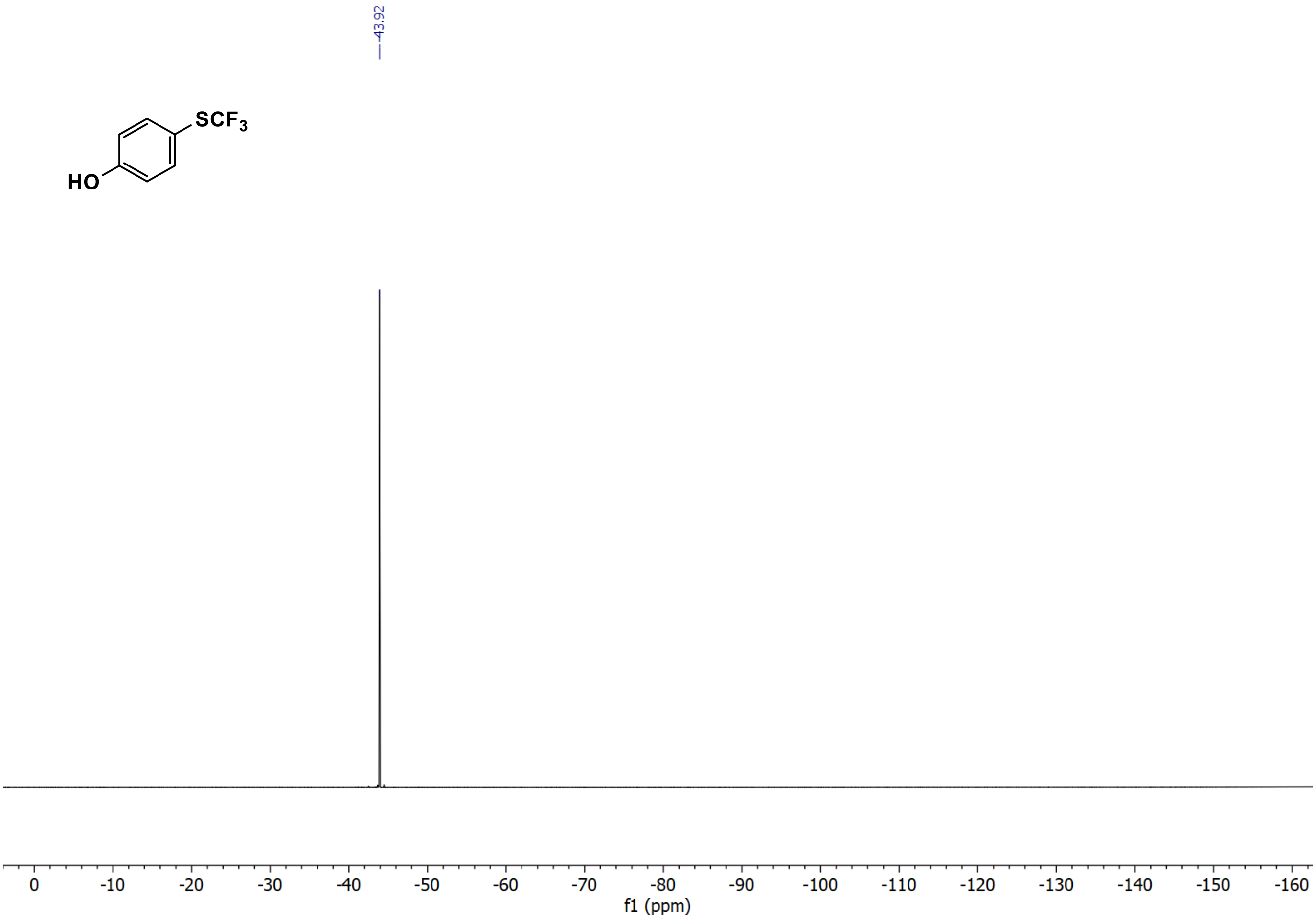

5g ^{1}H NMR (400 MHz, $CDCl_3$)

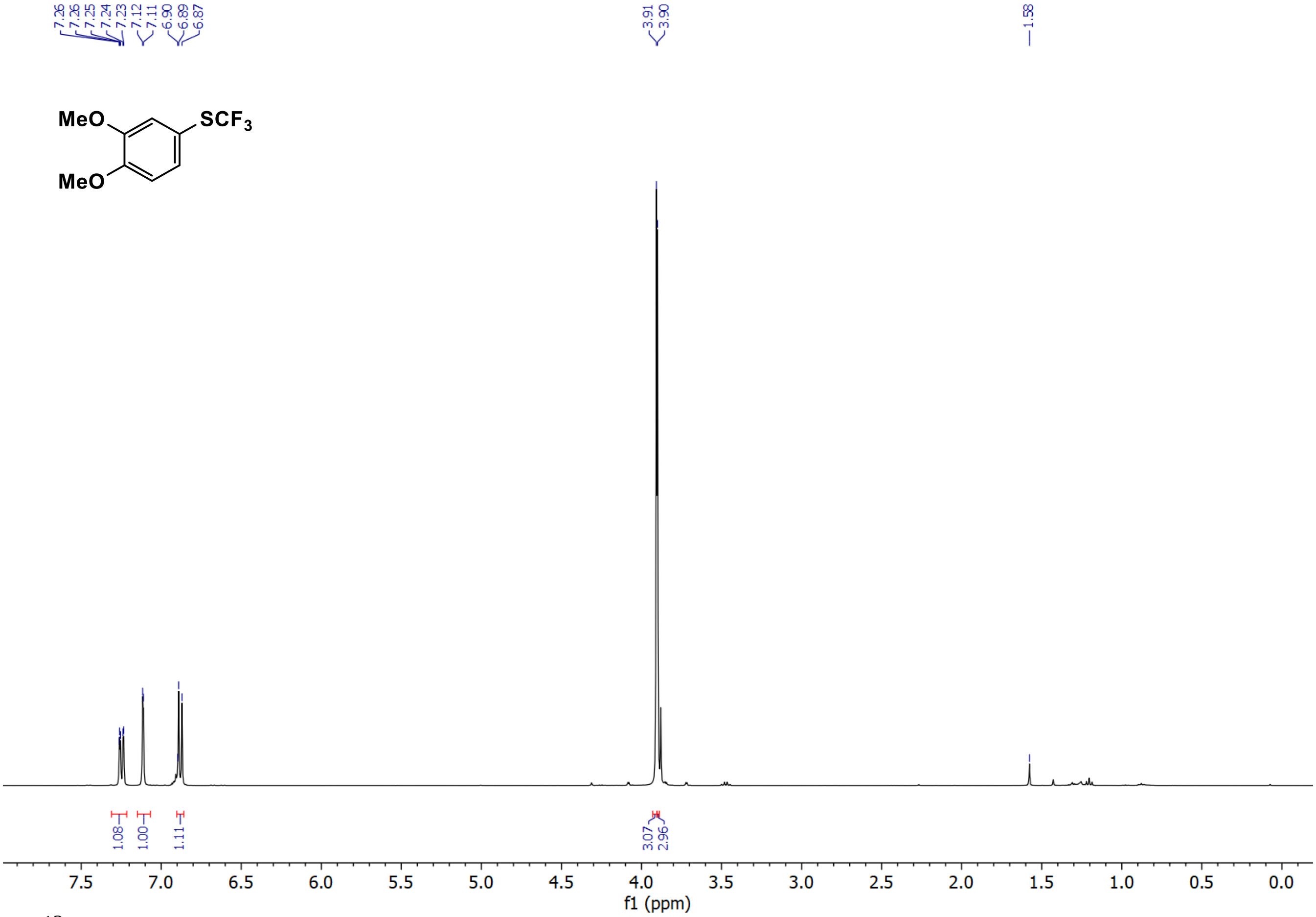

5g ^{13}C NMR (101 MHz, $CDCl_3$)

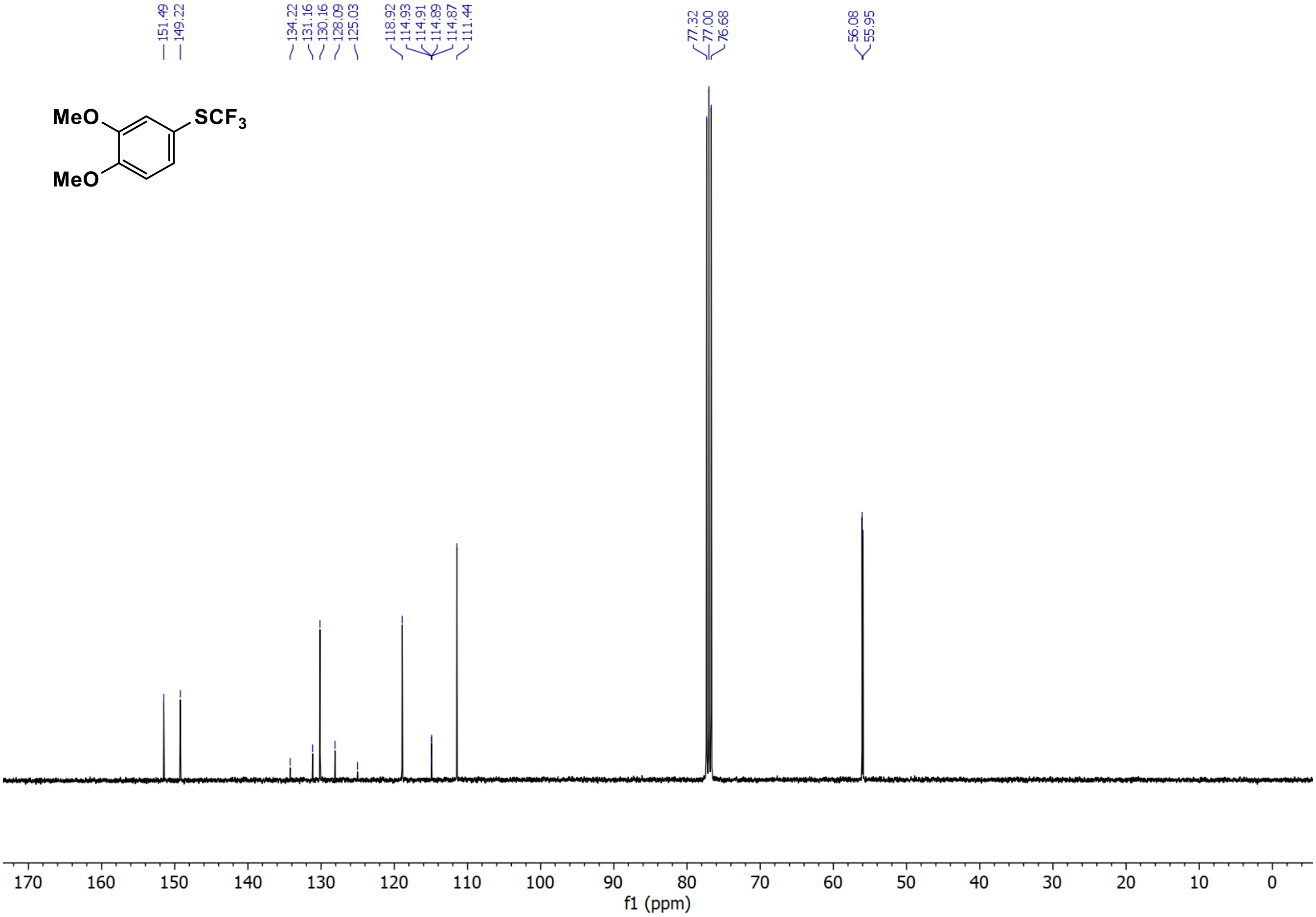

5g ^{19}F NMR (376 MHz, $CDCl_3$)

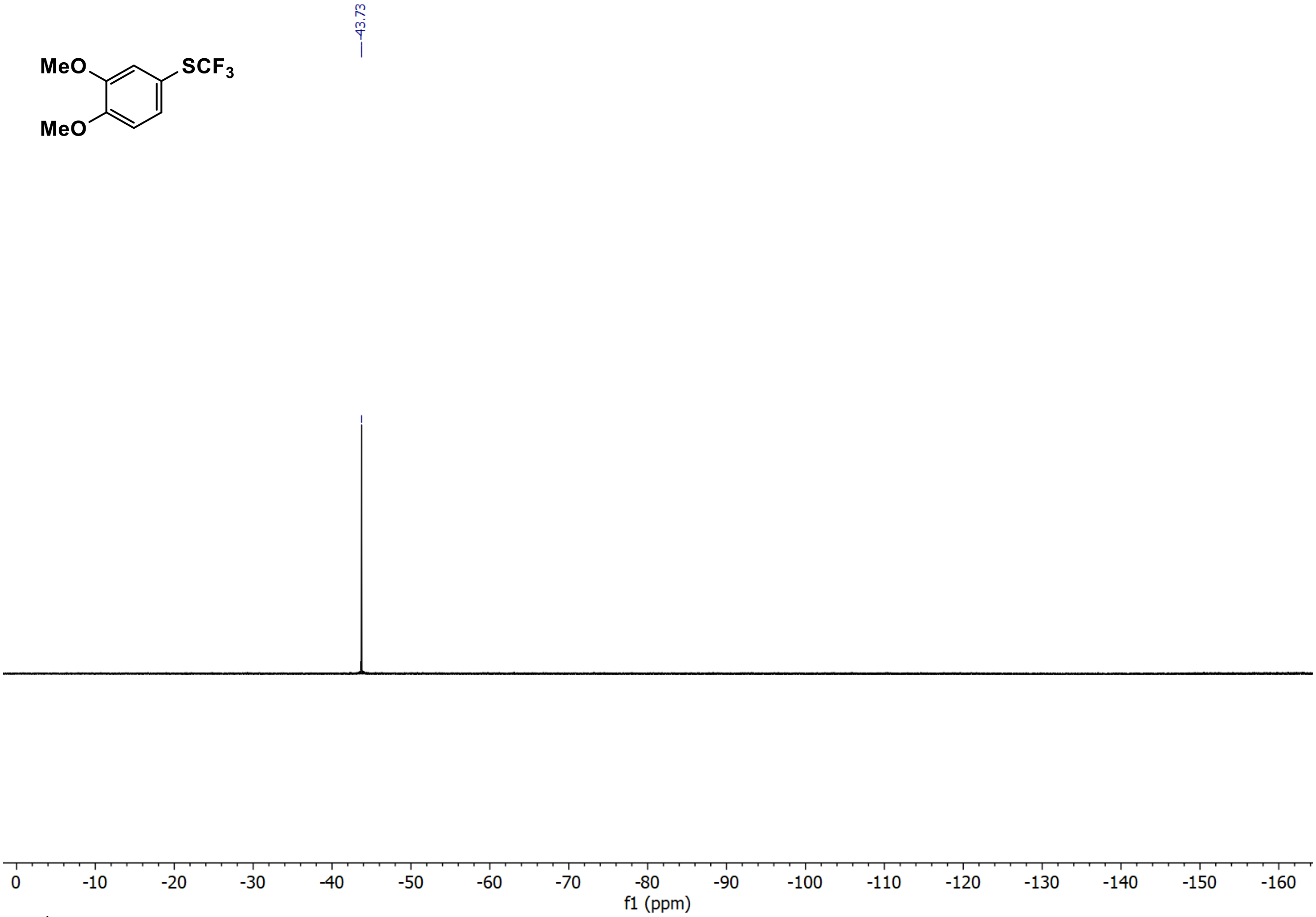

5h ^{1}H NMR (400 MHz, $CDCl_3$)

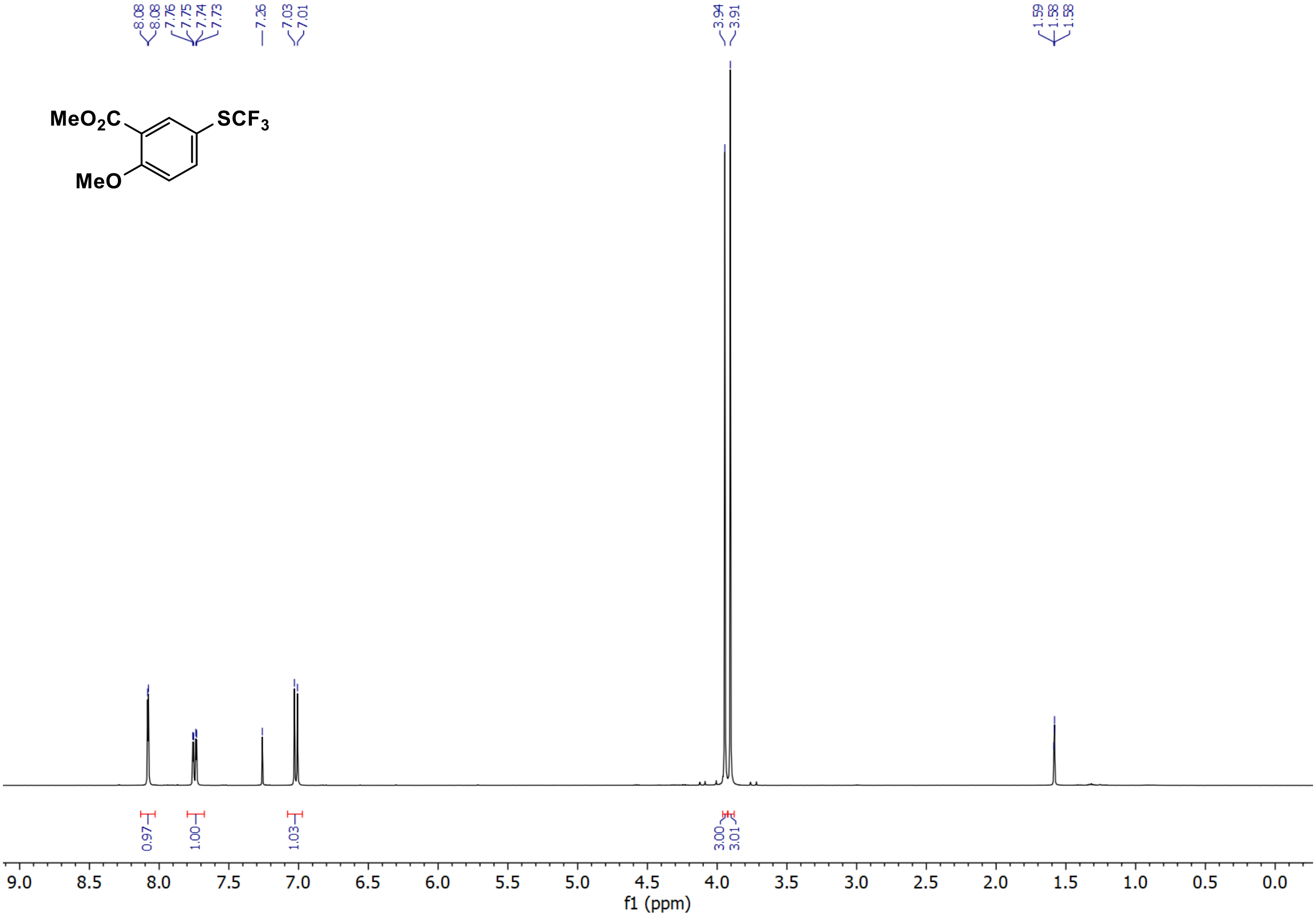

5h ^{13}C NMR (101 MHz, $CDCl_3$)

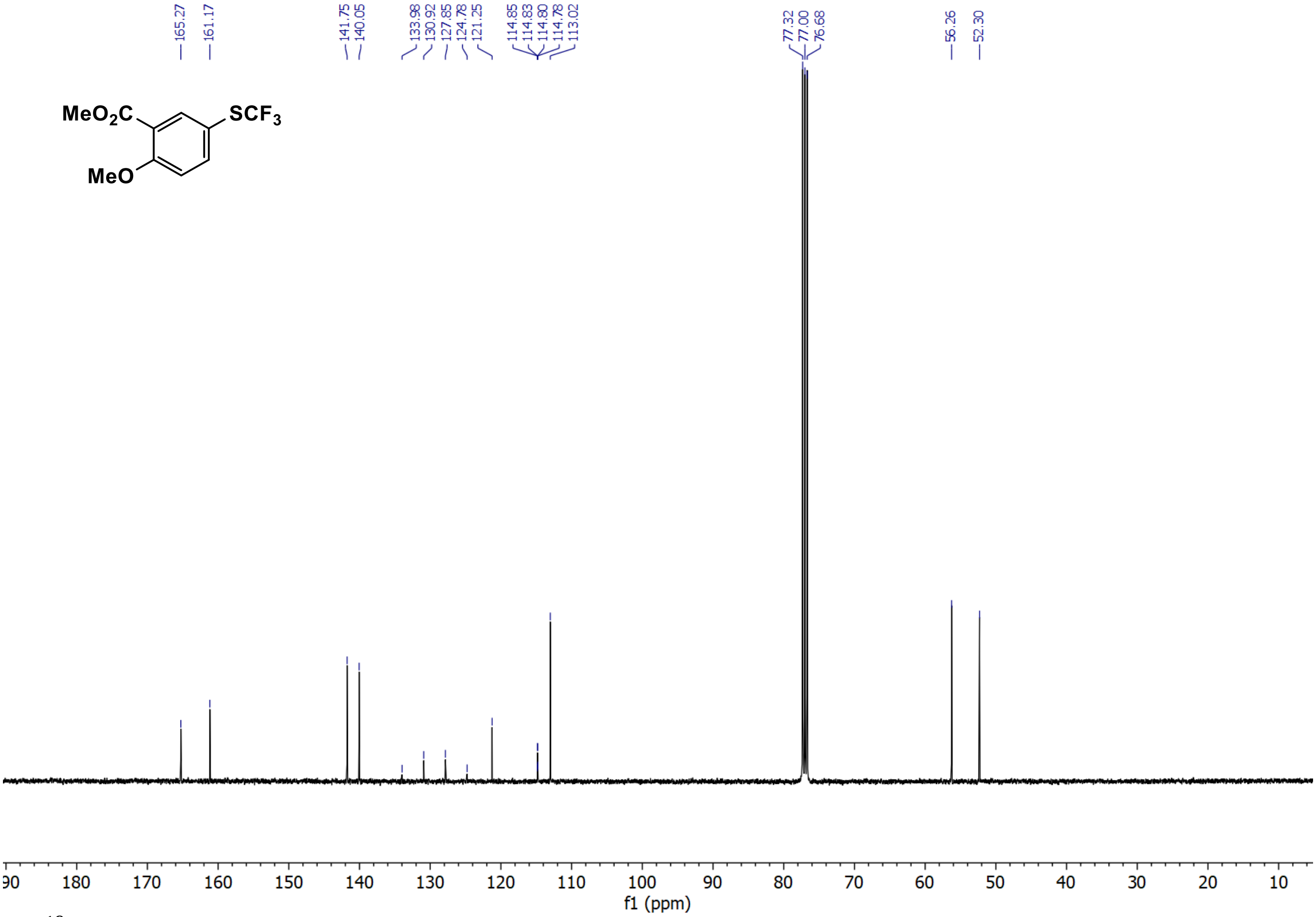

5h ^{19}F NMR (376 MHz, $CDCl_3$)

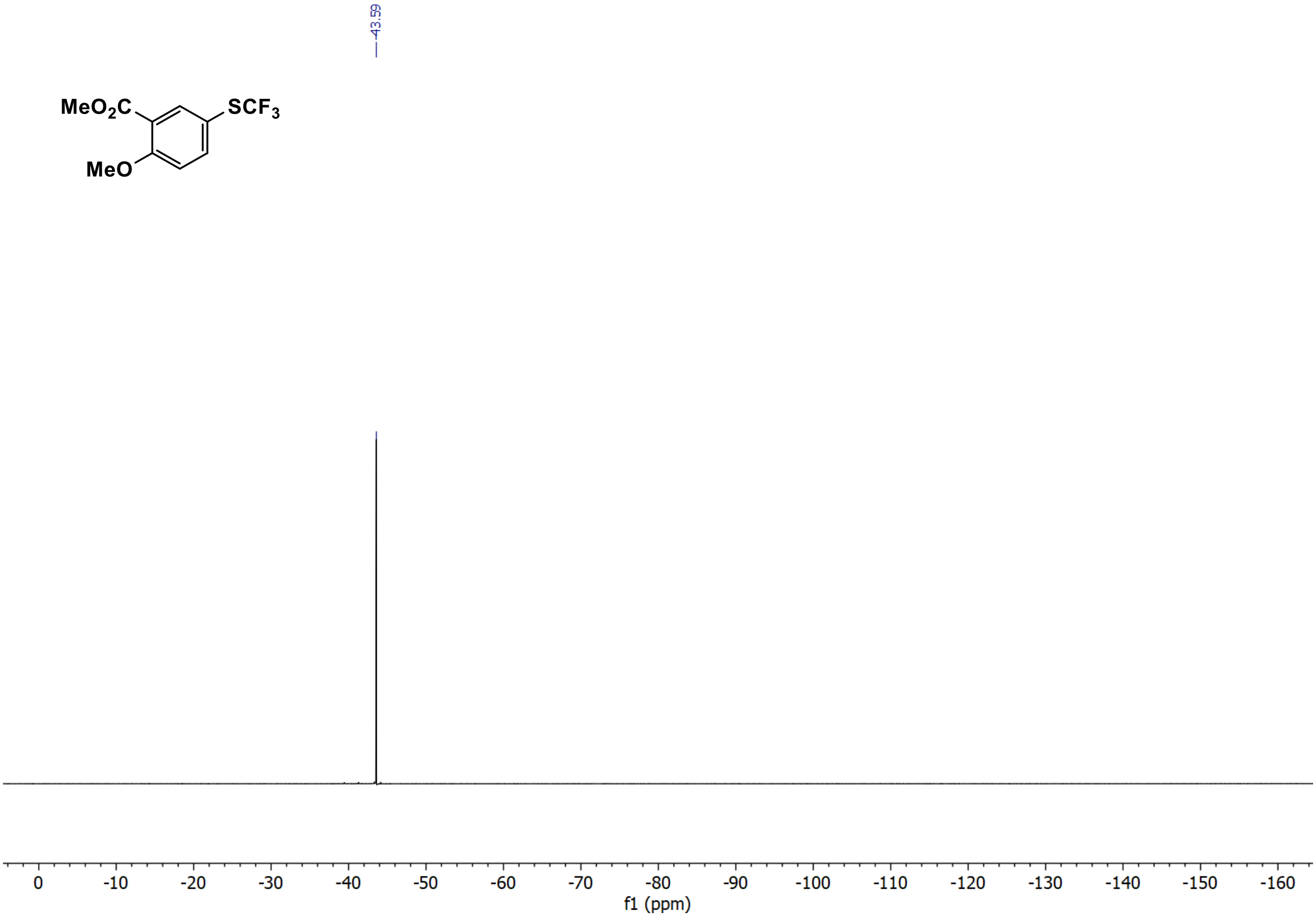

5i ^{1}H NMR (400 MHz, $CDCl_3$)

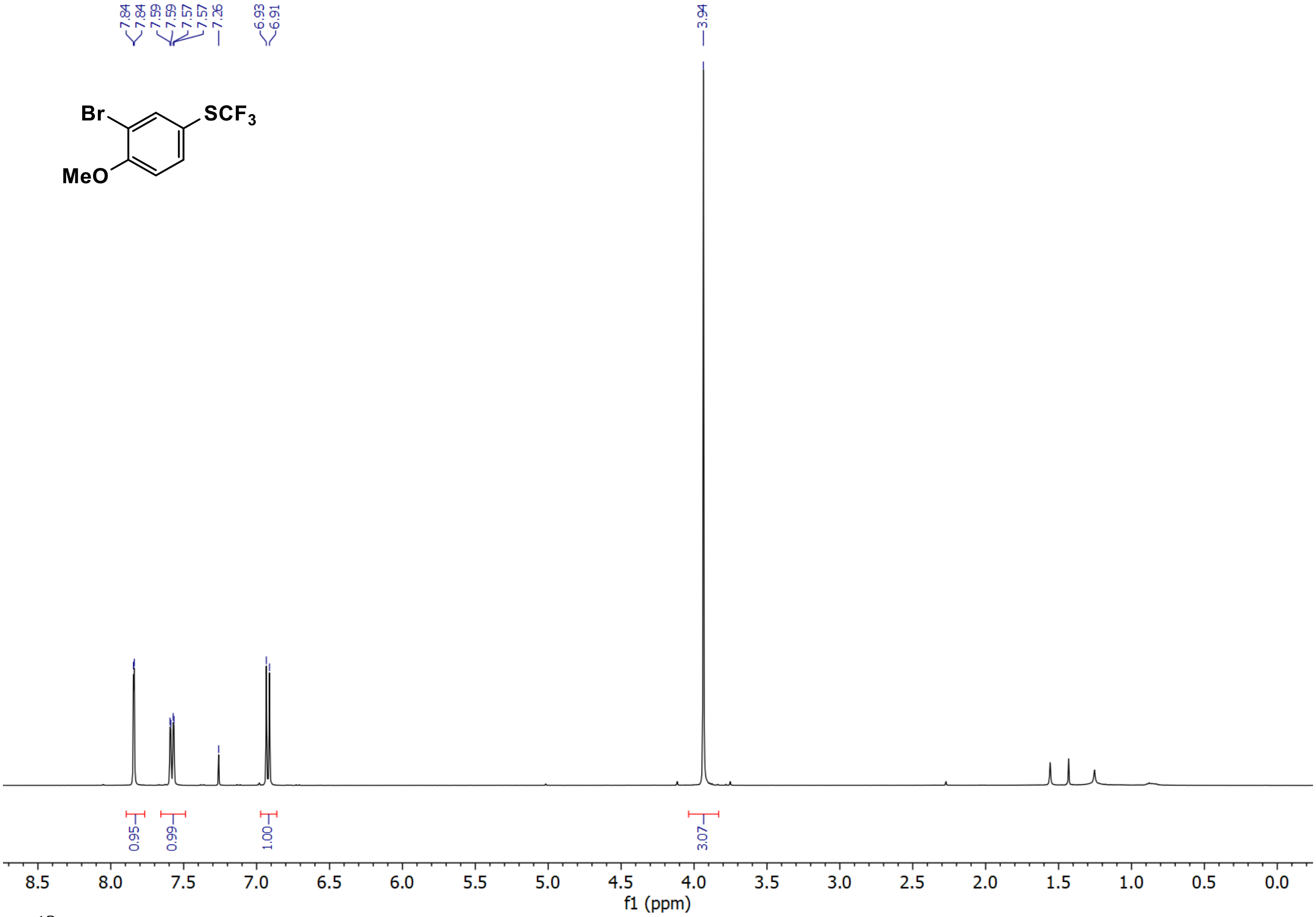

5i ^{13}C NMR (126 MHz, $CDCl_3$)

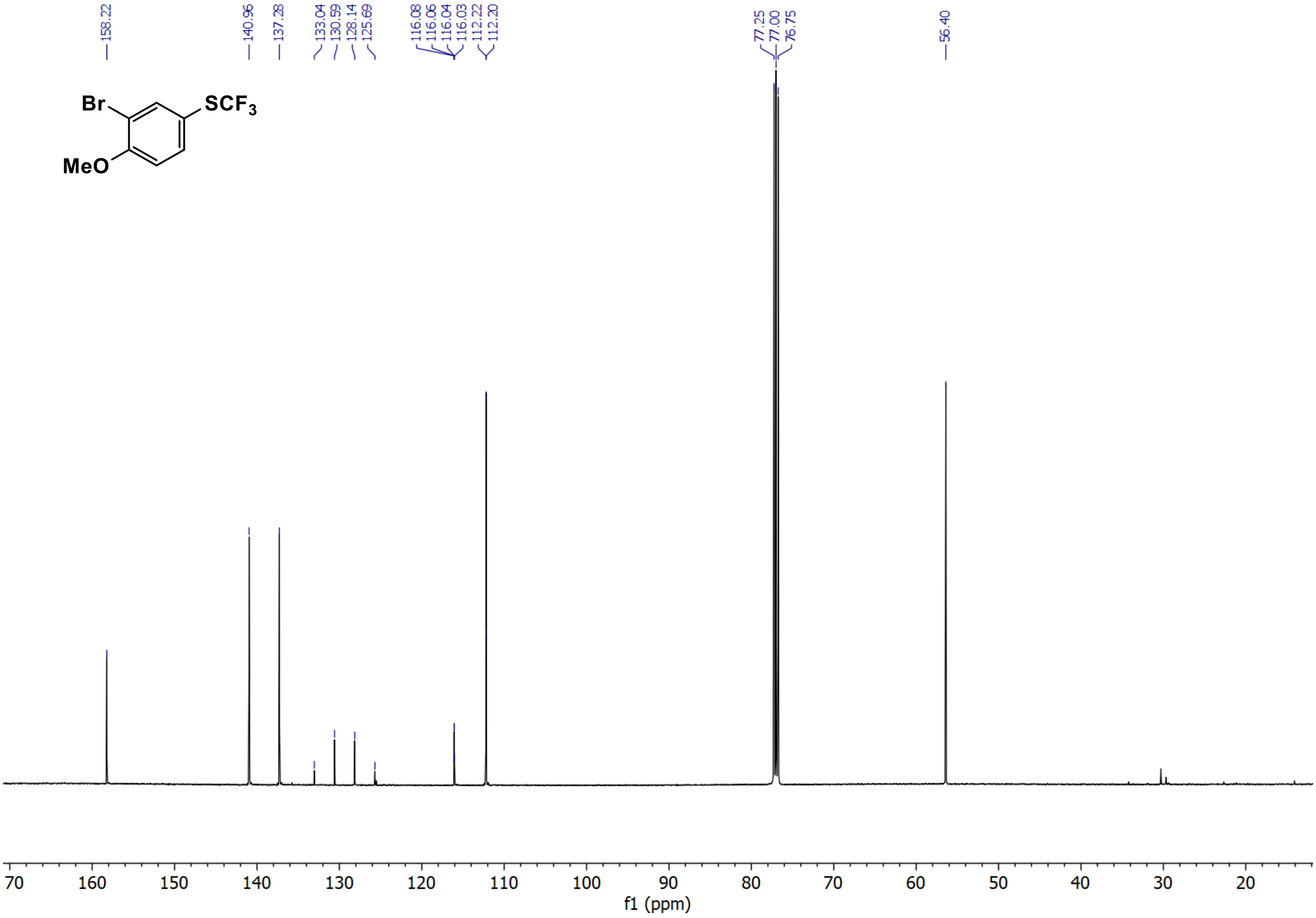

5i ^{19}F NMR (376 MHz, $CDCl_3$)

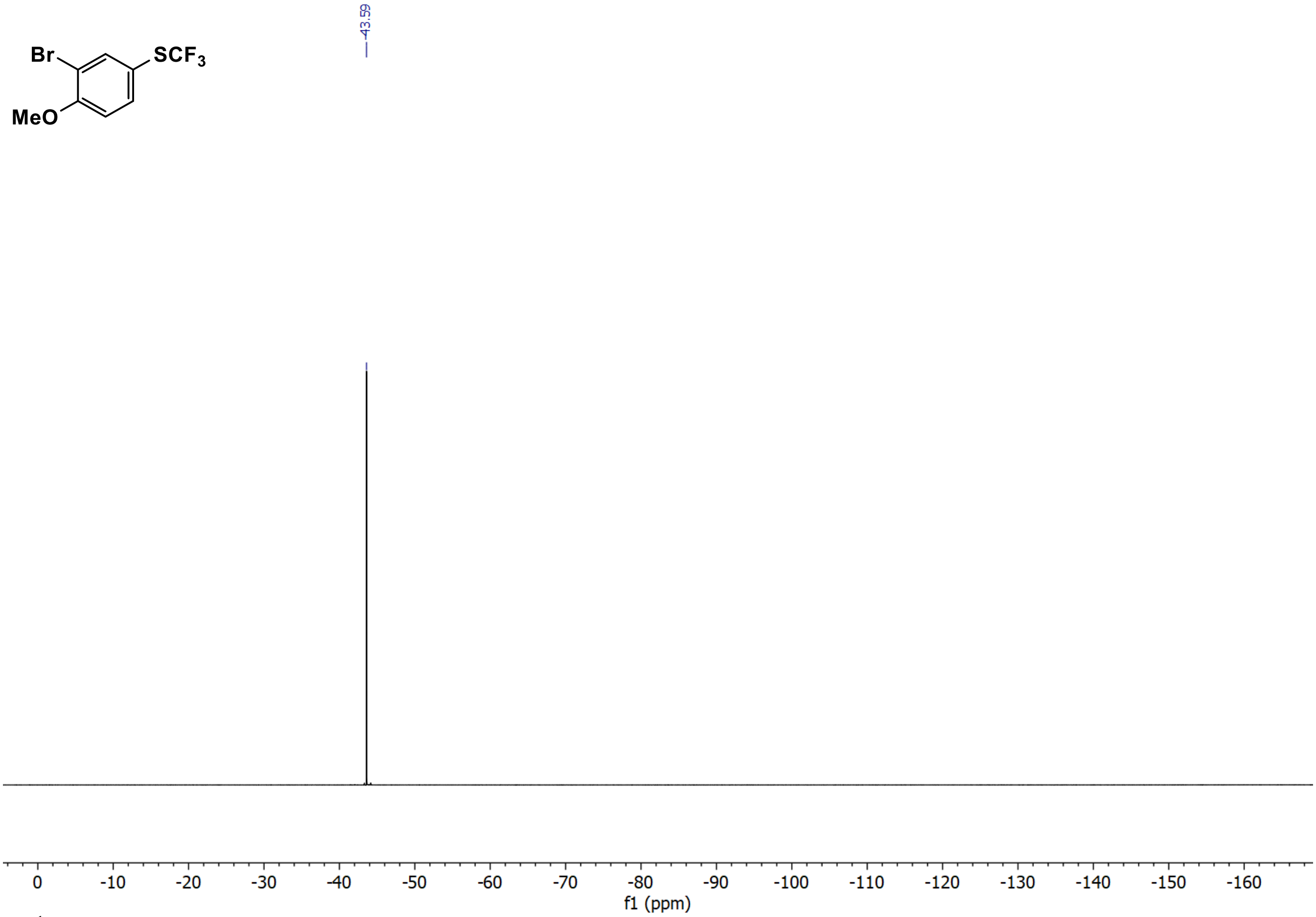

5j ^{1}H NMR (400 MHz, $CDCl_3$)

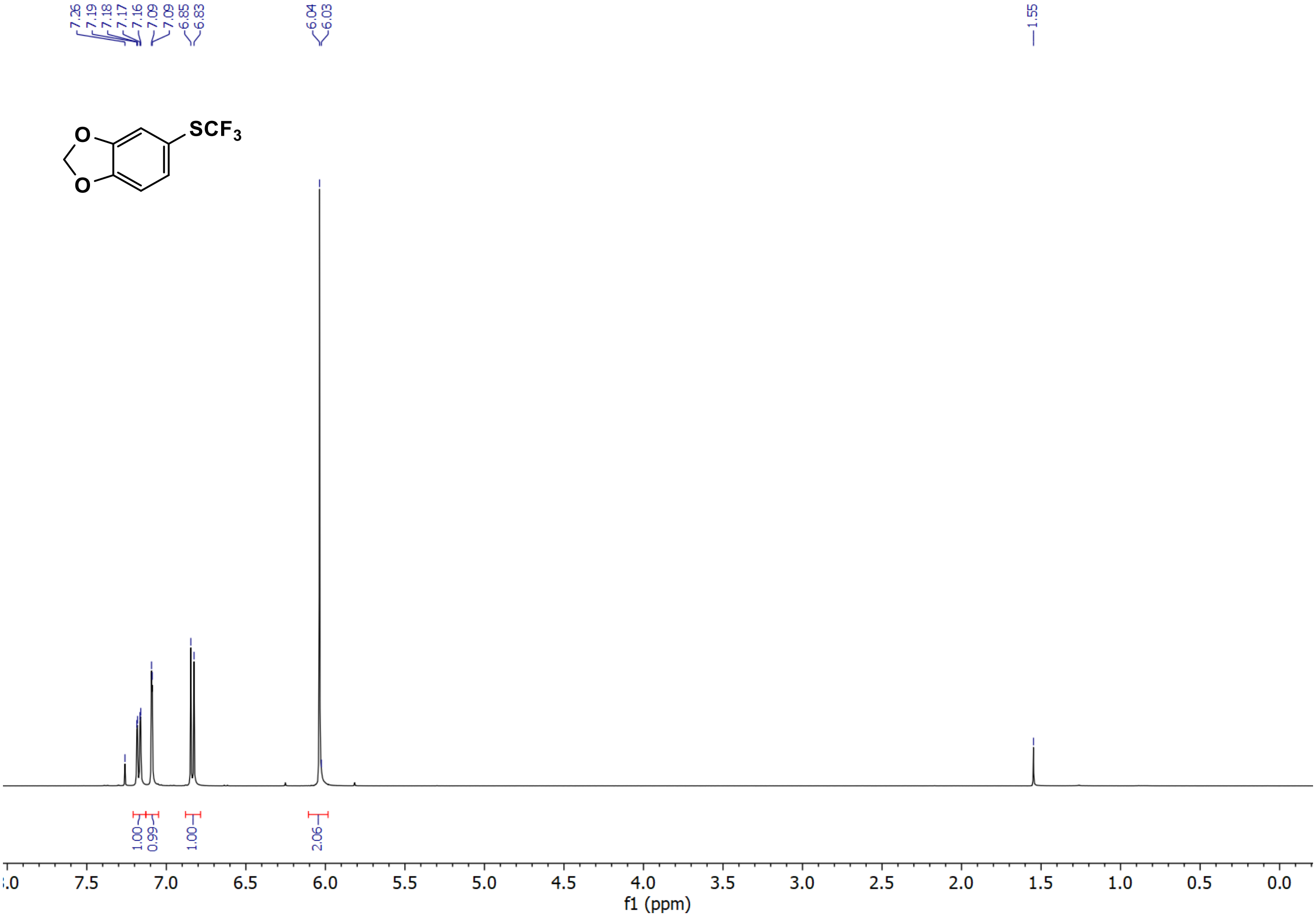

5j ^{13}C NMR (101 MHz, $CDCl_3$)

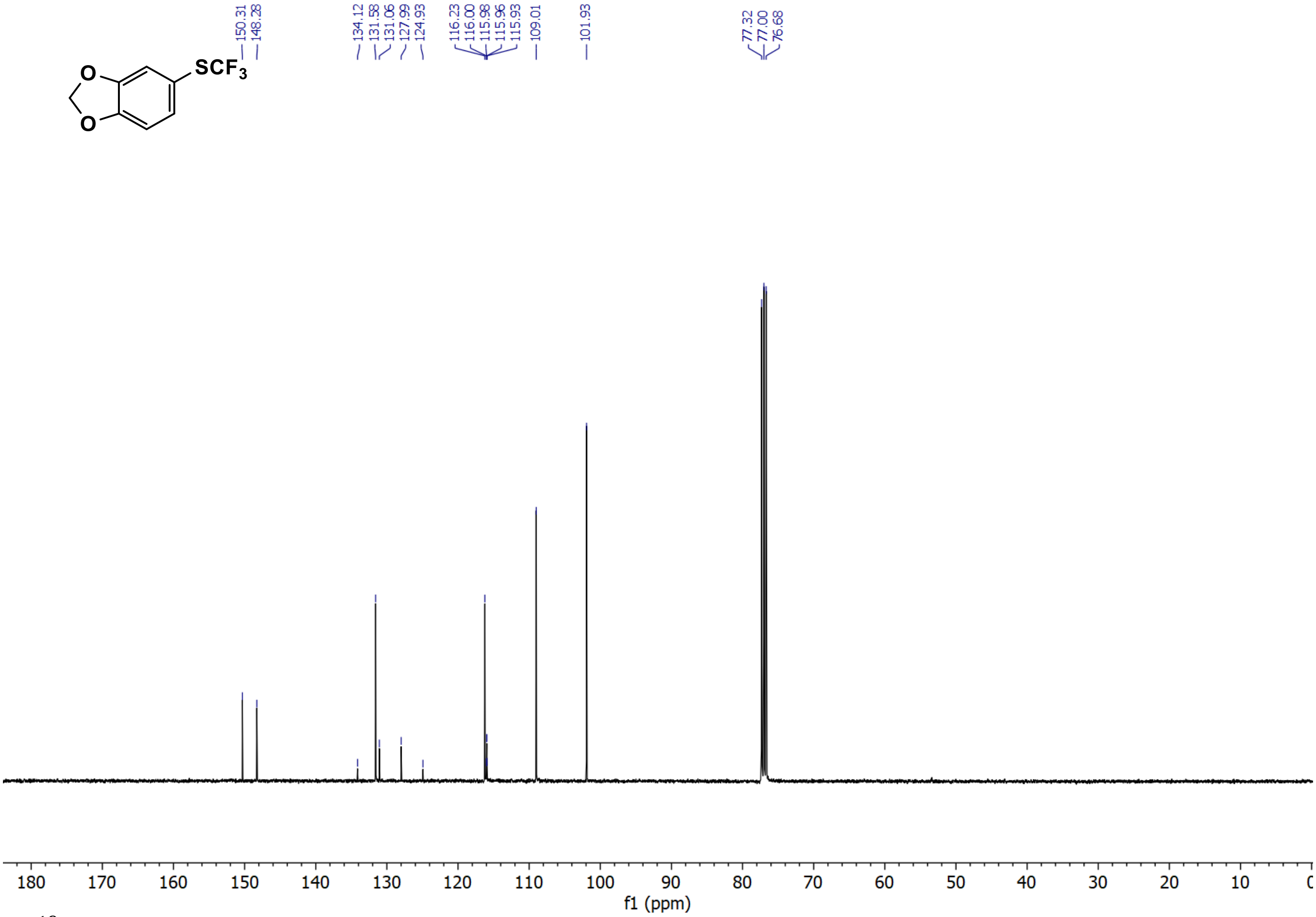

5j ^{19}F NMR (376 MHz, $CDCl_3$)

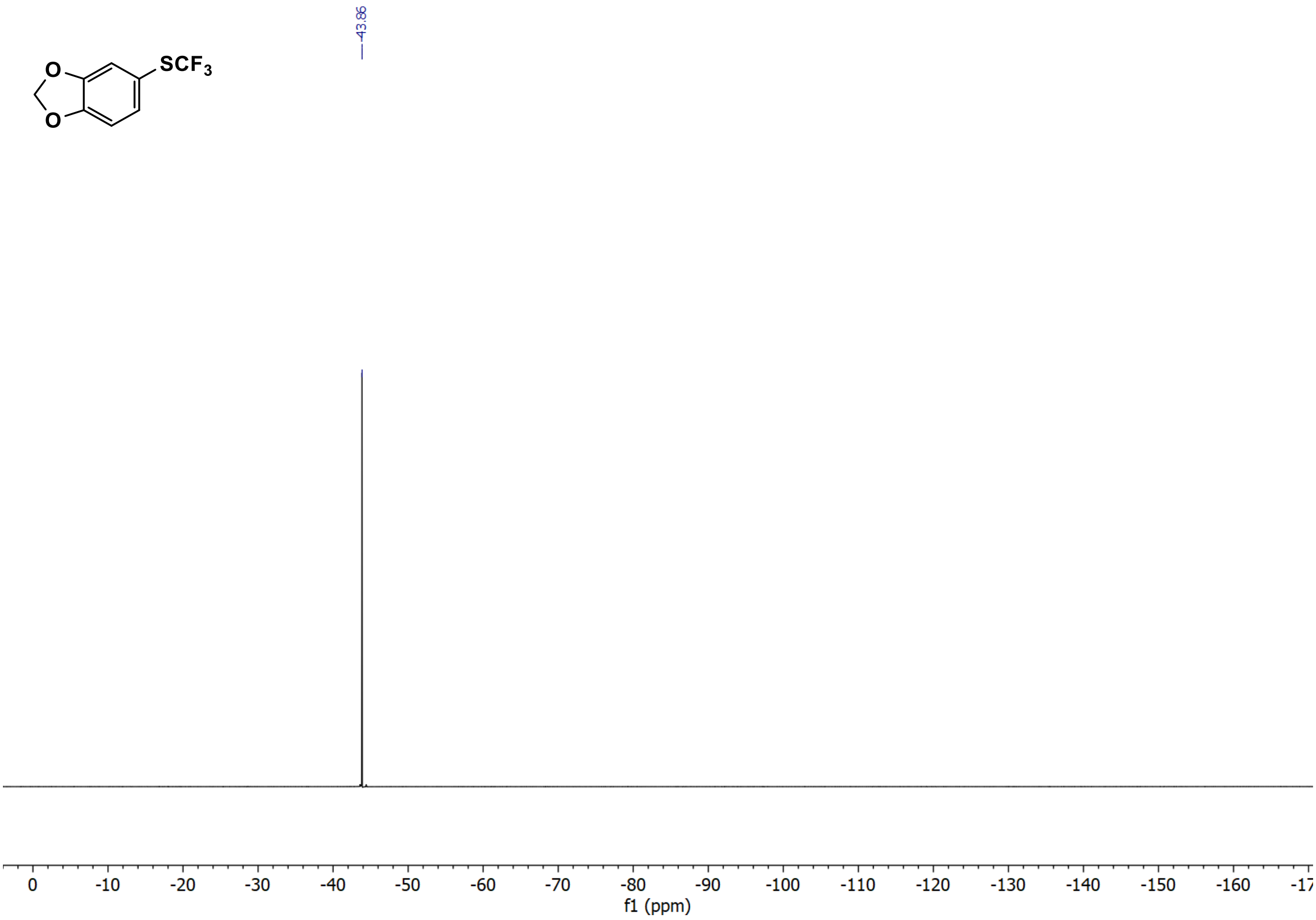

5k (3.9:1 mixture of regioisomers 4-**5k** and 2-**5k**) ^{1}H NMR (400 MHz, $CDCl_3$)

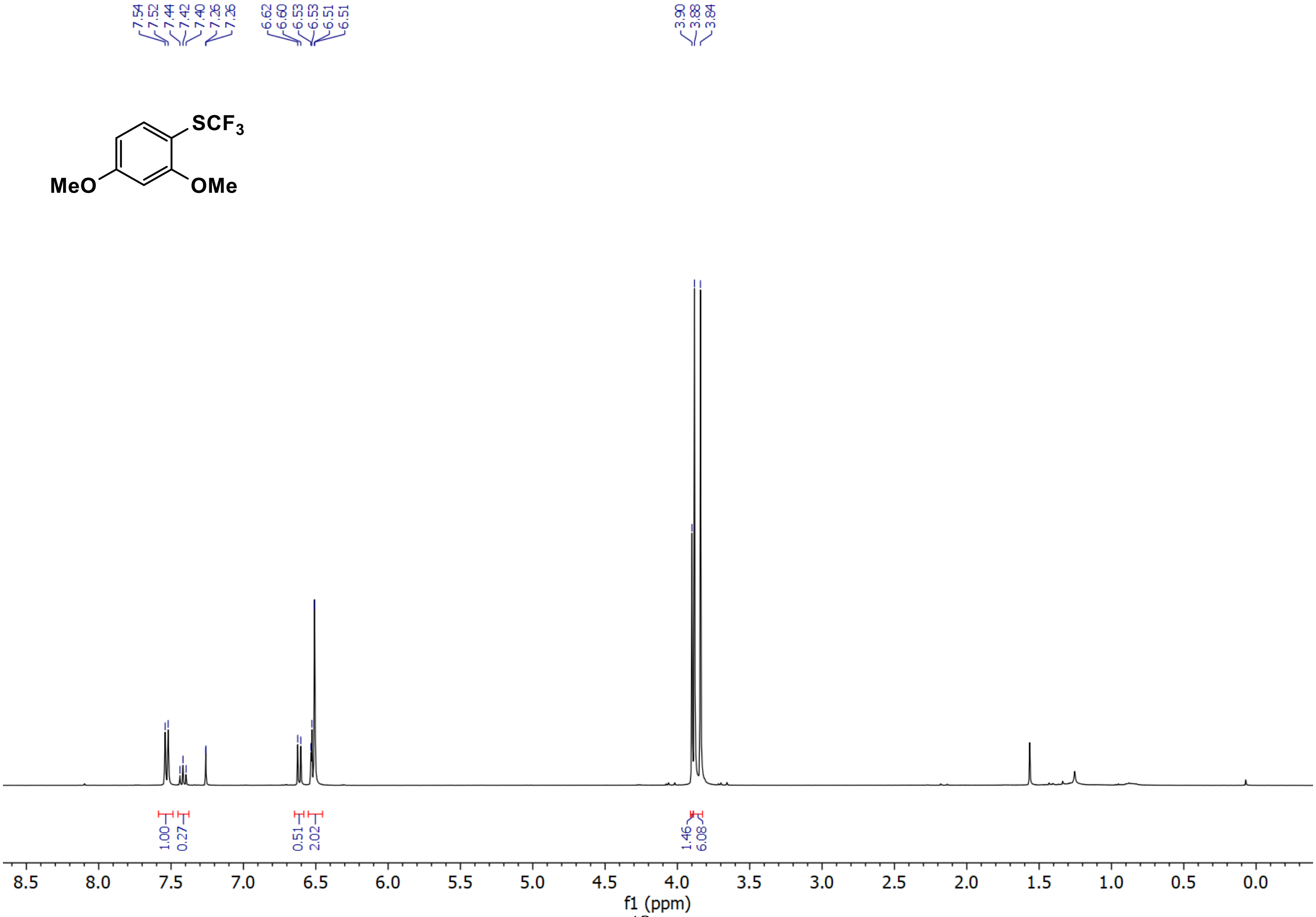

5k (3.9:1 mixture of regioisomers 4-**5k** and 2-**5k**) ^{13}C NMR (101 MHz, $CDCl_3$)

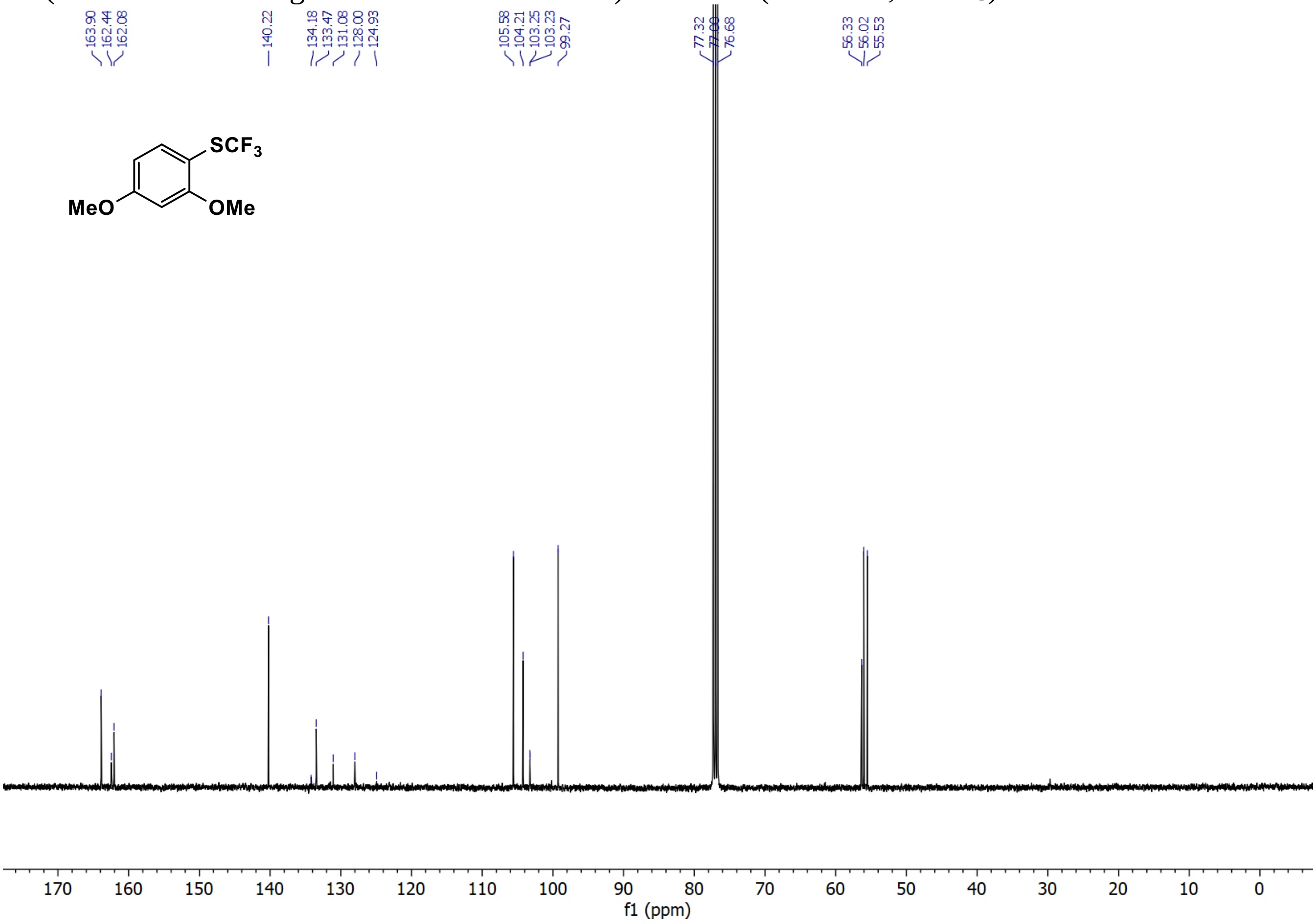

5k (3.9:1 mixture of regioisomers 4-**5k** and 2-**5k**) ^{19}F NMR (376 MHz, $CDCl_3$)

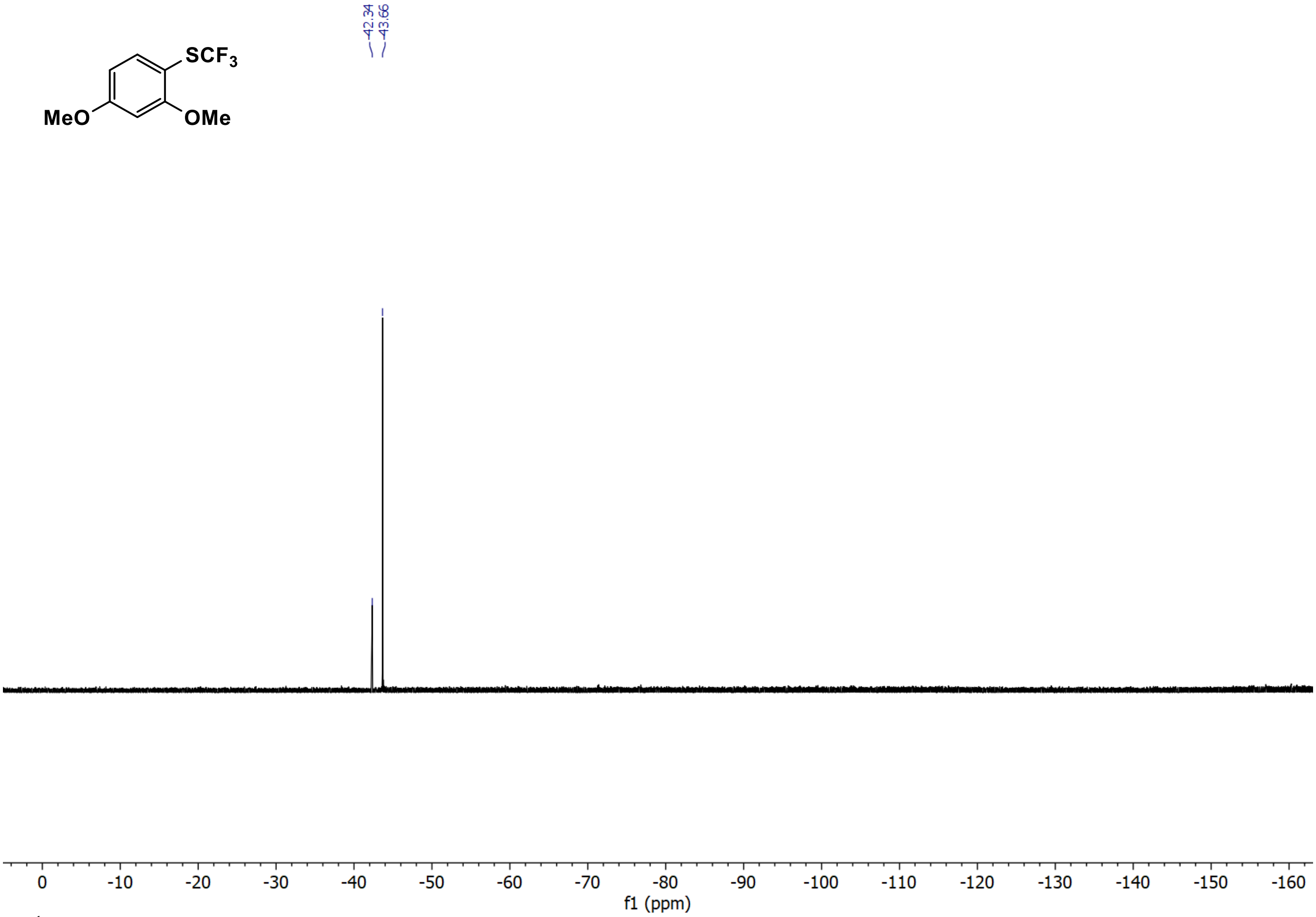

5l ^{1}H NMR (400 MHz, $CDCl_3$)

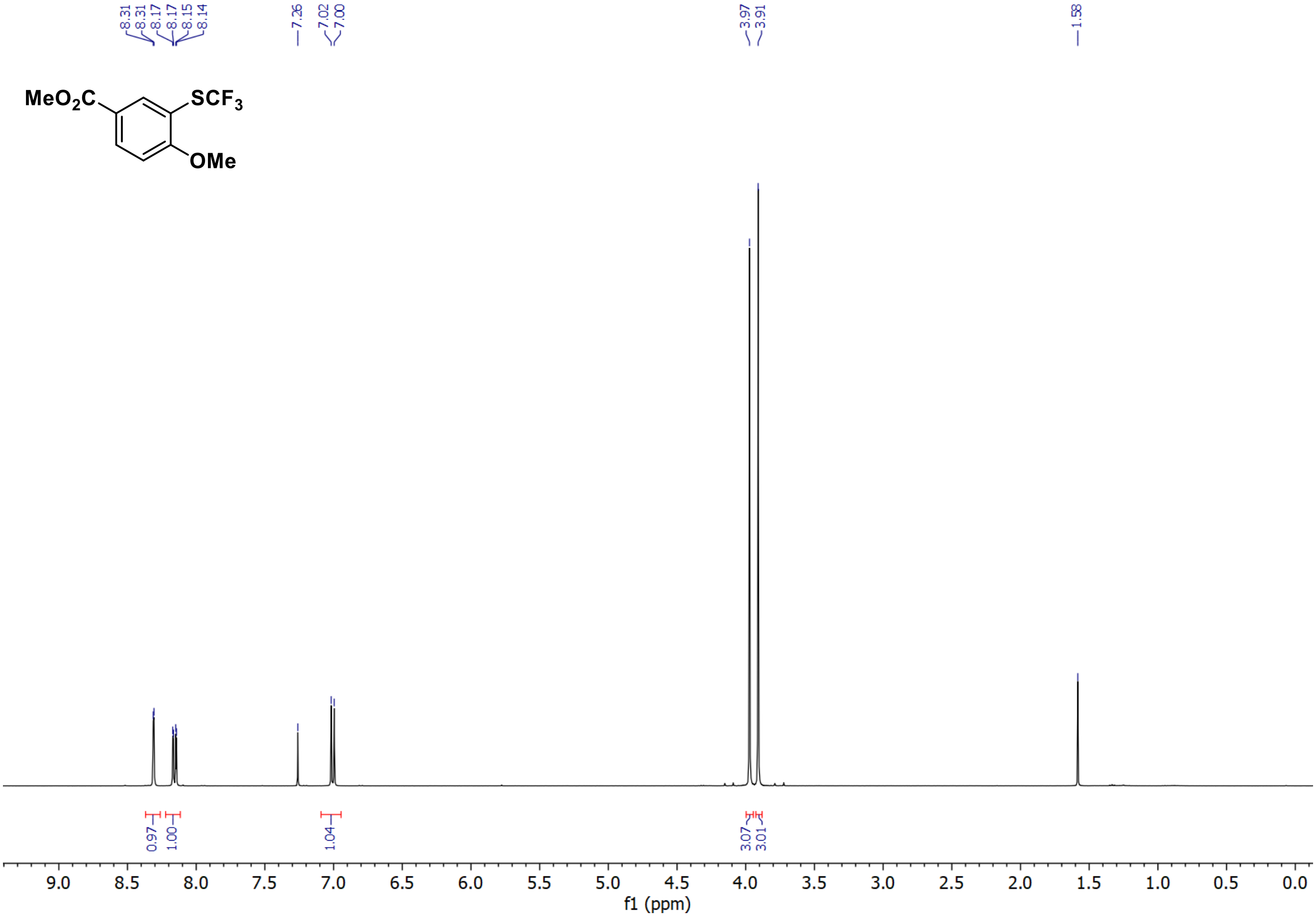

5l ^{13}C NMR (101 MHz, $CDCl_3$)

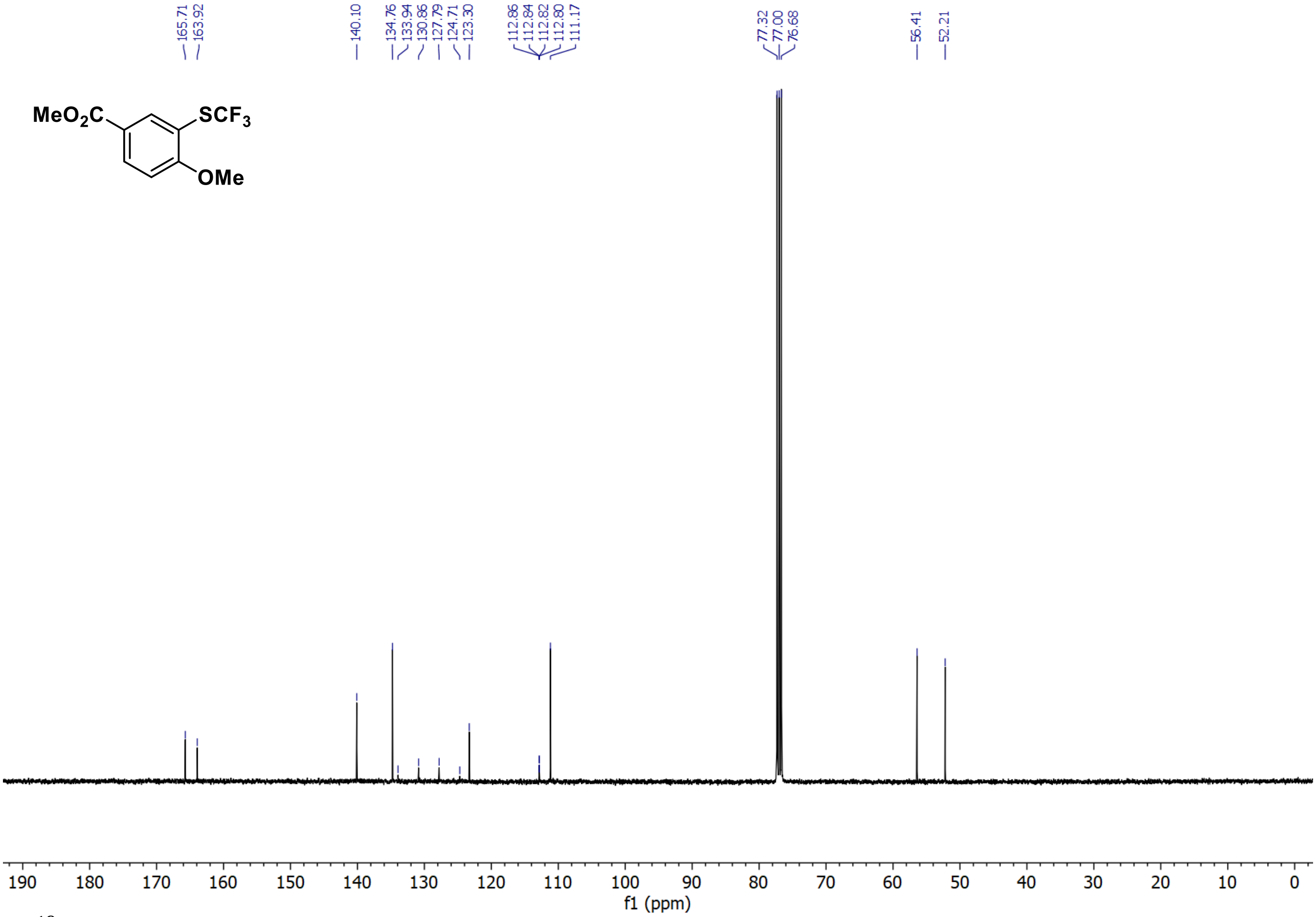

5l ^{19}F NMR (376 MHz, $CDCl_3$)

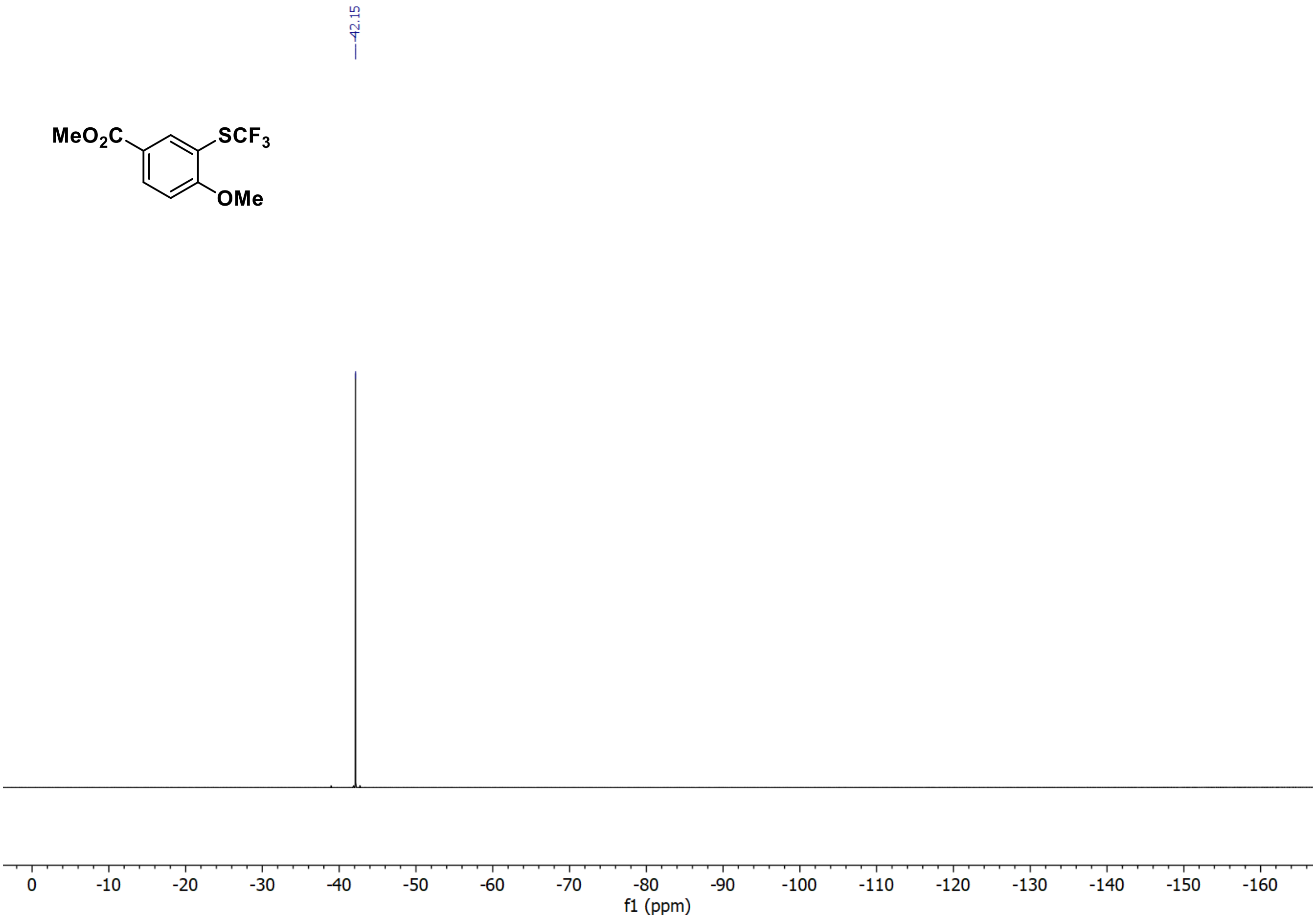

5m ^{1}H NMR (400 MHz, $CDCl_3$)

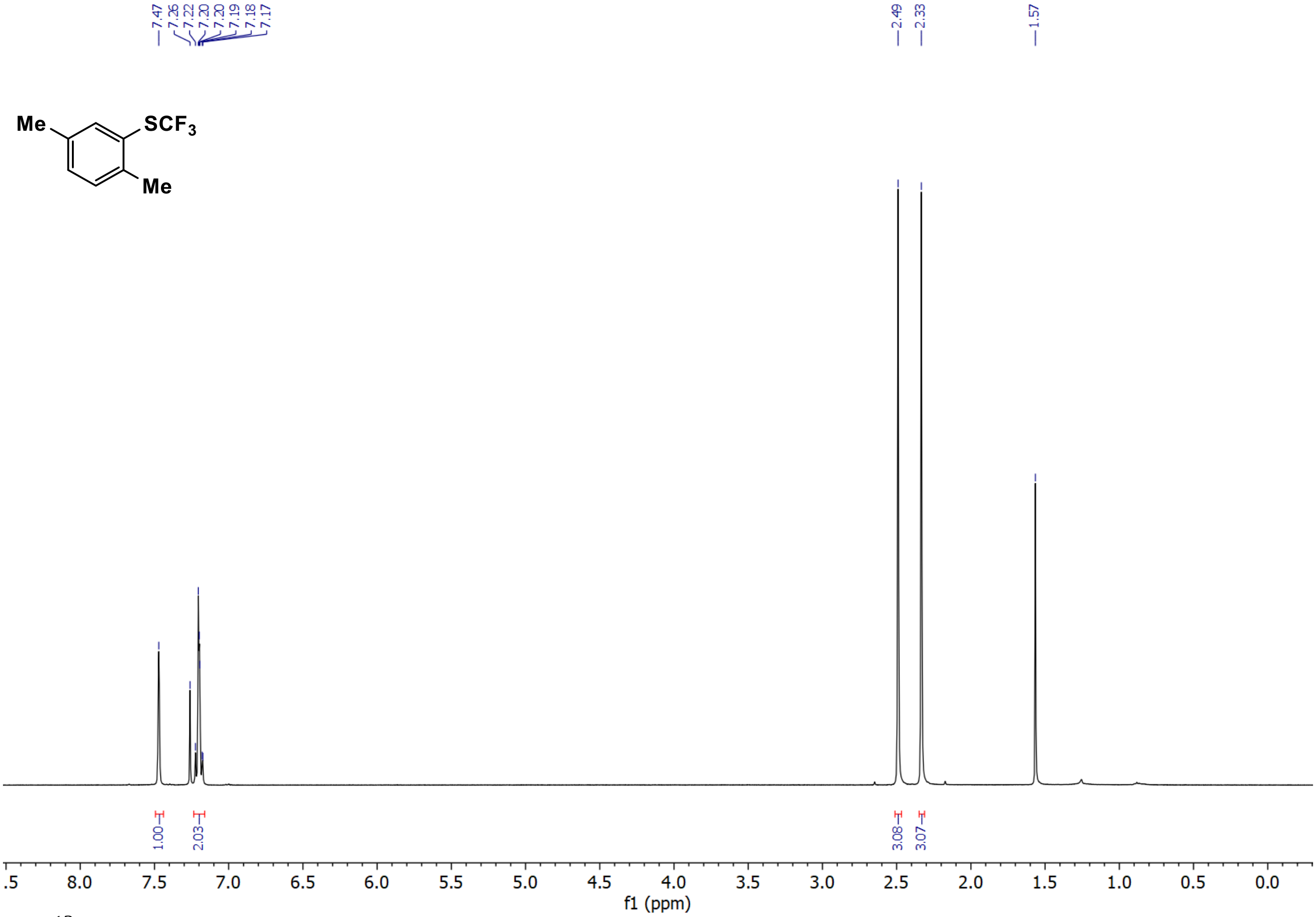

5m ^{13}C NMR (126 MHz, $CDCl_3$)

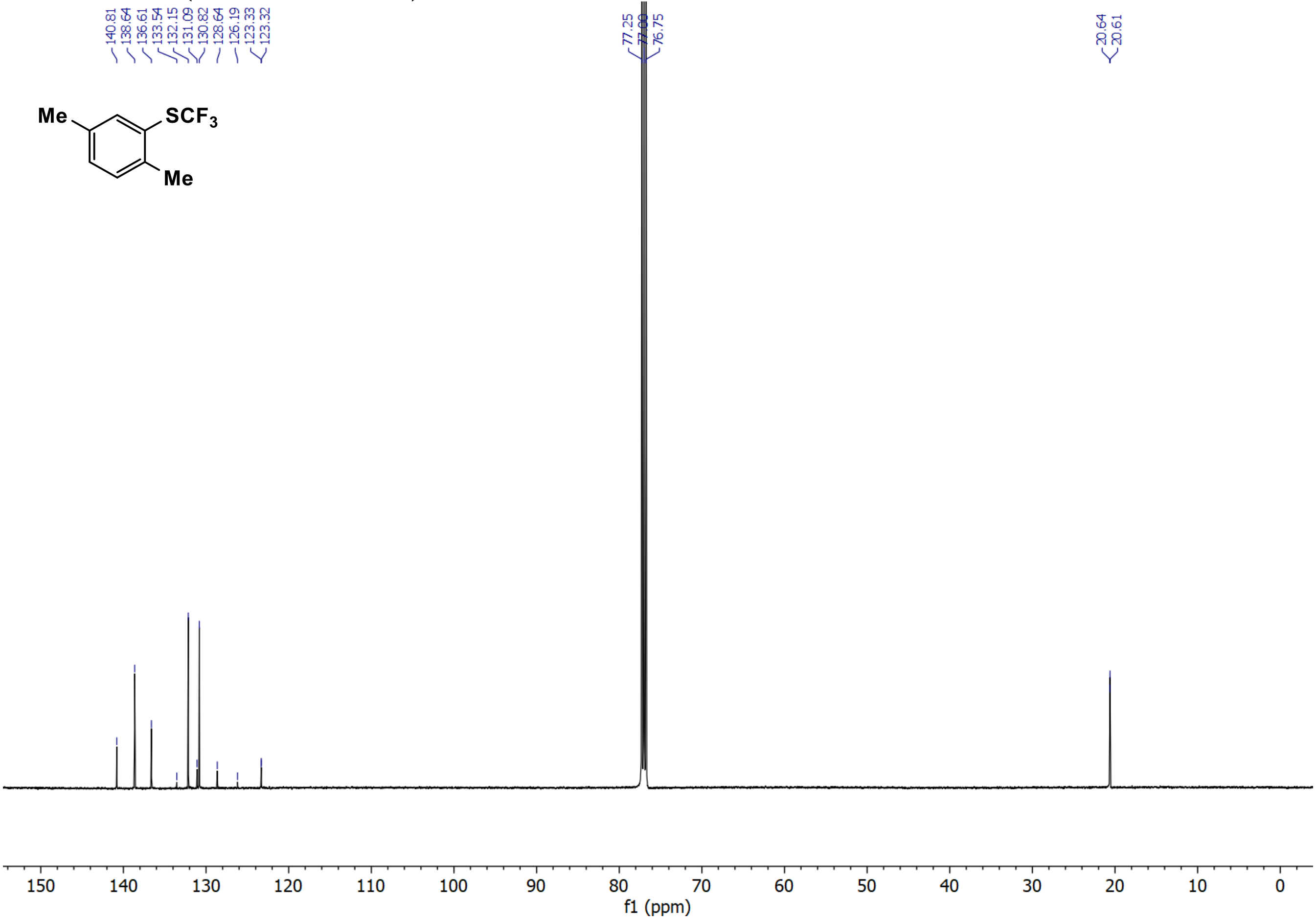

5m ^{19}F NMR (376 MHz, $CDCl_3$)

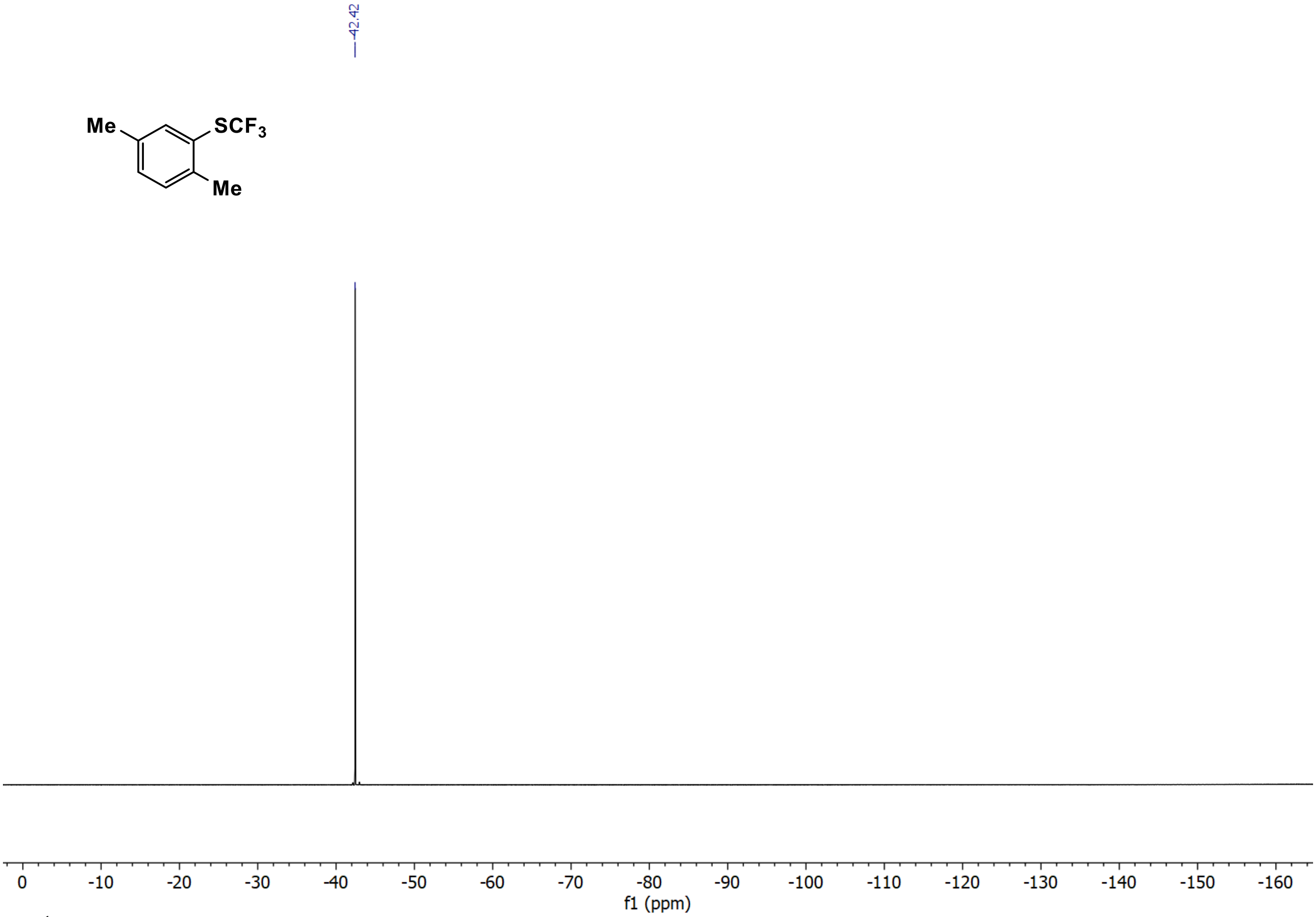

5n ^{1}H NMR (500 MHz, $CDCl_3$)

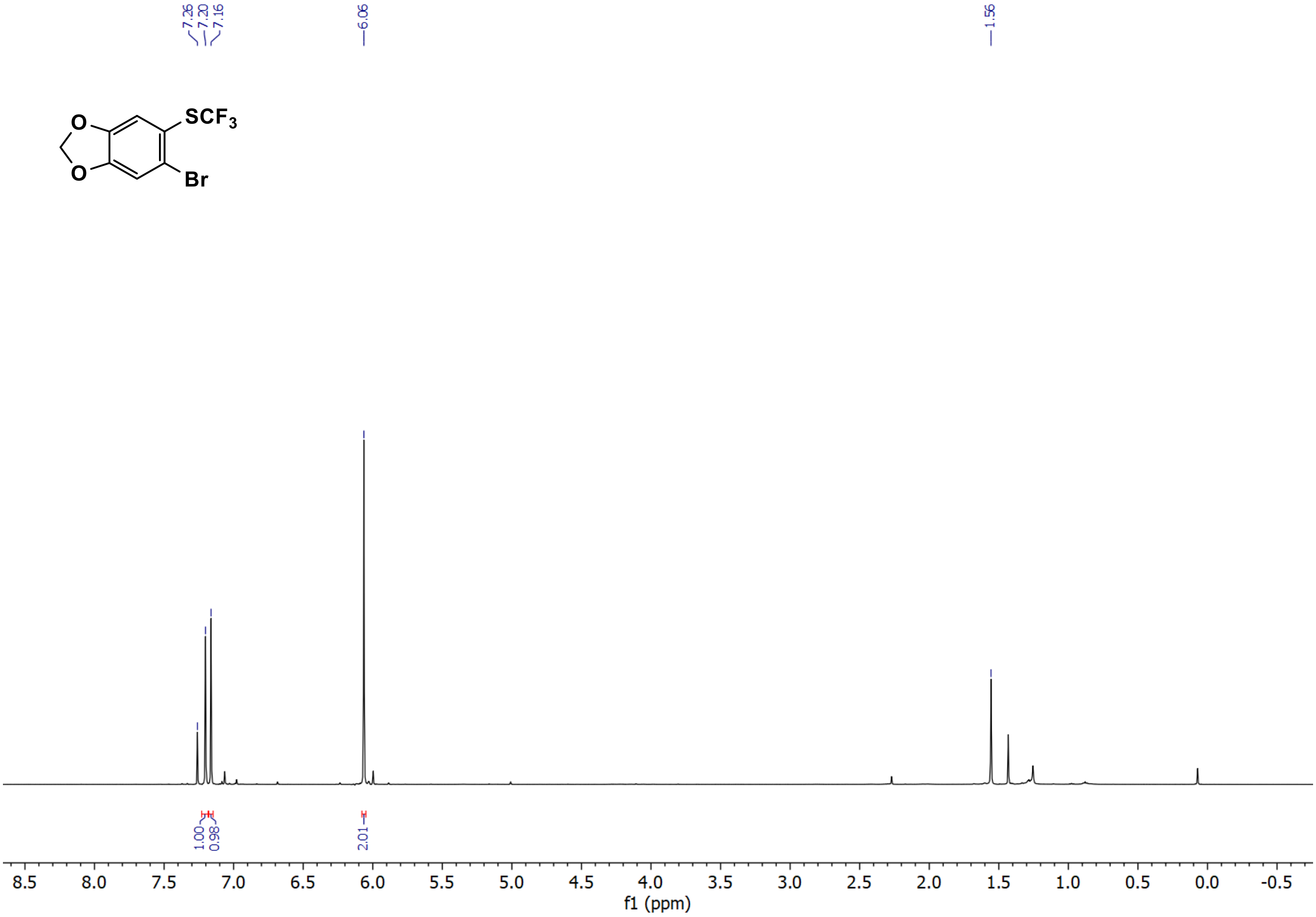

5n ^{13}C NMR (126 MHz, $CDCl_3$)

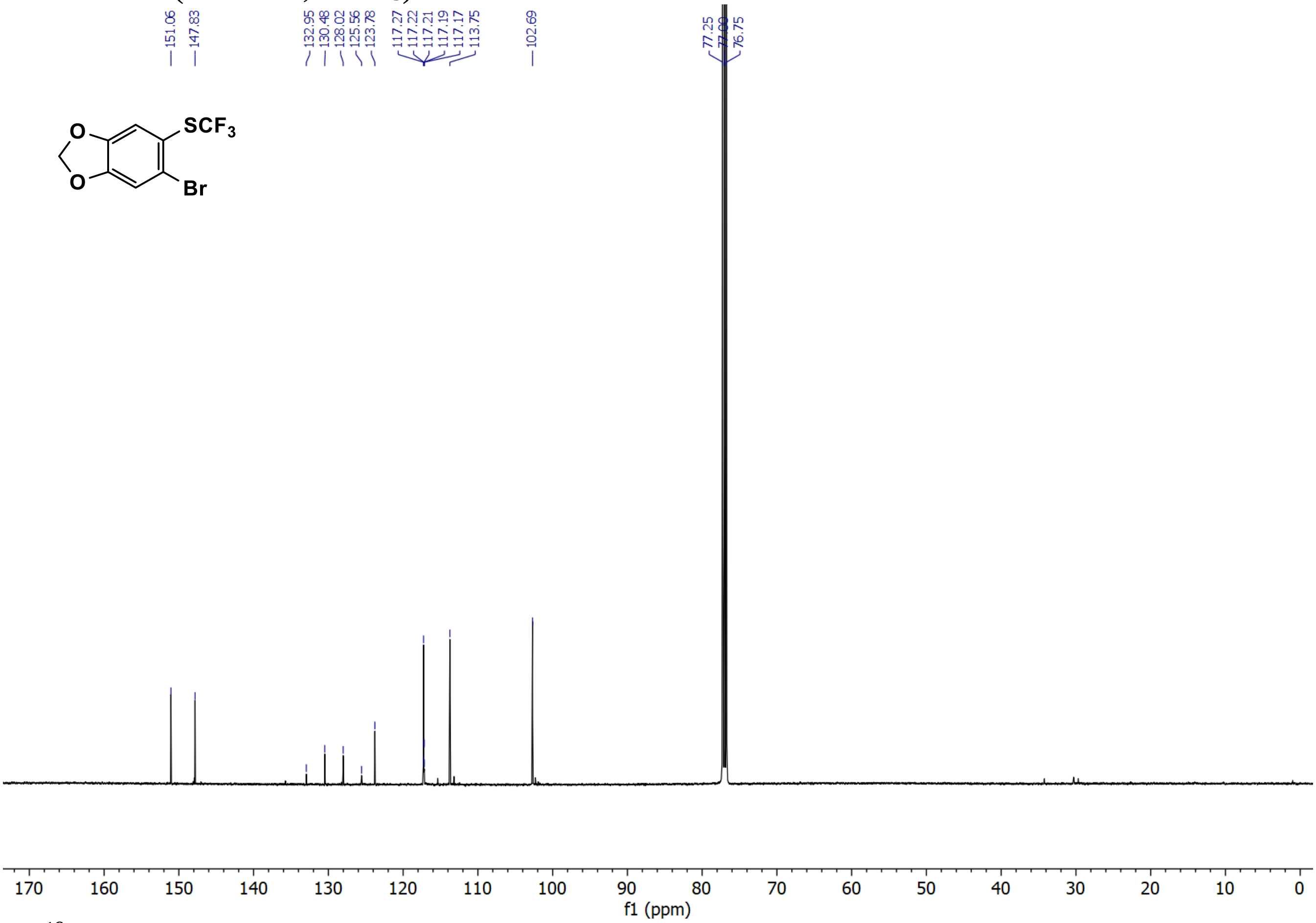

5n ^{19}F NMR (376 MHz, $CDCl_3$)

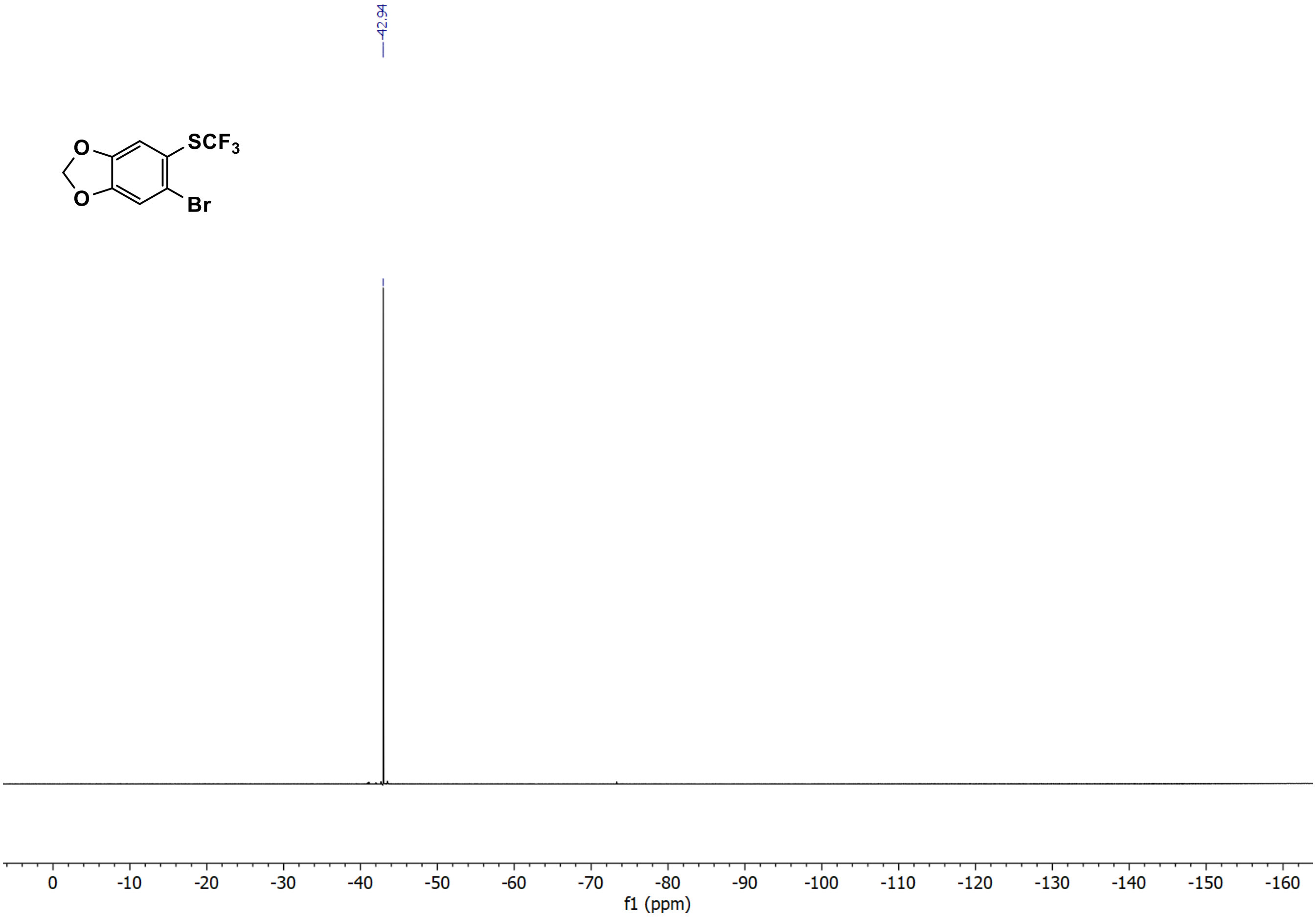

5o ^{1}H NMR (500 MHz, $CDCl_3$)

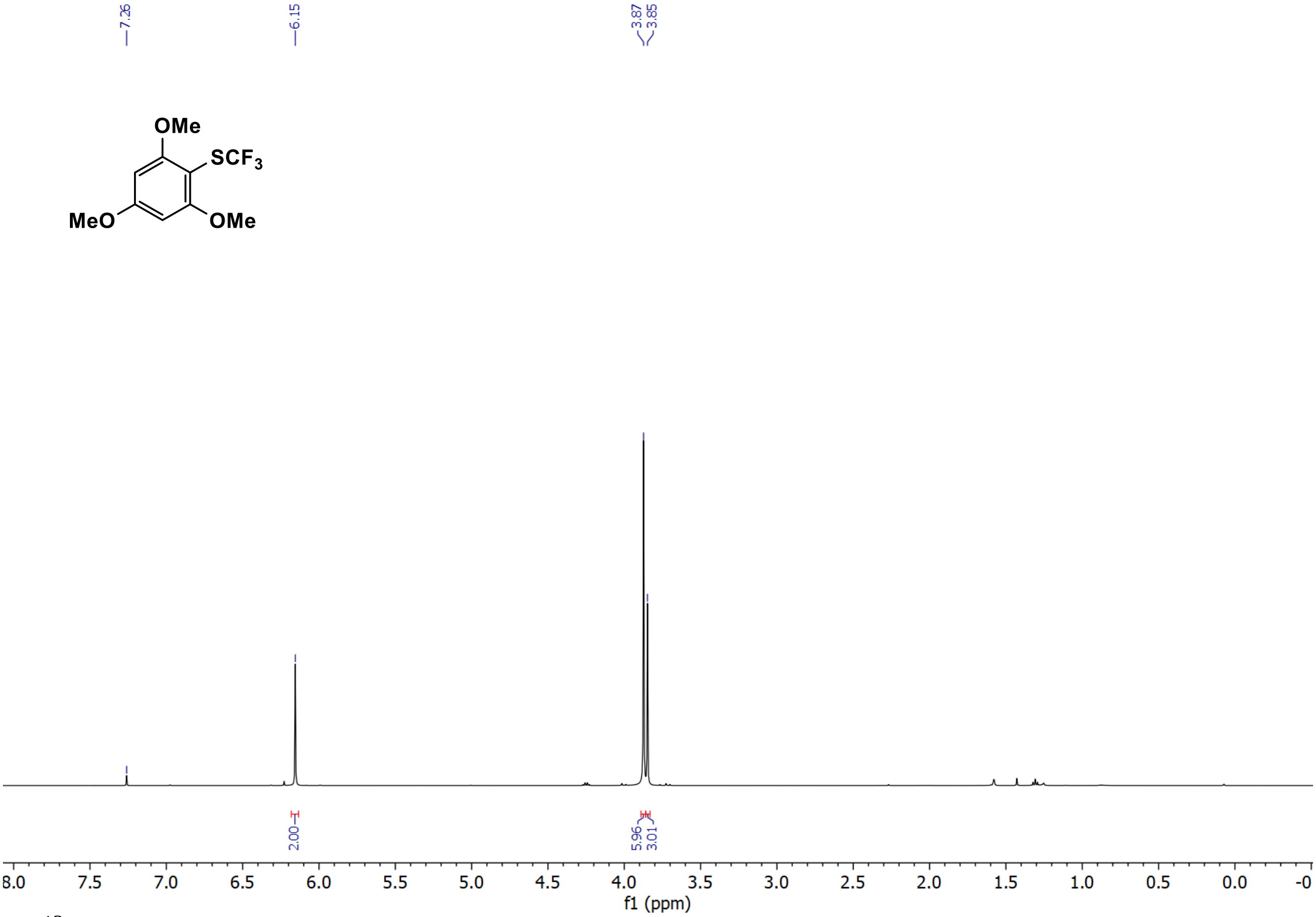

5o ^{13}C NMR (126 MHz, $CDCl_3$)

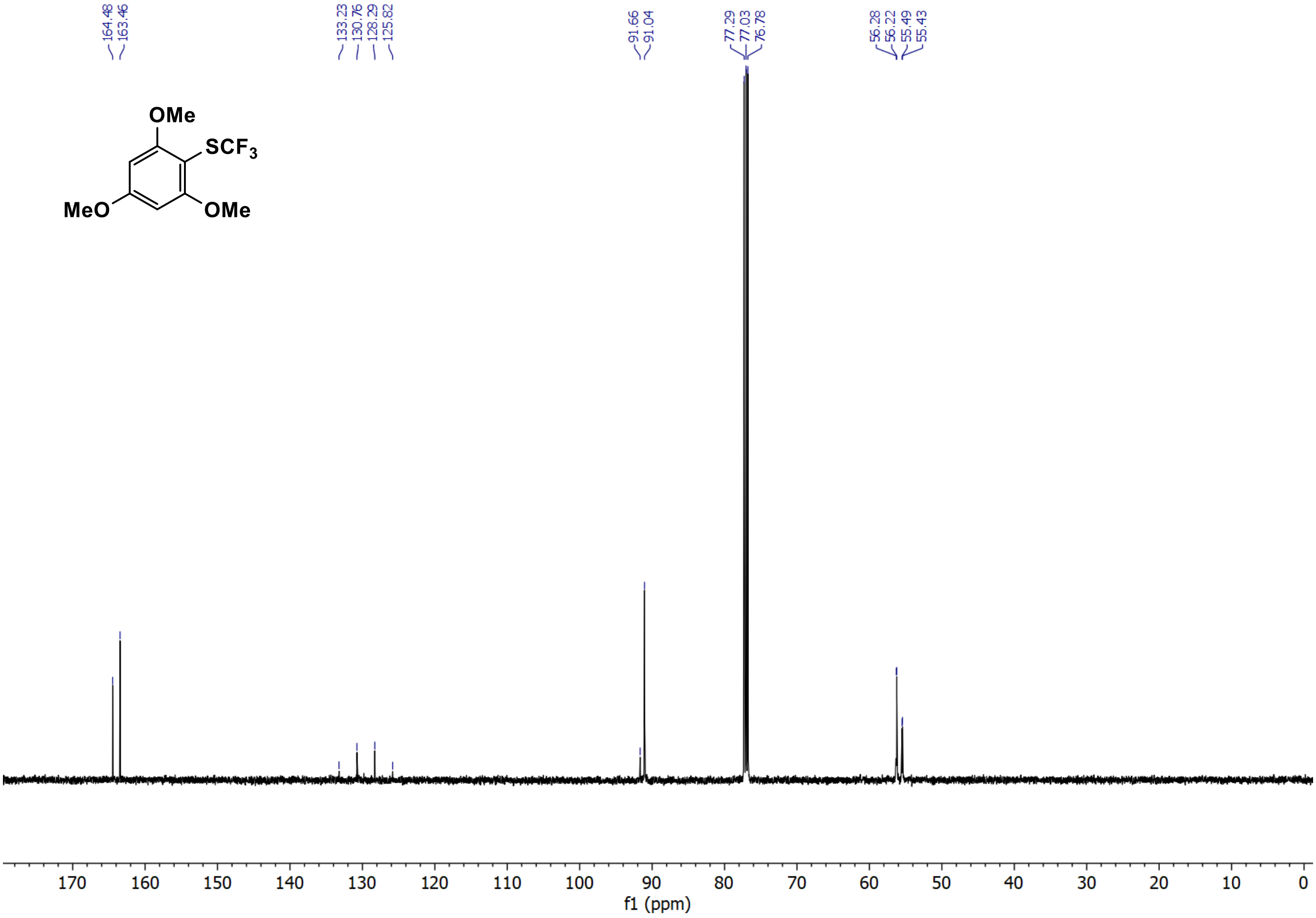

5o ^{19}F NMR (471 MHz, $CDCl_3$)

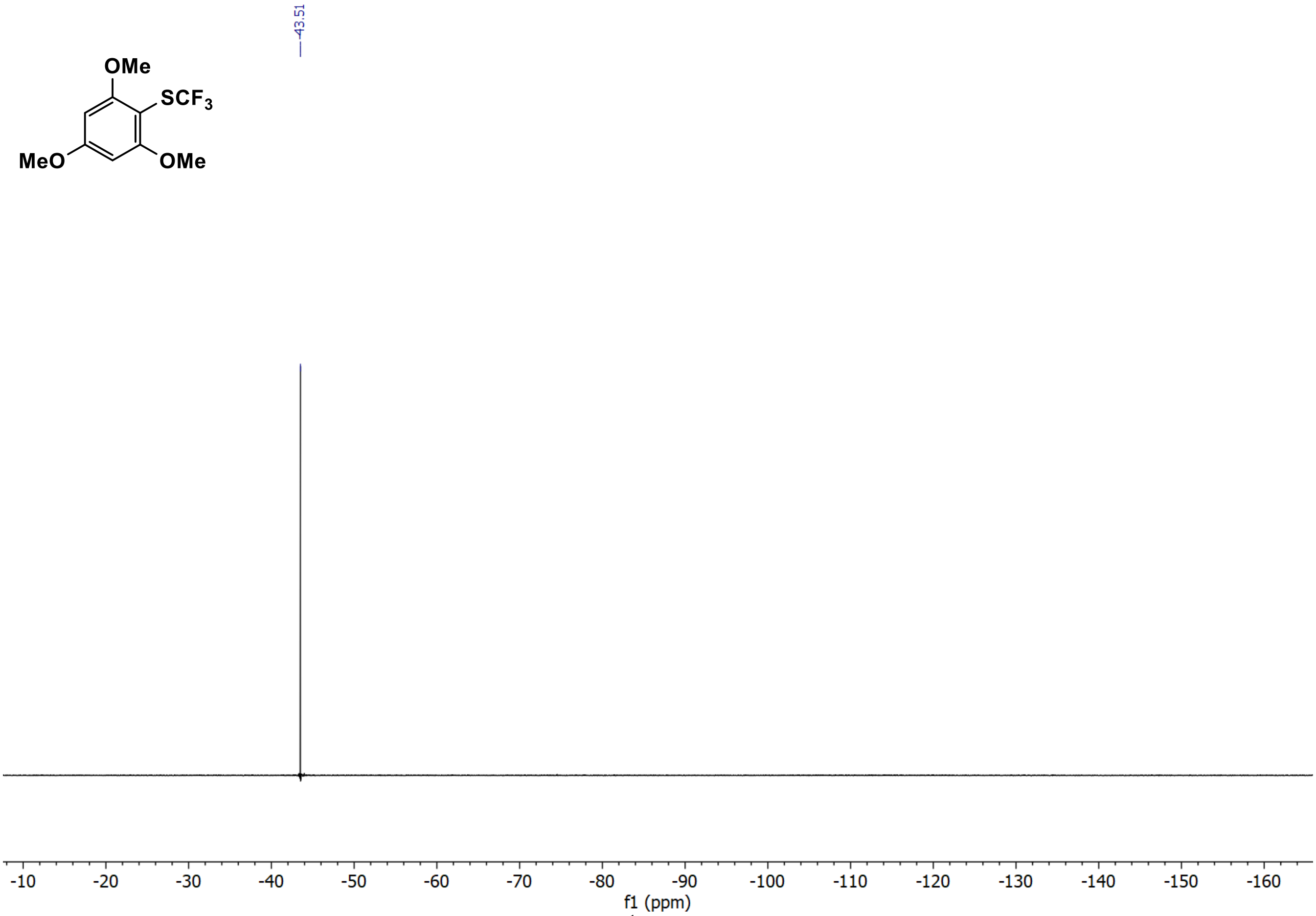

5p (6.3:1 mixture of regioisomers 1-**5p** and 2-**5p**) ^{1}H NMR (400 MHz, $CDCl_3$)

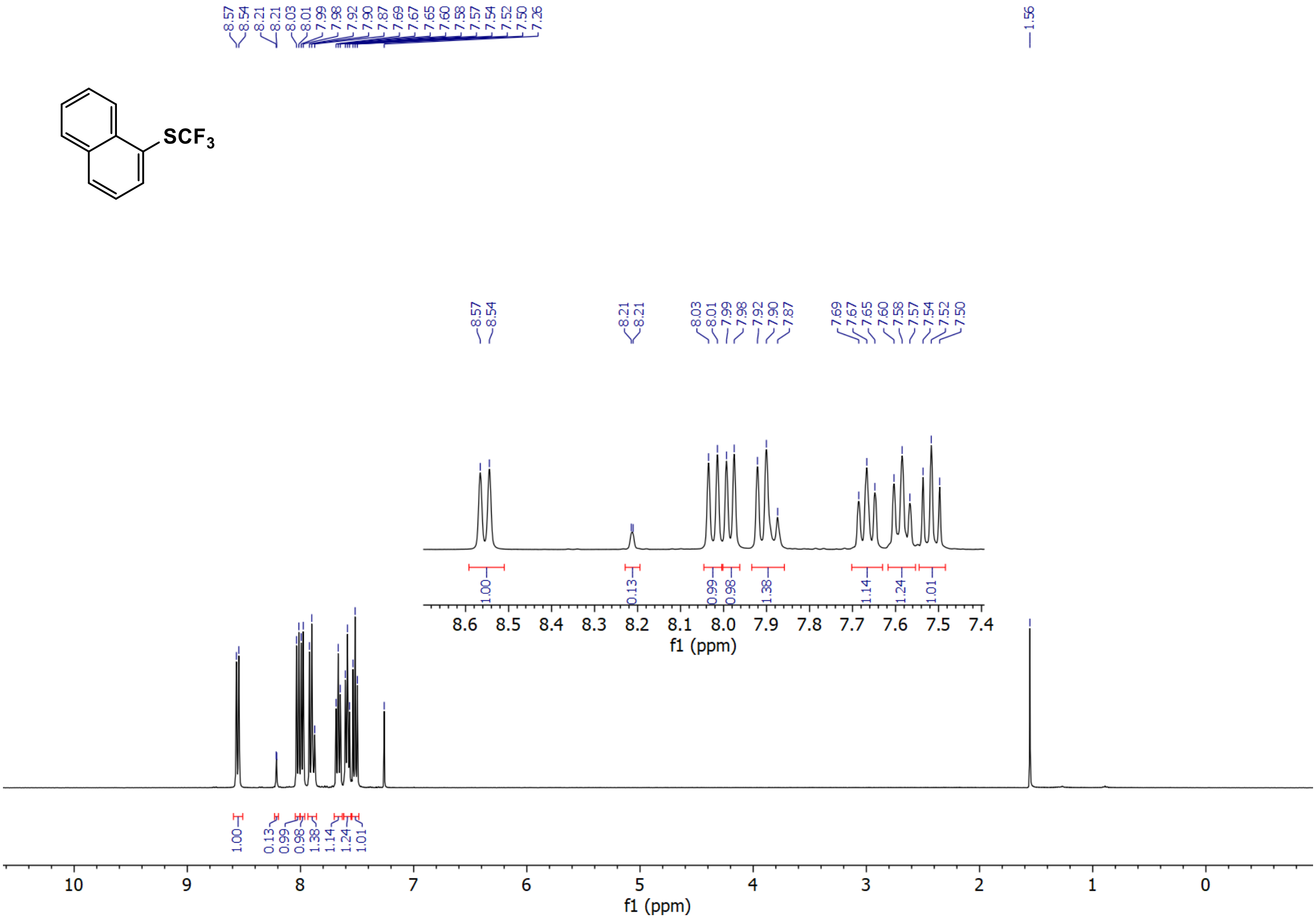

5p (6.3:1 mixture of regioisomers 1-**5p** and 2-**5p**) ^{13}C NMR (126 MHz, $CDCl_3$)

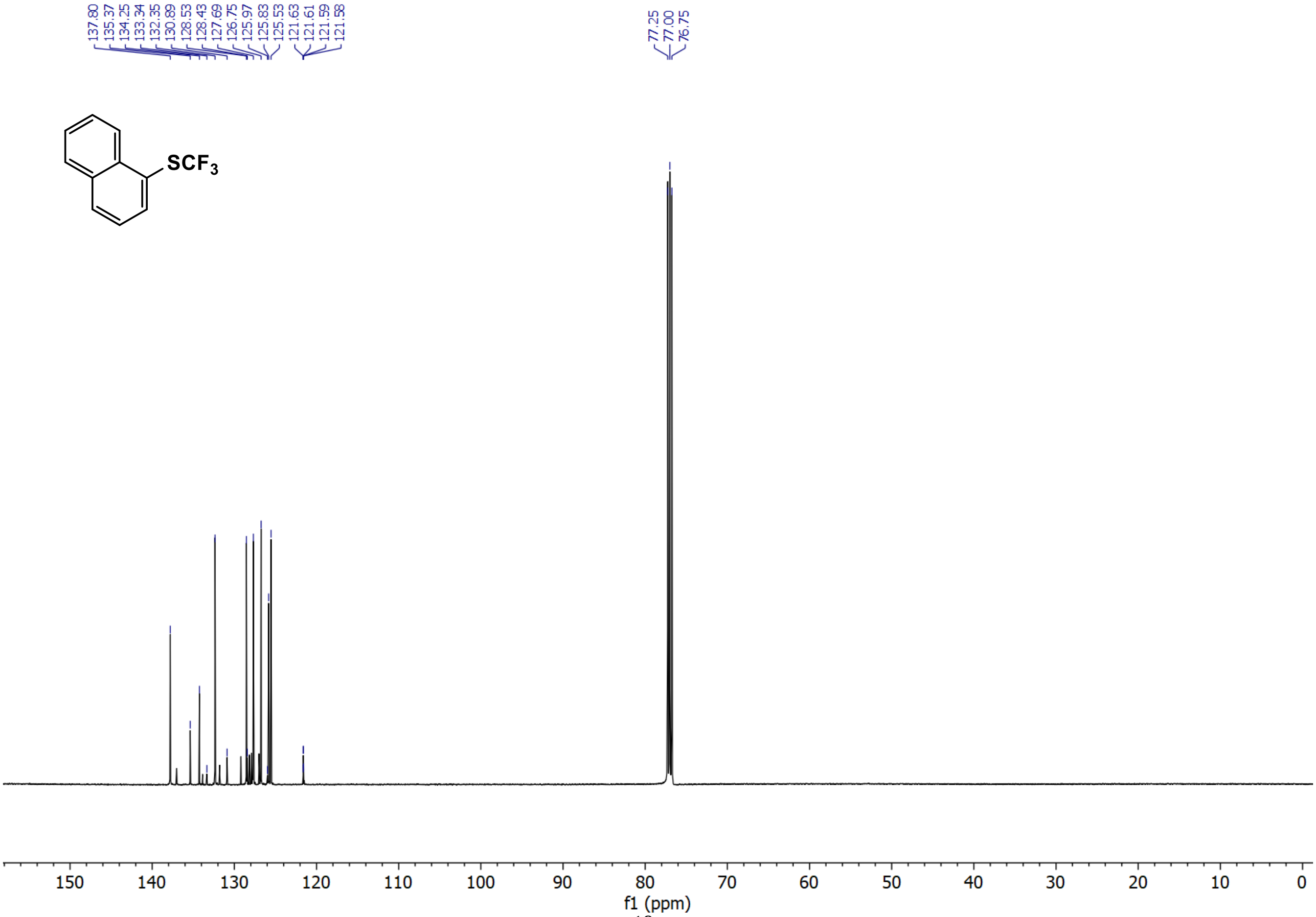

5p (6.3:1 mixture of regioisomers 1-**5p** and 2-**5p**) ^{19}F NMR (376 MHz, $CDCl_3$)

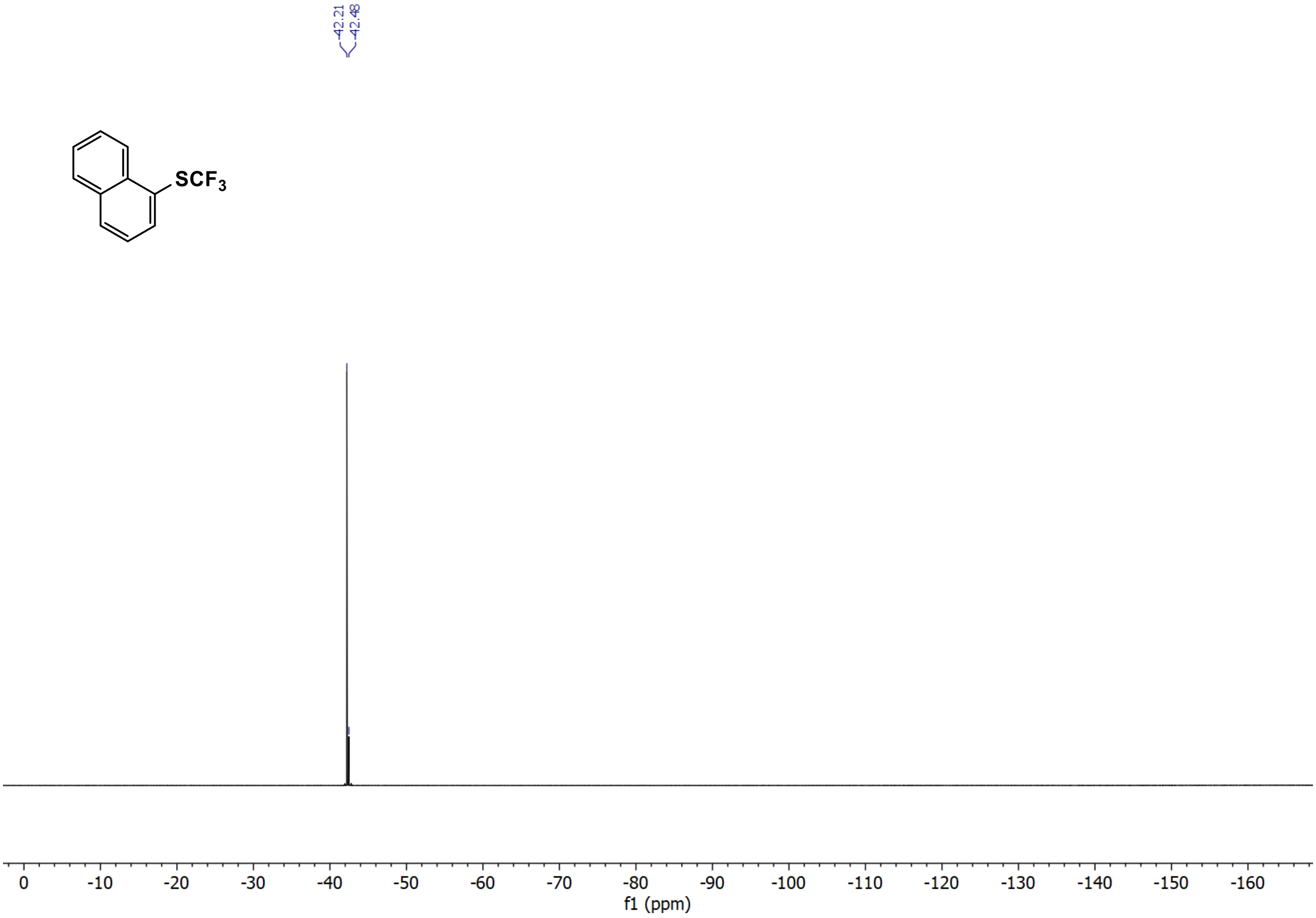

5q ^{1}H NMR (400 MHz, $CDCl_3$)

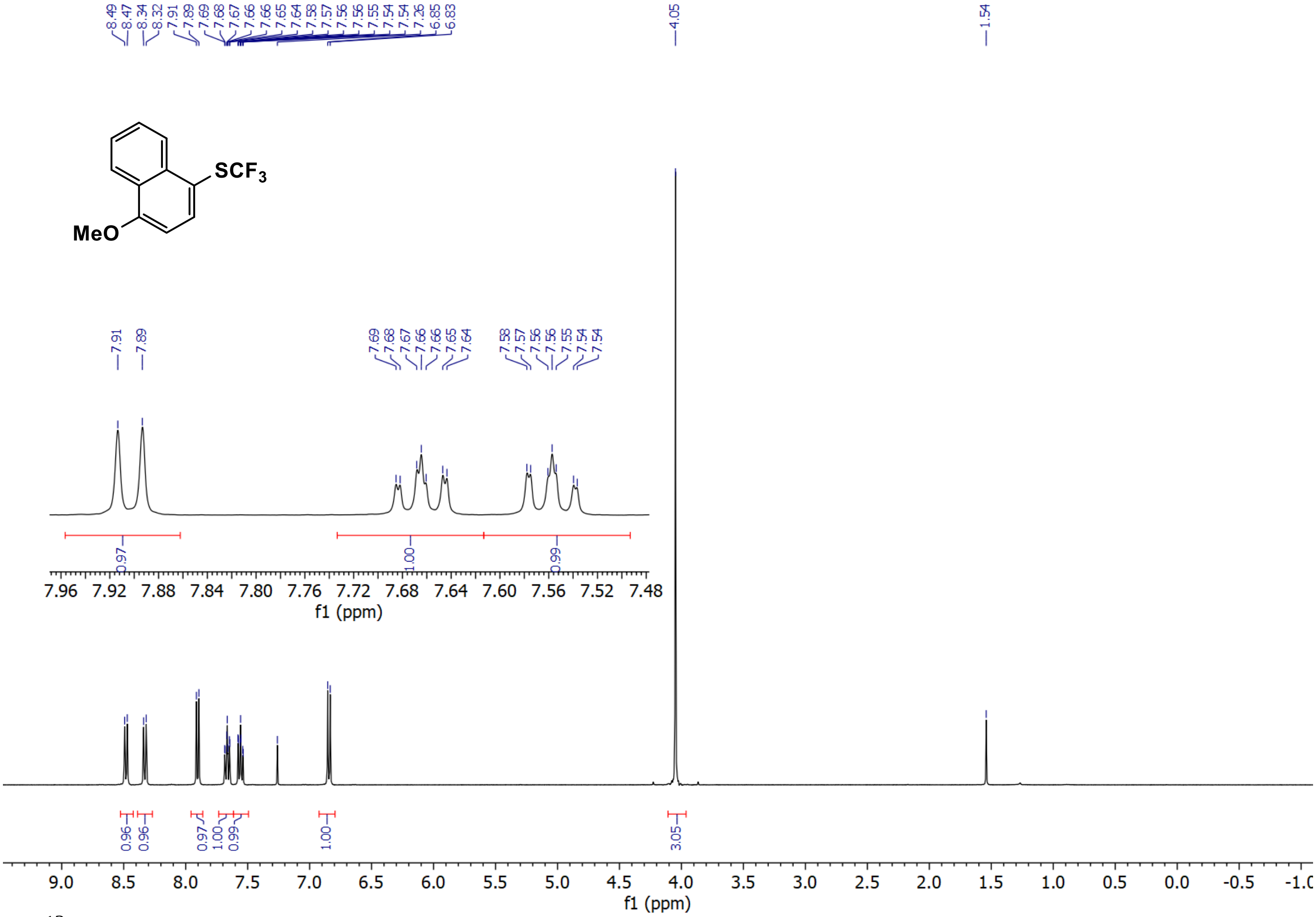

5q ^{13}C NMR (101 MHz, $CDCl_3$)

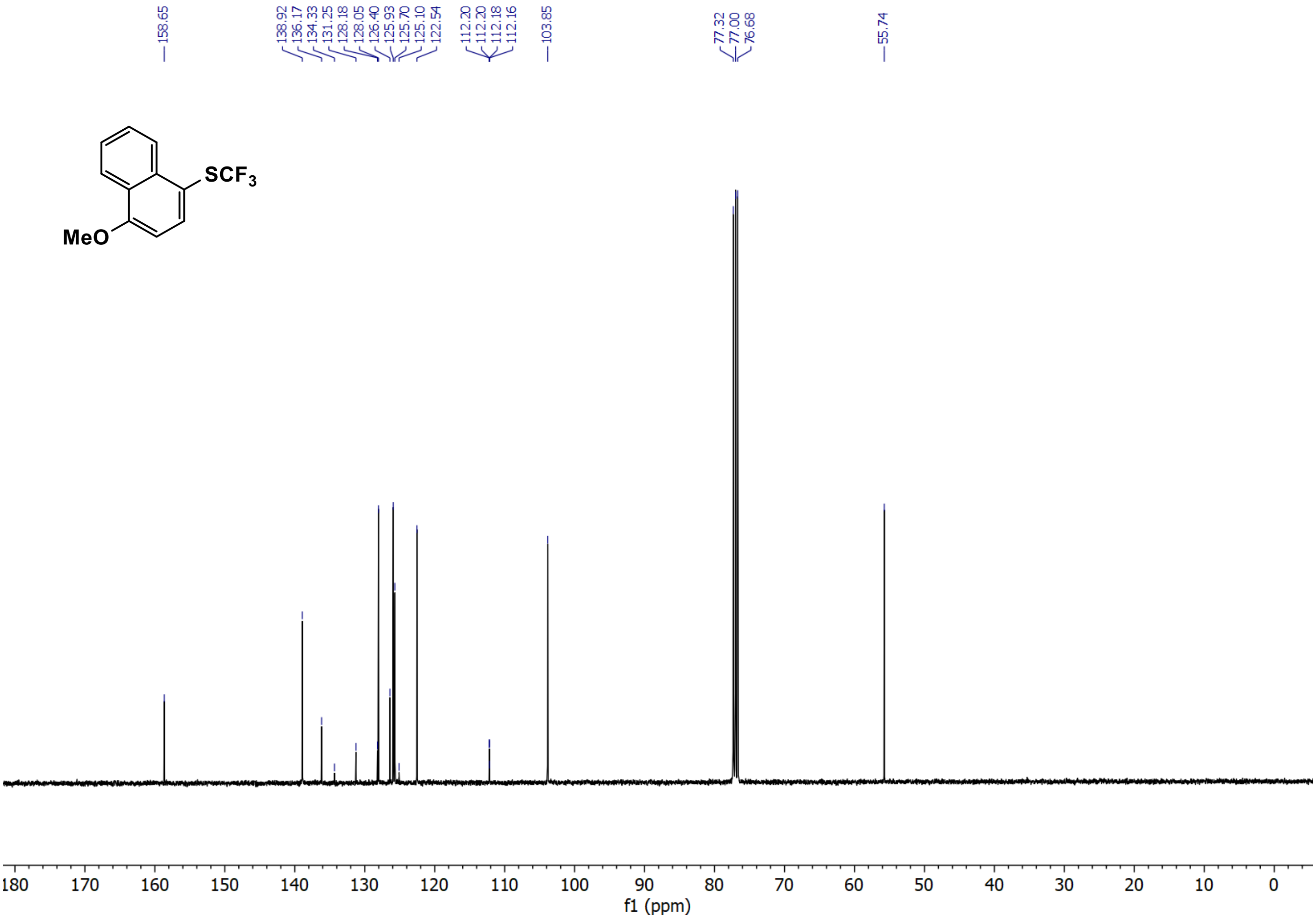

5q ^{19}F NMR (376 MHz, $CDCl_3$)

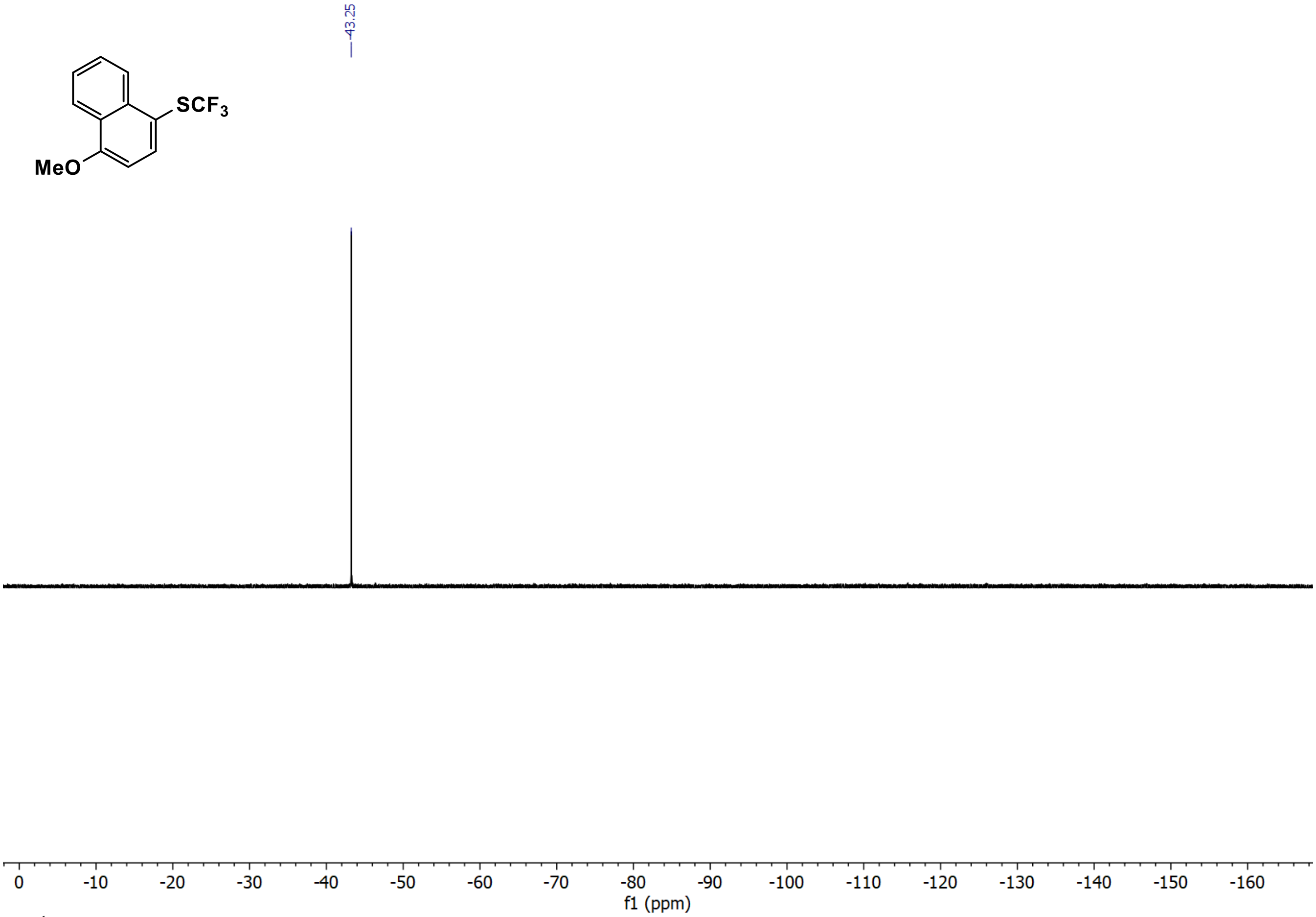

5r ^{1}H NMR (400 MHz, $CDCl_3$)

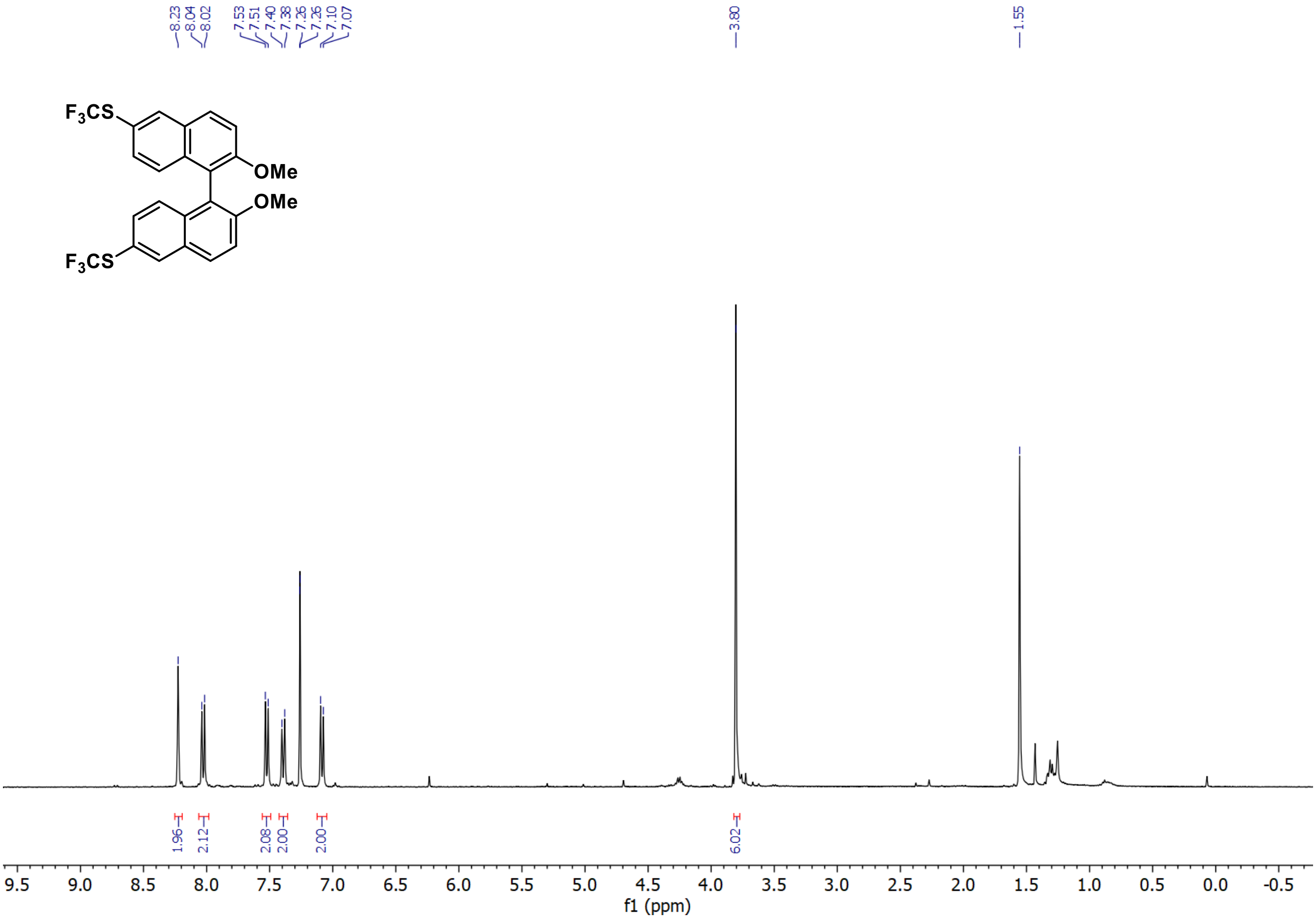

5r ^{13}C NMR (126 MHz, $CDCl_3$)

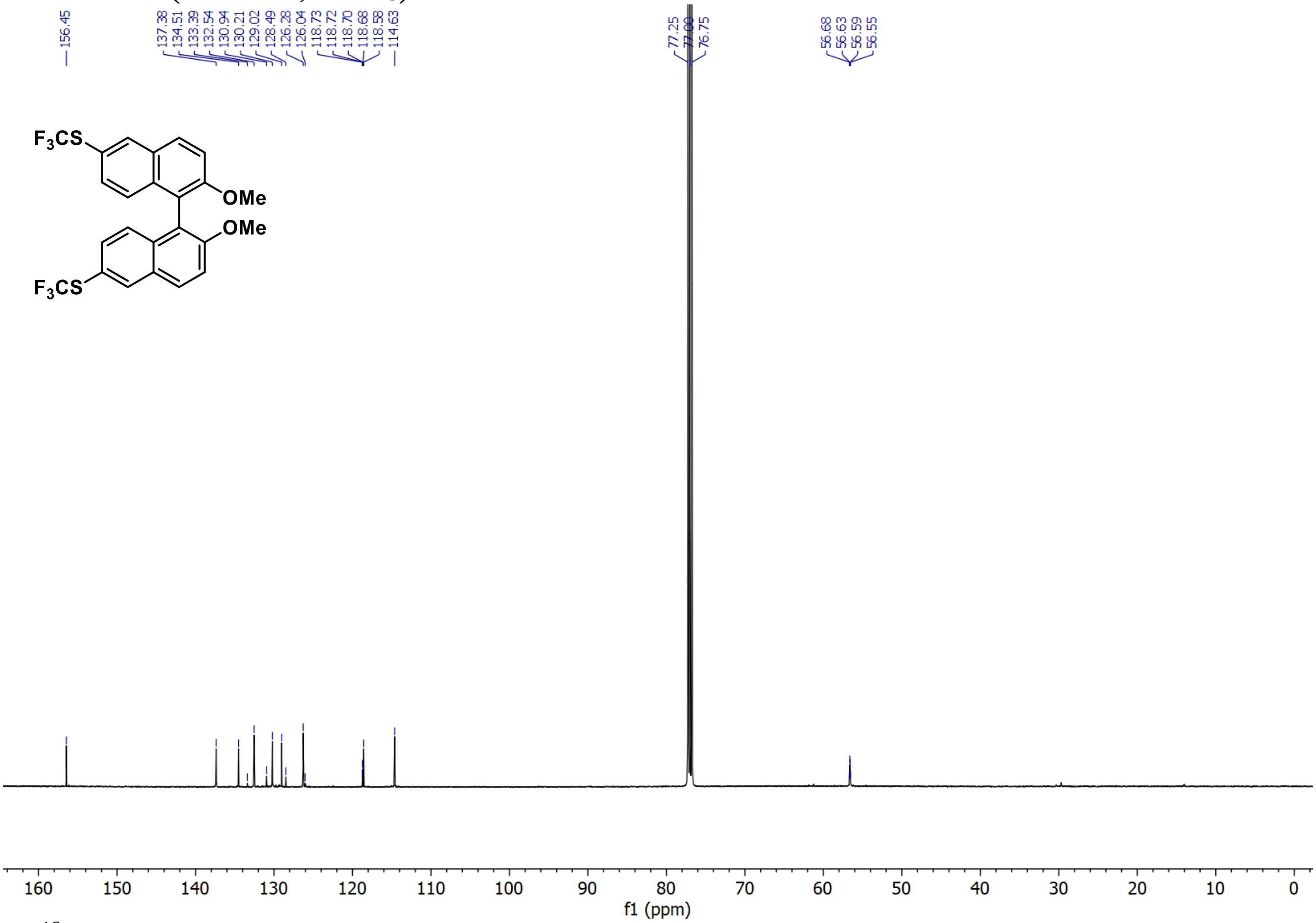

5r ^{19}F NMR (376 MHz, $CDCl_3$)

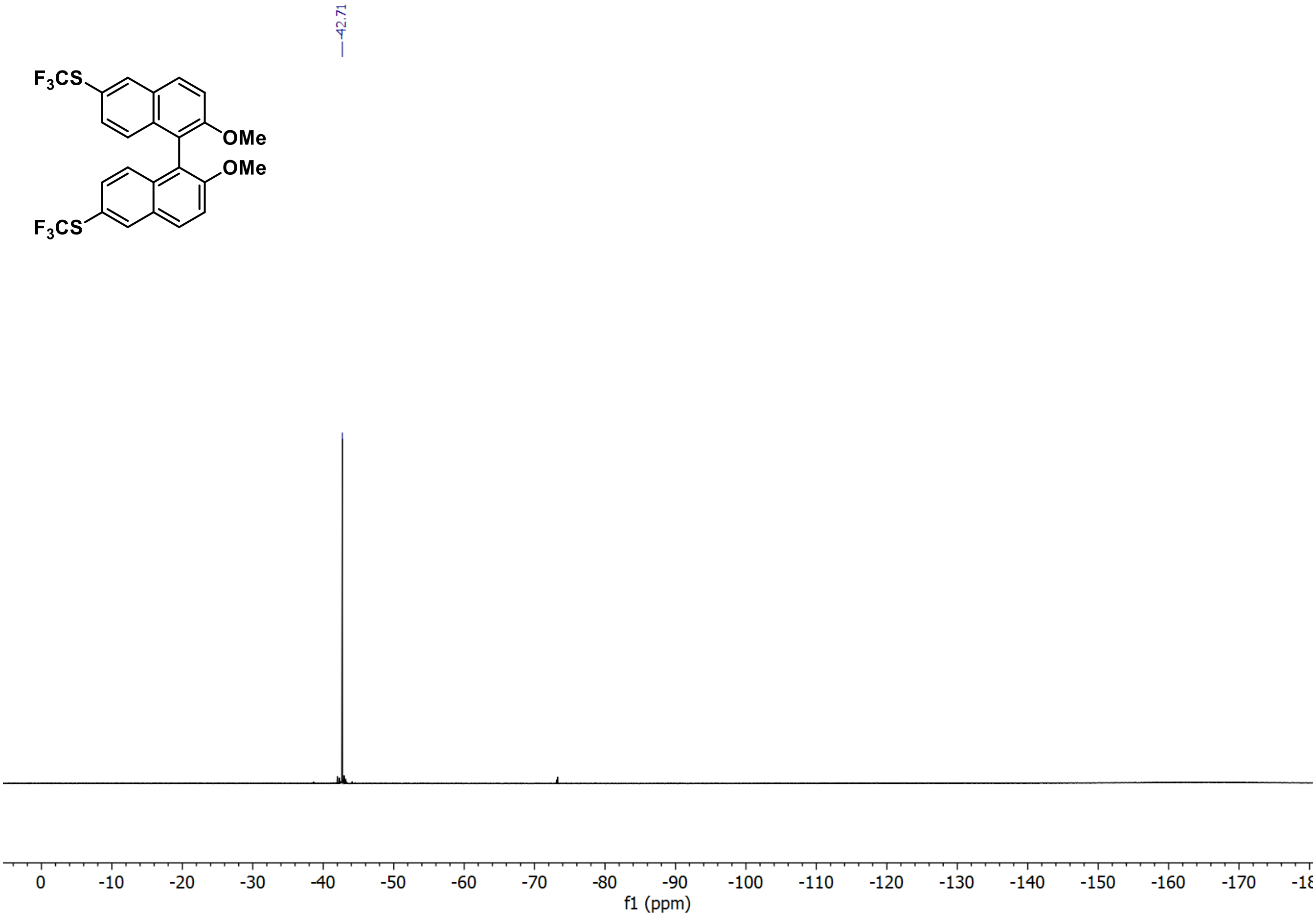

5s ^{1}H NMR (400 MHz, $CDCl_3$)

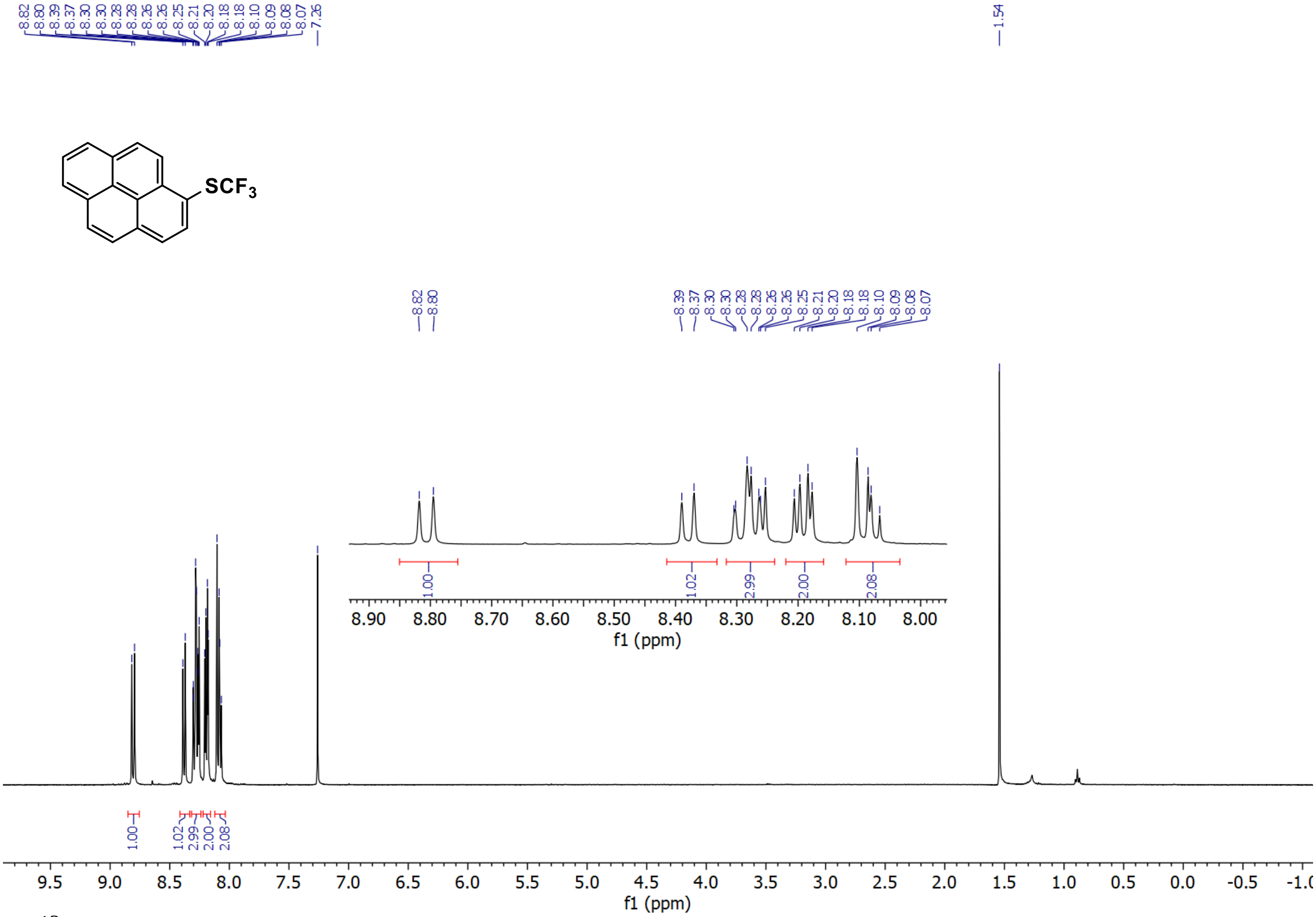

5s ^{13}C NMR (126 MHz, $CDCl_3$)

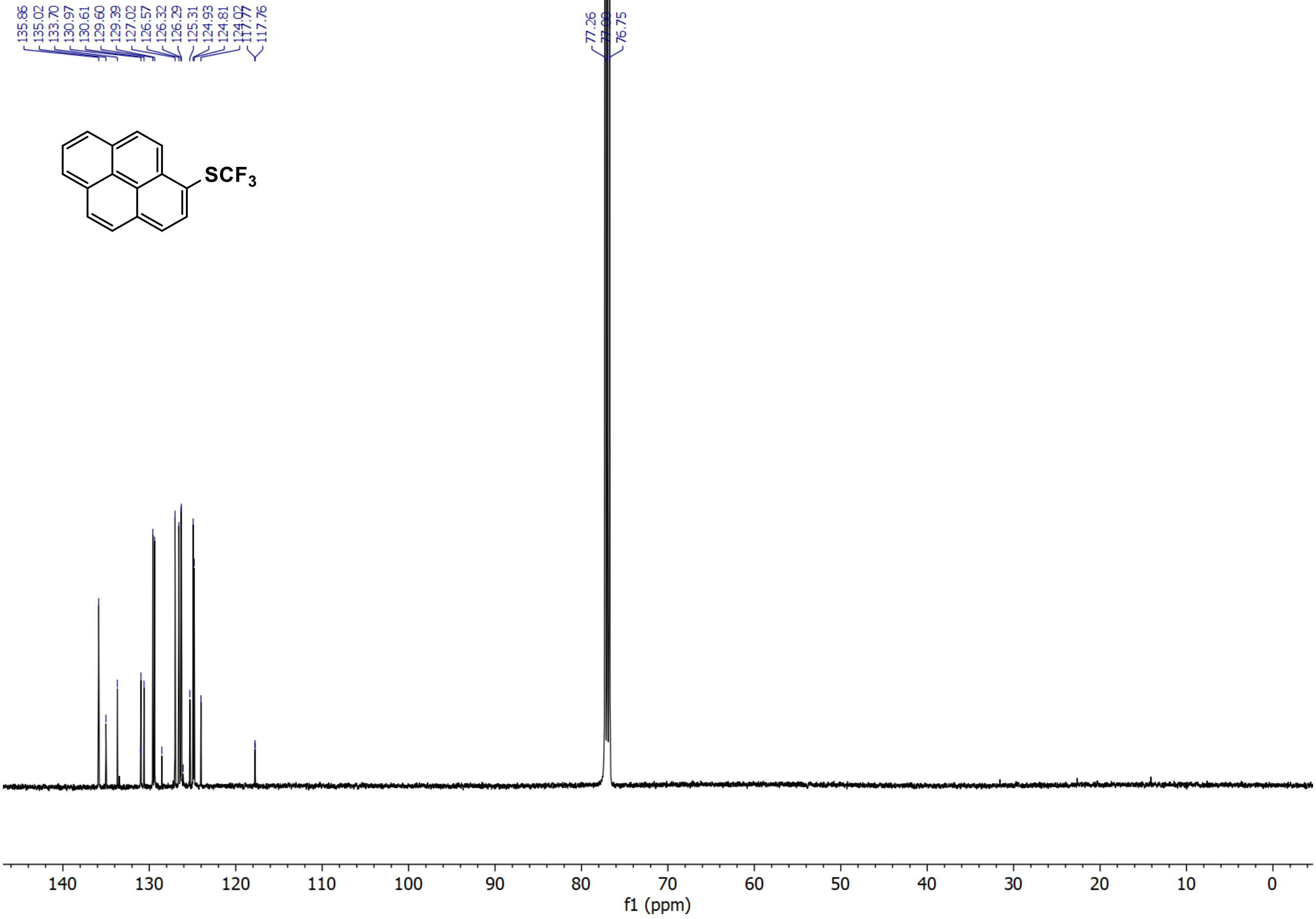

5s ^{19}F NMR (376 MHz, $CDCl_3$)

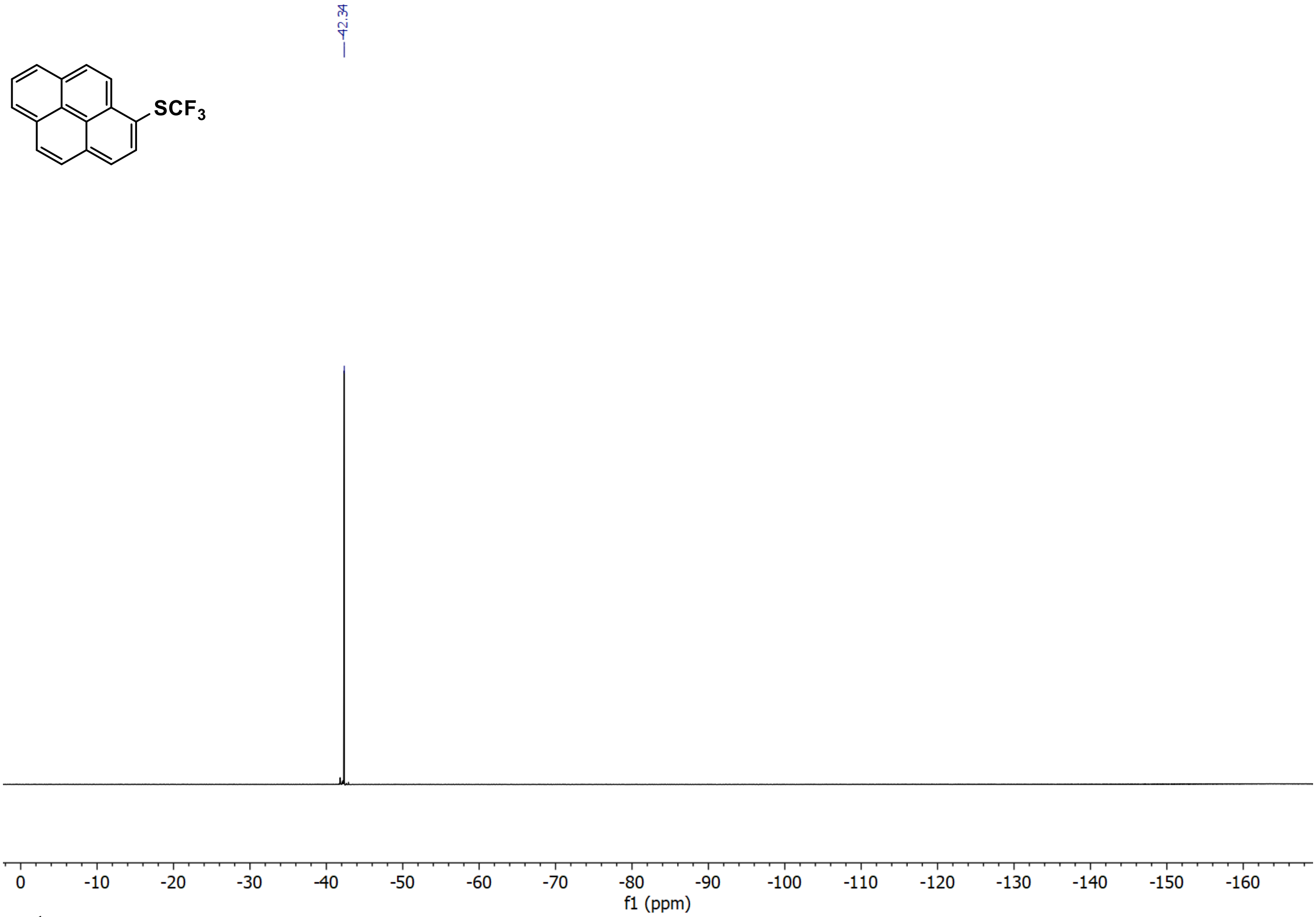

5t ^{1}H NMR (400 MHz, $CDCl_3$)

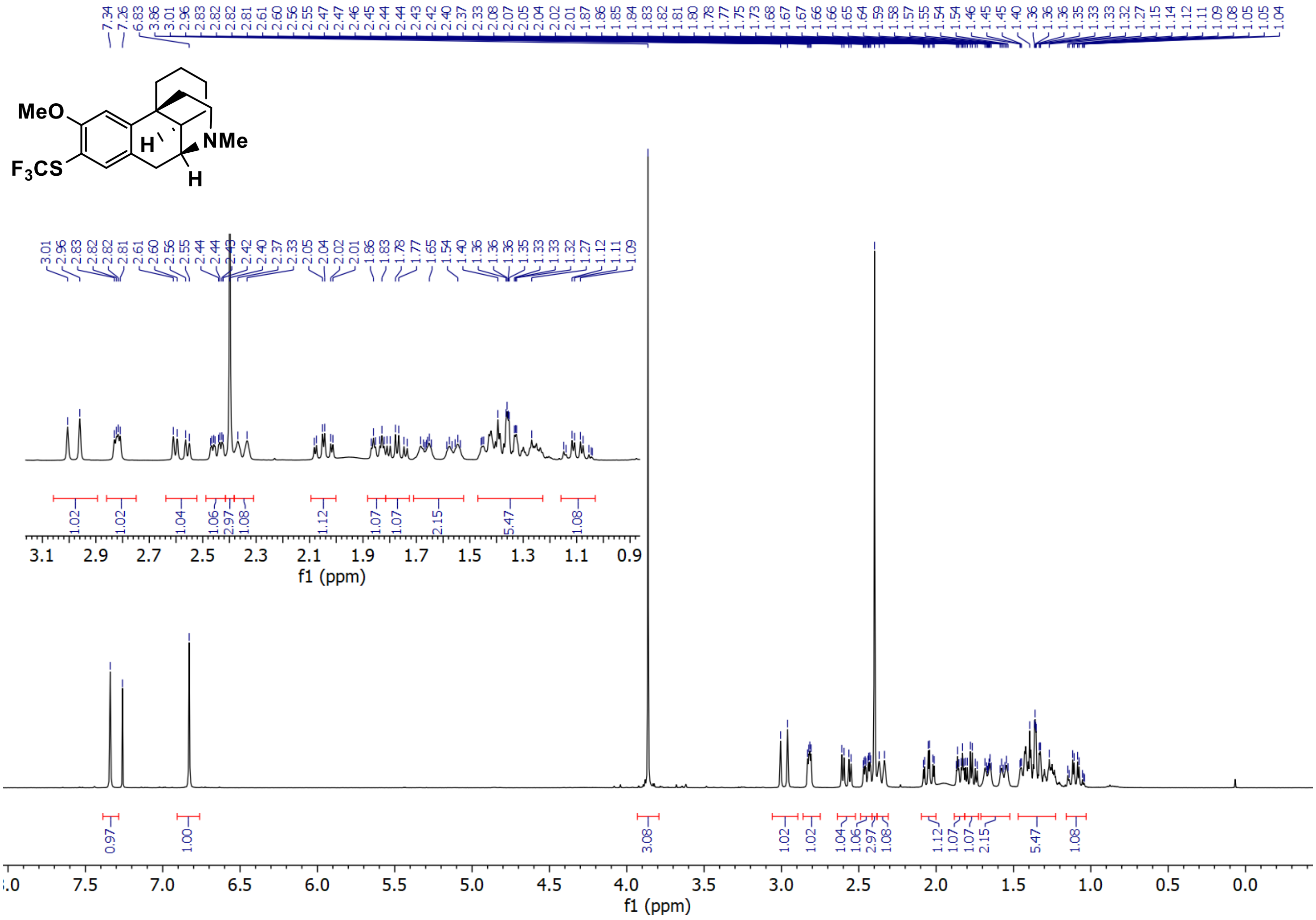

5t ^{13}C NMR (101 MHz, $CDCl_3$)

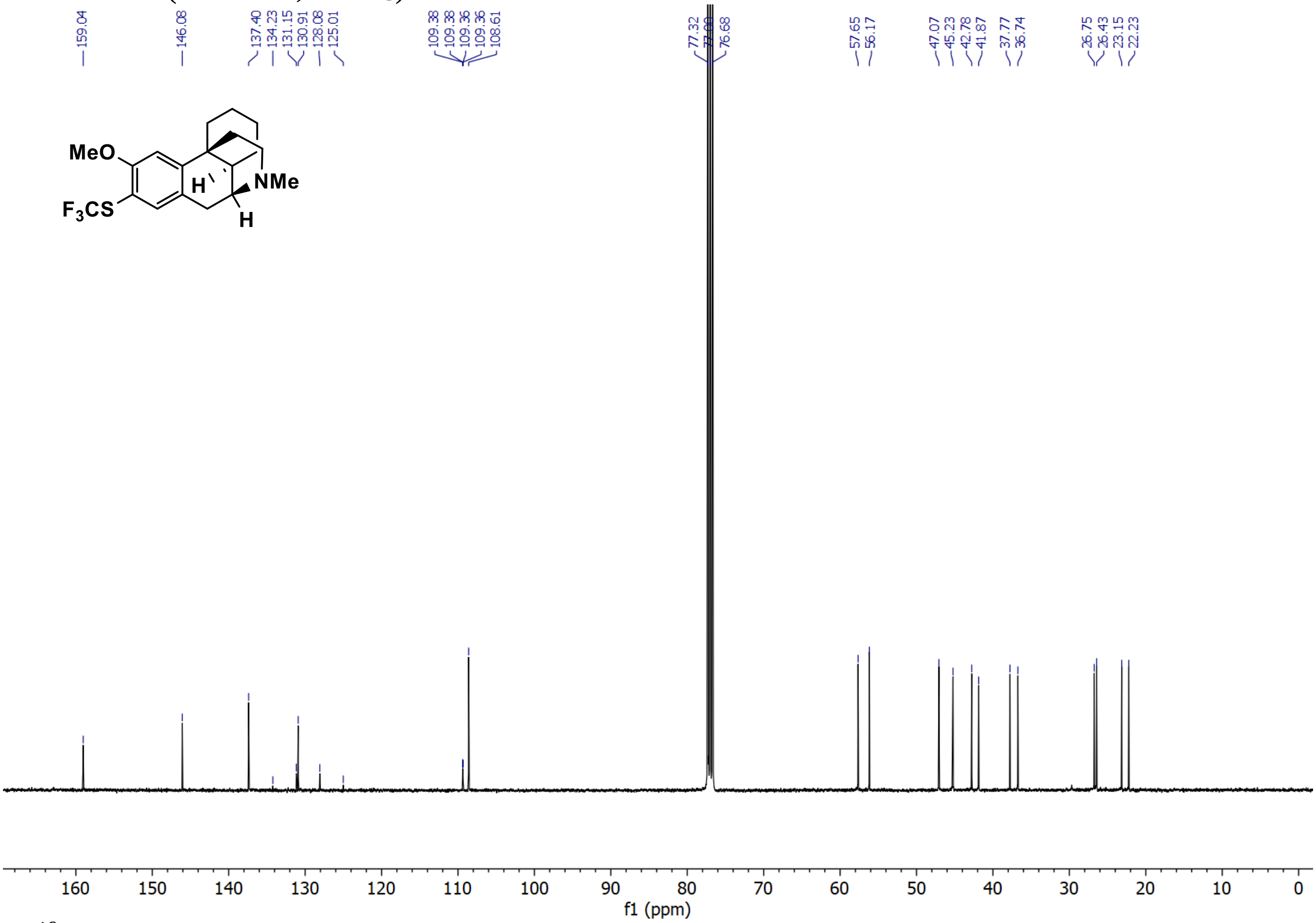

5t ^{19}F NMR (376 MHz, $CDCl_3$)

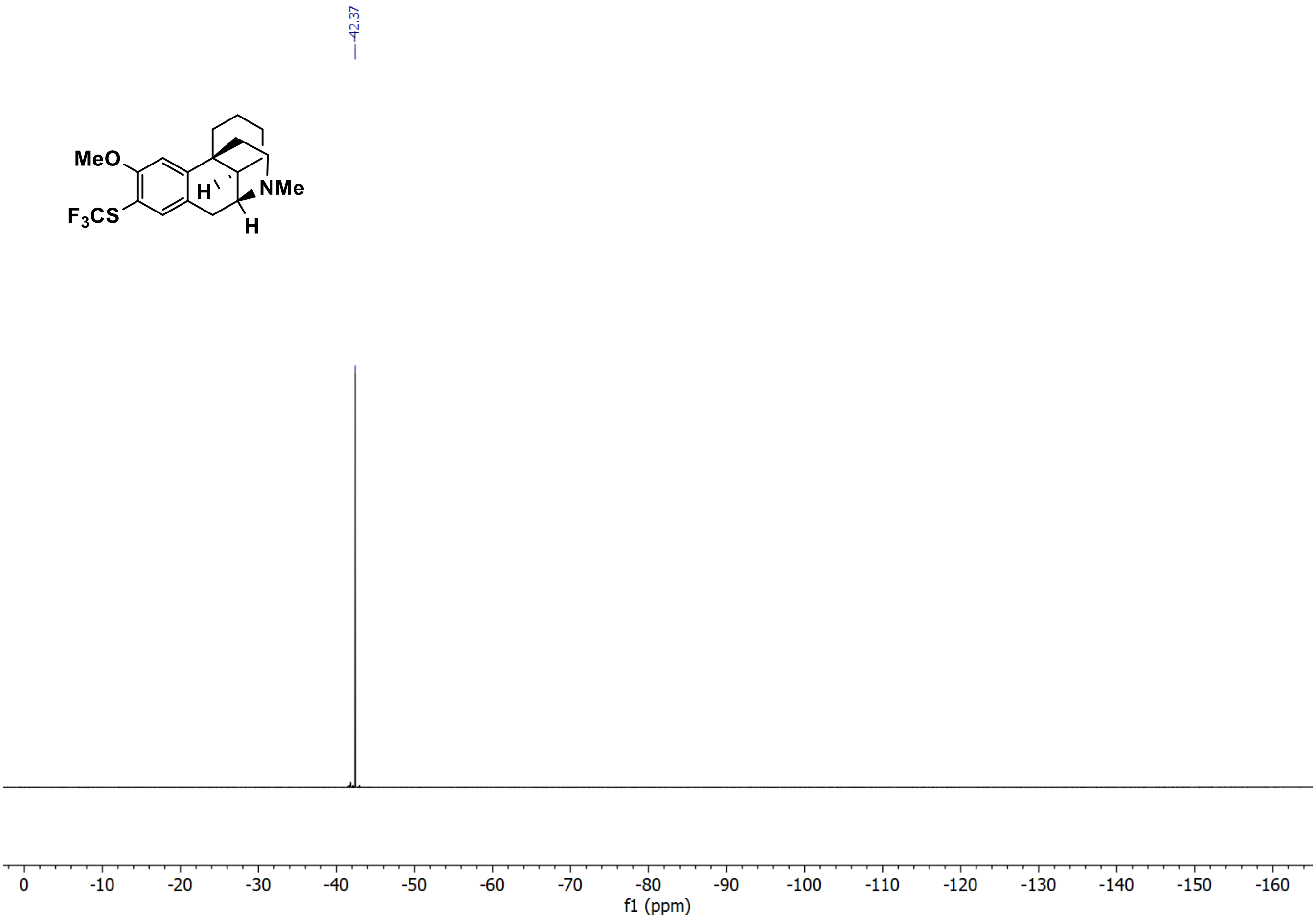

5u ^{1}H NMR (400 MHz, $CDCl_3$)

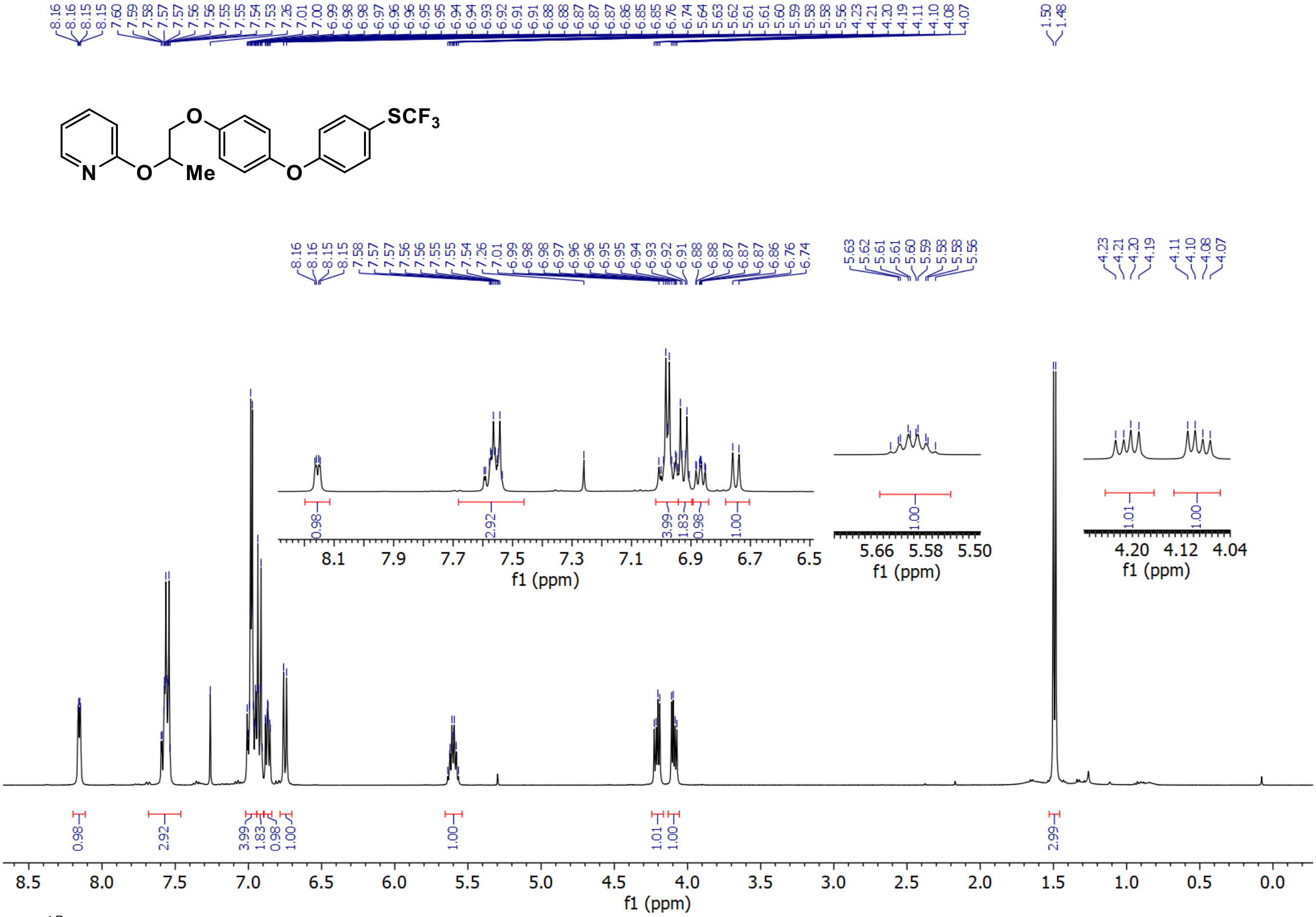

5u ^{13}C NMR (101 MHz, $CDCl_3$)

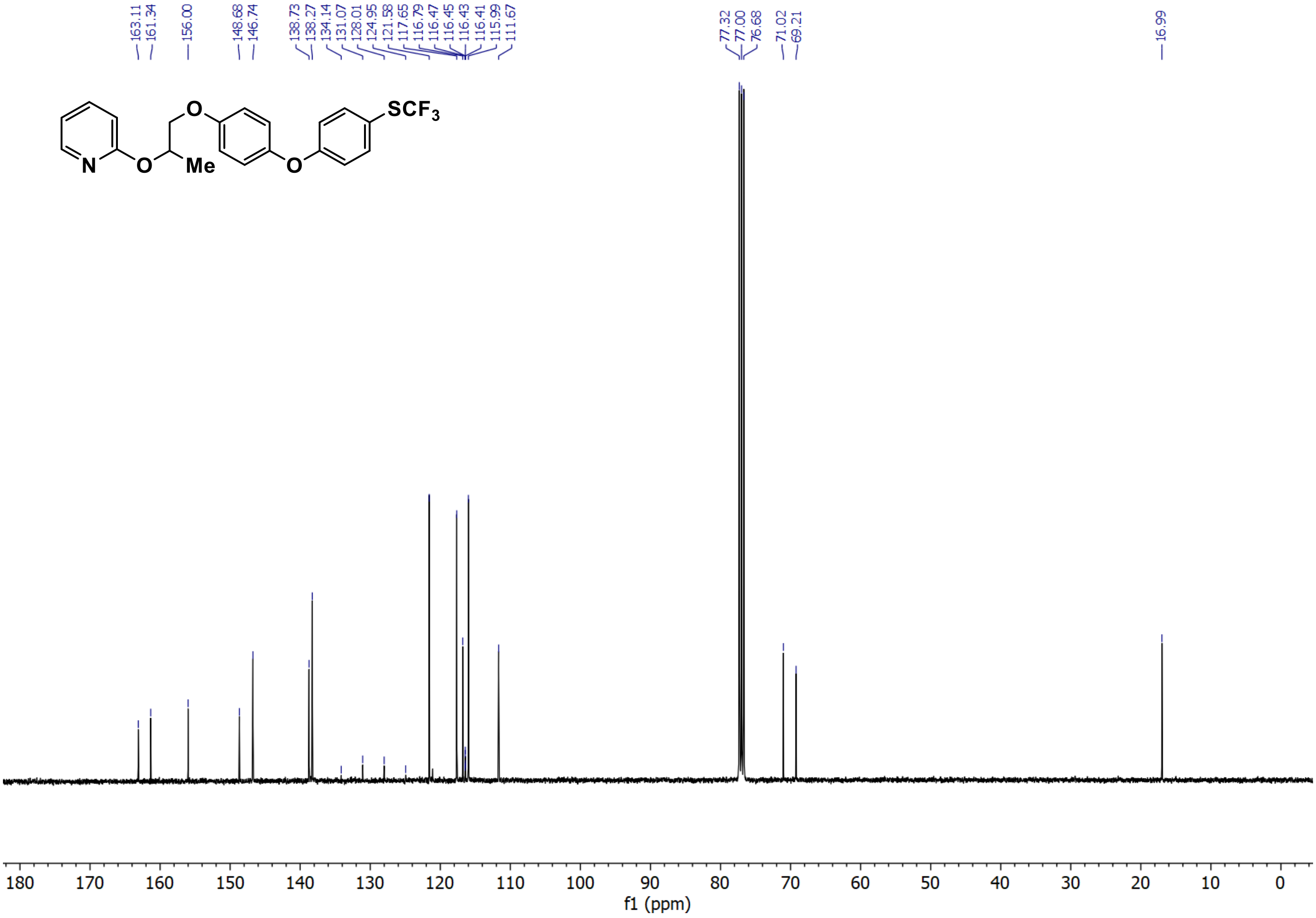

5u ^{19}F NMR (376 MHz, $CDCl_3$)

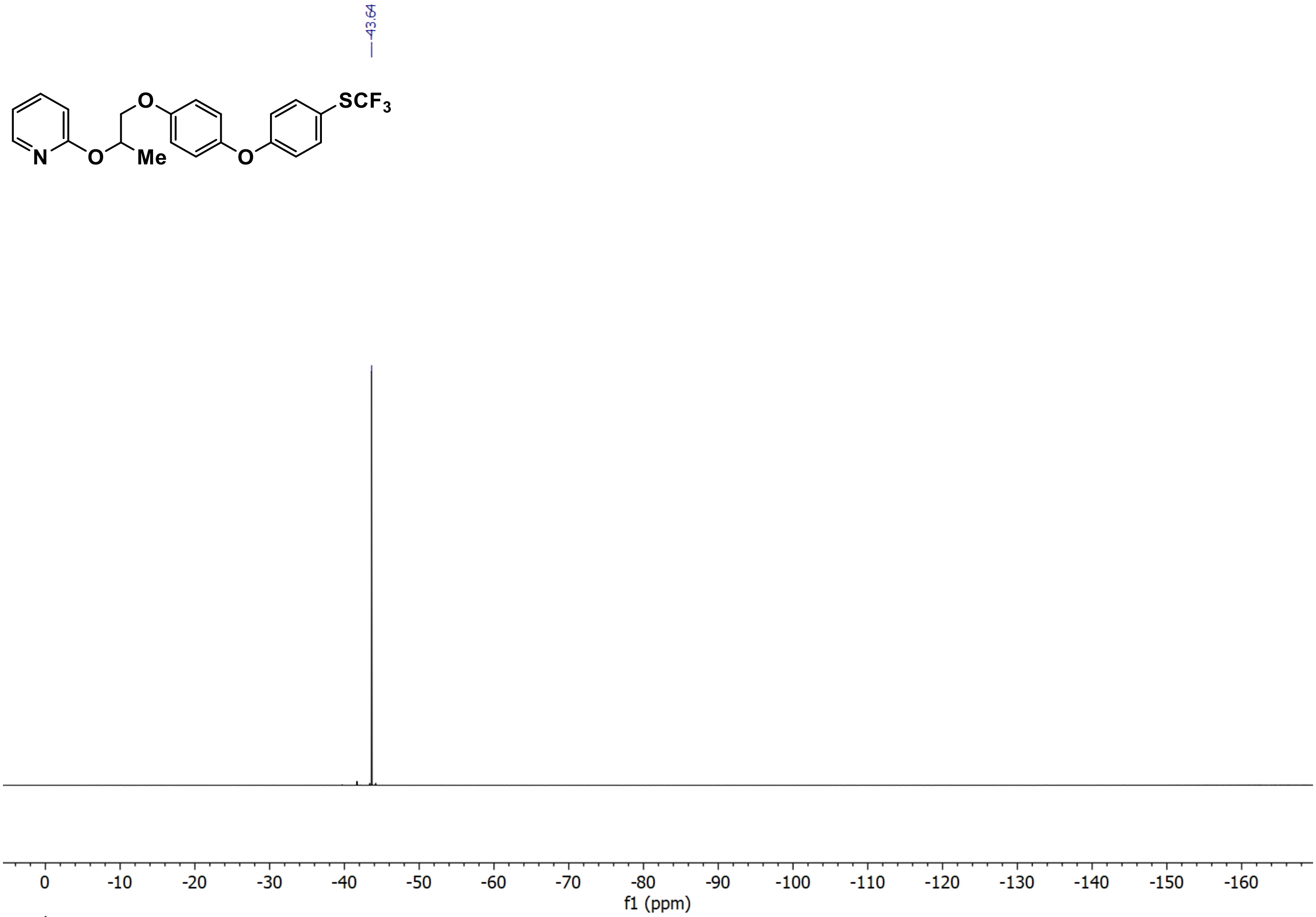

5v ^{1}H NMR (400 MHz, $CDCl_3$)

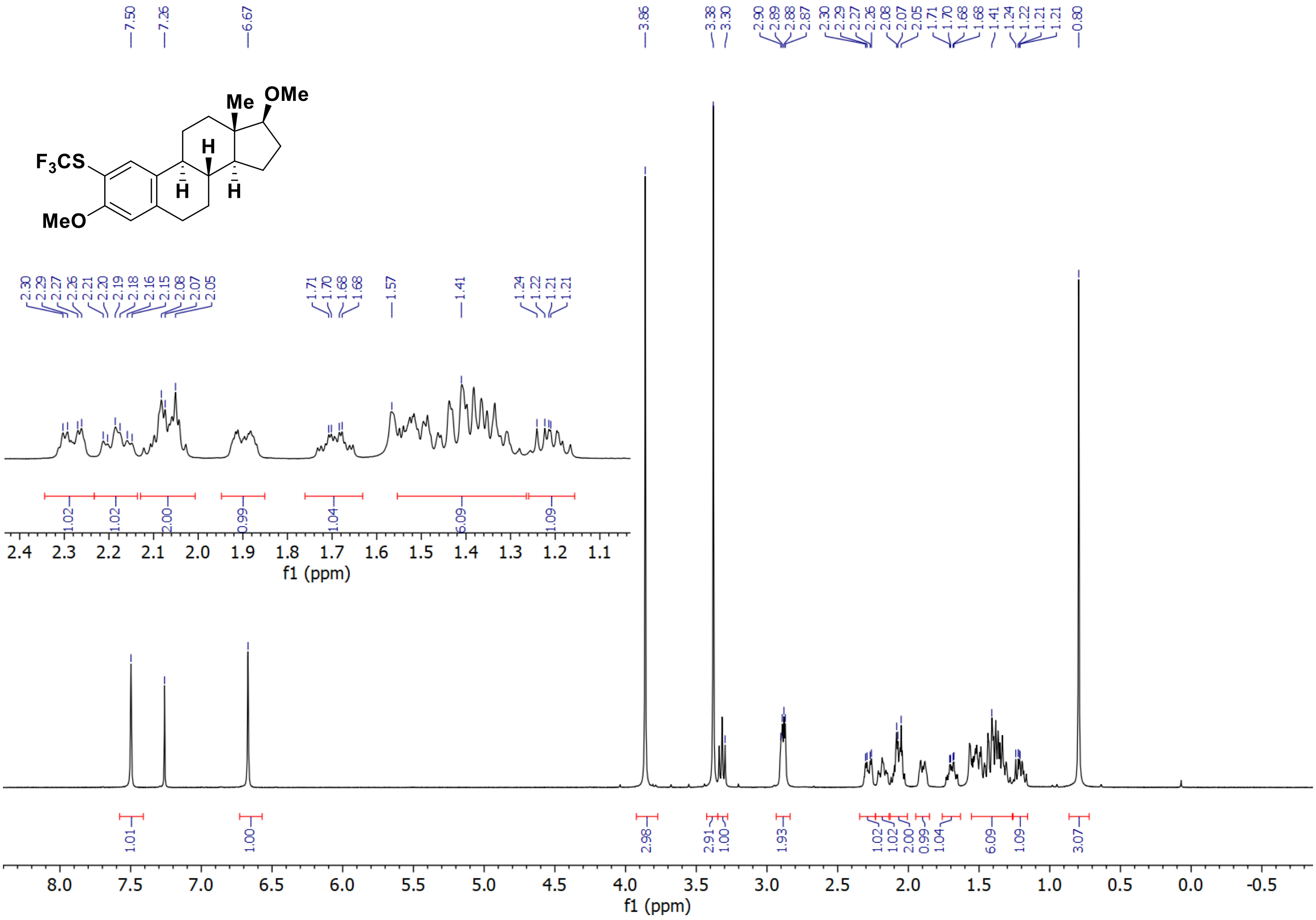

5v ^{13}C NMR (126 MHz, $CDCl_3$)

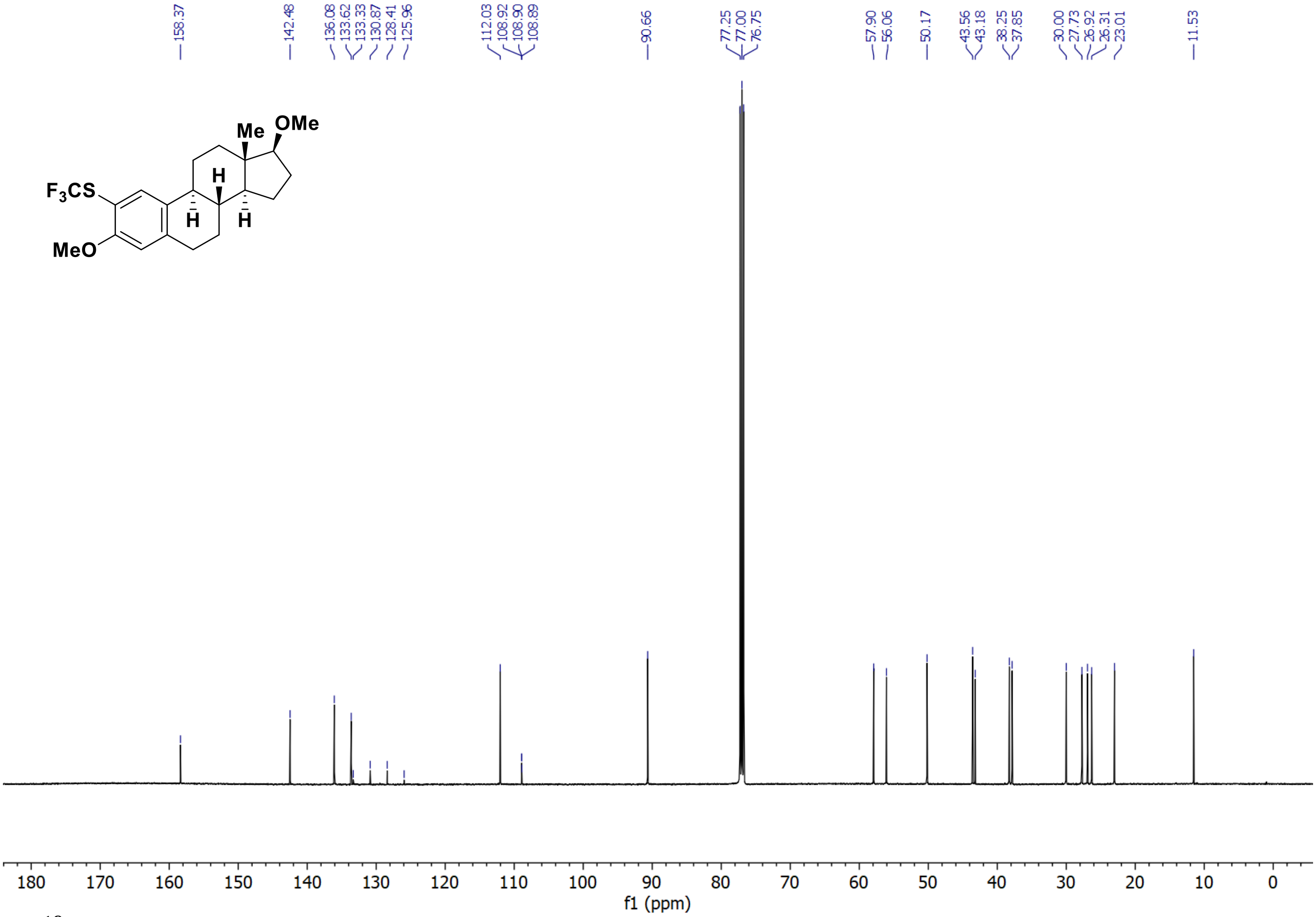

5v ^{19}F NMR (376 MHz, $CDCl_3$)

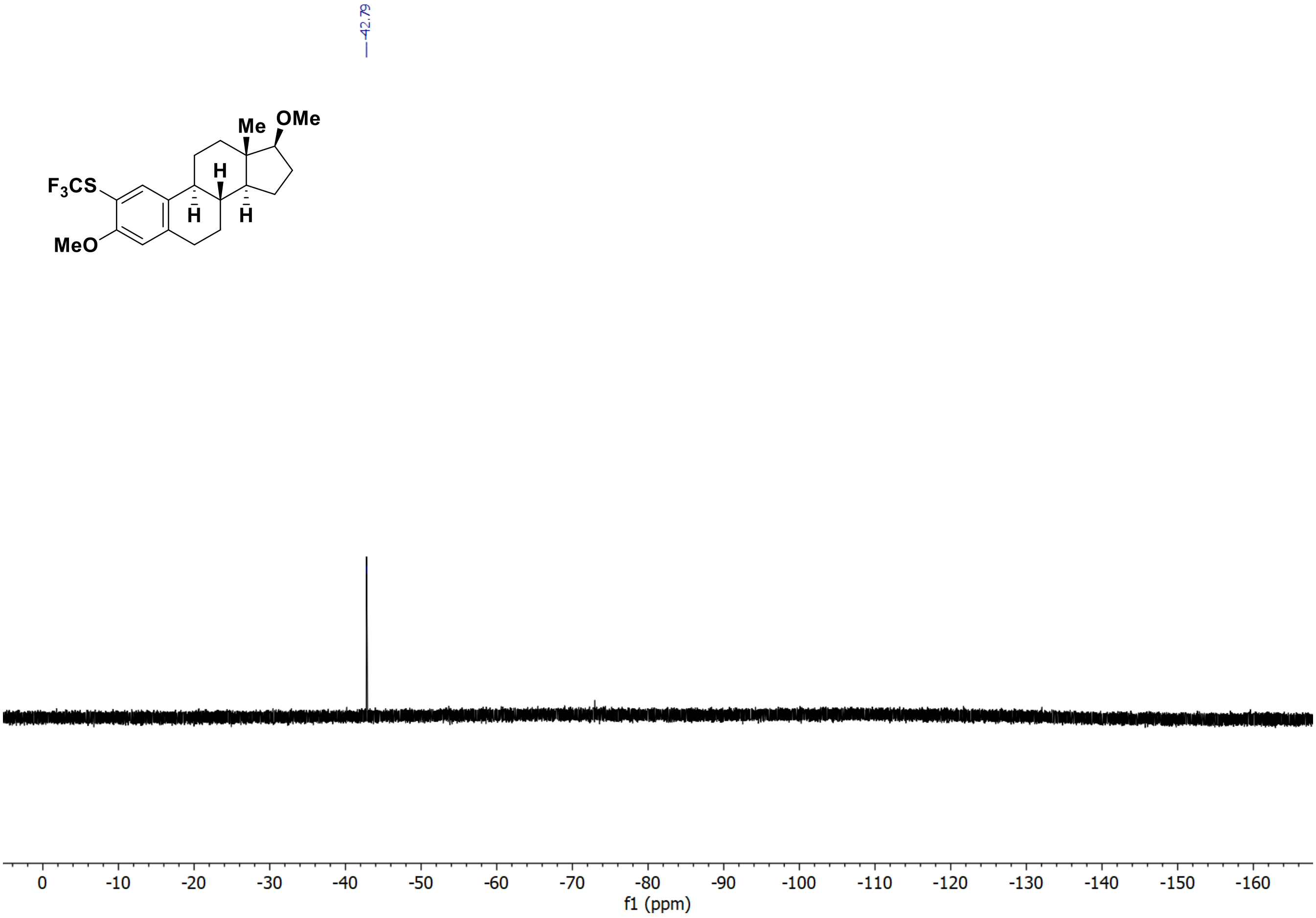

7m ^{1}H NMR (400 MHz, $CDCl_3$)

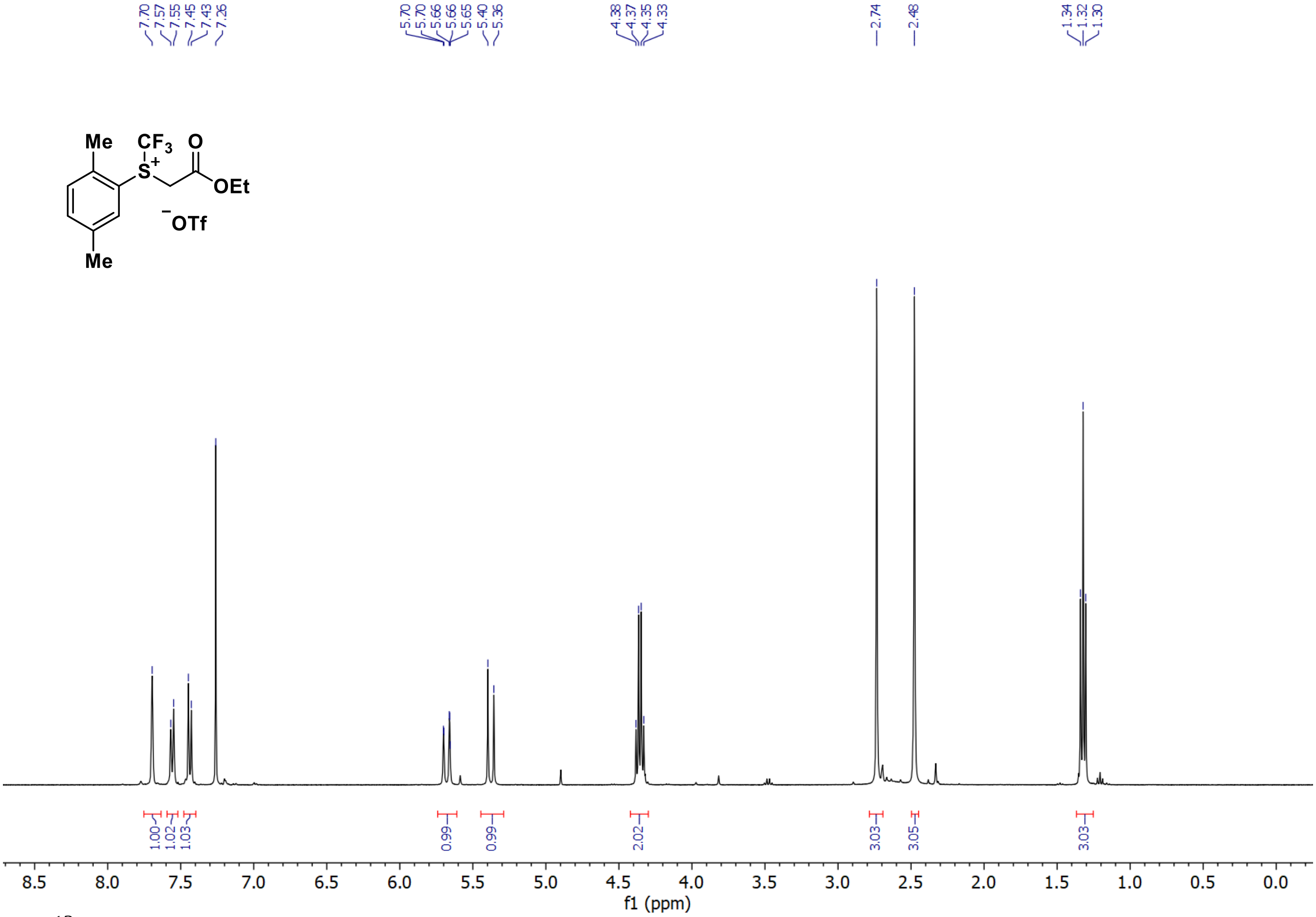

7m ^{13}C NMR (101 MHz, $CDCl_3$)

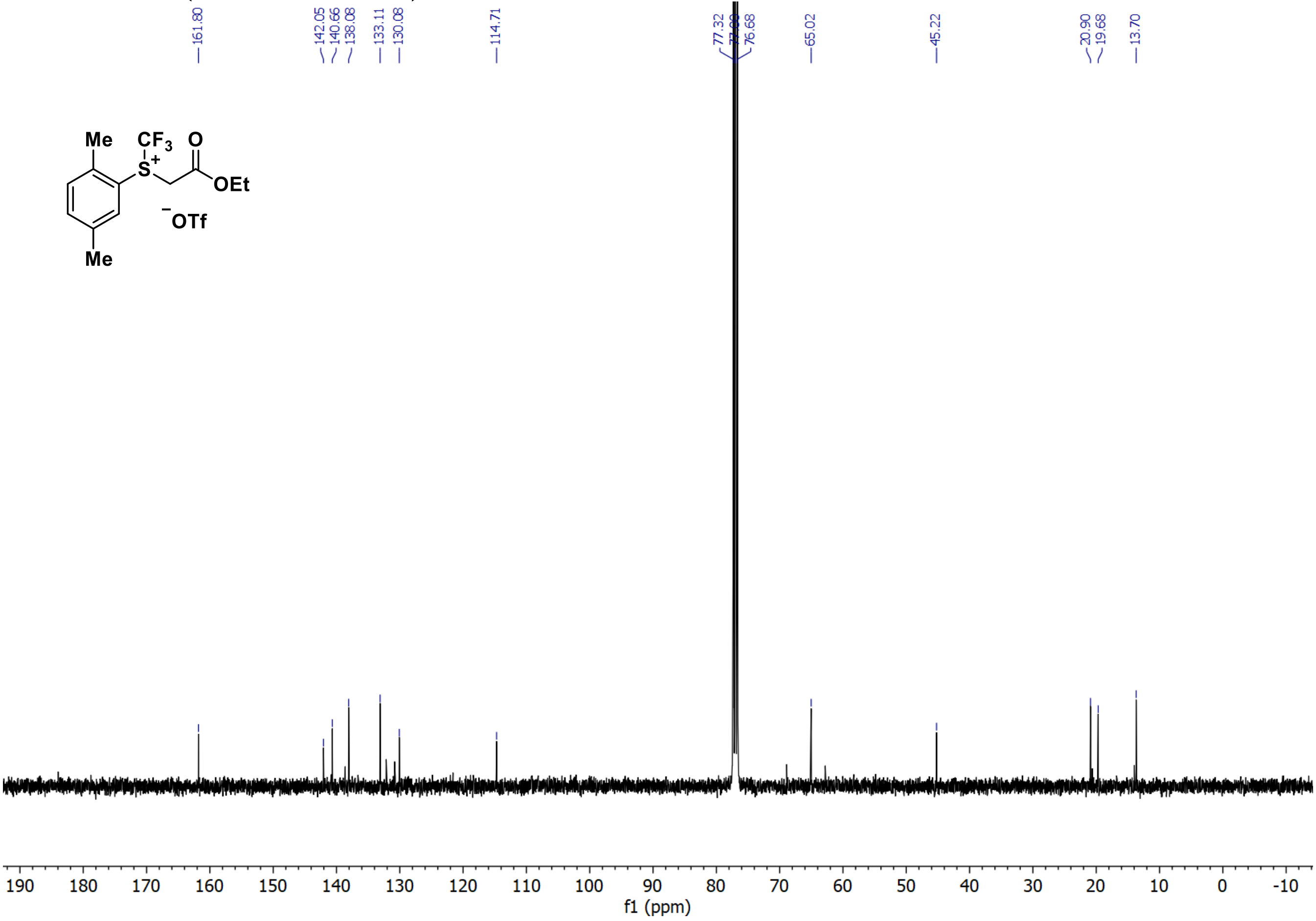

7m ^{19}F NMR (376 MHz, $CDCl_3$)

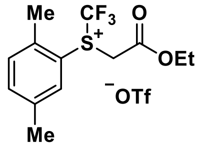

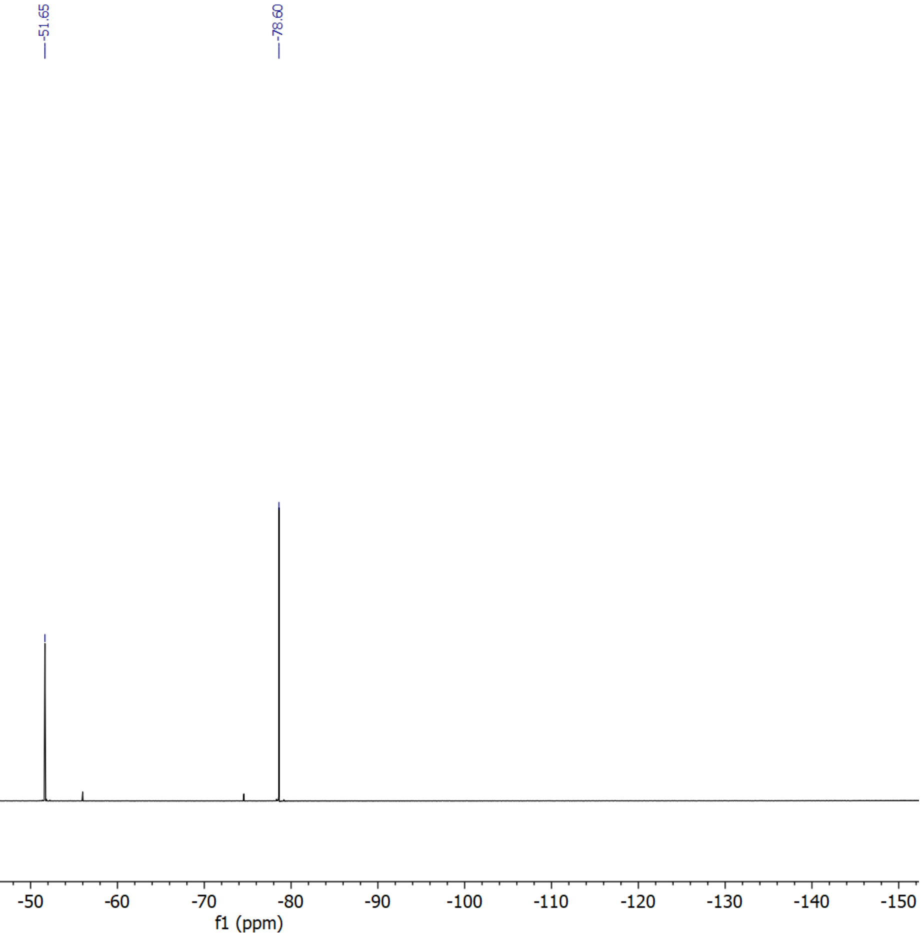

Supplement: Supplementary file 1 — Supplementary [file ANIE-59-15918-s001.pdf]
